# Multi-Step In Silico Discovery of Natural Drugs against COVID-19 Targeting Main Protease

**DOI:** 10.3390/ijms23136912

**Published:** 2022-06-21

**Authors:** Eslam B. Elkaeed, Fadia S. Youssef, Ibrahim H. Eissa, Hazem Elkady, Aisha A. Alsfouk, Mohamed L. Ashour, Mahmoud A. El Hassab, Sahar M. Abou-Seri, Ahmed M. Metwaly

**Affiliations:** 1 Department of Pharmaceutical Sciences, College of Pharmacy, AlMaarefa University, Riyadh 13713, Saudi Arabia; ikaeed@mcst.edu.sa; 2 Department of Pharmacognosy, Faculty of Pharmacy, Ain-Shams University, Abbasia, Cairo 11566, Egypt; fadiayoussef@pharma.asu.edu.eg (F.S.Y.); ashour@pharma.asu.edu.eg (M.L.A.); 3 Pharmaceutical Medicinal Chemistry & Drug Design Department, Faculty of Pharmacy (Boys), Al-Azhar University, Cairo 11884, Egypt; ibrahimeissa@azhar.edu.eg (I.H.E.); hazemelkady@azhar.edu.eg (H.E.); 4 Department of Pharmaceutical Sciences, College of Pharmacy, Princess Nourah bint Abdulrahman University, P.O. Box 84428, Riyadh 11671, Saudi Arabia; aaalsfouk@pnu.edu.sa; 5 Department of Pharmaceutical Sciences, Pharmacy Program, Batterjee Medical College, Jeddah 21442, Saudi Arabia; 6 Department of Medicinal Chemistry, Faculty of Pharmacy, King Salman International University (KSIU), South Sinai 46612, Egypt; mahmoud65582@pharm.tanta.edu.eg; 7 Department of Pharmaceutical Chemistry, Faculty of Pharmacy, Cairo University, Kasr El-Aini Street, Cairo P.O. Box 11562, Egypt; 8 Pharmacognosy and Medicinal Plants Department, Faculty of Pharmacy (Boys), Al-Azhar University, Cairo 11884, Egypt; 9 Biopharmaceutical Product Research Department, Genetic Engineering and Biotechnology Research Institute, City of Scientific Research and Technological Applications, Alexandria 21934, Egypt

**Keywords:** COVID-19, main protease, molecular fingerprints, structural similarity, docking, MD simulations, MM-PBSA

## Abstract

In continuation of our antecedent work against COVID-19, three natural compounds, namely, Luteoside C (**130**), Kahalalide E (**184**), and Streptovaricin B (**278**) were determined as the most promising SARS-CoV-2 main protease (M^pro^) inhibitors among 310 naturally originated antiviral compounds. This was performed via a multi-step in silico method. At first, a molecular structure similarity study was done with **PRD_002214**, the co-crystallized ligand of M^pro^ (PDB ID: 6LU7), and favored thirty compounds. Subsequently, the fingerprint study performed with respect to **PRD_002214** resulted in the election of sixteen compounds (**7**, **128**, **130**, **156**, **157**, **158**, **180**, **184**, **203**, **204**, **210**, **237**, **264**, **276**, **277**, and **278**). Then, results of molecular docking versus M^pro^ PDB ID: 6LU7 favored eight compounds (**128**, **130**, **156**, **180**, **184**, **203**, **204**, and **278**) based on their binding affinities. Then, in silico toxicity studies were performed for the promising compounds and revealed that all of them have good toxicity profiles. Finally, molecular dynamic (MD) simulation experiments were carried out for compounds **130**, **184**, and **278**, which exhibited the best binding modes against M^pro^. MD tests revealed that luteoside C (**130**) has the greatest potential to inhibit SARS-CoV-2 main protease.

## 1. Introduction

The WHO mentioned on 8 February 2022 that SARS-CoV-2 caused confirmed infections estimated by 396,558,014 people all over the globe and resulted in the death of an additional 5,745,032 [1]. The spreading of this notorious virus dramatically in addition to the shortage of effective treatment, mandates the utilization of new fast and efficient drug design strategies [2]. Computer-aided (computer-based, computational, or in silico) strategies in drug discovery represent quick and reliable approaches that could predict the bioactivity of any compound reducing the waste of effort, [3,4] time, and money. Computer-aided drug discovery approaches include molecular docking [5,6,7], molecular dynamic simulations [8], QSAR [9], pharmacophore modeling [10,11], ADMET [12,13], DFT [14], drug molecular design [15,16], and toxicity prediction [17,18,19]. These approaches target the enhancement of drug activity besides the discovery of new ligands [20].

Humans always depended on nature around them as the main source of food and medicine [21,22]. The compounds isolated from natural sources showed various bioactivities like anticancer [23,24,25,26,27], antileishmanial [28,29], antibacterial [30,31,32], neuro-protecting [33,34], antioxidant [35], and antiviral activity [36,37].

Viral proteases are successfully utilized as promising antiviral targets. For instance, the aspartyl protease and the serine proteases were effective targets for antivirals against human immunodeficiency virus and hepatitis C virus, respectively [38]. The vital role of M^pro^ during the replication of SARS-CoV-2 is to activate a group of sixteen functional and non-structural proteins through the separation of the two overlying polyproteins (pp1a and pp1ab). Consequently, the inhibition of M^pro^ will cause definite damage to the virus [39]. Additionally, the structure and the sequence of viral main protease (M^pro^) and human proteases are quietly different [40]. These properties suggest M^pro^ as a target for anti-COVID-19 drug discovery [41,42].

Our team used the computer-aided drug discovery approaches in the discovery of potential natural COVID-19 inhibitors several times. Four isoflavonoids with inhibitory potential against hACE2 and viral main protease have been selected among fifty-nine compounds [43]. Similarly, the anti-COVID-19 activity of fifteen guanidine alkaloids was screened in silico against five essential COVID-19 proteins [44]. Recently, our team utilized multistage in silico filtration techniques to point out the most potent natural inhibitor among a big group of compounds against certain COVID-19 enzymes. For instance, among a group of 310 natural antivirals, vidarabine was found to be the most promising natural inhibitor of SARS-CoV-2 nsp10 [45]. Similarly, the most relevant semisynthetic SARS-CoV-2 papain-like protease inhibitor has been chosen among 69 candidates [46].

**PRD_002214**,*N*-[(5-Methylisoxazol-3-yl)carbonyl]alanyl-l-valyl-*N*~1~-((1R,2Z)-4-(benzyloxy)-4-oxo-1-{[(3R)-2-oxopyrrolidin-3-yl]methyl}but-2-enyl)-l-leucinamide, also called inhibitor N3, is an irreversible peptide-like inhibitor of the main protease (MPRO) of SARS-CoV-2. The chemical structure of **PRD_002214** was obtained from the RCSB Protein Data Bank entry 6LU7 which shows the ligand in complex with the main protease [47].

**Figure 1:**
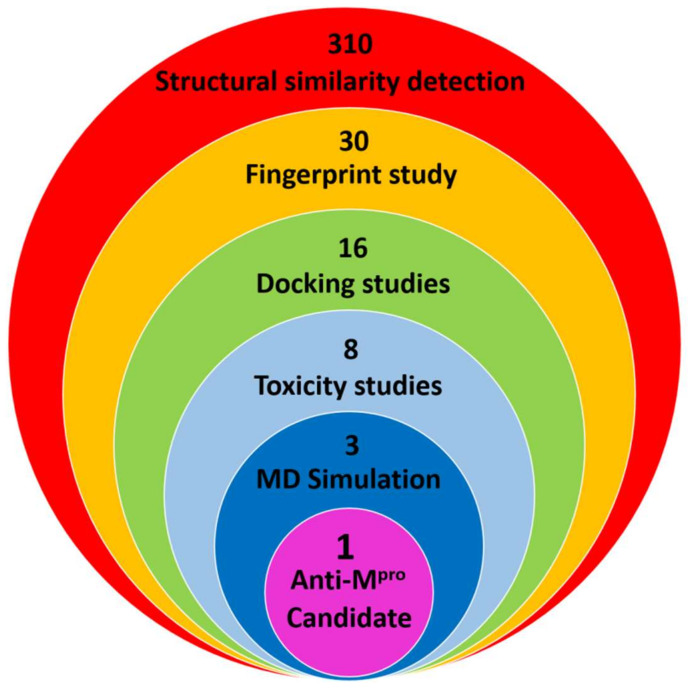
The utilized computational methods.

In this work, a collection of 310 naturally originated antiviral compounds has been screened using different computational methods to detect the most potent naturally derived M^pro^ inhibitor. The utilized methods included molecular structures similarity study with **PRD_002214**, a fingerprint study against **PRD_002214**, the molecular docking against M^pro^ PDB ID: 6LU7, in silico toxicity studies, and molecular dynamic (MD) simulation experiments (Figure 1).

## 2. Results and Discussion

### 2.1. Structural Similarity Detection

Structural similarity is a computational method that identifies the similarity of two compounds based on structural molecular properties (descriptors) [48]. Recently, this method has become a considerable and effective method in the field of drug design [49]. The applied molecular descriptors included hydrogen bond donors (HBA) [50], hydrogen bond acceptors (HBD) [51], partition coefficient (ALog *p*) [52], molecular weight (M. Wt) [53], molecular fractional polar surface area (MFPSA) [54], and number of rotatable bonds [55], rings, and aromatic rings [56].

**Figure 2:**
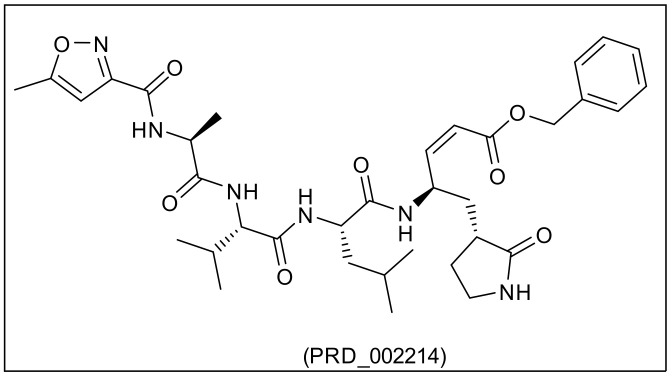
The chemical structure of **PRD_002214**.

The degree of molecular similarity between two molecules depends on the similarity coefficient (metric) which is used to compute a quantitative score for the degree of similarity based on the weighted values of structural descriptors. The similarity between two molecules is the inverse function of the distance between them in descriptor space [57]. When there are two or more reference ligands, the shortest distance to a reference ligand is used. In this work, Euclidean distances between the rank-ordering of different descriptors are calculated to determine descriptor similarity where Euclidean distances represent the shortest distance between two points [58]. Structural similarity studies between the 310 antiviral compounds (Supplementary Materials Figure S1) and the co-crystallized ligand **PRD_002214** (Figure 2) of M^pro^ PDB ID: 6LU7 have been applied by the software Discovery Studio depending on the previous descriptors.

**Figure 3:**
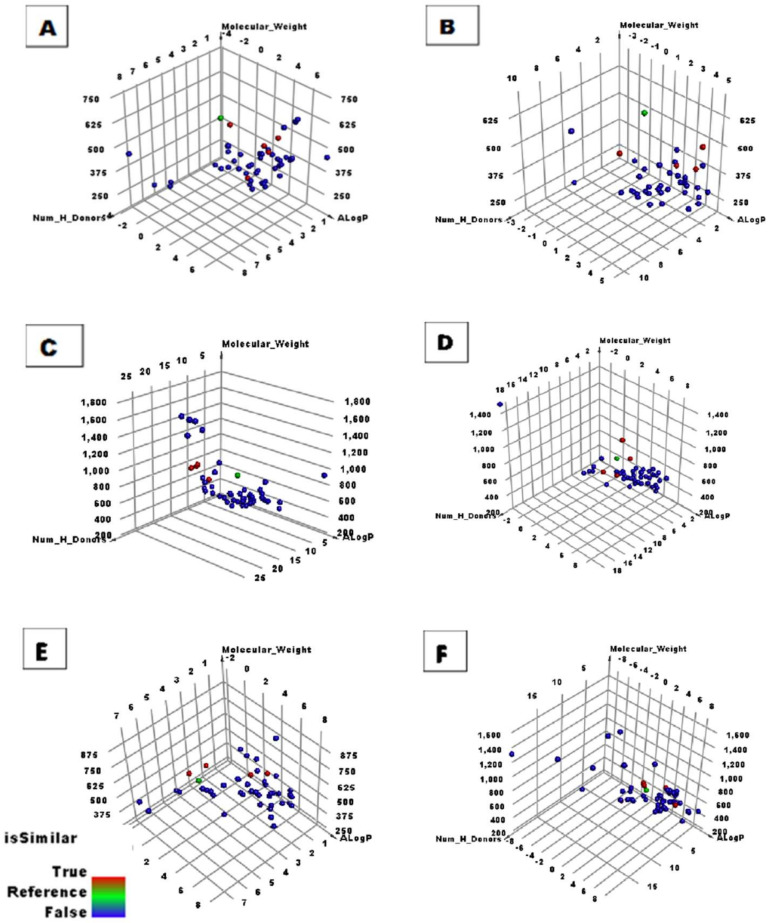
Results of the structural similarity of the antiviral compounds and **PRD_002214**. The green sphere is **PRD_002214**, the red sphere is a similar compound, and the blue sphere is a dissimilar compound. (**A**) first 50 compounds, (**B**) second 50 compounds, (**C**) third 50 compounds, (**D**) fourth 50 compounds, (**E**) fifth 50 compounds, and (**F**) sixth 60 compounds.

**Figure 4:**
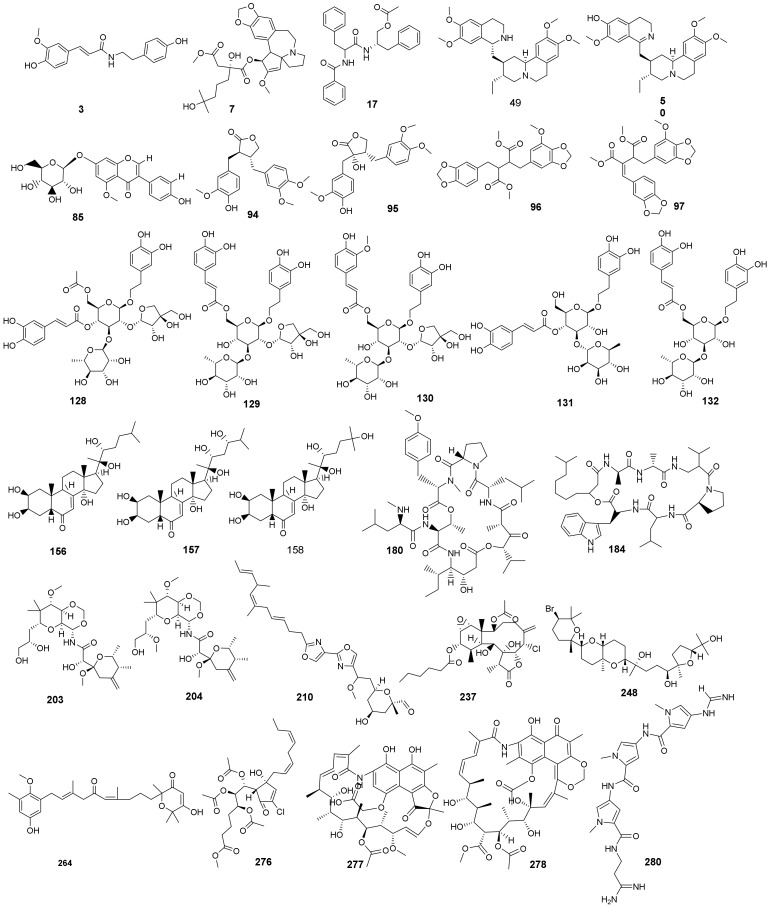
The filtered 30 compounds obtained from the molecular similarity technique.

The antiviral compounds were examined in six groups (Figure 3) and a similarity check was performed for each group separately using **PRD_002214** as a reference. The distance between **PRD_002214** and the tested compounds is illustrated in Figure 3. The results favored thirty compounds that have good structural similarity with **PRD_002214** (Figure 4). The values of molecular properties for compounds are listed in Table 1.

The selected 30 antiviral compounds are isolated from different natural sources including plants, marine organisms, and microbes, and were reported to exhibit promising antiviral activities. Sources and antiviral potentialities of the selected compounds are summarized in Table 2.

### 2.2. Structural Fingerprint Study

The fingerprints technique was applied using Discovery Studio software to identify the molecular structures (2D) of the similar 30 antiviral compounds in a binary format against **PRD_002214**. The examined descriptors were HBA and HBD [106], charge [107], hybridization [108], positive and negative ionizable groups [109], halogens, aromatics, or none of the above, and the ALogP category [110] of atoms. The study (Table 3) showed that the antiviral compounds **7**, **128**, **130**, **156**, **157**, **158**, **180**, **184**, **203**, **204**, **210**, **237**, **264**, **276**, **277**, and **278** were the most favorite.

### 2.3. Docking Studies

Docking studies were proceeded to inspect the binding free energies (∆G) and the binding modes [111,112,113,114,115,116] of the antiviral compounds against M^pro^ PDB ID: 6LU7 (Table 4) with **PRD_002214** as a reference. Eight compounds (**128**, **130**, **156**, **180**, **184**, **203**, **204**, and **278**) exhibited the most a-like binding mode and the highest binding energies.

**Figure 5:**
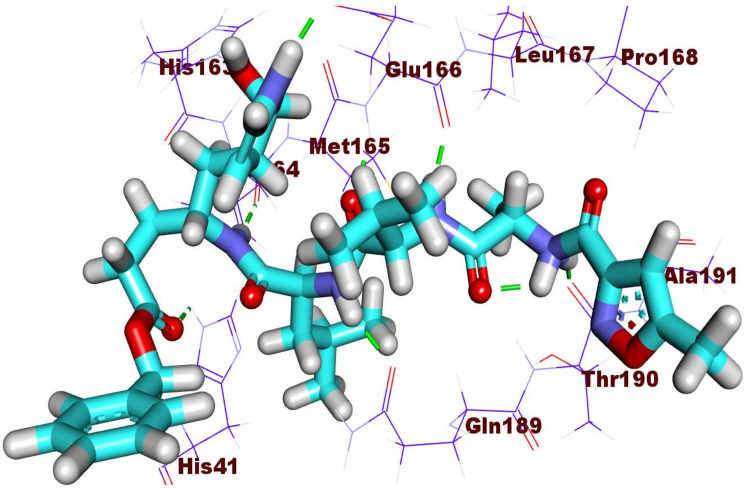

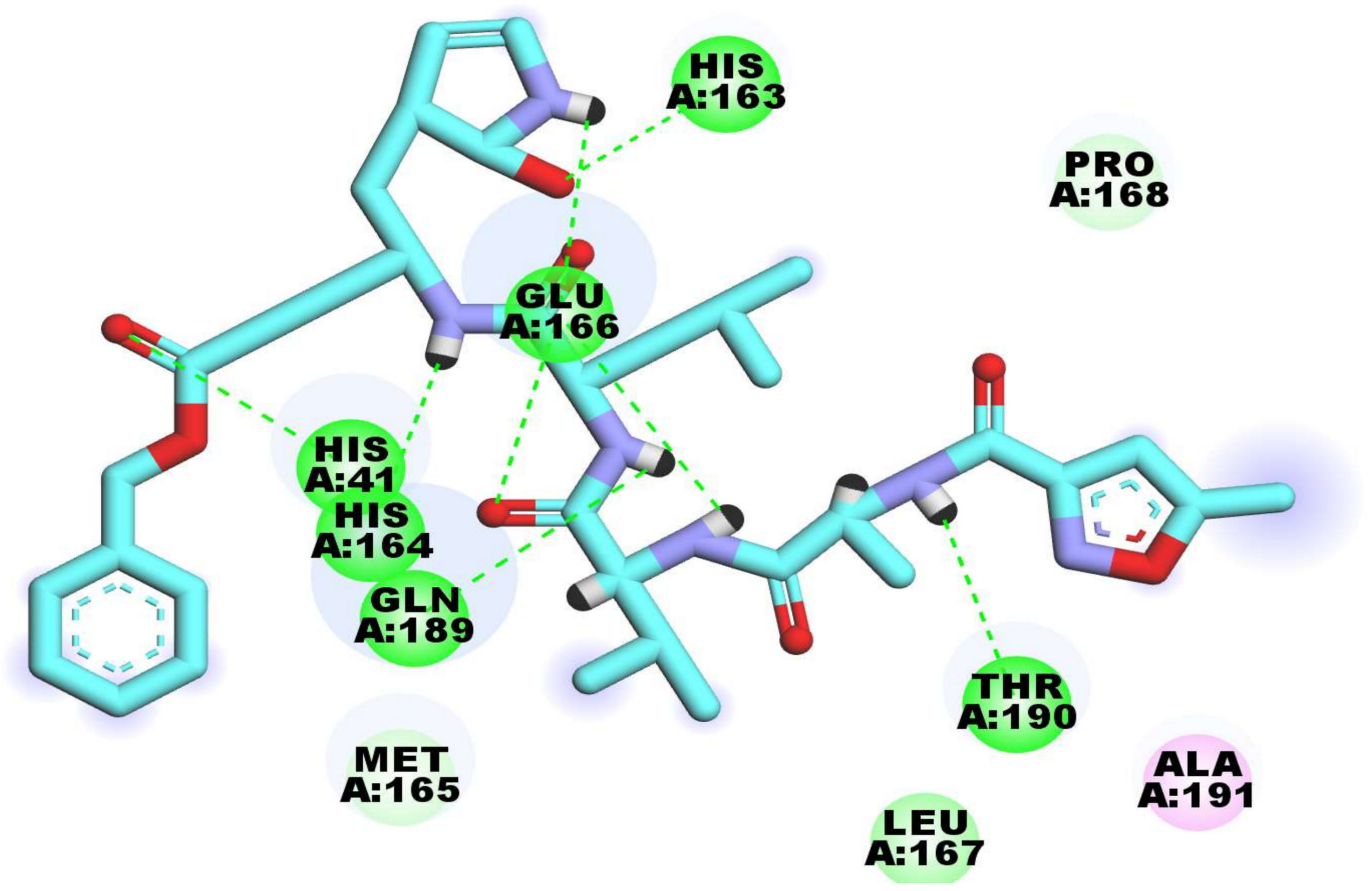
The binding pattern of **PRD_002214** inside the 6LU7 active site of M^pro^ PDB ID: 6LU7 active site.

Starting with **PRD_002214**, it showed eight hydrogen bonds in addition to four hydrophobic reactions. In detail, the first pocket of M^pro^ was occupied by the 2-oxopyrrolidin-3-yl moiety that was involved in two hydrogen-bonding interactions with His163 and Glu166. The 2-acetamido-3-methylbutanamido)-*N*-ethyl-4-methyl pentanamide moiety was buried in the second pocket with four hydrogen-bonding interactions with Gln189, Glu166, and Thr190 together with three hydrophobic interactions with Met165 and His41. The benzyl acetate moiety was suited in the third pocket of the receptor engaging in two hydrogen bonds with His164 and His41. Moreover, the 5-methylisoxazole-3-carboxamide moiety occupied the fourth pocket forming a hydrophobic interaction with Ala191 (Figure 5) [117].

**Figure 6:**
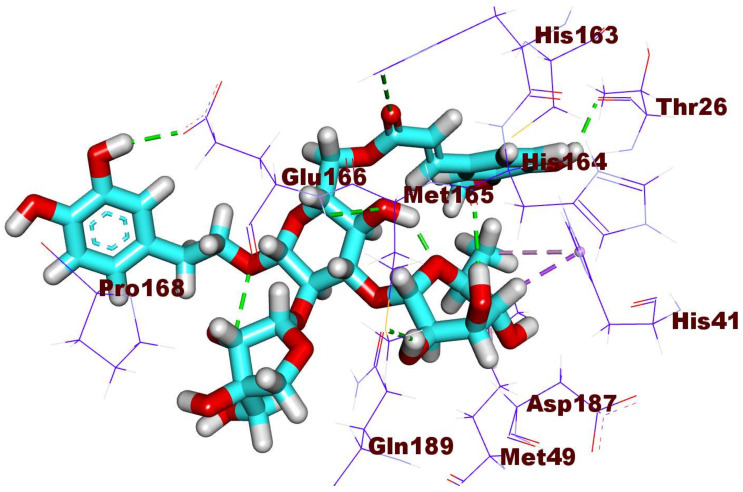

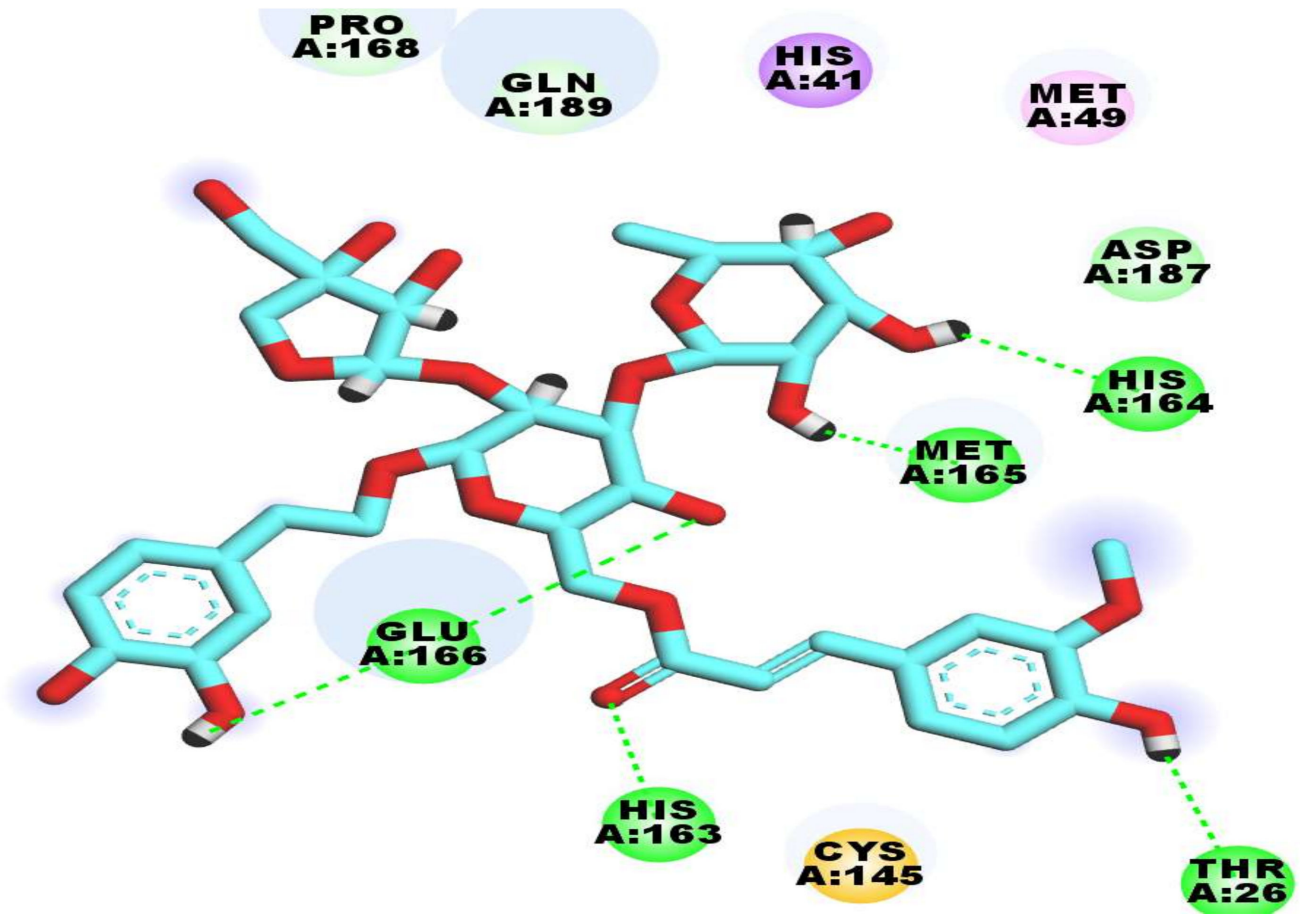
The binding pattern of compound **130** inside the M^pro^ PDB ID: 6LU7 active site.

The antiviral compound, **130** was engaged in six hydrogen bonds, three hydrophobic interactions, and one electrostatic attraction. Firstly, the methyl-3-(4-hydroxy-3-methoxyphenyl)acrylate moiety was fitted in the first pocket making two hydrogen bonds with His 163 and Thr26 and one electrostatic interaction with Cys145. Furthermore, the 6-methyltetrahydro-2*H*-pyran-3,4,5-triol moiety was engaged in two hydrogen-bonding interactions inside the second pocket with His 164 and Met165 in addition to hydrophobic interactions (three) with Met49 and His41. Additionally, compound **130** was involved in two hydrogen bonds with Glu166 in the third pocket. Finally, the (3R,4R)-3-(hydroxymethyl)tetrahydrofuran-3,4-diol was buried in the fourth pocket (Figure 6).

**Figure 7:**
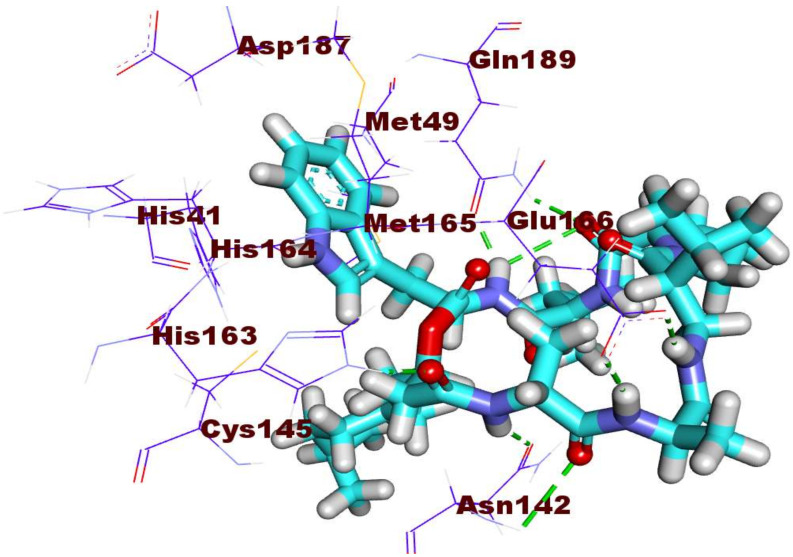

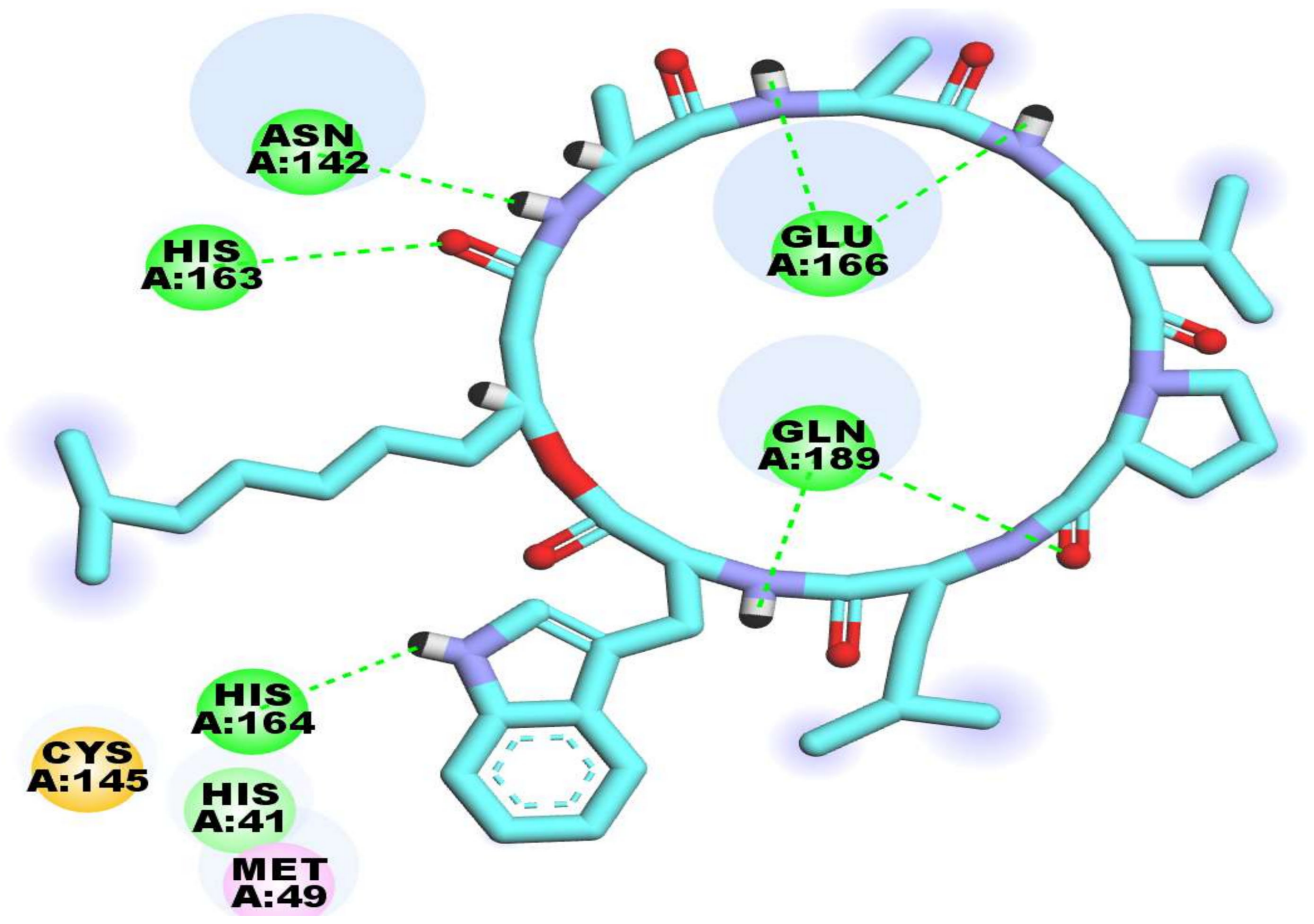
The binding pattern of compound **184** inside the M^pro^ PDB ID: 6LU7 active site.

The antiviral compound **184** (affinity value of −30.15 kcal/mol) revealed the engagement in many H-bonding as well as hydrophobic interactions in the different pockets of the main protease active pocket. At first, the 2-acetamido-*N*-((1-(isopentylamino)-1- oxopropan-2-yl)propanamide moiety was involved in four hydrogen bonds with Glu166, Asn142, and His 163 in the first pocket. Likely, the 2-formamido-*N*,4-dimethyl pentanamide moiety made two hydrogen bonds with Gln189 in the second pocket. Moreover, compound **184** was buried in the second pocket through the formation of one hydrogen bond with the amino acid Met165 and two hydrophobic interactions with the amino acid Met49 using its 1*H*-indole moiety (Figure 7).

**Figure 8:**
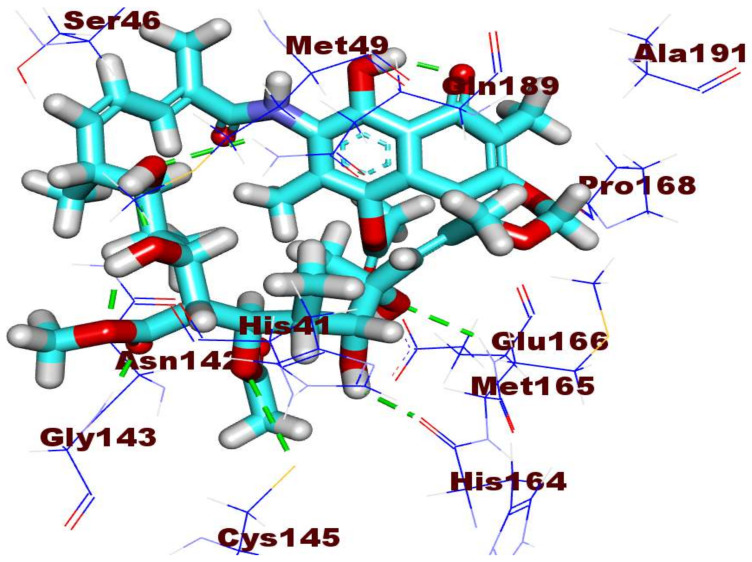

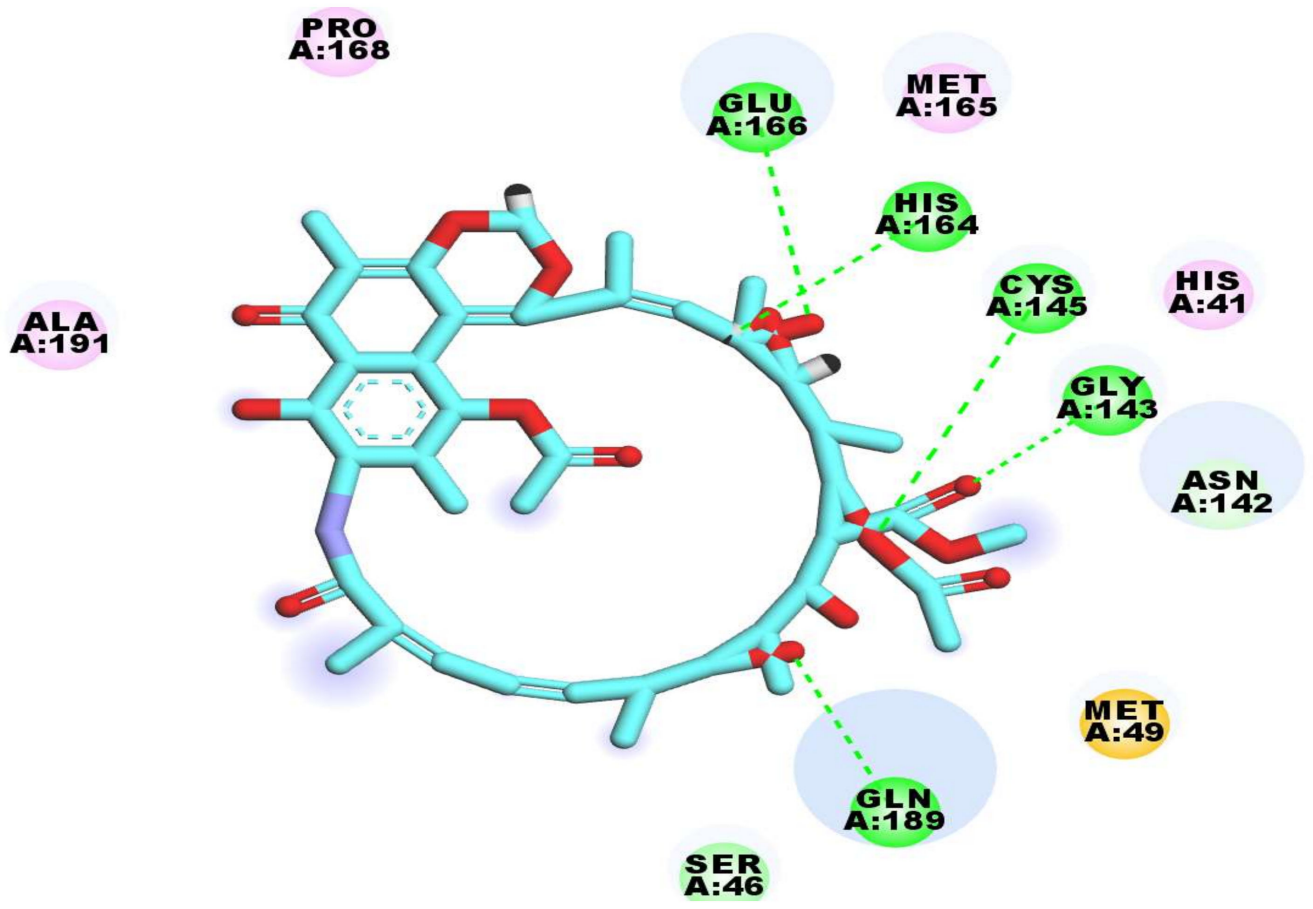
Binding mode of compound **278** inside the active site of M^pro^ PDB ID: 6LU7.

The antiviral compound **278** exhibited a ∆G of −29.00 kcal. mol^−1^. It was combined with receptor protein through five hydrogen bonds and four hydrophobic interactions as shown in Figure 8. Basically, the 7-hydroxy-5,9-dimethyl-6*H*-naphtho [2,1-*d*][1,3]dioxin -6-one occupied the first pocket of COVID-19 main protease with two hydrophobic interactions with Pro168 and Ala191. The macrocyclic structure of the tested compounds occupied the other three pockets of the target receptor engaging in five hydrogen bonds with Glu166, His164, Cys145, Gly143, and Gln189. Moreover, it formed two hydrophobic interactions with Met165 and His41.

The detailed binding modes of compounds **128**, **156**, **180**, **203**, and **204** are displayed in the Supplementary Materials (Figures S2–S6, respectively).

### 2.4. Toxicity Models

In this experiment, the toxicity profiles of the favored eight antiviral compounds (**128**, **130**, **156**, **180**, **184**, **203**, **204**, and **278**) were examined by seven toxicity models (illustrated in Table 5) in the Discovery Studio software version 4.0 [118,119].

All the examined antiviral compounds were estimated as non-carcinogenic in the FDA rodent carcinogenicity model. Additionally, all antiviral compounds except **130** showed TD_50_ values more than simeprevir where the values were ranging from 2.871 to 12.946 g/kg body. Furthermore, all antiviral compounds except **180** showed rat maximum tolerated dose values higher than that of simeprevir, the values were ranging from 0.018 to 2.382 g/kg body weight. Compounds **128**, **130**, **156**, **180**, **184**, and **204** revealed oral LD_50_ values in a range of 0.274 to 10.020 g/kg body weight, higher than that of simeprevir (0.209 g/kg body weight). On the other hand, compounds **203** and **278** showed oral LD_50_ values of 0.141 and 0.166 g/kg body weight, respectively which were lower than that of simeprevir. Compounds **128**, **130**, **156**, and **184** showed LOAEL values ranging from 0.012 to 0.040 g/kg body weight while simeprevir exhibited 0.002 g/kg body weight. Finally, all the antiviral compounds showed mild to moderate irritancy except **184** which showed no irritancy in both models (Table 5).

### 2.5. Molecular Dynamics

Molecular dynamics (MD) simulation has provided many valuable insights into the binding of drugs to their targets. This includes accurate evaluation of the binding strength between a ligand and its target, studying the nature of macromolecules, and characterizing the effect of certain mutations on the resistance profile of many drugs [120,121]. In this test, three compounds (**130**, **184**, and **278**) that exhibited good binding mode against M^pro^ were nominated for MD simulation studies.

#### 2.5.1. RMSD, RMSF, and RDF Analysis

**Figure 9:**
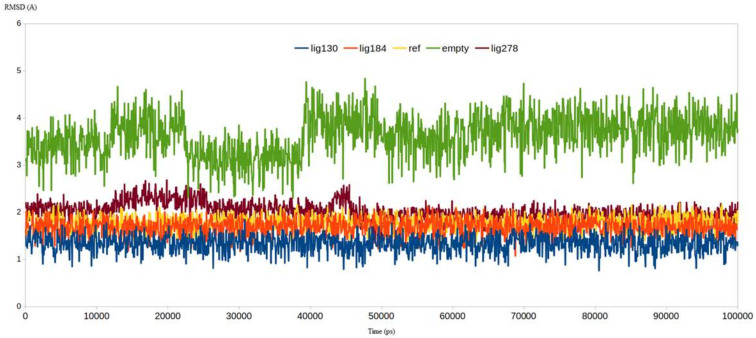
RMSD analysis for the MD simulations for the native enzyme (green), **PRD_002214** (yellow), **130** (blue), **184** (orange), and **278** (brown).

To endorse our virtual screening approach so far, five MD simulation experiments were conducted on the free M^pro^, co-crystallized ligand-M^pro^, **130**-M^pro^, **184**-M^pro^, and **278**-M^pro^. Interestingly, the calculated RMSD for the free M^pro^ exceeded 4.8 Å while the RMSD of the co-crystalized ligand-M^pro^ reached nearly 2.2 Å. As expected, the RMSD of **130**-M^pro^ and **184**-M^pro^ reached only 1.7 and 2.1 Å, respectively. In contrast, the **278**-M^pro^ complex had the highest RMSD value among the four ligands, reaching about 2.7 Å (Figure 9). The complex **130**-M^pro^ showed the least RMSD value which is a good indicator of its ability to further restrict the flexibility of M^pro^ compared to the co-crystalized ligand. Furthermore, its ability to stabilize the M^pro^ is attributed to the strong binding mode between the M^pro^ and compound **130**.

**Figure 10:**
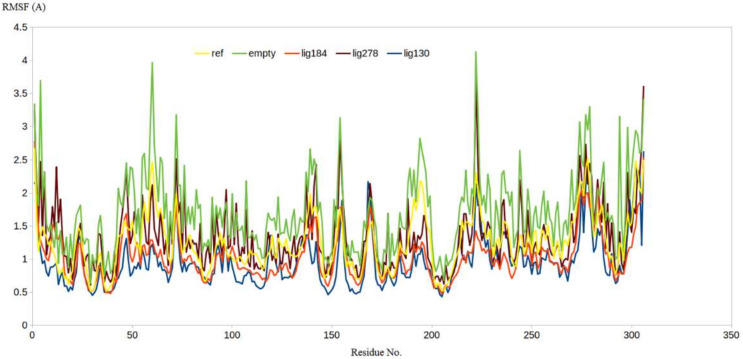
The RMSF analysis for the MD simulations for the native enzyme (orange), **PRD_002214** (blue), **130** (brown), **184** (yellow), and **278** (green).

Similar results were obtained when calculating the RMSF values for all the residues of the five systems where the native enzyme showed a significantly higher level of residues fluctuation during the simulation compared to the co-crystalized ligand and the three lead compounds (Figure 10). It is worth mentioning that the high flexibility of M^pro^ as shown by higher values for RMSD and RMSF is consistent with its intended function to process the resulting polyprotein from the replication cycle of the virus. Accordingly, the ability of compound **130** to produce lower values for RMSD and RMSF highlights its potentiality as a potent M^pro^ inhibitor.

**Figure 11:**
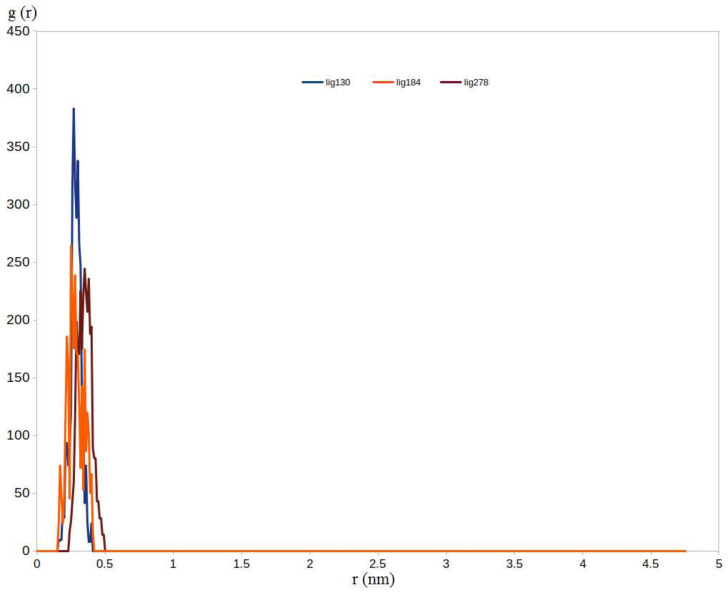
Radial distribution function (RDF) for the three ligands (**130**, **184,** and **278**) on the surface of the M^pro^ enzyme.

In addition to RMSD and RMSF calculations, the radial distribution function (RDF) was also computed to provide extra insights into the binding of the M^pro^ and the three selected compounds. RDF can explore the distance relationship (atom to atom) between two types of molecules (ligand, and receptor). The average density of ligand to protein (M^pro^) surface in the stable period was computed by analyzing the RDF of each ligand to M^pro^ surface [122]. The gmx rdf program was utilized to explore the RDF of the three ligands to the surface of the M^pro^, and the results are demonstrated in Figure 11. All of the RDF for the three ligands with the M^pro^ showed distinct peaks at 0.22~0.34 nm, a distance that enabled the three hits to form strong hydrogen bonds and hydrophobic interactions. Furthermore, among the three complexes, the **130**-M^pro^ complex reached the highest peak nearly at 0.25 nm, followed by **184**-M^pro^ and then **278**-M^pro^ which reached their maximum peaks at 0.23 nm and 0.34 nm, respectively. To this end, the RDF also proves the ability of the three compounds to predominantly distribute at a distance that allows strong interactions with the M^pro^ and also highlights the superiority of compound **130** over compounds **184** and **278**. In conclusion, the RDF calculations are highly matched with RMSD and RMSF calculations, endorsing the obtained results from the docking step.

#### 2.5.2. Binding Free Energy Calculations Using MM-PBSA Approach

The binding free energies between the four ligands (co-crystallized reference and the three retrieved lead compounds) with M^pro^ were computed from all the conformations in the saved trajectories utilizing the MM-PBSA approach. The g_mmpbsa package generated by Kumari et al. [123] was utilized to compute all the MM-PBSA binding free energy types (van der Waals, electrostatic, polar solvation, and SASA energies) for the four complexes of M^pro^ with the four ligands (Table 6). The calculated binding energy showed a significant higher binding affinity for **130** and **184** compared to the co-crystalized ligand and **278** which is consistent with the obtained results of both docking and MD simulations.

## 3. Method

### 3.1. Molecular Similarity Detection

Molecular Similarity detection was performed for the antiviral compounds using Discovery Studio software as described in the Supplementary Materials.

### 3.2. Molecular Fingerprint Detection

Molecular fingerprint detection was performed for the antiviral compounds using Discovery Studio software as described in the Supplementary Materials.

### 3.3. Docking Studies

Docking studies of the antiviral compounds were carried out for the antiviral compounds against SARS-CoV-2 main protease PDB ID: 6LU7 using MOE.14 software [124,125,126,127] as shown in the Supplementary Materials.

### 3.4. Toxicity Studies

In silico toxicity profiles were calculated for the antiviral compounds using Discovery Studio 4.0 [128,129,130] as shown in the Supplementary Materials.

### 3.5. MDS

All molecular dynamics (MD) simulations were performed for the antiviral compounds using the GROningen MAchine as shown in the Supplementary Materials.

## 4. Conclusions

Herein, it was concluded that luteoside C (**130**) was found to be the most potent inhibitor of M^pro^ among a collection of 310 natural antiviral compounds depending on a multi-phase in silico approach. The molecular structures similarity study against **PRD_002214**, the ligand of the target enzyme, favored thirty compounds. Then, the fingerprint study against **PRD_002214** elected the most similar sixteen compounds. The molecular docking against M^pro^ PDB ID: 6LU7 and toxicity studies favored eight compounds. The MD simulations experiments were carried out and revealed the superiority of **130** as the most potent inhibitor of M^pro^. Although the in vitro and in vivo examinations against COVID-19 are not accessible for our team, we depended on extensive well-structured in silico studies to offer all scientists who have the facilities the chance of strongly potential SARS-CoV-2 inhibitors. Our research is an important initial step that could be very helpful in the journey of finding a cure.

## Figures and Tables

**Table 1:** The calculated molecular properties of compounds having structural similarity with **PRD_002214**.

Comp.	ALog *p*	M. Wt	HBA	HBD	Rotatable Bonds	Rings	Aromatic Rings	MFPSA	Minimum Distance
**3**	2.91	313.348	4	3	6	2	2	0.239	1.802
**7**	0.857	546.629	9	3	11	5	1	0.223	1.053
**17**	3.911	444.522	4	2	11	3	3	0.183	1.382
**49**	3.714	481.647	5	1	7	5	2	0.11	1.682
**50**	3.387	465.604	5	2	6	5	2	0.132	1.689
**85**	0.436	446.404	10	5	5	4	2	0.373	1.801
**94**	3.743	372.412	6	1	7	3	2	0.192	1.804
**95**	2.879	388.411	7	2	7	3	2	0.235	1.671
**96**	3.513	444.431	9	0	10	4	2	0.225	1.515
**97**	3.474	442.415	9	0	9	4	2	0.226	1.586
**128**	−0.546	798.738	20	10	16	5	2	0.407	0.703
**129**	−0.925	756.702	19	11	14	5	2	0.425	0.795
**130**	−0.699	770.728	19	10	15	5	2	0.396	0.704
**131**	0.484	624.587	15	9	11	4	2	0.414	0.726
**132**	0.484	624.587	15	9	11	4	2	0.413	0.725
**156**	2.489	464.635	6	5	5	4	0	0.236	0.888
**157**	1.195	480.634	7	6	5	4	0	0.27	0.884
**158**	1.137	480.634	7	6	5	4	0	0.266	0.886
**180**	3.176	944.185	12	5	13	3	1	0.232	0.674
**184**	4.986	836.071	8	6	11	4	2	0.23	0.623
**203**	−0.499	503.583	10	4	8	3	0	0.268	1.002
**204**	−0.091	517.61	10	3	9	3	0	0.237	0.999
**210**	3.874	512.638	6	1	13	3	2	0.188	0.902
**237**	3.511	557.073	9	2	8	4	0	0.235	1.025
**248**	3.607	605.642	7	3	7	4	0	0.156	1.038
**264**	5.732	470.598	6	2	10	2	1	0.172	0.723
**276**	3.062	557.03	10	1	19	1	0	0.24	0.717
**277**	1.804	754.797	14	5	6	4	2	0.295	0.604
**278**	2.745	811.868	15	6	6	4	1	0.288	0.692
**280**	−0.367	480.523	5	7	10	3	3	0.359	0.649
**PRD_002214**	2.453	680.791	8	5	18	3	2	0.273	-

**Table 2:** Natural sources and antiviral activities of the most similar antiviral compounds.

No.	Name and Type	Source	Antiviral Activity
**3**	Moupinamide, an alkaloid	*Mollinedia* sp. [59]	Showed in silico inhibition against M^pro^ 6Y84 and the spike protein 6LXT) [60]
**7**	Homoharringtonine, an alkaloid	*Cephalotaxus* genus [61]	Inhibited the replication of SARS-CoV-2 (in vitro) with an EC_50_ value of 2.55 μM [62]
**17**	Aurantiamide acetate, a dipeptide	*Pongamia glabra* flowers [63] and *Aspergillus* sp [64]	In vitro inhibited the replication of Influenza A virus in MDCK cells [65]
**49**	Emetine, an alkaloid	*Cephaelis ipecacuanha* roots [66]	Inhibited SARS-CoV-2 replication in vitro with an EC_50_ of 0.46 μM [62] Inhibited SARS-CoV-2 protein synthesis and interaction of viral mRNA [67]
**50**	Psychotrine, an alkaloid	*Cephaelis acuminata* [68]	Inhibited COVID-19 M^pro^ in silico (ΔG = −3.5 kcal. mol^−1^) [69]
**85**	5-O-Methylgenistein-7-glucoside, an isoflavonoid	*Ulex europaeus* [70]	Inhibited herpes simplex virus (HSV) in vitro [70]
**94**	Arctigenin, a lignan	*Arctium lappa* [71]	In vivo inhibited influenza virus through interferon production [72]. Inhibited Spring viraemia of carp virus (SVCV) through inhibition of autophagy [73]
**95**	Trachelogenin, a lignan	*Ipomoea cairica* [74]	Inhibited the entry of hepatitis C virus through CD81 [75]
**96**	Rhinacanthin-F, a lignan	*Rhinacanthus nasutus* [76]	Inhibited of influenza virus type A [76]
**97**	Rhinacanthin-E, a lignan		
**128**	Luteosides A, B and C phenylpropanoid glycosides	*Markhamia lutea* [77]	Showed an in vitro inhibition of respiratory syncytial virus [77]
**129**			
**130**			
**131**	Verbascoside, a phenylpropanoid	*Verbascum olympicum* [78] and *Markhamia lutea* [77]	Inhibited in vitro herpes HSV-1, HSV-2 [79] and a respiratory syncytial virus [77]
**132**	Isoverbascoside, a phenylpropanoid		In vitro inhibited the respiratory syncytial virus [77]
**156**	Ponasterone A, a triterpenoidal saponins	*Podocarpus macrophyllus* [80] *Acrostichum aureum* [81]	Inhibited HIV-1 gene expression in mammalian cells [82]
**157**	Pterosterone, a triterpenoidal saponins		Exhibited an inhibition against (HIV-1) infection as CCR5 inhibitors [83]
**158**	Ecdysterone, a riterpenoidal saponins	*Diploclisia glaucescens* [84]	Inhibited HIV-1 in vitro [70]
**180**	Didemnin A, a peptide (depsipeptide)	Caribbean tunicate *Trididemnum solidum* [85]	Inhibited Coxsackie virus and equine rhinovirus in vitro [85] Inhibited both RNA and DNA viruses and HSV-2 in vitro [86]
**184**	Kahalalide E, a peptide	Marine Mollusk *Elysia rufescens* [87]	Inhibited HSV-2 in vitro [88]
**203**	Mycalamide A, an alkaloid	of the genus Mycale [89]	Inhibited SARS-CoV-1 in vitro with an IC_50_ of 0.2 µg kg^−1^ [90] and at a concentration of 5 ng/disc it stopped HSV-1 and Polio type I viruses [91]
**204**	Mycalamide B, an alkaloid		At a concentration of 2 ng/disc, it stopped HSV-1 and Polio-1 viruses [91]
**210**	Hennoxazole A, an alkaloid	A sponge *Polyfibrospongia* sp [92]	In vitro inhibited HSV-1 (IC_50_ of 0.6 lg/mL) [92]
**237**	Solenolide A, a diterpene	Marine Octocoral of the Genus *Solenopodium* [93]	In silico inhibition of M^pro^ PDB Id: 6LU7 with a binding free energy of −10.8 kcal. mol^−1^ [94]
**248**	Thyrsiferol, a triterpene	The red algae *Laurencia thyrsifera* [95]	In vitro inhibited VSV and HSV-1 at levels of 0.l-0μg/well and slight activity against A59 coronavirus [96]
**264**	Usneoidol Z a meroterpene	Brown Seaweed *Cystoseira usneoides* [97,98]	In vitro inhibited HSV-l/CV-I at concentrations of 20 and 10 μg/disk, respectively [98]
**276**	Punaglandin-1, an eicosanoid	The octocoral *Telesto riisei* [99]	Inhibited HSV in vitro [70]
**277**	Rifamycin B, a macrolide	The bacterium *Amycolatopsis rifamycinica* [100]	Inhibited (in vitro) murine sarcoma virus through cell transformation inhibition [101]
**278**	Streptovaricin B, an ansamycin	*Streptomyces spectabilis*, an actinomycete [102]	Stopped poxviruses replication through the inhibition of mRNA synthesis in early stages [101]
**280**	Distamycin A, an oligopeptide	*Streptomyces netropsis* [103]	Inhibited transcription and replication of different viruses [104] and inhibited the post-replicative mRNA synthesis of vaccinia virus [105]

**Table 3:** Degree of fingerprint similarity between the antiviral compounds and **PRD_002214**.

Comp.	Similarity	SA	SB	SC
**PRD_002214**	1	1116	0	0
**7**	0.683	772	15	344
**128**	0.648	926	313	190
**130**	0.651	889	249	227
**156**	0.652	818	139	298
**157**	0.654	824	143	292
**158**	0.644	819	156	297
**180**	0.718	1509	987	−393
**184**	0.800	1372	599	−256
**203**	0.654	755	39	361
**204**	0.644	780	95	336
**210**	0.666	748	7	368
**237**	0.676	868	168	248
**264**	0.681	725	−52	391
**276**	0.665	859	176	257
**277**	0.758	1026	237	90
**278**	0.724	1207	550	−91

SA: The bits number that was computed in the antiviral compounds and **PRD_002214**. SB: The bits number that was computed in the antiviral compounds but not **PRD_002214**. SC: The bits number that was computed in **PRD_002214** but not in the antiviral compounds.

**Table 4:** The computed values of ∆G of the antiviral compounds and the co-crystallized ligand against M^pro^.

Compound	∆G (kcal. mol^−1^)	Compound	∆G (kcal. mol^−1^)
**7**	−25.20	**204**	−33.03
**128**	−29.53	**210**	−28.41
**130**	−32.99	**237**	−23.75
**156**	−29.09	**264**	−25.85
**157**	−24.19	**276**	−21.66
**158**	−26.98	**277**	−24.08
**180**	−34.15	**278**	−29.00
**184**	−30.15	**PRD_002214**	−31.31
**203**	−31.20		

**Table 5:** Toxicity models of the antiviral compounds and the reference drug.

Comp.	128	130	156	180	184	203	204	278	Simeprevir
FDA rodent carcinogenicity	Non-Carcinogen								
Median carcinogenic potency (TD_50_), mg/kg/day	2.871	1.854	5.663	8.687	3.037	7.360	12.564	12.946	2.014
Rat maximum tolerated dose, g/kg body weight	2.382	1.277	0.137	0.002	0.021	0.018	0.029	0.020	0.003
Rat lethal dose (LD_50_) g/kg body weight	4.282	5.717	10.020	0.274	4.897	0.141	0.324	0.166	0.209
Rat chronic lowest observed adverse effect level (LOAEL), g/kg body weight	0.040	0.017	0.017	0.001	0.012	0.001	0.001	0.001	0.002
Ocular irritancy	Mild	Mild	Moderate	Moderate	None	Mild	Mild	Mild	Mild
Skin irritancy	Mild	Mild	Moderate	Mild	None	Mild	Mild	None	None

**Table 6:** Interaction energies and the binding free energy for the four complexes.

Complex	ΔE _Binding (kj/mol)_	ΔE _Electrostatic (kj/mol)_	ΔE *_Vander Waal_*_(kj/mol)_	ΔE _Polar Solvation (kj/mol)_	SASA _(kJ/mol)_
**130**	−286.9 ± 10.2	−139.1 ± 9.8	−245.7 ± 12.3	125.2 ± 8.1	−27.3 ± 0.9
**184**	−271.8 ± 8.9	−131.1 ± 9.1	−238.9 ± 8.5	124.9 ± 6.2	−26.7 ± 1.1
**278**	−236.6 ± 10.4	−111.8 ± 11.3	−205.6 ± 9.4	102.1 ± 10.7	−21.3 ± 0.8
**PRD_002214**	−252.5 ± 9.1	−119.5 ± 8.7	−226.7 ± 10.9	114.2 ± 7.4	−20.5 ± 1.2

## Data Availability

Data are avilabe with the corresponding author upon request.

## Supplementary Materials

The chemical structures of the tested compounds, detailed method in addition to the toxicity report can be downloaded at: https://www.mdpi.com/article/10.3390/ijms23136912/s1.
