# Supplementary material for: Multi-Step In Silico Discovery of Natural Drugs against COVID-19 Targeting Main Protease
Source: Int J Mol Sci. 2022 Jun 21;23(13):6912. doi: 10.3390/ijms23136912 (PMC9266348; doi:10.3390/ijms23136912)
Supplement: Supplementary file 1 [file ijms-23-06912-s001.zip › ijms-1725358-supplementary.pdf]

Supporting Data

Multi-Step *in Silico* Discovery of Natural Drugs against COVID-19 Targeting Main Protease

Eslam B. Elkaeed [1], Fadia S.Youssef [2], Ibrahim. H. Eissa [3], Hazem Elkady [3] Aisha A. Alsfouk[4], Mohamed L. Ashour [2,5], Mahmoud A. El Hassab[6], Sahar M. Abou-Seri [7]* and Ahmed M. Metwaly[8,9]*

[1] Department of Pharmaceutical Sciences, College of Pharmacy, AlMaarefa University, Riyadh 13713, Saudi Arabia
[2]Department of Pharmacognosy, Faculty of Pharmacy, Ain-Shams University, Abbasia, Cairo 11566, Egypt
[3] Pharmaceutical Medicinal Chemistry & Drug Design Department, Faculty of Pharmacy (Boys), Al-Azhar University, Cairo11884, Egypt
[4] Department of Pharmaceutical Sciences, College of Pharmacy, Princess Nourah bint Abdulrahman University, P.O. Box 84428, Riyadh 11671, Saudi Arabia
[5]Department of Pharmaceutical Sciences, Pharmacy Program, Batterjee Medical College, Jeddah 21442, Saudi Arabia
[6] Department of Pharmaceutical Chemistry, Faculty of Pharmacy, King Salman International University (KSIU), Ras Sudr City, South Sinai, Egypt
[7] Department of Pharmaceutical Chemistry, Faculty of Pharmacy, Cairo University, Kasr El-Aini Street, Cairo, P.O. Box 11562, Egypt.
[8] Pharmacognosy and Medicinal Plants Department, Faculty of Pharmacy (Boys), Al-Azhar University, Cairo 11884, Egypt
[9] Biopharmaceutical Product Research Department, Genetic Engineering and Biotechnology Research Institute, City of Scientific Research and Technological Applications, Alexandria, Egypt
*Correspondence: ametwaly@azhar.edu.eg, sahar.shaarawy@pharma.cu.edu.eg

Content

Figure S1. Chemical structures of the examined natural antiviral compounds<br>Figures S2-S6 (The binding modes of compounds **128**, **156, 180, 203, and 204**, respectively.	
Method	Molecular Similarity
	Fingerprint
	Docking studies
	Toxicity studies
	MDE studies
Toxicity report	

Chemical Structures

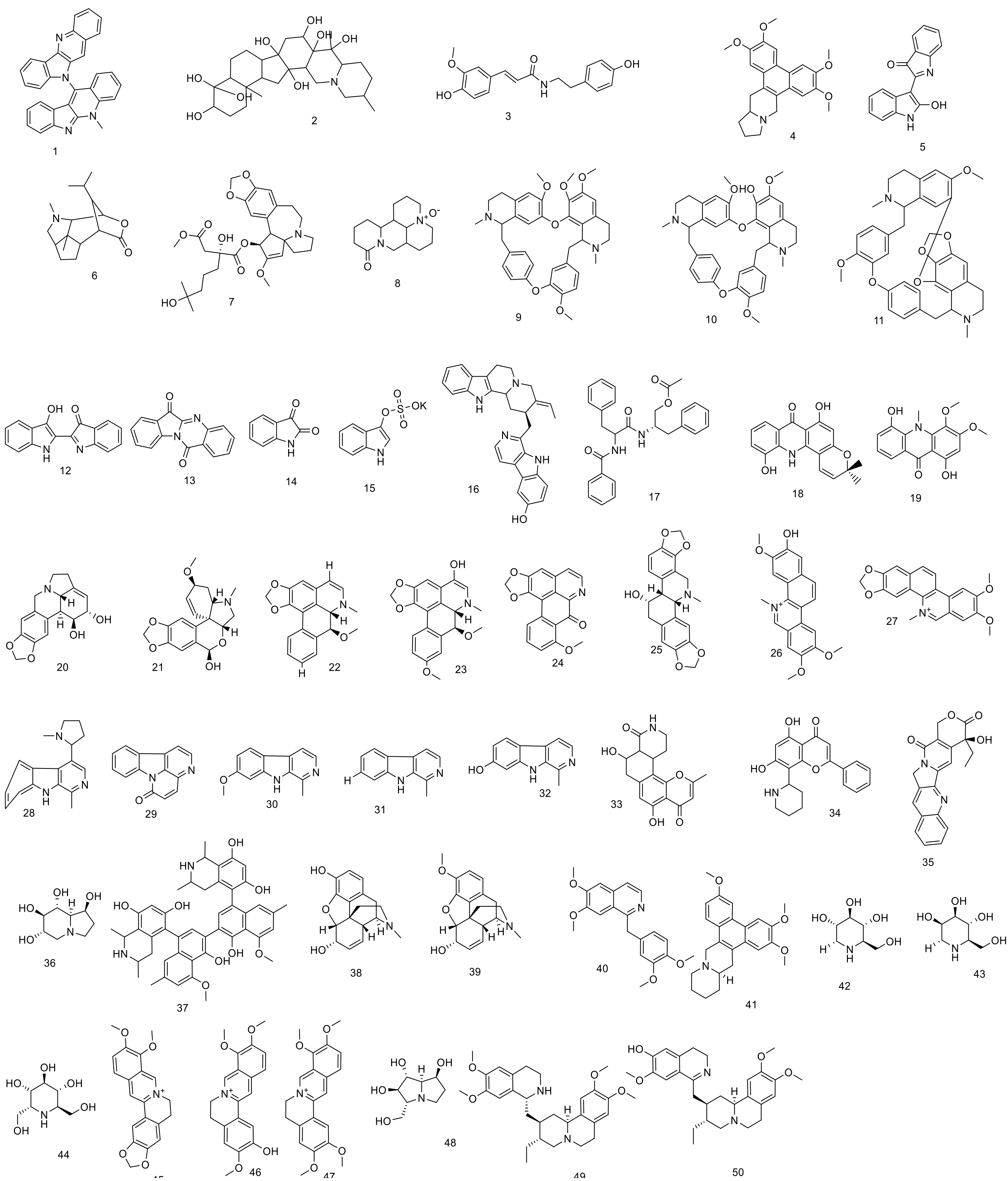

Figure S1. Chemical structures of the examined 310 natural antiviral compounds

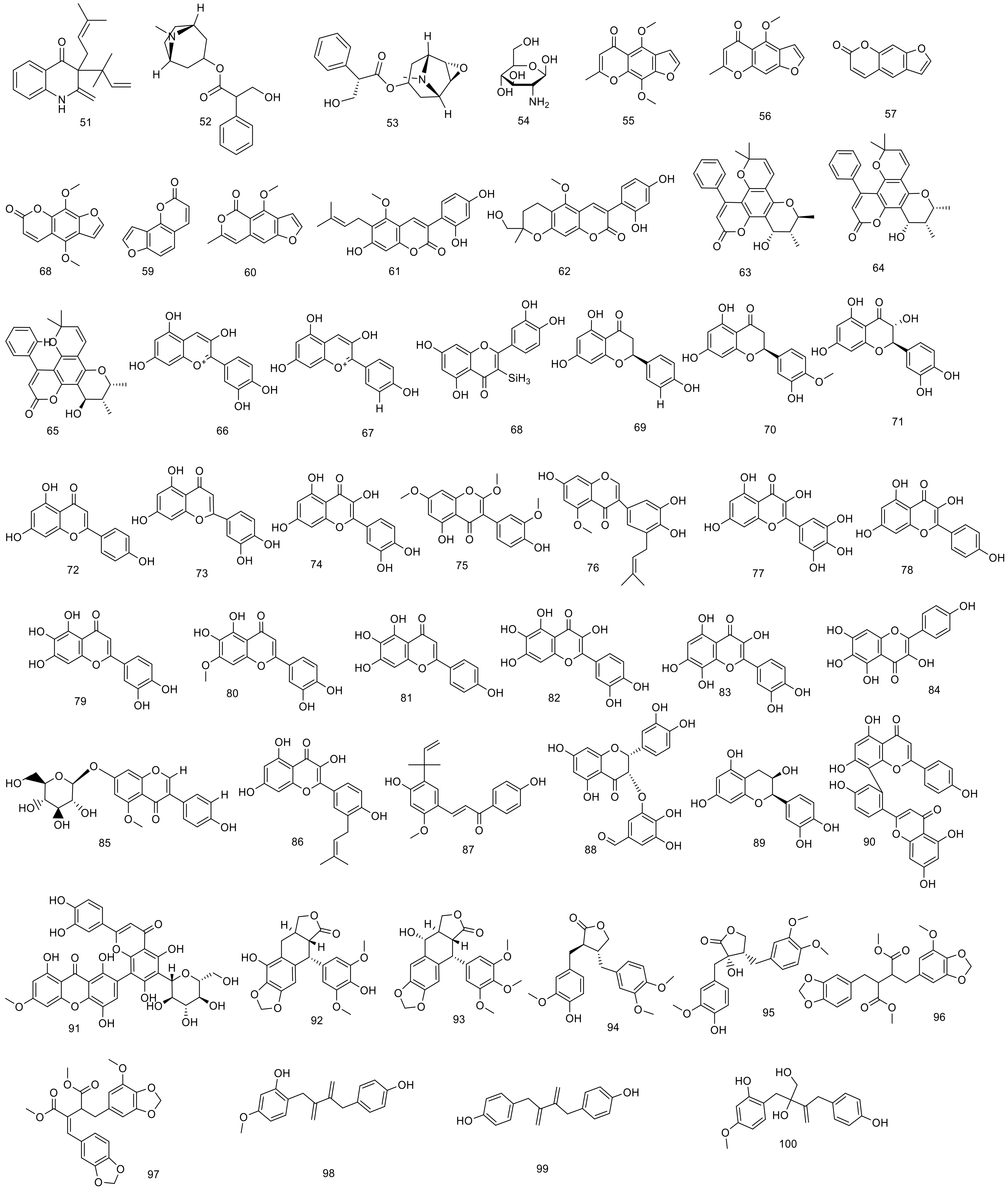

Figure S1. Chemical structures of the examined 310 natural antiviral compounds

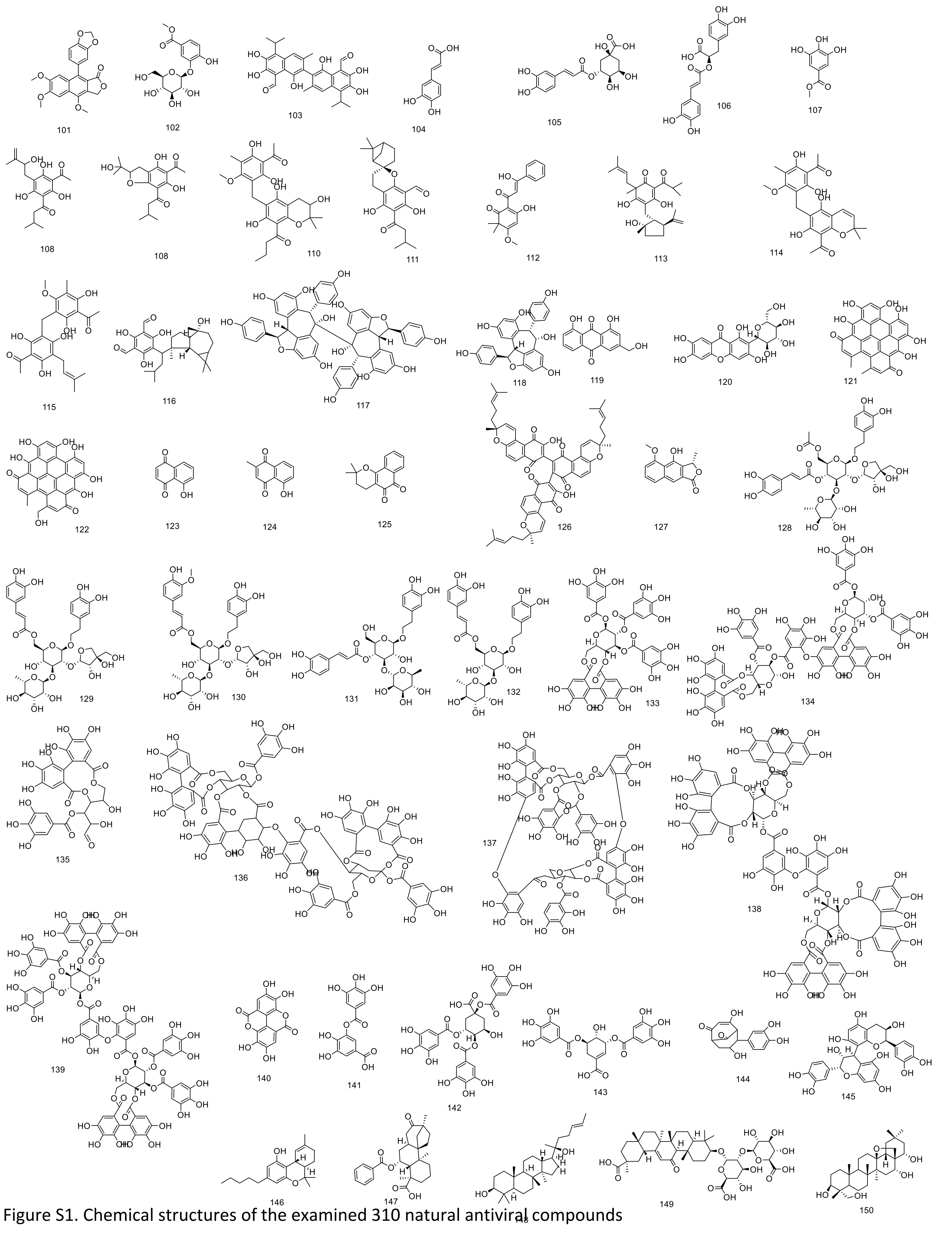

Figure S1. Chemical structures of the examined 310 natural antiviral compounds

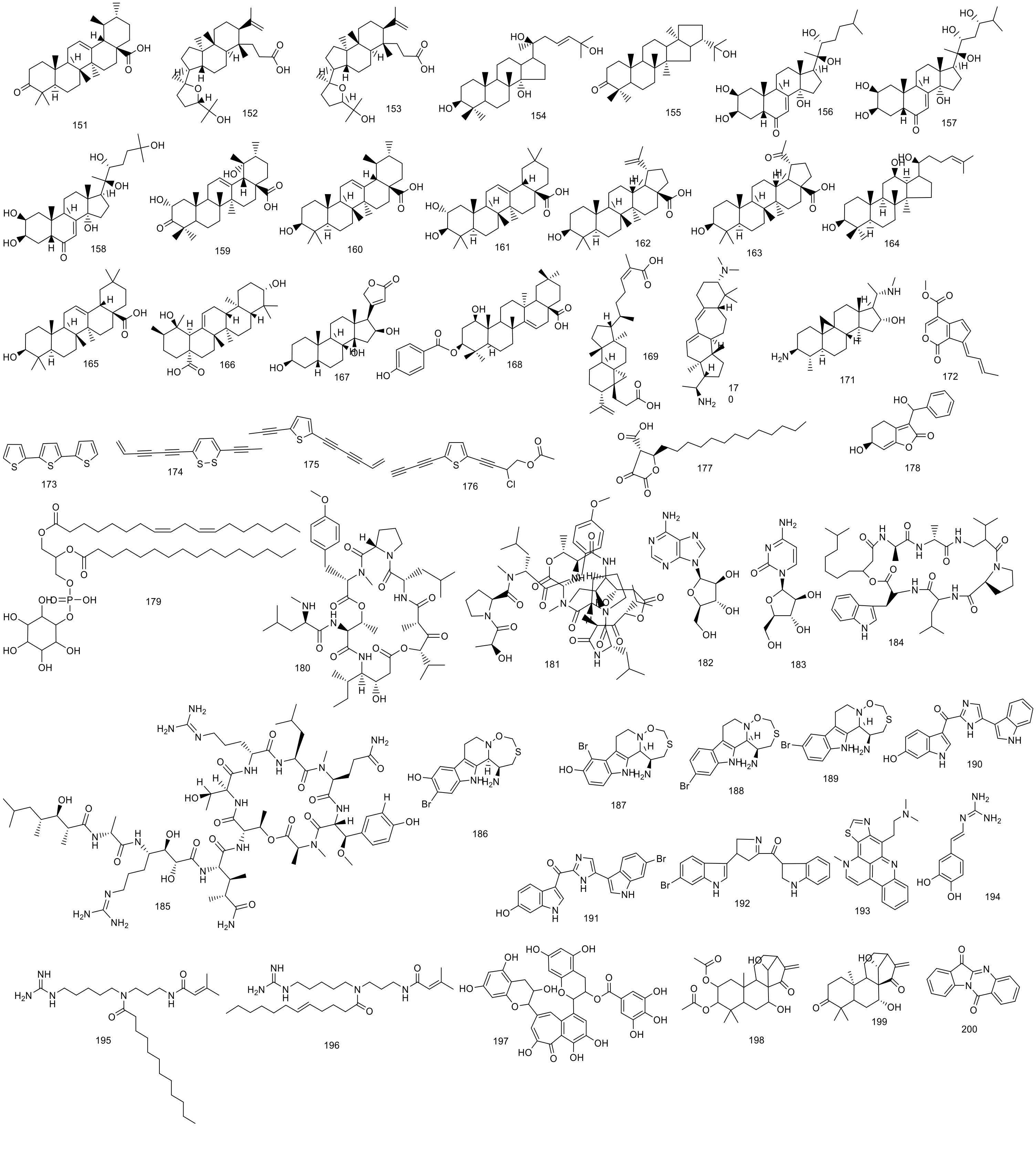

Figure S1. Chemical structures of the examined 310 natural antiviral compounds

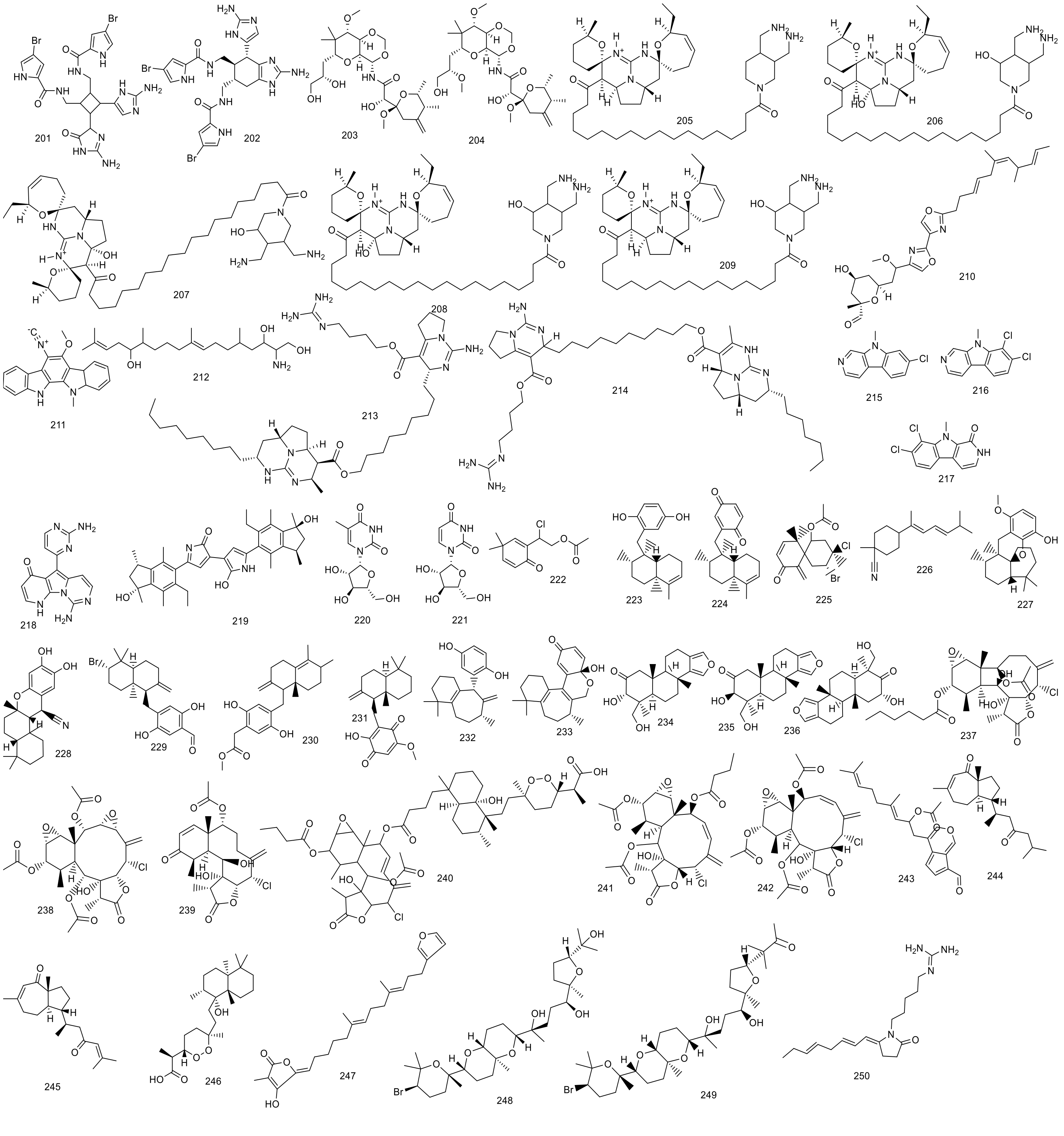

Figure S1. Chemical structures of the examined 310 natural antiviral compounds

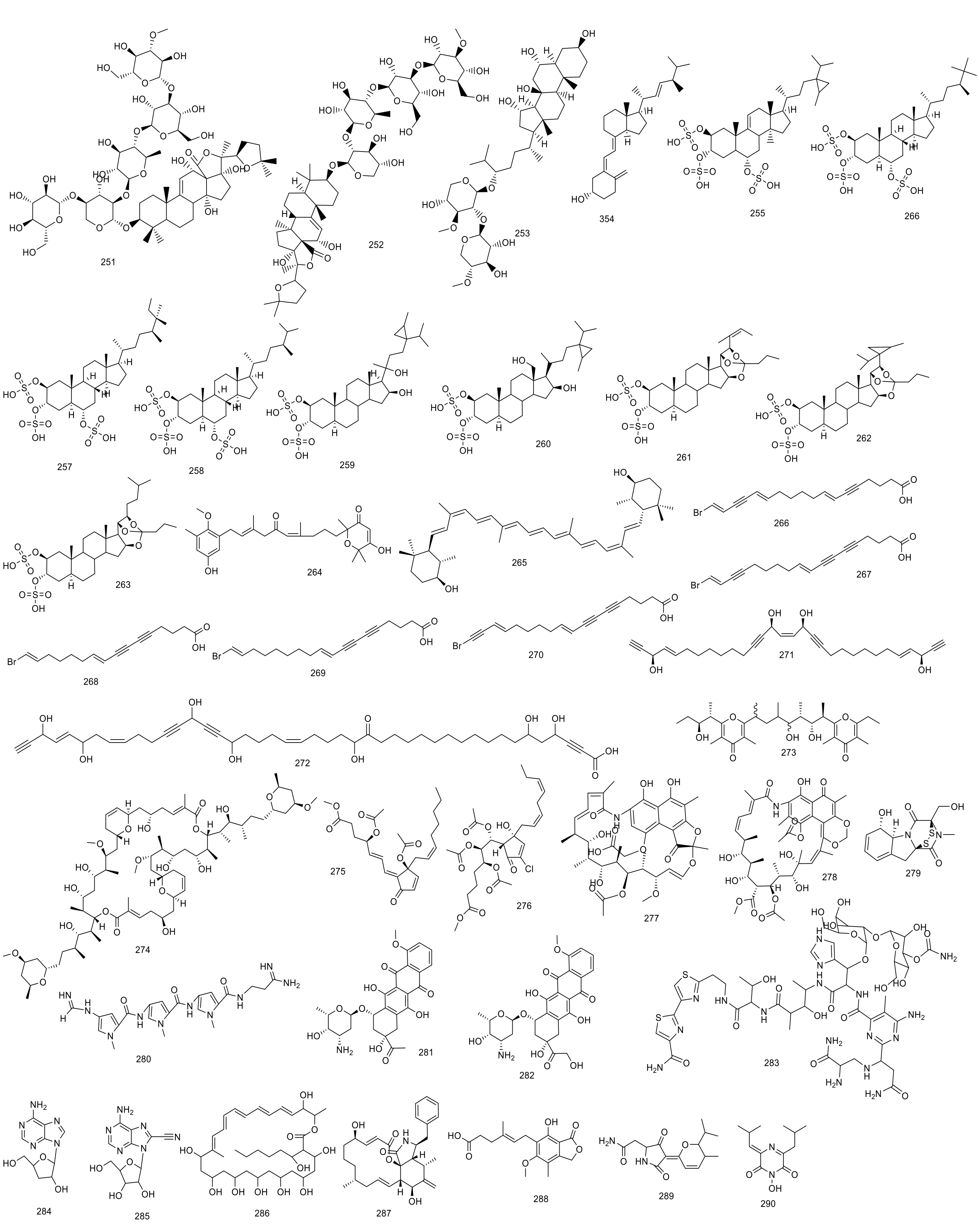

Figure S1. Chemical structures of the examined 310 natural antiviral compounds

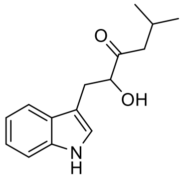
291

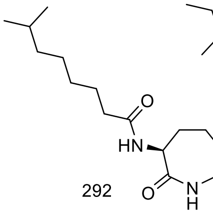
292

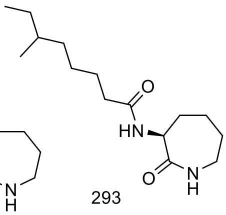
293

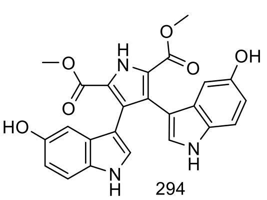
294

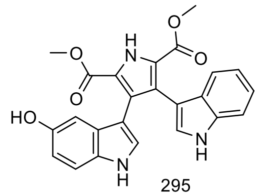
295

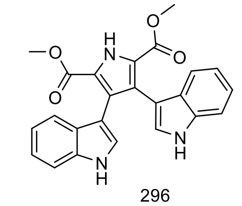
296

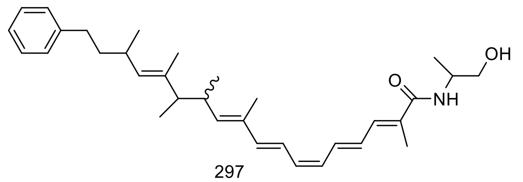
297

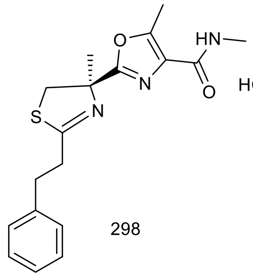
298

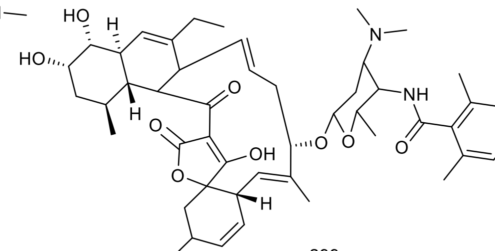
299

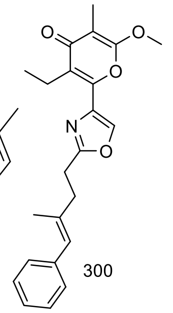
300

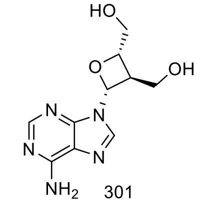
301

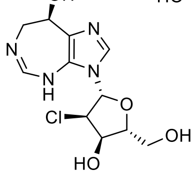
302

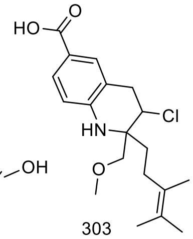
303

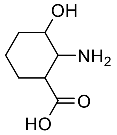
304

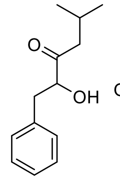
305

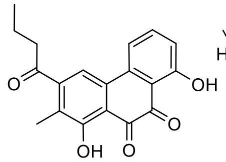
306

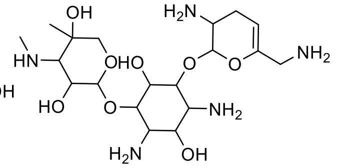
307

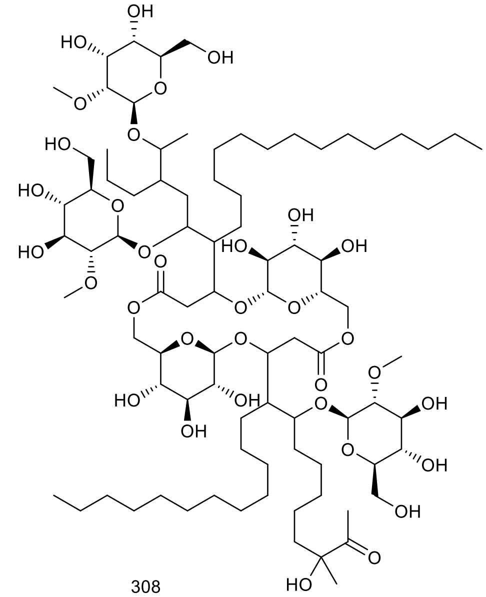
308

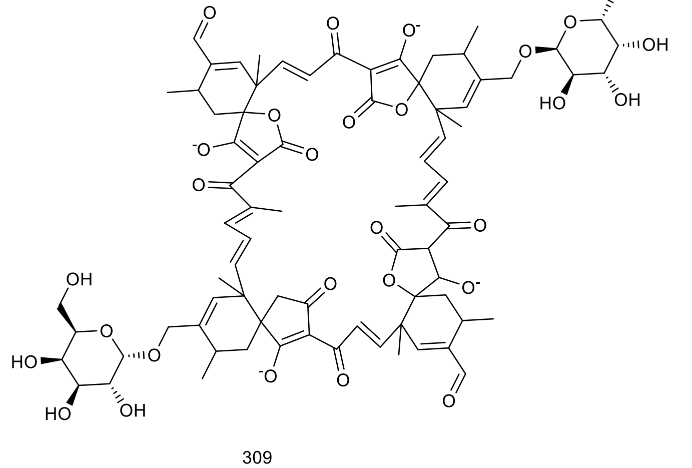
309

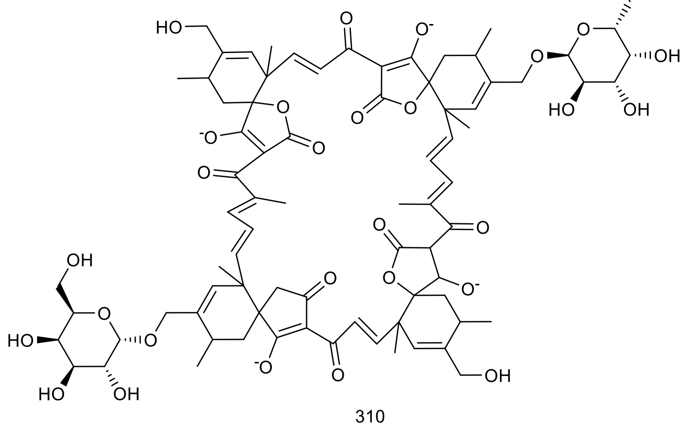
310

Figure S1. Chemical structures of the examined 310 natural antiviral compounds

The binding mode of compound 128

The antiviral compound (**128)** revealed five hydrogen bonds in addition to three hydrophobic interactions. The tetrahydro-2*H*-pyran-3,4,5-triol moiety was suited in the 1st pocket of the receptor making two hydrogen bonds with Gln189 and Asp187. Additionally, it was incorporated in three hydrophobic interactions with Met165, His164, and His41. The 3-(hydroxymethyl)tetrahydrofuran-3,4-diol moiety occupied the 2nd pocket and engaged in two hydrogen bonds with Thr190 and Glu166. Moreover, the 3-(3,4-dihydroxyphenyl)-1-(l-oxidanyl)prop-2-en-1-one formed a hydrophobic interaction with His41 in the 3rd pocket. Finally, the 4-ethylbenzene-1,2-diol moiety occupied the fourth pocket.

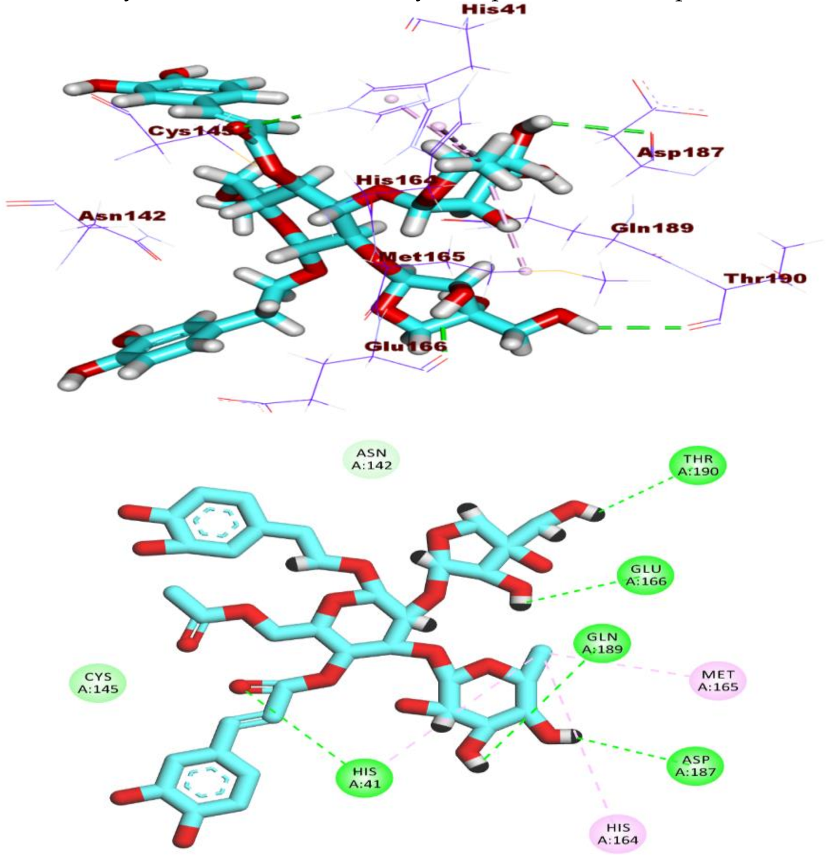

Figure S2. The binding pattern of compound **128** inside M pro (PDB ID: 6LU7) active site

The binding mode of compound 156

The antiviral compound, **156** revealed an affinity value of -29.09 kcal. Mol^{-1}. The docking pose showed that it occupied the four different pockets of the receptor through five hydrogen bonds with Phe140, Cys145, Leu141, Thr190, and Glu166. Additionally, it was engaged in two hydrophobic interactions with Met165 and Ala191.

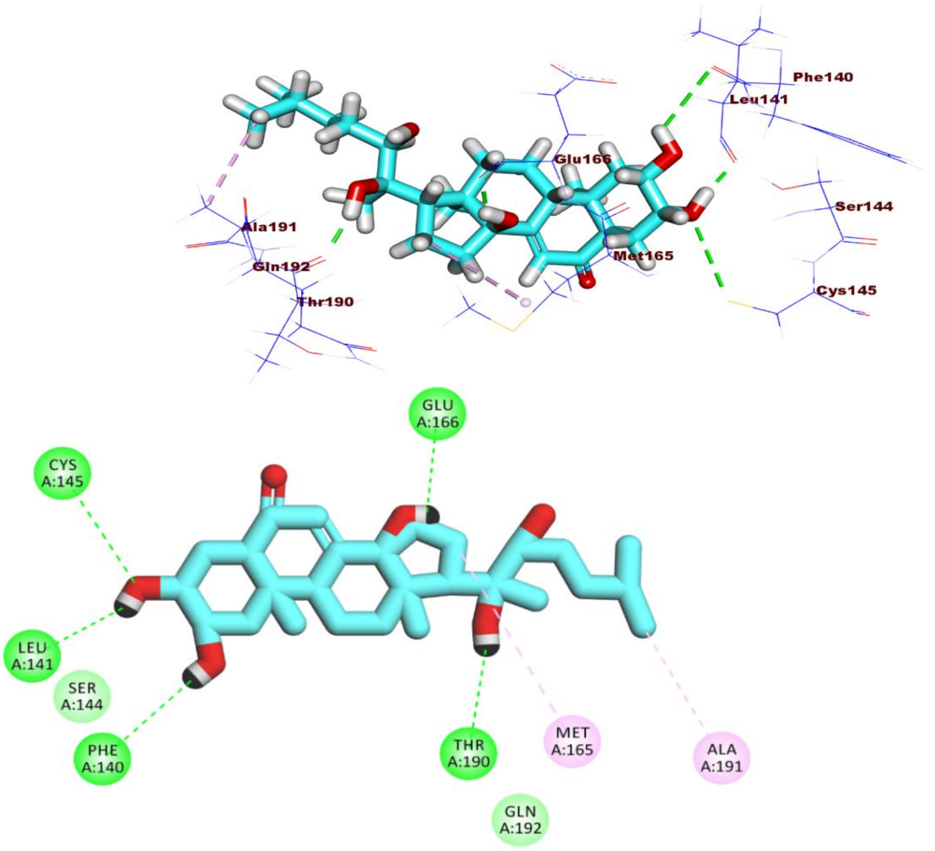

Figure S3. The binding pattern of compound **156** inside M^{pro} (PDB ID: 6LU7) active site

The binding mode of compound 180

The antiviral compound, **180** occupied the four different pockets of the main protease through the engagement in four hydrogen bonds in addition to thirteen hydrophobic interactions. The 4th pocket of M^{pro} was occupied by *p*.methoxybenzene and *N*,4-dimethyl-2-(methylamino)pentanamide moieties through the formation of five hydrophobic interactions with Pro168 and Leu167. While the 3rd pocket was occupied by the 1-hydroxy-3-methylpentan-2-yl)acetamide moiety through the formation of a hydrogen bond with His164 and hydrophobic interactions (three) with His41 and Met49. Likewise, the 2nd pocket was occupied by 2,5-dimethyl-6-((4-methyl-1-oxopentan-2-yl)amino)- 4,6-dioxohexan-3-yl acetate moiety through the engagement in hydrogen bonds with Cys145, Asn142, and His41 together with four hydrophobic interactions with His172, His163, and Cys145.

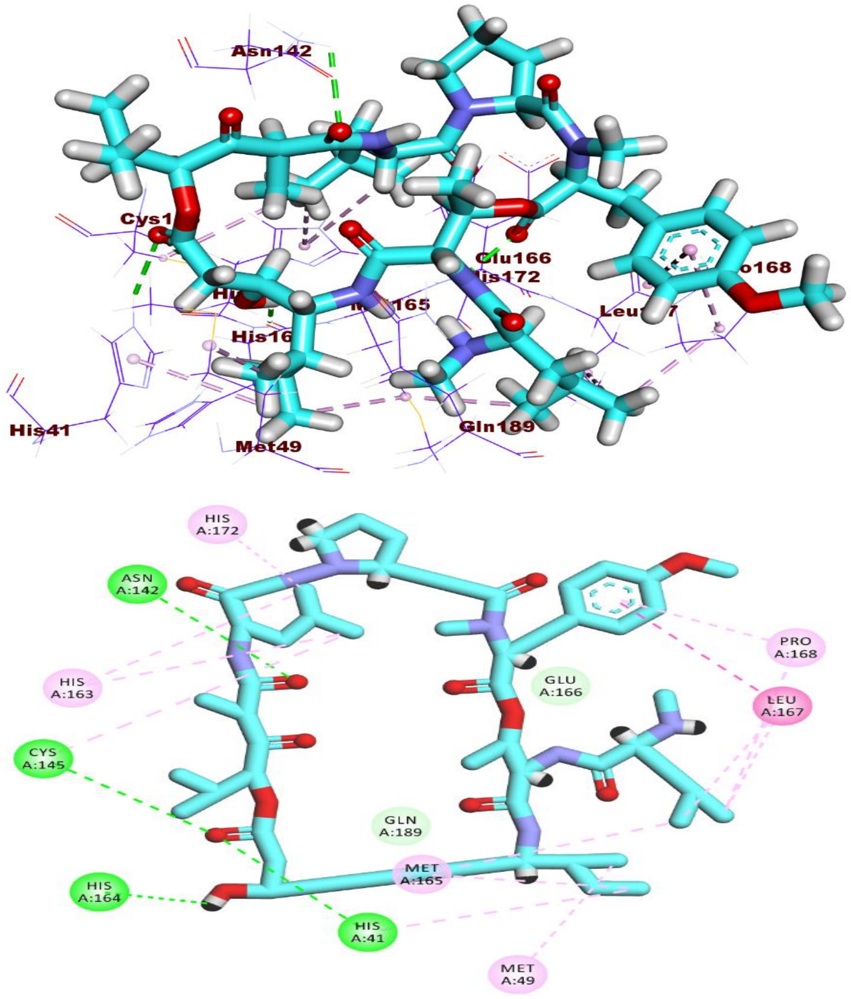

Figure S4. The binding pattern of compound **180** inside M ^{pro} (PDB ID: 6LU7) active site

The binding mode of compound 203

The proposed binding mode of **203** displayed occupation of the four pockets of the main protease. The 2,3-dimethyl-4-methylenetetrahydro-2*H*-pyran formed a hydrophobic interaction with Pro168 (in the 1st pocket). The butane-1,2-diol moiety was engaged in three hydrogen bonds with His163, Glu166, Phe140 (in the 2nd pocket). The 4-methoxy-3,3-dimethyltetrahydro-2*H*-pyran moiety formed a hydrophobic interaction with Cys145 (in the 3rd pocket). Meanwhile, the 2-hydroxy-*N*-methylacetamide moiety formed one hydrogen bond with Gln189 (in the 4th pocket).

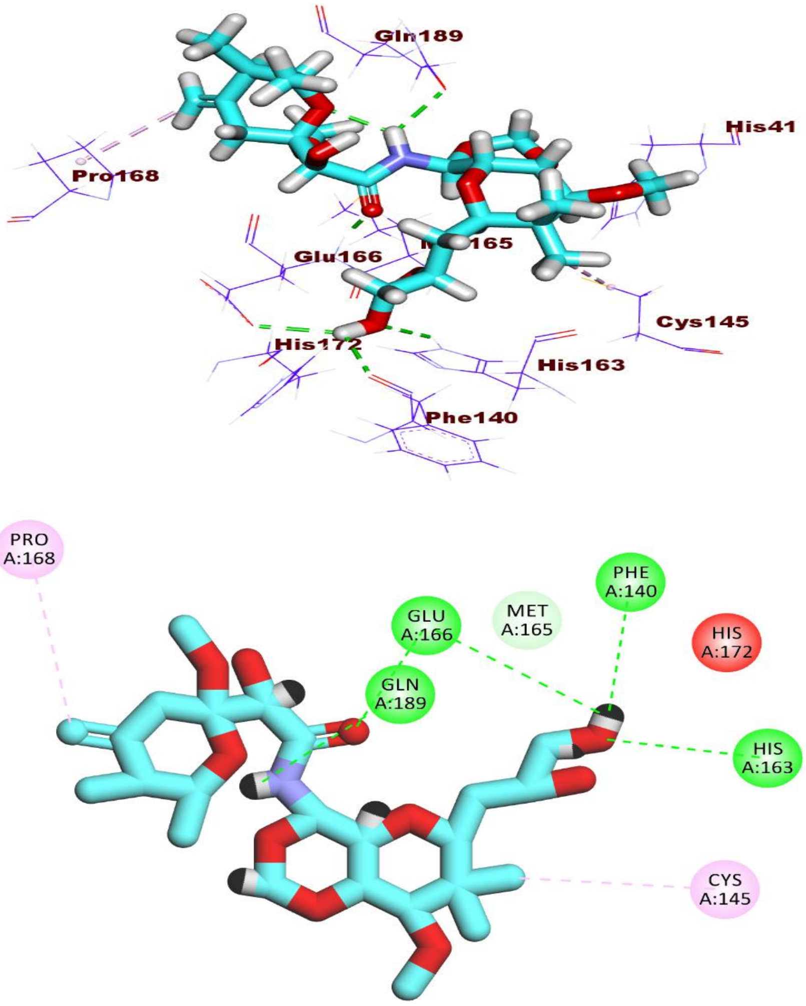

Figure S5. Binding mode of compound **203** inside M pro (PDB ID: 6LU7) active site

The binding mode of compound 204

Compound **204** showed a **ΔG** value of -33.03 kcal/mol and additionally buried in the main protease active pockets. The 6-methoxy-2,3-dimethyl-4-methylenetetrahydro-2*H*- pyran was engaged in a hydrogen bonding with Gly143 in addition to four hydrophobic interactions with Leu27, Cys145, His41. Also, 2-methoxybutan-1-ol made a hydrogen bond with Glu166. Finally, the 4-methoxy-3,3-dimethyltetrahydro-2*H*-pyran moiety was engaged in two hydrophobic interactions with His41 and Met165.

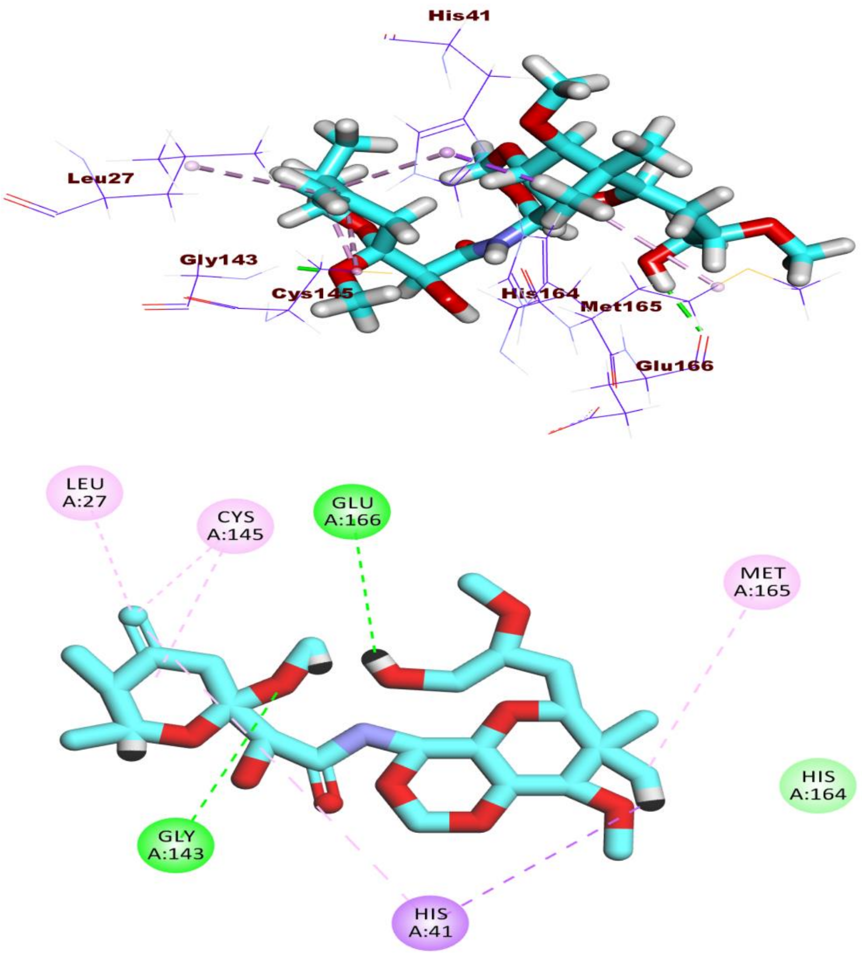

Figure S6. Binding mode of compound **204** inside M ^{pro} (PDB ID: 6LU7) active site

Method

Method

1- Molecular Similarity

Molecular Similarity of the 310 natural compounds against the co-crystallized ligand of SARS-CoV-2 main protease [(**PDB ID: 6LU7**) , resolution: 1.83Å] was carried out calculated using Discovery studio 4.0. At first, the CHARMM force field was applied then the compounds were prepared using prepare ligand protocol. Then, the 310 compounds were used as a test set while the co-crystallized ligand was used as a reference compounds. The protocol was adjusted to give 10% output. The default molecular properties were applied. The molecular properties include number of rotatable bonds, number of rings, number of aromatic rings, number of hydrogen bond donors (HBA), number of hydrogen bond acceptors (HBD), partition coefficient (ALog p), molecular weight (M. Wt), and molecular fractional polar surface area (MFPSA).

2- Fingerprint study

A fingerprint study of the 30 selected compounds against the co-crystallized ligand of SARS-Cov-2 main protease [(**PDB ID: 6LU7**) , resolution: 1.83Å] was carried out calculated using Discovery studio 4.0. At first, the CHARMM force field was applied then the compounds were prepared using prepare ligand protocol. Then, the 30 compounds were used as a test set while the co-crystallized ligand was used as a reference compound. The protocol was adjusted to give the most related compounds to the co-crystallized ligand (40-50%). The default molecular properties were applied. The used fingerprints were based on some parameters related to type of atoms which may be one of the following: charge, hybridization, H-bond acceptor, H-bond donor, Positive ionizable, Negative ionizable, Halogen, Aromatic, or None of the above. In addition, it includes the ALogP category of atoms.

3- Docking studies

Crystal structure of SARS-CoV-2 main protease [(**PDB ID: 6LU7**), resolution: 1.83Å] was obtained from Protein Data Bank. The docking investigation was accomplished using MOE2014 software. At first, the crystal structure of SARS-Cov-2 main protease was prepared by removing water molecules. Only one chain was retained besides the co-crystallized ligand. Then, the selected

chain was protonated and subjected to minimization of energy process. Next, the active site of the target protein was defined.

Structures of the tested compounds and the co-crystallized ligand were drawn using ChemBioDraw Ultra 14.0 and saved as MDL-SD format. Such file was opened using MOE to display the 3D structures which were protonated and subjected to energy minimization. Formerly, validation of the docking process was performed by docking the co-crystallized ligand against the isolated pocket of active site. The produced RMSD value indicated the validity of process. Finally, docking of the tested compounds was done through the dock option inserted in compute window. For each docked molecule, 30 docked poses were produced using ASE for scoring function and force field for refinement. The results of the docking process were then visualized using Discovery Studio 4.0 software.

4- Toxicity studies

The toxicity parameters of the tested compounds were calculated using Discovery studio 4.0. Indinavir was used as a reference drug. At first, the CHARMM force field was applied then the compounds were prepared and minimized according to the preparation of small molecule protocol. Then different parameters were calculated from the toxicity prediction (extensible) protocol.

5- Molecular Dynamics (MD)

All molecular dynamics (MD) simulations were performed using the GROningen MAchine for Chemical Simulations GROMACS 5.1.1 software developed by (University of Groningen, Royal Institute of Technology and Uppsala University) [1]. Five MD simulation experiments were conducted to validate the results retrieved from the docking step. One experiment was performed on the ligand-free Mpro, while the other four were conducted on the enzyme in complex with the retrieved **130, 184, 278** and the crystal reference. The ligand topologies were generated by the GlycoBioChem PRODRG2 Server under the GROMOS96 force field and were joined with the enzyme topology, using the standard published protocol. The typical workflow of the GROMACS enzyme–ligand simulations was applied with the solvation of the five systems, using the single point charge (SPC) water model to add water molecules to the cubic simulation boxes. The five systems were neutralized by adding the suitable number of counter-ions to the solvated complexes.

The energy minimization step for the unbound and the complexed enzyme structures was achieved, using the steepest descent minimization algorithm with a maximum of 50,000 steps and < 10.0 kJ/mol force under the GROMOS96 43a1 force field. Two consecutive equilibration ensembles were conducted on the energy-minimized structures.

Beginning with the NVT ensemble (constant number of particles, volume and temperature) (310 K) was done for 2 ns followed by the NPT ensemble (constant number of particles, pressure and temperature) for 8 ns. The particle mesh ewald (PME) method with a 12 Å cut-off and 12 Å Fourier spacing were used to get the long-range electrostatic value. The five equilibrated systems entered the production stage without any restraints for 100 ns with a time step of 2 fs, and the structural coordinates were saved every 20 ps. Both the temperature (310 K) and the pressure (1 atm) were regulated throughout the simulation V-rescale weak coupling method (modified Berendsen thermostat) and Parrinello−Rahman method. The root means square deviation (RMSD) and root means square fluctuation (RMSF) of the entire system and each residue respectively, were calculated from the generated trajectories from the production step. To provide further insights into the binding of the three ligands and the M^{pro} enzyme, the radial distribution function (RDF) was calculated

MM-PBSA Calculation and per-residue contribution

Binding-free energy calculations were performed, using MM–PBSA, which applies the following equation: $\Delta G_{(Binding)} = G_{(Complex)} - G_{(Receptor)} - G_{(Ligand)}$

where $G_{(Complex)}$ is the total free energy of the protein−ligand complex and $G_{(Receptor)}$ and $G_{(Ligand)}$ are the total free energies of the isolated protein and ligand in solvent, respectively. The total free energy of any of the three mentioned entities (complex, receptor and ligand) were calculated for all MD trajectories from its molecular mechanics potential energy plus the energy of the solvation, using the g_mmpbsa package implemented in the GROMACS software. Individual energies along with the values of standard deviations were calculated and then summed together to yield the average total free energy of each component. Finally, to calculate the binding-free energy, the total free energy of the receptor and the ligand were subtracted from the total free energy of the complex. These calculations were done for the four complexes of M^{pro}–130, M^{pro}-184, M^{pro}-278 and M^{pro}–crystal reference.

Toxicity Report

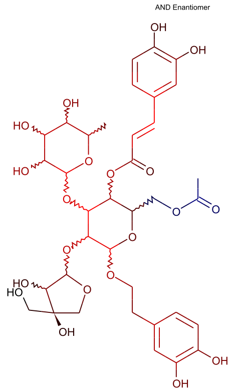

$C_{36}H_{46}O_{20}$
Molecular Weight: 798.73843
ALogP: -0.546
Rotatable Bonds: 16
Acceptors: 20
Donors: 10

Model Prediction

Prediction: Toxic
Probability: 0.973
Enrichment: 1.851
Bayesian Score: 15.947
Mahalanobis Distance: 15.893
Mahalanobis Distance p-value: 3.07e-013

Prediction: Positive if the Bayesian score is above the estimated best cutoff value from minimizing the false positive and false negative rate.
Probability: The esimated probability that the sample is in the positive category. This assumes that the Bayesian score follows a normal distribution and is different from the prediction using a cutoff.
Enrichment: An estimate of enrichment, that is, the increased likelihood (versus random) of this sample being in the category.
Bayesian Score: The standard Laplacian-modified Bayesian score.
Mahalanobis Distance: The Mahalanobis distance (MD) is the distance to the center of the training data. The larger the MD, the less trustworthy the prediction.
Mahalanobis Distance p-value: The p-value gives the fraction of training data with an MD greater than or equal to the one for the given sample, assuming normally distributed data. The smaller the p-value, the less trustworthy the prediciton. For highly non-normal X properties (e.g., fingerprints), the MD p-value is wildly inaccurate.

Structural Similar Compounds

Name	Solanine	alpha-Chaconine	Formoterol Fumarate
Structure	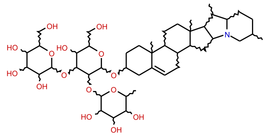	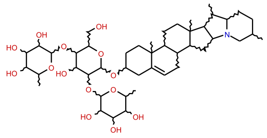	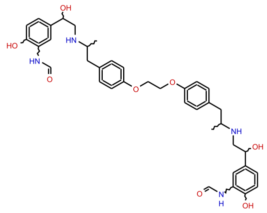
Actual Endpoint	Toxic	Toxic	Toxic
Predicted Endpoint	Toxic	Toxic	Toxic
Distance	1.009	1.149	1.297
Reference	Toxicol Appl Pharmacol 36(2):227-37; 1976	Toxicol Appl Pharmacol 36(2):227-37; 1976	Oyo Yakuri 27(2):239-249; 1984

Model Applicability

Unknown features are fingerprint features in the query molecule, but not found in the training set.

1. Num_H_Donors out of range. Value: 10. Training min, max, mean, SD: 0, 9, 1.4481, 1.588.
2. Num_H_Acceptors out of range. Value: 20. Training min, max, mean, SD: 0, 16, 3.9037, 3.081.
3. OPS PC1 out of range. Value: 8.8808. Training min, max, SD, explained variance: -5.294, 7.595, 2.775, 0.1128.
4. OPS PC19 out of range. Value: -3.221. Training min, max, SD, explained variance: -2.7817, 3.2747, 1.037, 0.0157.

Feature Contribution

Top features for positive contribution

Fingerprint	Bit/Smiles	Feature Structure	Score	Toxic in training set
SCFP_6	-2031220028	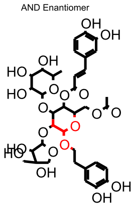 [*]OC(O[*])C([*])[*]	0.538	7 out of 7

SCFP_6	-37268149	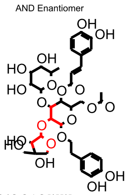 [*]C([*])C(OC1O[*][*]C1[*])C([*])[*]	0.523	6 out of 6
SCFP_6	-1642341584	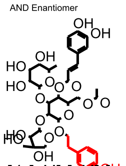 [*]CC[c]1:[cH]:[cH]:[c](O):[c]([*]):[cH]:1	0.504	5 out of 5

Top Features for negative contribution

Fingerprint	Bit/Smiles	Feature Structure	Score	Toxic in training set
SCFP_6	733985735	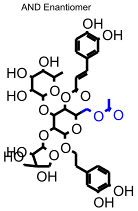 [*]COC(=O)C	-0.945	0 out of 3
SCFP_6	681123313	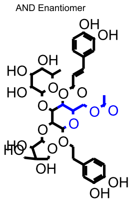 [*]OC(COC(=O)C)C([*])[*]	-0.422	0 out of 1
SCFP_6	-183093204	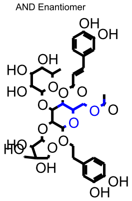 [*]OC(COC(=[*])[*])C([*])[*]	-0.422	0 out of 1

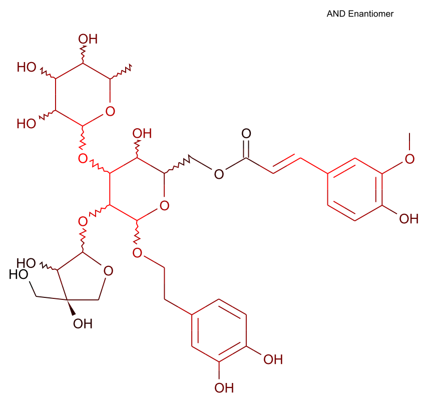

$C_{35}H_{46}O_{19}$
Molecular Weight: 770.72833
ALogP: -0.699
Rotatable Bonds: 15
Acceptors: 19
Donors: 10

Model Prediction

Prediction: Toxic
Probability: 0.990
Enrichment: 1.882
Bayesian Score: 19.253
Mahalanobis Distance: 14.317
Mahalanobis Distance p-value: 9.51e-010

Prediction: Positive if the Bayesian score is above the estimated best cutoff value from minimizing the false positive and false negative rate.
Probability: The esimated probability that the sample is in the positive category. This assumes that the Bayesian score follows a normal distribution and is different from the prediction using a cutoff.
Enrichment: An estimate of enrichment, that is, the increased likelihood (versus random) of this sample being in the category.
Bayesian Score: The standard Laplacian-modified Bayesian score.
Mahalanobis Distance: The Mahalanobis distance (MD) is the distance to the center of the training data. The larger the MD, the less trustworthy the prediction.
Mahalanobis Distance p-value: The p-value gives the fraction of training data with an MD greater than or equal to the one for the given sample, assuming normally distributed data. The smaller the p-value, the less trustworthy the prediciton. For highly non-normal X properties (e.g., fingerprints), the MD p-value is wildly inaccurate.

Structural Similar Compounds

Name	Solanine	alpha-Chaconine	Formoterol Fumarate
Structure	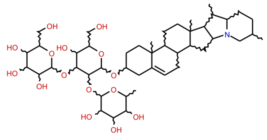	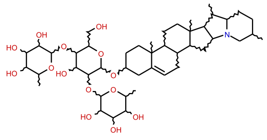	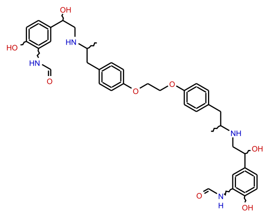
Actual Endpoint	Toxic	Toxic	Toxic
Predicted Endpoint	Toxic	Toxic	Toxic
Distance	0.936	1.085	1.255
Reference	Toxicol Appl Pharmacol 36(2):227-37; 1976	Toxicol Appl Pharmacol 36(2):227-37; 1976	Oyo Yakuri 27(2):239-249; 1984

Model Applicability

Unknown features are fingerprint features in the query molecule, but not found in the training set.

1. Num_H_Donors out of range. Value: 10. Training min, max, mean, SD: 0, 9, 1.4481, 1.588.
2. Num_H_Acceptors out of range. Value: 19. Training min, max, mean, SD: 0, 16, 3.9037, 3.081.
3. OPS PC1 out of range. Value: 8.6292. Training min, max, SD, explained variance: -5.294, 7.595, 2.775, 0.1128.
4. OPS PC4 out of range. Value: 5.4941. Training min, max, SD, explained variance: -4.3289, 5.2858, 1.931, 0.0547.

Feature Contribution

Top features for positive contribution				
**Fingerprint**	**Bit/Smiles**	**Feature Structure**	**Score**	**Toxic in training set**
SCFP_6	-2031220028	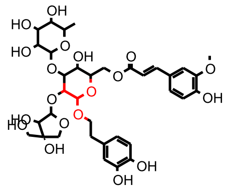 [*]OC(O[*])C([*])[*]	0.538	7 out of 7

SCFP_6	-37268149	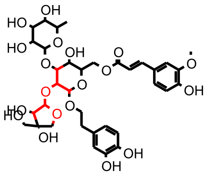 [*]C([*])C(OC1O[*][*] C1[*])C([*])[*]	0.523	6 out of 6
SCFP_6	-1642341584	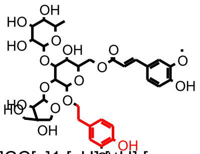 [*]CC[c]1:[cH]:[cH]:[c](O):[c]([*]):[cH]:1	0.504	5 out of 5
**Top Features for negative contribution**				
**Fingerprint**	**Bit/Smiles**	**Feature Structure**	**Score**	**Toxic in training set**
SCFP_6	1586247563	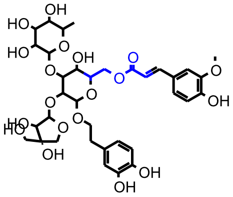 [*]C([*])COC(=O)C=[*]	-0.438	1 out of 4
SCFP_6	-183093204	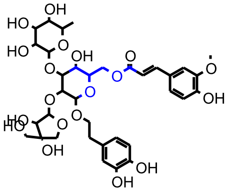 [*]OC(COC(=[*])[*])C([*])[*]	-0.422	0 out of 1
SCFP_6	1132907712	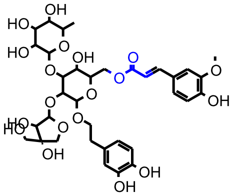 [*]OC(=O)C=[*]	-0.184	9 out of 21

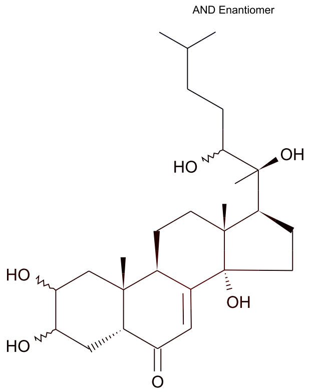

$C_{27}H_{44}O_6$
Molecular Weight: 464.63466
ALogP: 2.489
Rotatable Bonds: 5
Acceptors: 6
Donors: 5

Model Prediction

Prediction: Toxic
Probability: 0.684
Enrichment: 1.301
Bayesian Score: 3.514
Mahalanobis Distance: 8.742
Mahalanobis Distance p-value: 0.266

Prediction: Positive if the Bayesian score is above the estimated best cutoff value from minimizing the false positive and false negative rate.
Probability: The esimated probability that the sample is in the positive category. This assumes that the Bayesian score follows a normal distribution and is different from the prediction using a cutoff.
Enrichment: An estimate of enrichment, that is, the increased likelihood (versus random) of this sample being in the category.
Bayesian Score: The standard Laplacian-modified Bayesian score.
Mahalanobis Distance: The Mahalanobis distance (MD) is the distance to the center of the training data. The larger the MD, the less trustworthy the prediction.
Mahalanobis Distance p-value: The p-value gives the fraction of training data with an MD greater than or equal to the one for the given sample, assuming normally distributed data. The smaller the p-value, the less trustworthy the prediciton. For highly non-normal X properties (e.g., fingerprints), the MD p-value is wildly inaccurate.

Structural Similar Compounds

Name	Fenoterol	Diflorasone Diacetate	Ochratoxin a
Structure	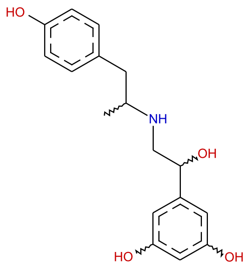	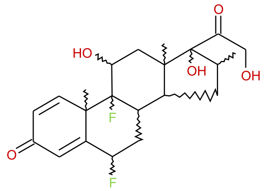	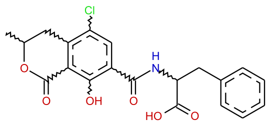
Actual Endpoint	Toxic	Toxic	Toxic
Predicted Endpoint	Toxic	Toxic	Toxic
Distance	0.719	0.759	0.759
Reference	Iyakuhin Kenkyu 12(3):742-761; 1981	Oyo Yakuri 28(2):207-224; 1984	Toxicol Appl Pharmacol 37(2):331-8; 1976

Model Applicability

Unknown features are fingerprint features in the query molecule, but not found in the training set.

1. All properties and OPS components are within expected ranges.

Feature Contribution

Top features for positive contribution

Fingerprint	Bit/Smiles	Feature Structure	Score	Toxic in training set
SCFP_6	-1971196727	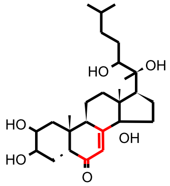 [*]\C=C\C(=[*])[*]	0.293	13 out of 18

SCFP_6	-424515134	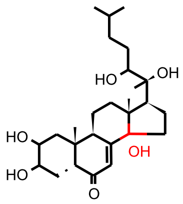[*]C([*])O	0.275	39 out of 56
SCFP_6	397733179	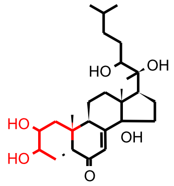[*]C1([*])[*]CC(O)C(O)C1	0.271	1 out of 1
**Top Features for negative contribution**				
**Fingerprint**	**Bit/Smiles**	**Feature Structure**	**Score**	**Toxic in training set**
SCFP_6	-1870530637	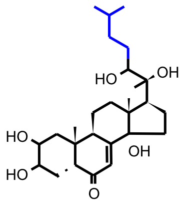[*]CCC(C)C	-0.422	0 out of 1
SCFP_6	-1043339860	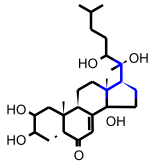[*]C([*])([*])[C@H]1C [*][*]C1([*])[*]	-0.114	13 out of 28
SCFP_6	13	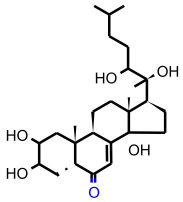[*]=O	0.000	81 out of 162

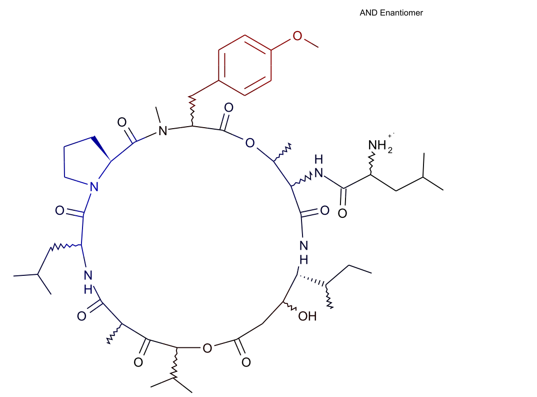

$C_{49}H_{79}N_6O_{12}$
Molecular Weight: 944.18455
ALogP: 3.176
Rotatable Bonds: 13
Acceptors: 12
Donors: 5

Model Prediction

Prediction: Non-Toxic
Probability: 0.430
Enrichment: 0.818
Bayesian Score: -3.396
Mahalanobis Distance: 13.003
Mahalanobis Distance p-value: 4.38e-007

Prediction: Positive if the Bayesian score is above the estimated best cutoff value from minimizing the false positive and false negative rate.
Probability: The esimated probability that the sample is in the positive category. This assumes that the Bayesian score follows a normal distribution and is different from the prediction using a cutoff.
Enrichment: An estimate of enrichment, that is, the increased likelihood (versus random) of this sample being in the category.
Bayesian Score: The standard Laplacian-modified Bayesian score.
Mahalanobis Distance: The Mahalanobis distance (MD) is the distance to the center of the training data. The larger the MD, the less trustworthy the prediction.
Mahalanobis Distance p-value: The p-value gives the fraction of training data with an MD greater than or equal to the one for the given sample, assuming normally distributed data. The smaller the p-value, the less trustworthy the prediciton. For highly non-normal X properties (e.g., fingerprints), the MD p-value is wildly inaccurate.

Structural Similar Compounds

Name	Aclacinomycin a	alpha-Chaconine	Formoterol Fumarate
Structure	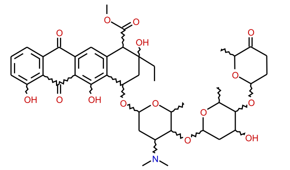	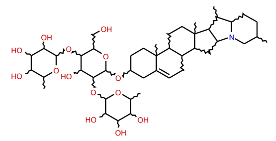	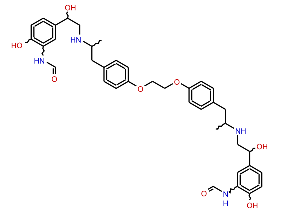
Actual Endpoint	Toxic	Toxic	Toxic
Predicted Endpoint	Toxic	Toxic	Toxic
Distance	0.838	1.062	1.069
Reference	Oyo Yakuri 19(5):783-790; 1980	Toxicol Appl Pharmacol 36(2):227-37; 1976	Oyo Yakuri 27(2):239-249; 1984

Model Applicability

Unknown features are fingerprint features in the query molecule, but not found in the training set.

1. OPS PC1 out of range. Value: 8.9727. Training min, max, SD, explained variance: -5.294, 7.595, 2.775, 0.1128.

Feature Contribution

Top features for positive contribution

Fingerprint	Bit/Smiles	Feature Structure	Score	Toxic in training set
SCFP_6	-1642341584	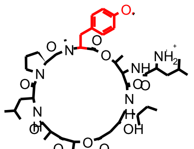 [*]CC[c]1:[cH]:[cH]:[c](O):[c]([*]):[cH]:1	0.504	5 out of 5

SCFP_6	-1849095515	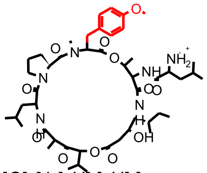 [*]C[c]1:[cH]:[cH]:[c](OC):[cH]:[cH]:1	0.478	4 out of 4
SCFP_6	1237755852	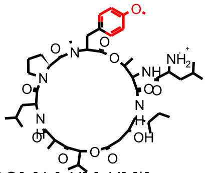 CO[c]1:[cH]:[cH]:[*]:[cH]:[cH]:1	0.453	8 out of 9
**Top Features for negative contribution**				
**Fingerprint**	**Bit/Smiles**	**Feature Structure**	**Score**	**Toxic in training set**
SCFP_6	-1026877279	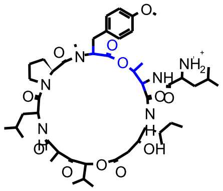 [*]C([*])C(C)OC(=O)C([*])[*]	-0.718	0 out of 2
SCFP_6	-1946889102	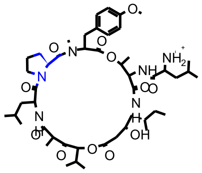 [*]N1[*][*]C[C@H]1C(=[*])[*]	-0.512	4 out of 14
SCFP_6	2002926168	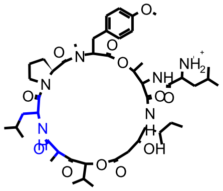 [*]CC(NC(=O)C([*])[*])C(=[*])[*]	-0.443	2 out of 7

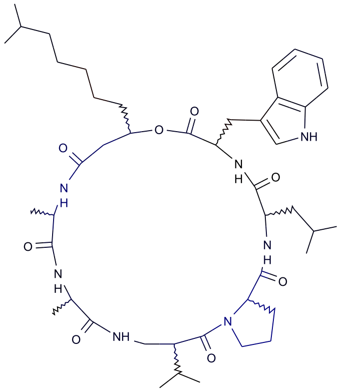

$C_{45}H_{69}N_7O_8$
Molecular Weight: 836.07145
ALogP: 4.986
Rotatable Bonds: 11
Acceptors: 8
Donors: 6

Model Prediction

Prediction: Non-Toxic
Probability: 0.333
Enrichment: 0.633
Bayesian Score: -7.103
Mahalanobis Distance: 13.670
Mahalanobis Distance p-value: 2.1e-008

Prediction: Positive if the Bayesian score is above the estimated best cutoff value from minimizing the false positive and false negative rate.
Probability: The esimated probability that the sample is in the positive category. This assumes that the Bayesian score follows a normal distribution and is different from the prediction using a cutoff.
Enrichment: An estimate of enrichment, that is, the increased likelihood (versus random) of this sample being in the category.
Bayesian Score: The standard Laplacian-modified Bayesian score.
Mahalanobis Distance: The Mahalanobis distance (MD) is the distance to the center of the training data. The larger the MD, the less trustworthy the prediction.
Mahalanobis Distance p-value: The p-value gives the fraction of training data with an MD greater than or equal to the one for the given sample, assuming normally distributed data. The smaller the p-value, the less trustworthy the prediciton. For highly non-normal X properties (e.g., fingerprints), the MD p-value is wildly inaccurate.

Structural Similar Compounds

Name	Bromocriptine	Formoterol Fumarate	Reserpate
Structure	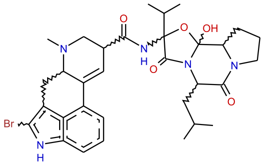	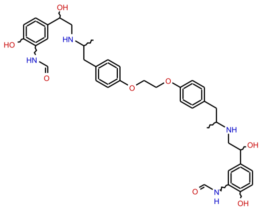	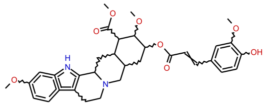
Actual Endpoint	Non-Toxic	Toxic	Toxic
Predicted Endpoint	Non-Toxic	Toxic	Toxic
Distance	1.002	1.058	1.062
Reference	Toxicol Lett 50:189-194; 1990	Oyo Yakuri 27(2):239-249; 1984	Oyo Yakuri 18:105-124; 1979

Model Applicability

Unknown features are fingerprint features in the query molecule, but not found in the training set.

1. All properties and OPS components are within expected ranges.

Feature Contribution

Top features for positive contribution

Fingerprint	Bit/Smiles	Feature Structure	Score	Toxic in training set
SCFP_6	1513450550	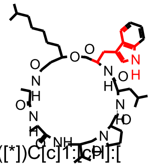 [*]C([*])C[c]1:[cH]:[nH]:[c](:[*]):[c]:1:[cH]:[*]	0.271	1 out of 1

SCFP_6	1966183260	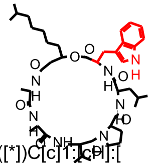 [*]C([*])C[c]1:[cH]:[nH]:[c]2:[cH]:[cH]:[cH]:[cH]:[c]:1:2	0.271	1 out of 1
SCFP_6	-1529004787	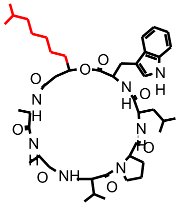 [*]CCCCCC(C)C	0.271	1 out of 1

Top Features for negative contribution

Fingerprint	Bit/Smiles	Feature Structure	Score	Toxic in training set
SCFP_6	-1047009138	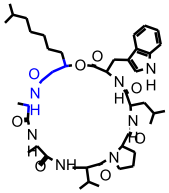 [*]C([*])CC(=O)NC([*])[*]	-0.718	0 out of 2
SCFP_6	-1026877279	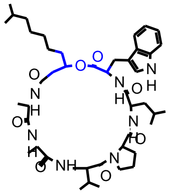 [*]C([*])C(C)OC(=O)C([*])[*]	-0.718	0 out of 2
SCFP_6	2005026407	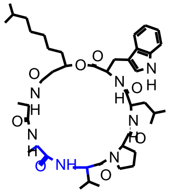 [*]C([*])CNC(=O)C([*])[*]	-0.718	0 out of 2

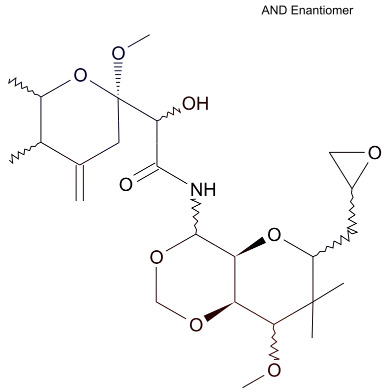

$C_{24}H_{39}NO_9$
Molecular Weight: 485.56776
ALogP: 0.267
Rotatable Bonds: 7
Acceptors: 9
Donors: 2

Model Prediction

Prediction: Toxic
Probability: 0.607
Enrichment: 1.154
Bayesian Score: 1.540
Mahalanobis Distance: 9.818
Mahalanobis Distance p-value: 0.033

Prediction: Positive if the Bayesian score is above the estimated best cutoff value from minimizing the false positive and false negative rate.
Probability: The esimated probability that the sample is in the positive category. This assumes that the Bayesian score follows a normal distribution and is different from the prediction using a cutoff.
Enrichment: An estimate of enrichment, that is, the increased likelihood (versus random) of this sample being in the category.
Bayesian Score: The standard Laplacian-modified Bayesian score.
Mahalanobis Distance: The Mahalanobis distance (MD) is the distance to the center of the training data. The larger the MD, the less trustworthy the prediction.
Mahalanobis Distance p-value: The p-value gives the fraction of training data with an MD greater than or equal to the one for the given sample, assuming normally distributed data. The smaller the p-value, the less trustworthy the prediciton. For highly non-normal X properties (e.g., fingerprints), the MD p-value is wildly inaccurate.

Structural Similar Compounds

Name	Lenampicillin .HCl (Free base form)	Bacampicillin .HCl (Free base form)	Cyclic AMP Bucladesine
Structure	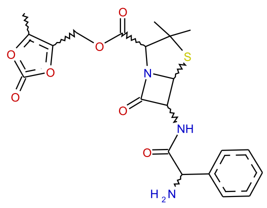	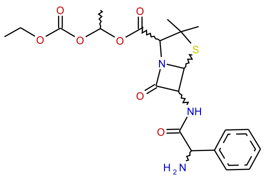	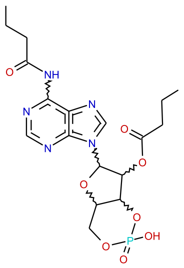
Actual Endpoint	Non-Toxic	Toxic	Non-Toxic
Predicted Endpoint	Non-Toxic	Non-Toxic	Non-Toxic
Distance	0.653	0.714	0.720
Reference	Chemotherapy 32:130-145; 1984	Chemotherapy 27:30-35; 1979	Oyo Yakuri 27(3):585-597; 1984

Model Applicability

Unknown features are fingerprint features in the query molecule, but not found in the training set.

1. All properties and OPS components are within expected ranges.

Feature Contribution

Top features for positive contribution				
**Fingerprint**	**Bit/Smiles**	**Feature Structure**	**Score**	**Toxic in training set**
SCFP_6	-1485821968	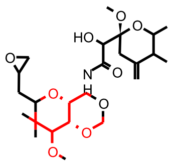 [*]OC1C([*])[*]C([*]) C(O[*])C1OC([*])[*]	0.441	3 out of 3

SCFP_6	1702664599	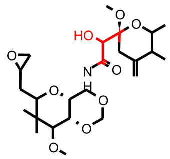 [*]OC(C([*])[*])C(=[*])[*]	0.322	4 out of 5
SCFP_6	-424515134	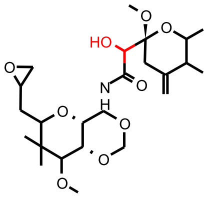 [*]C([*])O	0.275	39 out of 56
**Top Features for negative contribution**				
**Fingerprint**	**Bit/Smiles**	**Feature Structure**	**Score**	**Toxic in training set**
SCFP_6	-33065173	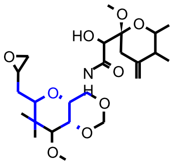 [*]CC1O[C@H](C([*])[*])[C@H]([*])[*]C1([*])[*]	-0.438	1 out of 4
SCFP_6	-36419783	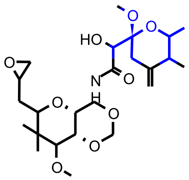 [*]O[C@@]1(C[*]C([*]) C(C)O1)C([*])[*]	-0.422	0 out of 1
SCFP_6	-132795725	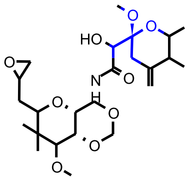 [*]CC(O[*])(O[*])C([*])[*]	-0.422	0 out of 1

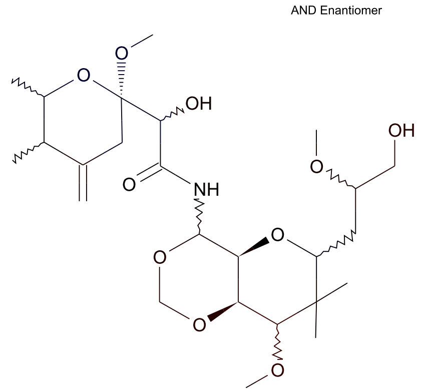

$C_{25}H_{43}NO_{10}$
Molecular Weight: 517.60962
ALogP: -9.1e-002
Rotatable Bonds: 9
Acceptors: 10
Donors: 3

Model Prediction

Prediction: Toxic
Probability: 0.641
Enrichment: 1.218
Bayesian Score: 2.398
Mahalanobis Distance: 9.601
Mahalanobis Distance p-value: 0.0545

Prediction: Positive if the Bayesian score is above the estimated best cutoff value from minimizing the false positive and false negative rate.
Probability: The esimated probability that the sample is in the positive category. This assumes that the Bayesian score follows a normal distribution and is different from the prediction using a cutoff.
Enrichment: An estimate of enrichment, that is, the increased likelihood (versus random) of this sample being in the category.
Bayesian Score: The standard Laplacian-modified Bayesian score.
Mahalanobis Distance: The Mahalanobis distance (MD) is the distance to the center of the training data. The larger the MD, the less trustworthy the prediction.
Mahalanobis Distance p-value: The p-value gives the fraction of training data with an MD greater than or equal to the one for the given sample, assuming normally distributed data. The smaller the p-value, the less trustworthy the prediciton. For highly non-normal X properties (e.g., fingerprints), the MD p-value is wildly inaccurate.

Structural Similar Compounds

Name	Bacampicillin .HCl (Free base form)	Cyclic AMP Bucladesine	Lenampicillin .HCl (Free base form)
Structure	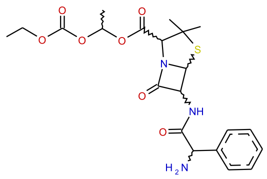	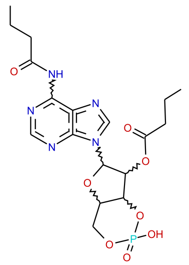	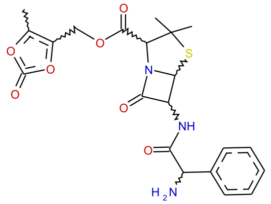
Actual Endpoint	Toxic	Non-Toxic	Non-Toxic
Predicted Endpoint	Non-Toxic	Non-Toxic	Non-Toxic
Distance	0.719	0.732	0.737
Reference	Chemotherapy 27:30-35; 1979	Oyo Yakuri 27(3):585-597; 1984	Chemotherapy 32:130-145; 1984

Model Applicability

Unknown features are fingerprint features in the query molecule, but not found in the training set.

1. All properties and OPS components are within expected ranges.

Feature Contribution

Top features for positive contribution				
Fingerprint	Bit/Smiles	Feature Structure	Score	Toxic in training set
SCFP_6	-1485821968	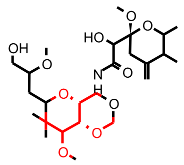 [*]OC1C([*])[*]C([*]) C(O[*])C1OC([*])[*]	0.441	3 out of 3

SCFP_6	-188384666	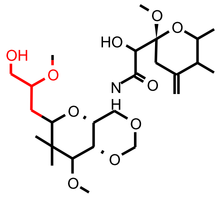 [*]CC(CO)O[*]	0.431	7 out of 8
SCFP_6	1702664599	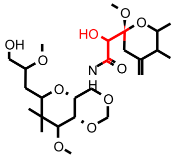 [*]OC(C([*])[*])C(=[*])[*]	0.322	4 out of 5

Top Features for negative contribution

Fingerprint	Bit/Smiles	Feature Structure	Score	Toxic in training set
SCFP_6	-33065173	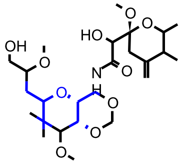 [*]CC1O[C@H](C([*])[*])[C@H]([*])[*]C1([*])[*]	-0.438	1 out of 4
SCFP_6	-132795725	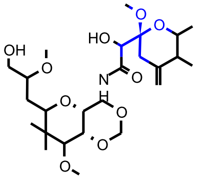 [*]CC(O[*])(O[*])C([*])[*]	-0.422	0 out of 1
SCFP_6	-36419783	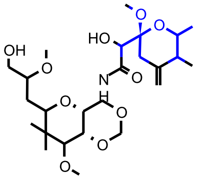 [*]O[C@@]1(C[*]C([*]) C(C)O1)C([*])[*]	-0.422	0 out of 1

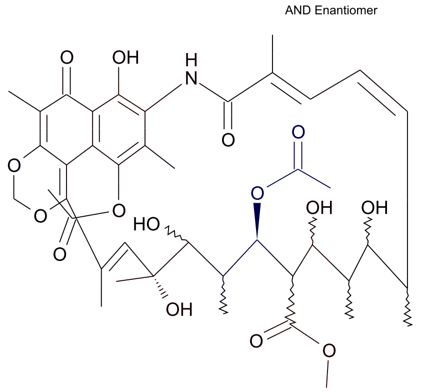

$C_{42}H_{53}NO_{15}$
Molecular Weight: 811.86791
ALogP: 2.745
Rotatable Bonds: 6
Acceptors: 15
Donors: 6

Model Prediction

Prediction: Toxic
Probability: 0.595
Enrichment: 1.131
Bayesian Score: 1.227
Mahalanobis Distance: 17.359
Mahalanobis Distance p-value: 1.14e-016

Prediction: Positive if the Bayesian score is above the estimated best cutoff value from minimizing the false positive and false negative rate.
Probability: The esimated probability that the sample is in the positive category. This assumes that the Bayesian score follows a normal distribution and is different from the prediction using a cutoff.
Enrichment: An estimate of enrichment, that is, the increased likelihood (versus random) of this sample being in the category.
Bayesian Score: The standard Laplacian-modified Bayesian score.
Mahalanobis Distance: The Mahalanobis distance (MD) is the distance to the center of the training data. The larger the MD, the less trustworthy the prediction.
Mahalanobis Distance p-value: The p-value gives the fraction of training data with an MD greater than or equal to the one for the given sample, assuming normally distributed data. The smaller the p-value, the less trustworthy the prediciton. For highly non-normal X properties (e.g., fingerprints), the MD p-value is wildly inaccurate.

Structural Similar Compounds

Name	alpha-Chaconine	Aclacinomycin a	Solanine
Structure	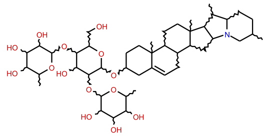	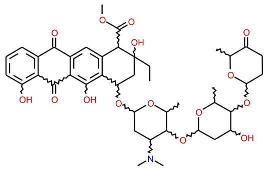	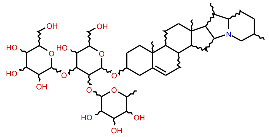
Actual Endpoint	Toxic	Toxic	Toxic
Predicted Endpoint	Toxic	Toxic	Toxic
Distance	0.762	0.808	0.926
Reference	Toxicol Appl Pharmacol 36(2):227-37; 1976	Oyo Yakuri 19(5):783-790; 1980	Toxicol Appl Pharmacol 36(2):227-37; 1976

Model Applicability

Unknown features are fingerprint features in the query molecule, but not found in the training set.

1. All properties and OPS components are within expected ranges.

Feature Contribution

Top features for positive contribution

Fingerprint	Bit/Smiles	Feature Structure	Score	Toxic in training set
SCFP_6	-1031415239	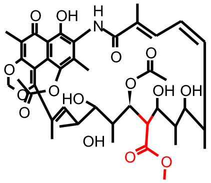 [*]C([*])C(=O)OC	0.381	2 out of 2

SCFP_6	-1971196727	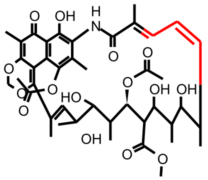 [*]\C=C\C(=[*])[*]	0.293	13 out of 18
SCFP_6	-424515134	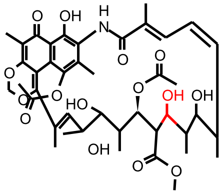 [*]C([*])O	0.275	39 out of 56

Top Features for negative contribution

Fingerprint	Bit/Smiles	Feature Structure	Score	Toxic in training set
SCFP_6	733985735	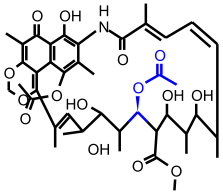 [*]COC(=O)C	-0.945	0 out of 3
SCFP_6	-1026877279	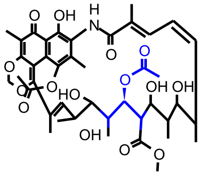 [*]C([*])C(C)OC(=O)C([*])[*]	-0.718	0 out of 2
SCFP_6	136686699	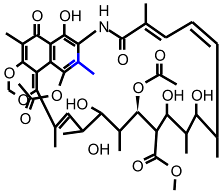 [*]:[c](:[*])C	-0.316	7 out of 19

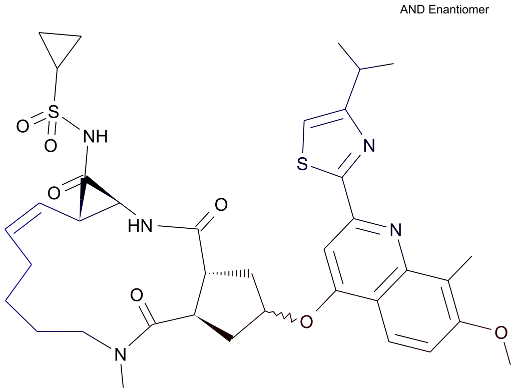

$C_{38}H_{47}N_5O_7S_2$
Molecular Weight: 749.93907
ALogP: 4.796
Rotatable Bonds: 8
Acceptors: 9
Donors: 2

Model Prediction

Prediction: Non-Toxic
Probability: 0.438
Enrichment: 0.833
Bayesian Score: -3.140
Mahalanobis Distance: 13.351
Mahalanobis Distance p-value: 9.2e-008

Prediction: Positive if the Bayesian score is above the estimated best cutoff value from minimizing the false positive and false negative rate.
Probability: The esimated probability that the sample is in the positive category. This assumes that the Bayesian score follows a normal distribution and is different from the prediction using a cutoff.
Enrichment: An estimate of enrichment, that is, the increased likelihood (versus random) of this sample being in the category.
Bayesian Score: The standard Laplacian-modified Bayesian score.
Mahalanobis Distance: The Mahalanobis distance (MD) is the distance to the center of the training data. The larger the MD, the less trustworthy the prediction.
Mahalanobis Distance p-value: The p-value gives the fraction of training data with an MD greater than or equal to the one for the given sample, assuming normally distributed data. The smaller the p-value, the less trustworthy the prediciton. For highly non-normal X properties (e.g., fingerprints), the MD p-value is wildly inaccurate.

Structural Similar Compounds

Name	Reserpate	Bromocriptine	Estramustine Phosphate Disodium (Free acid form)
Structure	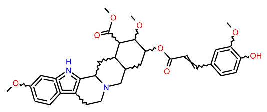	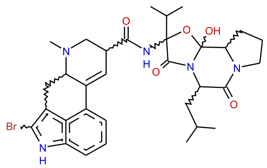	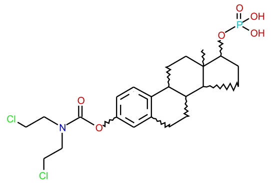
Actual Endpoint	Toxic	Non-Toxic	Non-Toxic
Predicted Endpoint	Toxic	Non-Toxic	Non-Toxic
Distance	0.675	0.778	0.819
Reference	Oyo Yakuri 18:105-124; 1979	Toxicol Lett 50:189-194; 1990	Oyo Yakuri 20(6):1219-1236; 1980

Model Applicability

Unknown features are fingerprint features in the query molecule, but not found in the training set.

1. OPS PC12 out of range. Value: 3.5244. Training min, max, SD, explained variance: -3.7514, 3.3159, 1.318, 0.0255.

Feature Contribution

Top features for positive contribution

Fingerprint	Bit/Smiles	Feature Structure	Score	Toxic in training set
SCFP_6	591469355	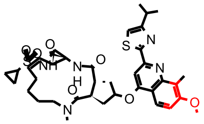 [*][c](:[*]):[c](OC):[cH]:[*]	0.411	10 out of 12

SCFP_6	-395254381	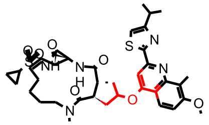 [*]:[cH]:[c](OC1C[*][*]C1):[c](:[*]):[*]	0.271	1 out of 1
SCFP_6	-109092631	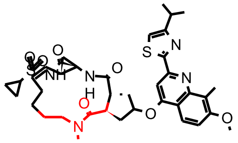 [*]CCN(C)C(=O)C([*])[*]	0.271	1 out of 1

Top Features for negative contribution

Fingerprint	Bit/Smiles	Feature Structure	Score	Toxic in training set
SCFP_6	-98332825	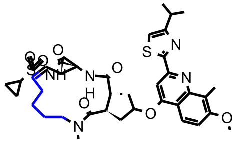 [*]CCCCC=[*]	-0.718	0 out of 2
SCFP_6	-1476112164	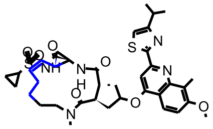 [*]CC\C=C/C1[*][*]1	-0.718	0 out of 2
SCFP_6	1260369147	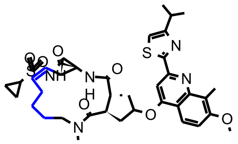 [*]CCC\C=C/[*]	-0.718	0 out of 2

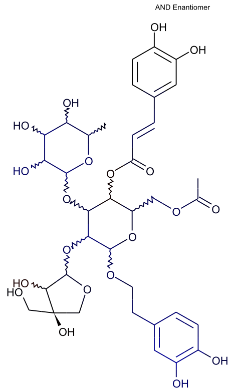

$C_{36}H_{46}O_{20}$
Molecular Weight: 798.73843
ALogP: -0.546
Rotatable Bonds: 16
Acceptors: 20
Donors: 10

Model Prediction

Prediction: Non-Carcinogen
Probability: 0.224
Enrichment: 0.698
Bayesian Score: -11.922
Mahalanobis Distance: 19.407
Mahalanobis Distance p-value: 1.07e-016

Prediction: Positive if the Bayesian score is above the estimated best cutoff value from minimizing the false positive and false negative rate.
Probability: The esimated probability that the sample is in the positive category. This assumes that the Bayesian score follows a normal distribution and is different from the prediction using a cutoff.
Enrichment: An estimate of enrichment, that is, the increased likelihood (versus random) of this sample being in the category.
Bayesian Score: The standard Laplacian-modified Bayesian score.
Mahalanobis Distance: The Mahalanobis distance (MD) is the distance to the center of the training data. The larger the MD, the less trustworthy the prediction.
Mahalanobis Distance p-value: The p-value gives the fraction of training data with an MD greater than or equal to the one for the given sample, assuming normally distributed data. The smaller the p-value, the less trustworthy the prediciton. For highly non-normal X properties (e.g., fingerprints), the MD p-value is wildly inaccurate.

Structural Similar Compounds

Name	Rifampin	Netilmicin	Rifabutin
Structure	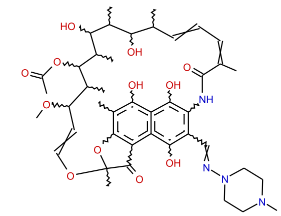	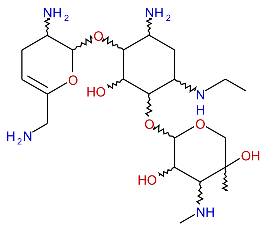	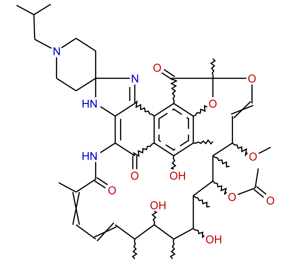
Actual Endpoint	Carcinogen	Non-Carcinogen	Non-Carcinogen
Predicted Endpoint	Carcinogen	Non-Carcinogen	Non-Carcinogen
Distance	1.505	1.527	1.649
Reference	US FDA (Centre for Drug Eval.& Res./Off. Testing & Res.) Sept. 1997	US FDA (Centre for Drug Eval.& Res./Off. Testing & Res.) Sept. 1997	US FDA (Centre for Drug Eval.& Res./Off. Testing & Res.) Sept. 1997

Model Applicability

Unknown features are fingerprint features in the query molecule, but not found in the training set.

1. Num_H_Donors out of range. Value: 10. Training min, max, mean, SD: 0, 8, 1.7372, 1.457.
2. Num_H_Acceptors out of range. Value: 20. Training min, max, mean, SD: 0, 15, 4.2436, 2.443.
3. OPS PC2 out of range. Value: 7.5498. Training min, max, SD, explained variance: -7.2665, 6.2932, 2.637, 0.0707.
4. Unknown ECFP_2 feature: 1953003528: [*]C[C@@]1(O)C[*][*]C1[*]
5. Unknown ECFP_2 feature: -176483725: [*]=C[c](:c:[*]):c:[*]

Feature Contribution

Top features for positive contribution

Fingerprint	Bit/Smiles	Feature Structure	Score	Carcinogen in training set
ECFP_6	456978524	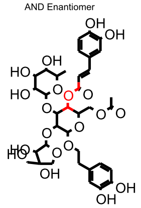 [*]C([*])OC(=[*])[*]	0.529	7 out of 12

ECFP_6	-521596699	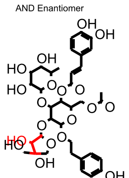 [*]C1[*][*]C([*])([*])C1O	0.451	3 out of 5
ECFP_6	-1925046727	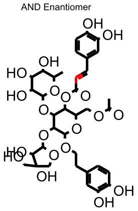 [*]C=[*]	0.391	11 out of 23

Top Features for negative contribution

Fingerprint	Bit/Smiles	Feature Structure	Score	Carcinogen in training set
ECFP_6	-1831055759	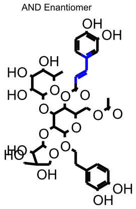 [*]\C=C\[c](:[*]):[*]	-0.805	0 out of 4
ECFP_6	456242574	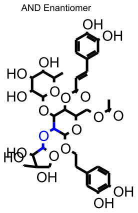 [*]C([*])OC([*])[*]	-0.805	0 out of 4
ECFP_6	-2060414325	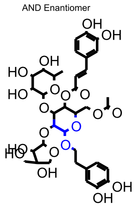 [*]OC(O[*])C([*])[*]	-0.657	0 out of 3

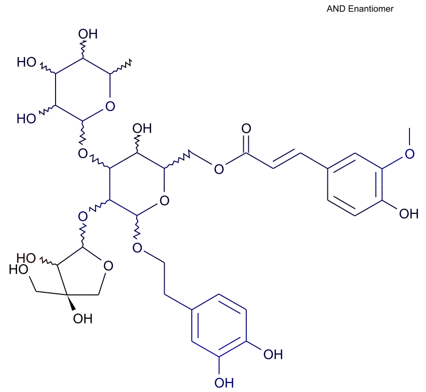

$C_{35}H_{46}O_{19}$
Molecular Weight: 770.72833
ALogP: -0.699
Rotatable Bonds: 15
Acceptors: 19
Donors: 10

Structural Similar Compounds

Name	Netilmicin	Rifampin	Rifabutin
Structure	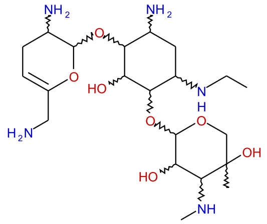	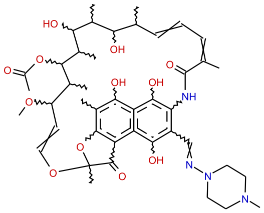	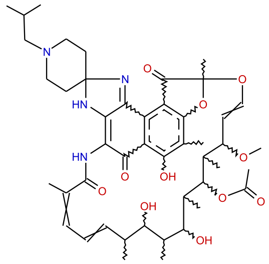
Actual Endpoint	Non-Carcinogen	Carcinogen	Non-Carcinogen
Predicted Endpoint	Non-Carcinogen	Carcinogen	Non-Carcinogen
Distance	1.420	1.447	1.594
Reference	US FDA (Centre for Drug Eval.& Res./Off. Testing & Res.) Sept. 1997	US FDA (Centre for Drug Eval.& Res./Off. Testing & Res.) Sept. 1997	US FDA (Centre for Drug Eval.& Res./Off. Testing & Res.) Sept. 1997

Model Prediction

Prediction: Non-Carcinogen
Probability: 0.207
Enrichment: 0.646
Bayesian Score: -14.043
Mahalanobis Distance: 19.037
Mahalanobis Distance p-value: 7.51e-016

Prediction: Positive if the Bayesian score is above the estimated best cutoff value from minimizing the false positive and false negative rate.
Probability: The esimated probability that the sample is in the positive category. This assumes that the Bayesian score follows a normal distribution and is different from the prediction using a cutoff.
Enrichment: An estimate of enrichment, that is, the increased likelihood (versus random) of this sample being in the category.
Bayesian Score: The standard Laplacian-modified Bayesian score.
Mahalanobis Distance: The Mahalanobis distance (MD) is the distance to the center of the training data. The larger the MD, the less trustworthy the prediction.
Mahalanobis Distance p-value: The p-value gives the fraction of training data with an MD greater than or equal to the one for the given sample, assuming normally distributed data. The smaller the p-value, the less trustworthy the prediciton. For highly non-normal X properties (e.g., fingerprints), the MD p-value is wildly inaccurate.

Model Applicability

Unknown features are fingerprint features in the query molecule, but not found in the training set.

1. Num_H_Donors out of range. Value: 10. Training min, max, mean, SD: 0, 8, 1.7372, 1.457.
2. Num_H_Acceptors out of range. Value: 19. Training min, max, mean, SD: 0, 15, 4.2436, 2.443.
3. OPS PC2 out of range. Value: 7.9262. Training min, max, SD, explained variance: -7.2665, 6.2932, 2.637, 0.0707.
4. OPS PC4 out of range. Value: 5.2279. Training min, max, SD, explained variance: -4.7116, 4.7287, 2.103, 0.0450.
5. Unknown ECFP_2 feature: 1953003528: [*]C[C@@]1(O)C[*][*]C1[*]
6. Unknown ECFP_2 feature: -176483725: [*]=C[c](:c:[*]):c:[*]

Feature Contribution

Top features for positive contribution

Fingerprint	Bit/Smiles	Feature Structure	Score	Carcinogen in training set

ECFP_6	-521596699	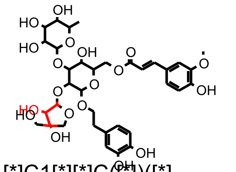 [*]C1[*][*]C([*])([*])C1O	0.451	3 out of 5
ECFP_6	-1925046727	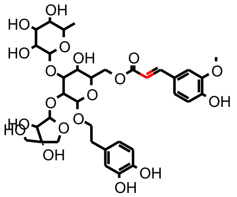 [*]C=[*]	0.391	11 out of 23
ECFP_6	865482986	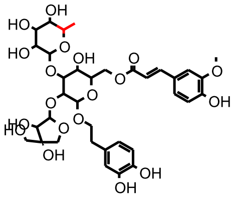 [*]C([*])C	0.350	7 out of 15

Top Features for negative contribution

Fingerprint	Bit/Smiles	Feature Structure	Score	Carcinogen in training set
ECFP_6	456242574	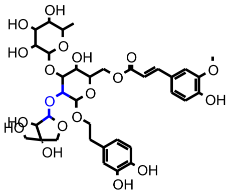 [*]C([*])OC([*])[*]	-0.805	0 out of 4
ECFP_6	-1831055759	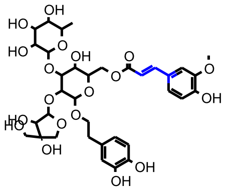 [*]\C=C\[c](:[*]):[*]	-0.805	0 out of 4

ECFP_6	-470416293	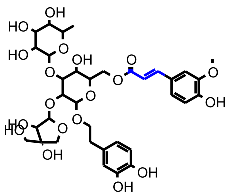 [*]\C=C\C(=[*])[*]	-0.657	0 out of 3

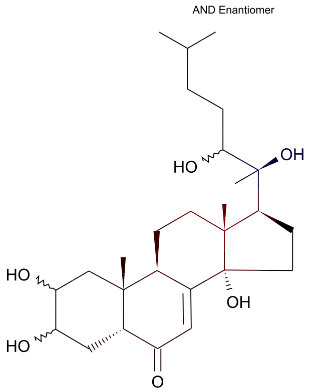

$C_{27}H_{44}O_6$
Molecular Weight: 464.63466
ALogP: 2.489
Rotatable Bonds: 5
Acceptors: 6
Donors: 5

Structural Similar Compounds

Name	Triamcinolone	Budesonide	Nadolol
Structure	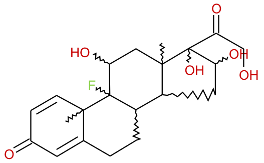	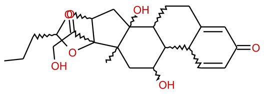	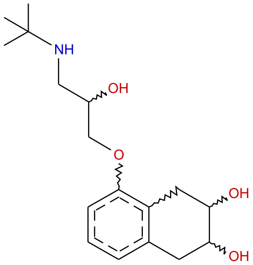
Actual Endpoint	Carcinogen	Non-Carcinogen	Non-Carcinogen
Predicted Endpoint	Carcinogen	Non-Carcinogen	Non-Carcinogen
Distance	0.764	0.785	0.789
Reference	US FDA (Centre for Drug Eval.& Res./Off. Testing & Res.) Sept. 1997	US FDA (Centre for Drug Eval.& Res./Off. Testing & Res.) Sept. 1997	US FDA (Centre for Drug Eval.& Res./Off. Testing & Res.) Sept. 1997

Model Prediction

Prediction: Carcinogen
Probability: 0.280
Enrichment: 0.873
Bayesian Score: 0.777
Mahalanobis Distance: 15.625
Mahalanobis Distance p-value: 1.58e-008

Prediction: Positive if the Bayesian score is above the estimated best cutoff value from minimizing the false positive and false negative rate.
Probability: The esimated probability that the sample is in the positive category. This assumes that the Bayesian score follows a normal distribution and is different from the prediction using a cutoff.
Enrichment: An estimate of enrichment, that is, the increased likelihood (versus random) of this sample being in the category.
Bayesian Score: The standard Laplacian-modified Bayesian score.
Mahalanobis Distance: The Mahalanobis distance (MD) is the distance to the center of the training data. The larger the MD, the less trustworthy the prediction.
Mahalanobis Distance p-value: The p-value gives the fraction of training data with an MD greater than or equal to the one for the given sample, assuming normally distributed data. The smaller the p-value, the less trustworthy the prediciton. For highly non-normal X properties (e.g., fingerprints), the MD p-value is wildly inaccurate.

Model Applicability

Unknown features are fingerprint features in the query molecule, but not found in the training set.

1. OPS PC31 out of range. Value: -2.7471. Training min, max, SD, explained variance: -2.3769, 4.158, 0.968, 0.0095.
2. Unknown ECFP_2 feature: -483166673: [*]C(=[*])[C@]1(O)C[*][*]C1([*])[*]
3. Unknown ECFP_2 feature: -1049278438: [*]C([*])([*])[C@H]1C[*][*]C1([*])[*]
4. Unknown ECFP_2 feature: 1195714366: [*]C([*])C(C)(O)C([*])[*]
5. Unknown ECFP_2 feature: -211745668: [*]C([*])C(=O)C=[*]
6. Unknown ECFP_2 feature: 80071435: [*]CC(O)C([*])([*])[*]

Feature Contribution

Top features for positive contribution				
Fingerprint	Bit/Smiles	Feature Structure	Score	Carcinogen in training set

ECFP_6	-84975114	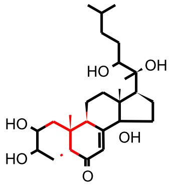<br>[*]CC(C)(C([*])[*])C([*])[*]	0.700	6 out of 8
ECFP_6	1908990050	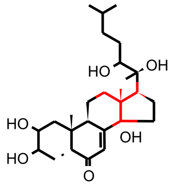<br>[*]C[C@]1(C)[C@@H]([*])[*][*]C1([*])[*]	0.700	6 out of 8
ECFP_6	-565844211	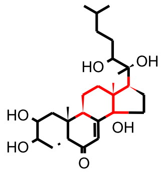<br>[*][C@@H]1[*][C@]2([*])[*][*][C@H]([*])[C@@]2(C)CC1	0.675	4 out of 5

Top Features for negative contribution

Fingerprint	Bit/Smiles	Feature Structure	Score	Carcinogen in training set
ECFP_6	2025123907	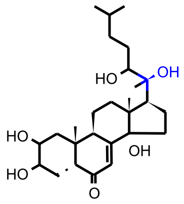<br>[*]C([*])([*])O	-1.049	0 out of 6
ECFP_6	-1910270391	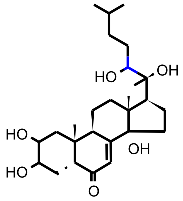<br>[*]C([*])[*]	-0.307	20 out of 89

ECFP_6	333674688	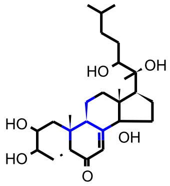 [*]C[C@@H](C(=[*])[*])C([*])([*])[*]	-0.270	0 out of 1

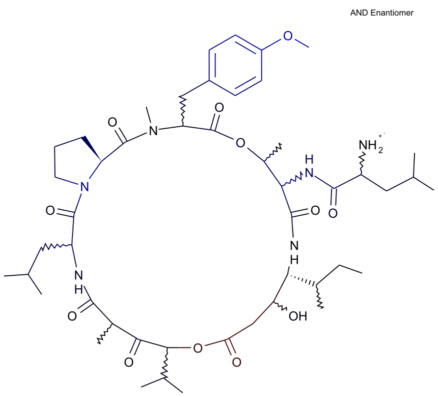

$C_{49}H_{79}N_6O_{12}$
Molecular Weight: 944.18455
ALogP: 3.176
Rotatable Bonds: 13
Acceptors: 12
Donors: 5

Model Prediction

Prediction: Non-Carcinogen
Probability: 0.206
Enrichment: 0.644
Bayesian Score: -14.119
Mahalanobis Distance: 17.887
Mahalanobis Distance p-value: 2.87e-013

Prediction: Positive if the Bayesian score is above the estimated best cutoff value from minimizing the false positive and false negative rate.
Probability: The esimated probability that the sample is in the positive category. This assumes that the Bayesian score follows a normal distribution and is different from the prediction using a cutoff.
Enrichment: An estimate of enrichment, that is, the increased likelihood (versus random) of this sample being in the category.
Bayesian Score: The standard Laplacian-modified Bayesian score.
Mahalanobis Distance: The Mahalanobis distance (MD) is the distance to the center of the training data. The larger the MD, the less trustworthy the prediction.
Mahalanobis Distance p-value: The p-value gives the fraction of training data with an MD greater than or equal to the one for the given sample, assuming normally distributed data. The smaller the p-value, the less trustworthy the prediciton. For highly non-normal X properties (e.g., fingerprints), the MD p-value is wildly inaccurate.

Structural Similar Compounds

Name	Erythromycin	Rifabutin	Rifampin
Structure	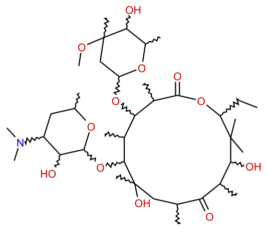	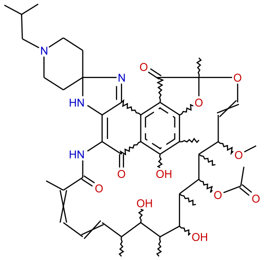	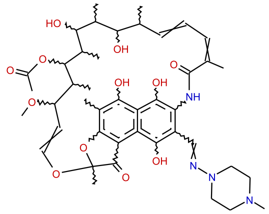
Actual Endpoint	Non-Carcinogen	Non-Carcinogen	Carcinogen
Predicted Endpoint	Non-Carcinogen	Non-Carcinogen	Carcinogen
Distance	1.004	1.004	1.067
Reference	US FDA (Centre for Drug Eval.& Res./Off. Testing & Res.) Sept. 1997	US FDA (Centre for Drug Eval.& Res./Off. Testing & Res.) Sept. 1997	US FDA (Centre for Drug Eval.& Res./Off. Testing & Res.) Sept. 1997

Model Applicability

Unknown features are fingerprint features in the query molecule, but not found in the training set.

1. Molecular_Weight out of range. Value: 944.18. Training min, max, mean, SD: 46.068, 847, 305.71, 119.
2. OPS PC2 out of range. Value: 6.7492. Training min, max, SD, explained variance: -7.2665, 6.2932, 2.637, 0.0707.
3. OPS PC4 out of range. Value: 4.8988. Training min, max, SD, explained variance: -4.7116, 4.7287, 2.103, 0.0450.
4. Unknown ECFP_2 feature: -591526139: [*][NH2+][*]
5. Unknown ECFP_2 feature: 1352327988: [*]C([*])[NH2+]C
6. Unknown ECFP_2 feature: 1691770380: [*]NC(C([*])[*])C([*])[*]
7. Unknown ECFP_2 feature: 771121623: [*]C([*])C[c](:[*]):[*]
8. Unknown ECFP_2 feature: -714938792: [*]C(=[*])C(C)C(=[*])[*]
9. Unknown ECFP_2 feature: 20550775: [*]CC([NH2+][*])C(=[*])[*]
10. Unknown ECFP_2 feature: 866401773: [*][NH2+]C

Feature Contribution

Top features for positive contribution				
Fingerprint	Bit/Smiles	Feature Structure	Score	Carcinogen in training set

ECFP_6	-2058379036	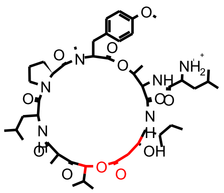 [*]C([*])CC(=O)OC([*])[*]	0.617	2 out of 2
ECFP_6	-1887539559	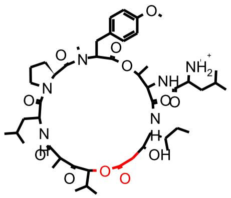 [*]CC(=O)O[*]	0.617	2 out of 2
ECFP_6	1617733200	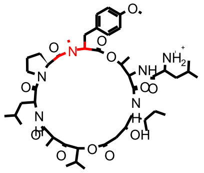 [*]C([*])N(C)C(=[*])[*]	0.424	1 out of 1

Top Features for negative contribution

Fingerprint	Bit/Smiles	Feature Structure	Score	Carcinogen in training set
ECFP_6	-484970154	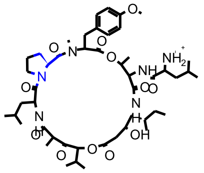 [*]N1[*][*]C[C@H]1C(= [*])[*]	-0.805	0 out of 4
ECFP_6	693720869	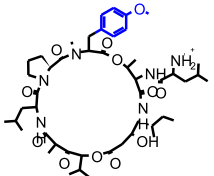 [*][c]1:[cH]:[cH]:[c] (OC):[cH]:[cH]:1	-0.805	0 out of 4

ECFP_6	-1271104377	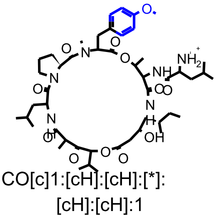 CO[c]1:[cH]:[cH]:[*]:[cH]:[cH]:1	-0.805	0 out of 4

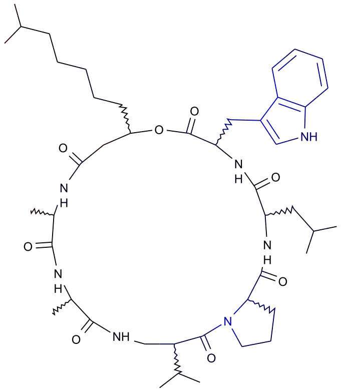

$C_{45}H_{69}N_7O_8$
Molecular Weight: 836.07145
ALogP: 4.986
Rotatable Bonds: 11
Acceptors: 8
Donors: 6

Structural Similar Compounds

Name	Erythromycin	Bromocriptine	Rifabutin
Structure	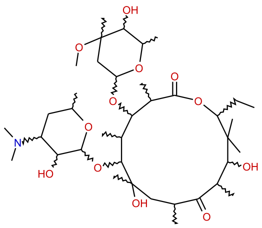	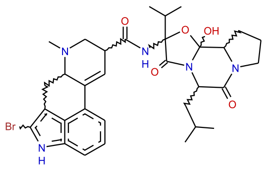	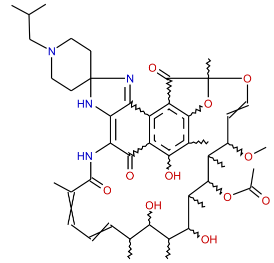
Actual Endpoint	Non-Carcinogen	Non-Carcinogen	Non-Carcinogen
Predicted Endpoint	Non-Carcinogen	Non-Carcinogen	Non-Carcinogen
Distance	1.091	1.098	1.113
Reference	US FDA (Centre for Drug Eval.& Res./Off. Testing & Res.) Sept. 1997	US FDA (Centre for Drug Eval.& Res./Off. Testing & Res.) Sept. 1997	US FDA (Centre for Drug Eval.& Res./Off. Testing & Res.) Sept. 1997

Model Prediction

Prediction: Non-Carcinogen
Probability: 0.227
Enrichment: 0.708
Bayesian Score: -10.323
Mahalanobis Distance: 17.509
Mahalanobis Distance p-value: 1.92e-012

Prediction: Positive if the Bayesian score is above the estimated best cutoff value from minimizing the false positive and false negative rate.
Probability: The esimated probability that the sample is in the positive category. This assumes that the Bayesian score follows a normal distribution and is different from the prediction using a cutoff.
Enrichment: An estimate of enrichment, that is, the increased likelihood (versus random) of this sample being in the category.
Bayesian Score: The standard Laplacian-modified Bayesian score.
Mahalanobis Distance: The Mahalanobis distance (MD) is the distance to the center of the training data. The larger the MD, the less trustworthy the prediction.
Mahalanobis Distance p-value: The p-value gives the fraction of training data with an MD greater than or equal to the one for the given sample, assuming normally distributed data. The smaller the p-value, the less trustworthy the prediciton. For highly non-normal X properties (e.g., fingerprints), the MD p-value is wildly inaccurate.

Model Applicability

Unknown features are fingerprint features in the query molecule, but not found in the training set.

1. All properties and OPS components are within expected ranges.
2. Unknown ECFP_2 feature: 771121623: [*]C([*])C[c](:[*]):[*]
3. Unknown ECFP_2 feature: 1863041499: [*]NC(C)C(=[*])[*]
4. Unknown ECFP_2 feature: -2096927833: [*]CC(C([*])[*])C(=[*])[*]

Feature Contribution

Top features for positive contribution				
Fingerprint	Bit/Smiles	Feature Structure	Score	Carcinogen in training set
ECFP_6	-1291950249	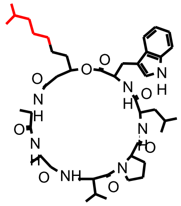 [*]CCCC(C)C	0.424	1 out of 1

ECFP_6	-745187652	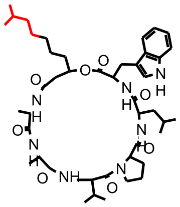 [*]CCC(C)C	0.424	1 out of 1
ECFP_6	-953984246	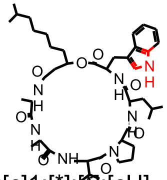 [*]:[c]1:[*]:[*]:[cH]:[nH]:1	0.364	4 out of 8
**Top Features for negative contribution**				
**Fingerprint**	**Bit/Smiles**	**Feature Structure**	**Score**	**Carcinogen in training set**
ECFP_6	-484970154	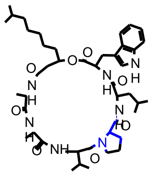 [*]N1[*][*]C[C@H]1C(=[*])[*]	-0.805	0 out of 4
ECFP_6	-1659633832	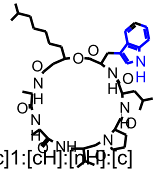 [*][c]1:[cH]:[nH]:[c]2:[cH]:[cH]:[*]:[cH]:[c]:1:2	-0.657	0 out of 3
ECFP_6	1526862590	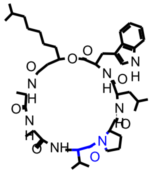 [*]C([*])C(=O)N([*])[*]	-0.638	1 out of 9

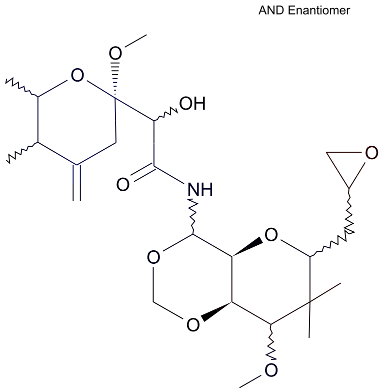

$C_{24}H_{39}NO_9$
Molecular Weight: 485.56776
ALogP: 0.267
Rotatable Bonds: 7
Acceptors: 9
Donors: 2

Structural Similar Compounds

Name	Bacampicillin	Budesonide	Amlodipine
Structure	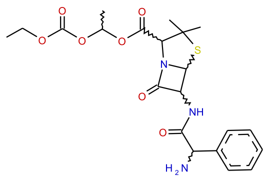	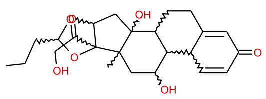	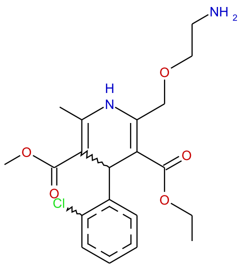
Actual Endpoint	Non-Carcinogen	Non-Carcinogen	Non-Carcinogen
Predicted Endpoint	Non-Carcinogen	Non-Carcinogen	Non-Carcinogen
Distance	0.713	0.765	0.775
Reference	US FDA (Centre for Drug Eval.& Res./Off. Testing & Res.) Sept. 1997	US FDA (Centre for Drug Eval.& Res./Off. Testing & Res.) Sept. 1997	US FDA (Centre for Drug Eval.& Res./Off. Testing & Res.) Sept. 1997

Model Prediction

Prediction: Non-Carcinogen
Probability: 0.211
Enrichment: 0.657
Bayesian Score: -4.217
Mahalanobis Distance: 16.163
Mahalanobis Distance p-value: 1.33e-009

Prediction: Positive if the Bayesian score is above the estimated best cutoff value from minimizing the false positive and false negative rate.
Probability: The esimated probability that the sample is in the positive category. This assumes that the Bayesian score follows a normal distribution and is different from the prediction using a cutoff.
Enrichment: An estimate of enrichment, that is, the increased likelihood (versus random) of this sample being in the category.
Bayesian Score: The standard Laplacian-modified Bayesian score.
Mahalanobis Distance: The Mahalanobis distance (MD) is the distance to the center of the training data. The larger the MD, the less trustworthy the prediction.
Mahalanobis Distance p-value: The p-value gives the fraction of training data with an MD greater than or equal to the one for the given sample, assuming normally distributed data. The smaller the p-value, the less trustworthy the prediciton. For highly non-normal X properties (e.g., fingerprints), the MD p-value is wildly inaccurate.

Model Applicability

Unknown features are fingerprint features in the query molecule, but not found in the training set.

1. All properties and OPS components are within expected ranges.
2. Unknown ECFP_2 feature: -457637478: [*]NC(O[*])C([*])[*]
3. Unknown ECFP_2 feature: 837844420: [*]CC(O[*])(O[*])C([*])[*]
4. Unknown ECFP_2 feature: 825070174: [*]C(=[*])C(O)C([*])([*])[*]

Feature Contribution

Top features for positive contribution				
**Fingerprint**	**Bit/Smiles**	**Feature Structure**	**Score**	**Carcinogen in training set**
ECFP_6	728225615	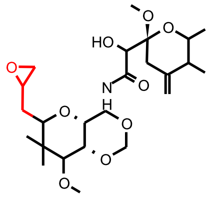 [*]CC1CO1	0.424	1 out of 1

ECFP_6	859433814	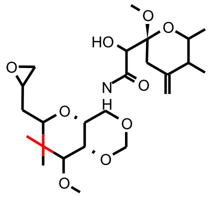 [*]C([*])([*])C	0.408	14 out of 29
ECFP_6	-554480104	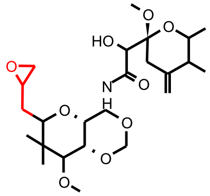 [*]CC(C[*])O[*]	0.364	4 out of 8
**Top Features for negative contribution**				
**Fingerprint**	**Bit/Smiles**	**Feature Structure**	**Score**	**Carcinogen in training set**
ECFP_6	407900312	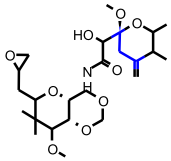 [*]C(=[*])CC([*])([*])[*]	-0.657	0 out of 3
ECFP_6	-2091181441	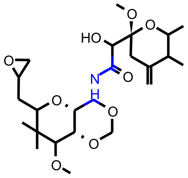 [*]C([*])NC(=[*])[*]	-0.638	1 out of 9
ECFP_6	1535429263	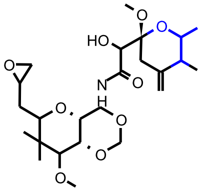 [*]OC(C)C([*])[*]	-0.482	0 out of 2

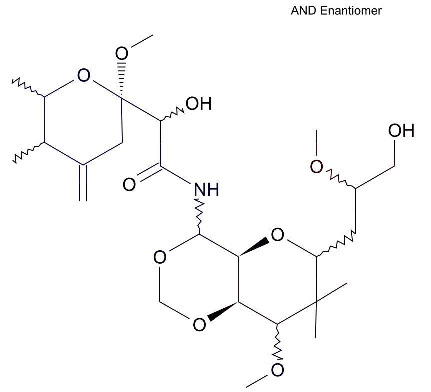

$C_{25}H_{43}NO_{10}$
Molecular Weight: 517.60962
ALogP: -9.1e-002
Rotatable Bonds: 9
Acceptors: 10
Donors: 3

Structural Similar Compounds

Name	Bacampicillin	Amlodipine	Dipyridamole
Structure	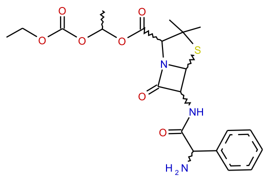	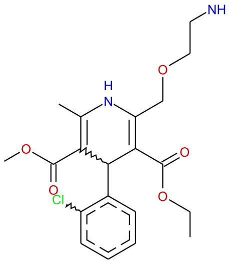	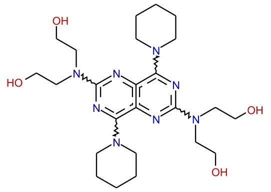
Actual Endpoint	Non-Carcinogen	Non-Carcinogen	Non-Carcinogen
Predicted Endpoint	Non-Carcinogen	Non-Carcinogen	Non-Carcinogen
Distance	0.729	0.851	0.853
Reference	US FDA (Centre for Drug Eval.& Res./Off. Testing & Res.) Sept. 1997	US FDA (Centre for Drug Eval.& Res./Off. Testing & Res.) Sept. 1997	US FDA (Centre for Drug Eval.& Res./Off. Testing & Res.) Sept. 1997

Model Prediction

Prediction: Non-Carcinogen
Probability: 0.209
Enrichment: 0.652
Bayesian Score: -4.513
Mahalanobis Distance: 15.242
Mahalanobis Distance p-value: 8.68e-008

Prediction: Positive if the Bayesian score is above the estimated best cutoff value from minimizing the false positive and false negative rate.
Probability: The esimated probability that the sample is in the positive category. This assumes that the Bayesian score follows a normal distribution and is different from the prediction using a cutoff.
Enrichment: An estimate of enrichment, that is, the increased likelihood (versus random) of this sample being in the category.
Bayesian Score: The standard Laplacian-modified Bayesian score.
Mahalanobis Distance: The Mahalanobis distance (MD) is the distance to the center of the training data. The larger the MD, the less trustworthy the prediction.
Mahalanobis Distance p-value: The p-value gives the fraction of training data with an MD greater than or equal to the one for the given sample, assuming normally distributed data. The smaller the p-value, the less trustworthy the prediciton. For highly non-normal X properties (e.g., fingerprints), the MD p-value is wildly inaccurate.

Model Applicability

Unknown features are fingerprint features in the query molecule, but not found in the training set.

1. All properties and OPS components are within expected ranges.
2. Unknown ECFP_2 feature: -410173153: [*]C([*])OC
3. Unknown ECFP_2 feature: -457637478: [*]NC(O[*])C([*])[*]
4. Unknown ECFP_2 feature: 837844420: [*]CC(O[*])(O[*])C([*])[*]
5. Unknown ECFP_2 feature: 825070174: [*]C(=[*])C(O)C([*])([*])[*]

Feature Contribution

Top features for positive contribution

Fingerprint	Bit/Smiles	Feature Structure	Score	Carcinogen in training set
ECFP_6	1066876733	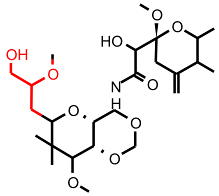 [*]CC(CO)O[*]	0.424	1 out of 1

ECFP_6	197414153	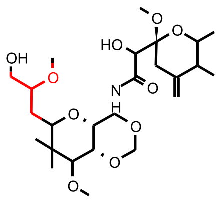 [*]CC(C[*])O[*]	0.424	1 out of 1
ECFP_6	859433814	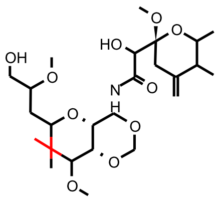 [*]C([*])([*])C	0.408	14 out of 29

Top Features for negative contribution

Fingerprint	Bit/Smiles	Feature Structure	Score	Carcinogen in training set
ECFP_6	407900312	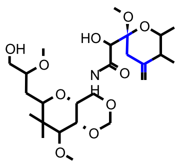 [*]C(=[*])CC([*])([*])[*]	-0.657	0 out of 3
ECFP_6	-2091181441	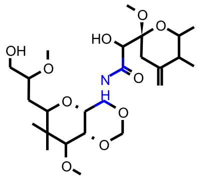 [*]C([*])NC(=[*])[*]	-0.638	1 out of 9
ECFP_6	-89681466	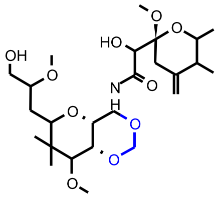 [*]OCO[*]	-0.482	0 out of 2

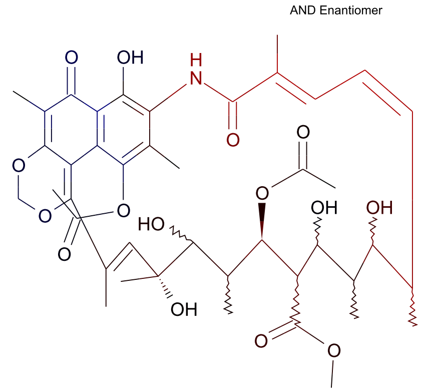

$C_{42}H_{53}NO_{15}$
Molecular Weight: 811.86791
ALogP: 2.745
Rotatable Bonds: 6
Acceptors: 15
Donors: 6

Model Prediction

Prediction: Carcinogen
Probability: 0.321
Enrichment: 1.000
Bayesian Score: 2.288
Mahalanobis Distance: 11.738
Mahalanobis Distance p-value: 0.0211

Prediction: Positive if the Bayesian score is above the estimated best cutoff value from minimizing the false positive and false negative rate.
Probability: The esimated probability that the sample is in the positive category. This assumes that the Bayesian score follows a normal distribution and is different from the prediction using a cutoff.
Enrichment: An estimate of enrichment, that is, the increased likelihood (versus random) of this sample being in the category.
Bayesian Score: The standard Laplacian-modified Bayesian score.
Mahalanobis Distance: The Mahalanobis distance (MD) is the distance to the center of the training data. The larger the MD, the less trustworthy the prediction.
Mahalanobis Distance p-value: The p-value gives the fraction of training data with an MD greater than or equal to the one for the given sample, assuming normally distributed data. The smaller the p-value, the less trustworthy the prediciton. For highly non-normal X properties (e.g., fingerprints), the MD p-value is wildly inaccurate.

Structural Similar Compounds

Name	Rifampin	Rifabutin	Erythromycin
Structure	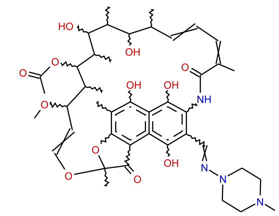	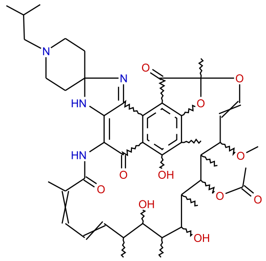	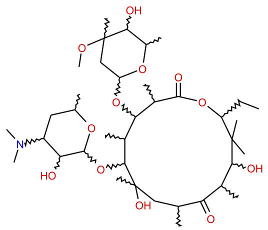
Actual Endpoint	Carcinogen	Non-Carcinogen	Non-Carcinogen
Predicted Endpoint	Carcinogen	Non-Carcinogen	Non-Carcinogen
Distance	0.453	0.596	0.829
Reference	US FDA (Centre for Drug Eval.& Res./Off. Testing & Res.) Sept. 1997	US FDA (Centre for Drug Eval.& Res./Off. Testing & Res.) Sept. 1997	US FDA (Centre for Drug Eval.& Res./Off. Testing & Res.) Sept. 1997

Model Applicability

Unknown features are fingerprint features in the query molecule, but not found in the training set.

1. All properties and OPS components are within expected ranges.
2. Unknown ECFP_2 feature: -40769921: [*]C([*])[C@](C)(O)C=[*]
3. Unknown ECFP_2 feature: 464446479: [*]C(=CC([*])([*])[*])[*]
4. Unknown ECFP_2 feature: 1796421070: [*]OC(=C([*])[*])C(=[*])[*]
5. Unknown ECFP_2 feature: 1792159373: [*]C(=C(C)C(=[*])[*])[*]

Feature Contribution

Top features for positive contribution

Fingerprint	Bit/Smiles	Feature Structure	Score	Carcinogen in training set
ECFP_6	-3067141	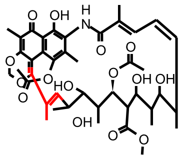 [*]\C=C(/C)\C(=[*])[*]	0.675	4 out of 5

ECFP_6	-812320335	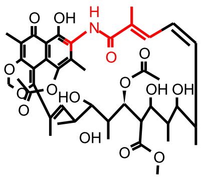 [*]\C=C(/C)\C(=O)N[c](:[*]):[*]	0.581	3 out of 4
ECFP_6	456978524	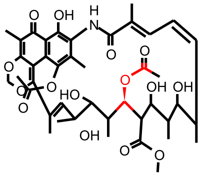 [*]C([*])OC(=[*])[*]	0.529	7 out of 12

Top Features for negative contribution

Fingerprint	Bit/Smiles	Feature Structure	Score	Carcinogen in training set
ECFP_6	1717462980	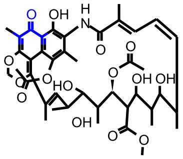 [*]C(=[*])C(=O)[c](:[*]):[*]	-1.246	0 out of 8
ECFP_6	305695353	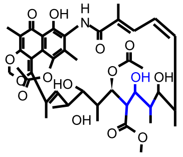 [*]C([*])C(O)C([*])[*]	-0.638	1 out of 9
ECFP_6	-89681466	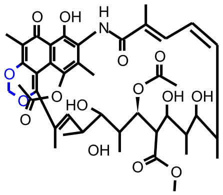 [*]OCO[*]	-0.482	0 out of 2

Simeprevir

TOPKAT_Mouse_Female_FDA_None_vs_Carcinogen

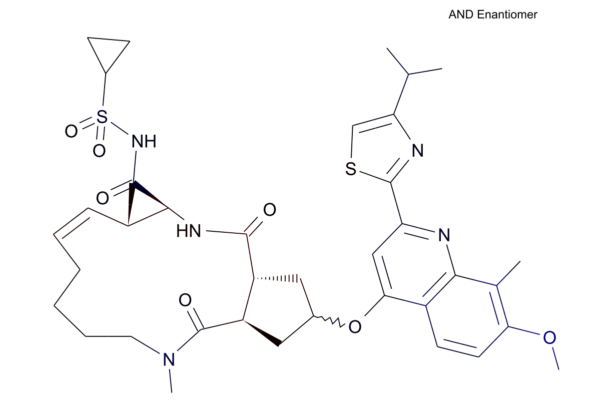

$C_{38}H_{47}N_5O_7S_2$

Molecular Weight: 749.93907

ALogP: 4.796

Rotatable Bonds: 8

Acceptors: 9

Donors: 2

Structural Similar Compounds

Name	Reserpine	Bromocriptine	Itraconazole
Structure	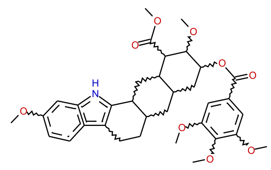	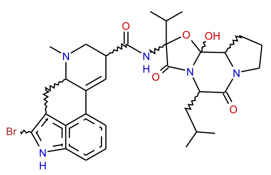	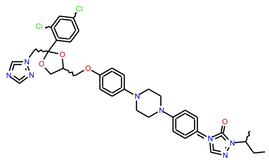
Actual Endpoint	Carcinogen	Non-Carcinogen	Non-Carcinogen
Predicted Endpoint	Carcinogen	Non-Carcinogen	Non-Carcinogen
Distance	0.783	0.847	0.870
Reference	US FDA (Centre for Drug Eval.& Res./Off. Testing & Res.) Sept. 1997	US FDA (Centre for Drug Eval.& Res./Off. Testing & Res.) Sept. 1997	US FDA (Centre for Drug Eval.& Res./Off. Testing & Res.) Sept. 1997

Model Prediction

Prediction: Non-Carcinogen

Probability: 0.207

Enrichment: 0.646

Bayesian Score: -4.971

Mahalanobis Distance: 15.384

Mahalanobis Distance p-value: 4.64e-008

Prediction: Positive if the Bayesian score is above the estimated best cutoff value from minimizing the false positive and false negative rate.
Probability: The esimated probability that the sample is in the positive category. This assumes that the Bayesian score follows a normal distribution and is different from the prediction using a cutoff.
Enrichment: An estimate of enrichment, that is, the increased likelihood (versus random) of this sample being in the category.
Bayesian Score: The standard Laplacian-modified Bayesian score.
Mahalanobis Distance: The Mahalanobis distance (MD) is the distance to the center of the training data. The larger the MD, the less trustworthy the prediction.
Mahalanobis Distance p-value: The p-value gives the fraction of training data with an MD greater than or equal to the one for the given sample, assuming normally distributed data. The smaller the p-value, the less trustworthy the prediciton. For highly non-normal X properties (e.g., fingerprints), the MD p-value is wildly inaccurate.

Model Applicability

Unknown features are fingerprint features in the query molecule, but not found in the training set.

1. OPS PC4 out of range. Value: 5.1697. Training min, max, SD, explained variance: -4.7116, 4.7287, 2.103, 0.0450.
2. OPS PC14 out of range. Value: -3.7168. Training min, max, SD, explained variance: -3.5274, 4.3994, 1.457, 0.0216.
3. OPS PC17 out of range. Value: 3.3515. Training min, max, SD, explained variance: -4.7306, 3.3103, 1.364, 0.0189.
4. Unknown ECFP_2 feature: -309676563: [*]N[C@]1(C[C@H]1[*])C(=[*])[*]
5. Unknown ECFP_2 feature: -1049290660: [*]C1([*])C[C@H]1C=[*]
6. Unknown ECFP_2 feature: 360408239: [*]C\C=C/[*]
7. Unknown ECFP_2 feature: 1616402542: [*]CN(C)C(=[*])[*]
8. Unknown ECFP_2 feature: -1818486371: [*]NC(=O)C1([*])[*][*]1
9. Unknown ECFP_2 feature: 1310940530: [*]C([*])O[c](:[*]):[*]
10. Unknown ECFP_2 feature: -253227249: [*]:[c](:[*])[c]1:n:[*]:[*]:s:1
11. Unknown ECFP_2 feature: 733491677: [*]:[c](:[*])C(C)C
12. Unknown ECFP_2 feature: 2121941848: [*]NS(=O)(=O)C1[*][*]1
13. Unknown ECFP_2 feature: -622223421: [*]S(=[*])(=[*])C1CC1

Feature Contribution

Top features for positive contribution

Fingerprint	Bit/Smiles	Feature Structure	Score	Carcinogen in training set
ECFP_6	-2095963820	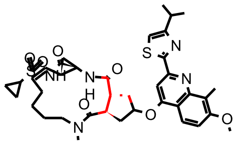 [*][C@@H]1[*][*]C[C@H]1C(=[*])[*]	0.891	12 out of 14
ECFP_6	2082767335	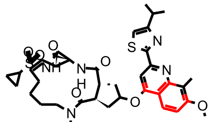 [*][c](:[*]):[c]1:[cH]:[cH]:[c]([*]):[*]:[c]:1:[*]	0.617	2 out of 2
ECFP_6	-1331088410	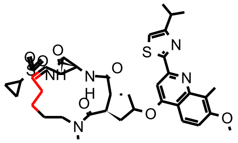 [*]CCC=[*]	0.442	2 out of 3

Top Features for negative contribution

Fingerprint	Bit/Smiles	Feature Structure	Score	Carcinogen in training set
ECFP_6	2077607946	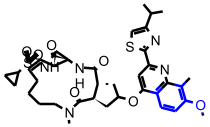 [*]O[c]1:[cH]:[cH]:[c](:[*]):[*]:[c]:1[*]	-1.152	0 out of 7

ECFP_6	1526862590	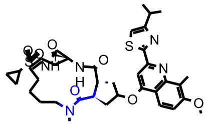<br>[*]C([*])C(=O)N([*])[*]	-0.638	1 out of 9
ECFP_6	1307307440	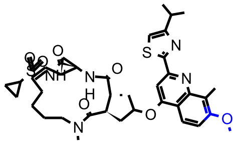<br>[*]:[c](:[*])OC	-0.558	4 out of 25

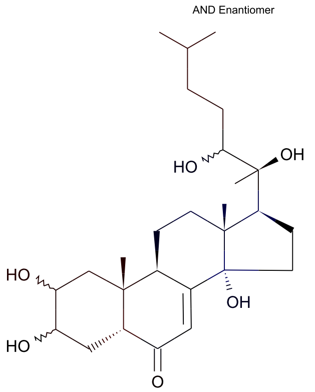

$C_{27}H_{44}O_6$
Molecular Weight: 464.63466
ALogP: 2.489
Rotatable Bonds: 5
Acceptors: 6
Donors: 5

Model Prediction

Prediction: Multiple-Carcinogen
Probability: 0.467
Enrichment: 1.139
Bayesian Score: 1.502
Mahalanobis Distance: 18.032
Mahalanobis Distance p-value: 2.27e-009

Prediction: Positive if the Bayesian score is above the estimated best cutoff value from minimizing the false positive and false negative rate.
Probability: The esimated probability that the sample is in the positive category. This assumes that the Bayesian score follows a normal distribution and is different from the prediction using a cutoff.
Enrichment: An estimate of enrichment, that is, the increased likelihood (versus random) of this sample being in the category.
Bayesian Score: The standard Laplacian-modified Bayesian score.
Mahalanobis Distance: The Mahalanobis distance (MD) is the distance to the center of the training data. The larger the MD, the less trustworthy the prediction.
Mahalanobis Distance p-value: The p-value gives the fraction of training data with an MD greater than or equal to the one for the given sample, assuming normally distributed data. The smaller the p-value, the less trustworthy the prediciton. For highly non-normal X properties (e.g., fingerprints), the MD p-value is wildly inaccurate.

Structural Similar Compounds

Name	Triamcinolone	Labetalol	Glimepride
Structure	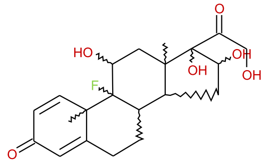	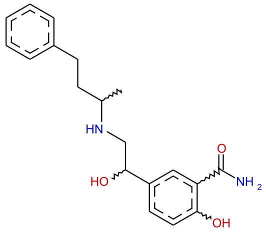	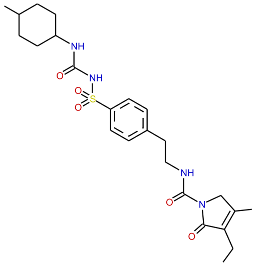
Actual Endpoint	Single-Carcinogen	Single-Carcinogen	Single-Carcinogen
Predicted Endpoint	Single-Carcinogen	Single-Carcinogen	Single-Carcinogen
Distance	0.747	0.836	0.863
Reference	US FDA (Centre for Drug Eval.& Res./Off. Testing & Res.) Sept. 1997	US FDA (Centre for Drug Eval.& Res./Off. Testing & Res.) Sept. 1997	US FDA (Centre for Drug Eval.& Res./Off. Testing & Res.) Sept. 1997

Model Applicability

Unknown features are fingerprint features in the query molecule, but not found in the training set.

1. All properties and OPS components are within expected ranges.
2. Unknown ECFP_2 feature: 2025123907: [*]C([*])([*])O
3. Unknown ECFP_2 feature: -483166673: [*]C(=[*])[C@]1(O)C[*][*]C1([*])[*]
4. Unknown ECFP_2 feature: -1049278438: [*]C([*])([*])[C@H]1C[*][*]C1([*])[*]
5. Unknown ECFP_2 feature: 333674688: [*]C[C@@H](C(=[*])[*])C([*])([*])[*]
6. Unknown ECFP_2 feature: 1195714366: [*]C([*])C(C)(O)C([*])[*]
7. Unknown ECFP_2 feature: -211745668: [*]C([*])C(=O)C=[*]
8. Unknown ECFP_2 feature: 80071435: [*]CC(O)C([*])([*])[*]

Feature Contribution

Top features for positive contribution				
Fingerprint	Bit/Smiles	Feature Structure	Score	Multiple-Carcinogen in training set

ECFP_4	-84975114	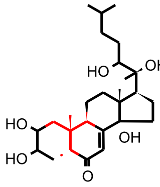 [*]CC(C)(C([*])[*])C([*])[*]	0.707	6 out of 6
ECFP_4	2024749573	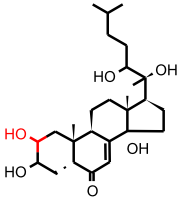 [*]C([*])O	0.494	8 out of 11
ECFP_4	-801490360	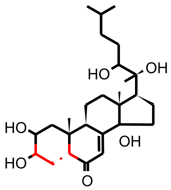 [*]C([*])CC([*])[*]	0.422	8 out of 12

Top Features for negative contribution

Fingerprint	Bit/Smiles	Feature Structure	Score	Multiple-Carcinogen in training set
ECFP_4	-565844211	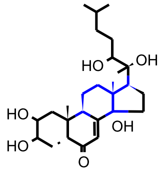 [*][C@@H]1[*][C@]2([*])[*][*][C@H]([*])[C@@]2(C)CC1	-0.968	0 out of 4
ECFP_4	2018700401	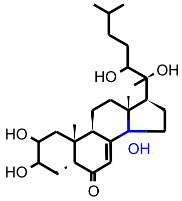 [*]C([*])([*])O	-0.657	1 out of 7

ECFP_4	1908990050	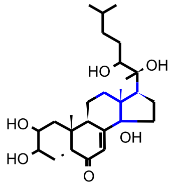[*]C[C@]1(C)[C@@H]([*])[*][*]C1([*])[*]	-0.545	1 out of 6

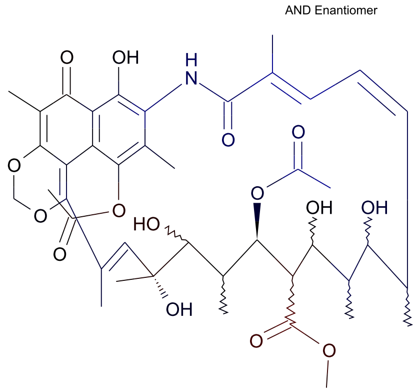

$C_{42}H_{53}NO_{15}$
Molecular Weight: 811.86791
ALogP: 2.745
Rotatable Bonds: 6
Acceptors: 15
Donors: 6

Structural Similar Compounds

Name	Rifampin	Sulfasalazine	Reserpine
Structure	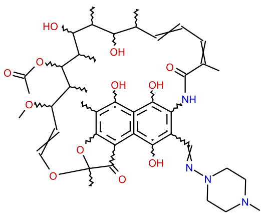	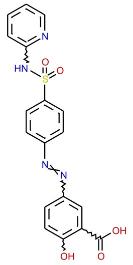	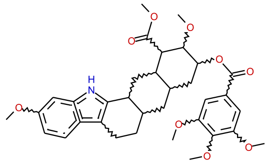
Actual Endpoint	Single-Carcinogen	Single-Carcinogen	Multiple-Carcinogen
Predicted Endpoint	Single-Carcinogen	Single-Carcinogen	Multiple-Carcinogen
Distance	0.412	1.574	1.596
Reference	US FDA (Centre for Drug Eval.& Res./Off. Testing & Res.) Sept. 1997	US FDA (Centre for Drug Eval.& Res./Off. Testing & Res.) Sept. 1997	US FDA (Centre for Drug Eval.& Res./Off. Testing & Res.) Sept. 1997

Model Prediction

Prediction: Single-Carcinogen
Probability: 0.240
Enrichment: 0.585
Bayesian Score: -6.577
Mahalanobis Distance: 11.645
Mahalanobis Distance p-value: 0.000802

Prediction: Positive if the Bayesian score is above the estimated best cutoff value from minimizing the false positive and false negative rate.
Probability: The esimated probability that the sample is in the positive category. This assumes that the Bayesian score follows a normal distribution and is different from the prediction using a cutoff.
Enrichment: An estimate of enrichment, that is, the increased likelihood (versus random) of this sample being in the category.
Bayesian Score: The standard Laplacian-modified Bayesian score.
Mahalanobis Distance: The Mahalanobis distance (MD) is the distance to the center of the training data. The larger the MD, the less trustworthy the prediction.
Mahalanobis Distance p-value: The p-value gives the fraction of training data with an MD greater than or equal to the one for the given sample, assuming normally distributed data. The smaller the p-value, the less trustworthy the prediciton. For highly non-normal X properties (e.g., fingerprints), the MD p-value is wildly inaccurate.

Model Applicability

Unknown features are fingerprint features in the query molecule, but not found in the training set.

1. OPS PC7 out of range. Value: -4.1734. Training min, max, SD, explained variance: -4.1501, 4.2431, 1.518, 0.0422.
2. OPS PC15 out of range. Value: -2.4651. Training min, max, SD, explained variance: -2.3157, 2.7205, 1.023, 0.0192.
3. Unknown ECFP_2 feature: -40769921: [*]C([*])[C@](C)(O)C=[*]
4. Unknown ECFP_2 feature: 464446479: [*]C(=CC([*])([*])[*])[*]
5. Unknown ECFP_2 feature: 1796421070: [*]OC(=C([*])[*])C(=[*])[*]
6. Unknown ECFP_2 feature: 1790105651: [*]C(=C(C(=[*])[*])[c](:[*]):[*])[*]
7. Unknown ECFP_2 feature: 1792159373: [*]C(=C(C)C(=[*])[*])[*]
8. Unknown ECFP_2 feature: -89681466: [*]OCO[*]
9. Unknown ECFP_2 feature: 1717462980: [*]C(=[*])C(=O)[c](:[*]):[*]

Feature Contribution

Top features for positive contribution

Fingerprint	Bit/Smiles	Feature Structure	Score	Multiple-Carcinogen in training set

ECFP_4	-521596699	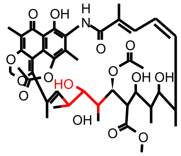 [*]C([*])C(O)C([*])([*])[*]	0.586	3 out of 3
ECFP_4	-1683911134	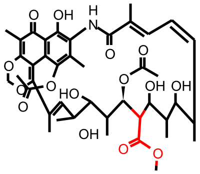 [*]OC(=O)C([*])[*]	0.501	2 out of 2
ECFP_4	2024749573	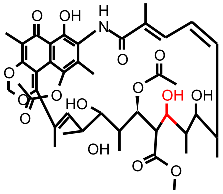 [*]C([*])O	0.494	8 out of 11
**Top Features for negative contribution**				
**Fingerprint**	**Bit/Smiles**	**Feature Structure**	**Score**	**Multiple-Carcinogen in training set**
ECFP_4	866218936	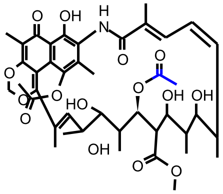 [*]C(=[*])C	-1.239	0 out of 6
ECFP_4	-3067141	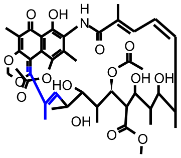 [*]\C=C(/C)\C(=[*])[*]	-0.968	0 out of 4

ECFP_4	-812320335	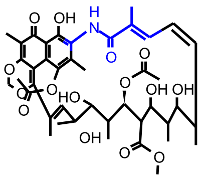 [*]\C=C(/C)\C(=O)N[c](:[*]):[*]	-0.800	0 out of 3

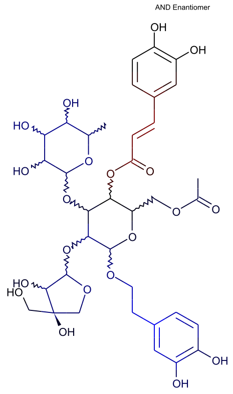

$C_{36}H_{46}O_{20}$
Molecular Weight: 798.73843
ALogP: -0.546
Rotatable Bonds: 16
Acceptors: 20
Donors: 10

Model Prediction

Prediction: Non-Carcinogen
Probability: 0.129
Enrichment: 0.440
Bayesian Score: -11.300
Mahalanobis Distance: 15.541
Mahalanobis Distance p-value: 7.88e-009

Prediction: Positive if the Bayesian score is above the estimated best cutoff value from minimizing the false positive and false negative rate.
Probability: The esimated probability that the sample is in the positive category. This assumes that the Bayesian score follows a normal distribution and is different from the prediction using a cutoff.
Enrichment: An estimate of enrichment, that is, the increased likelihood (versus random) of this sample being in the category.
Bayesian Score: The standard Laplacian-modified Bayesian score.
Mahalanobis Distance: The Mahalanobis distance (MD) is the distance to the center of the training data. The larger the MD, the less trustworthy the prediction.
Mahalanobis Distance p-value: The p-value gives the fraction of training data with an MD greater than or equal to the one for the given sample, assuming normally distributed data. The smaller the p-value, the less trustworthy the prediciton. For highly non-normal X properties (e.g., fingerprints), the MD p-value is wildly inaccurate.

Structural Similar Compounds

Name	Rifampin	Netilmicin	Rifabutin
Structure	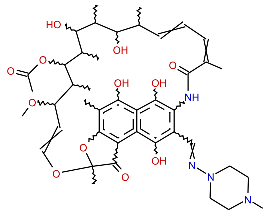	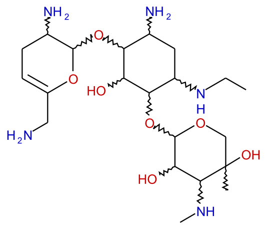	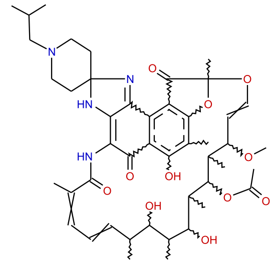
Actual Endpoint	Non-Carcinogen	Non-Carcinogen	Non-Carcinogen
Predicted Endpoint	Non-Carcinogen	Non-Carcinogen	Non-Carcinogen
Distance	1.501	1.509	1.647
Reference	US FDA (Centre for Drug Eval.& Res./Off. Testing & Res.) Sept. 1997	US FDA (Centre for Drug Eval.& Res./Off. Testing & Res.) Sept. 1997	US FDA (Centre for Drug Eval.& Res./Off. Testing & Res.) Sept. 1997

Model Applicability

Unknown features are fingerprint features in the query molecule, but not found in the training set.

1. Num_H_Donors out of range. Value: 10. Training min, max, mean, SD: 0, 8, 1.7342, 1.449.
2. Num_H_Acceptors out of range. Value: 20. Training min, max, mean, SD: 0, 15, 4.2627, 2.435.
3. OPS PC6 out of range. Value: 5.7174. Training min, max, SD, explained variance: -5.6153, 5.6497, 1.991, 0.0416.

Feature Contribution

Top features for positive contribution

Fingerprint	Bit/Smiles	Feature Structure	Score	Carcinogen in training set
FCFP_6	-146015125	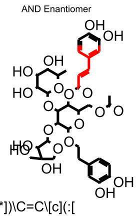 [*]C(=[*])\C=C\[c](:[cH]:[*]):[cH]:[*]	0.676	2 out of 2

FCFP_6	451847724	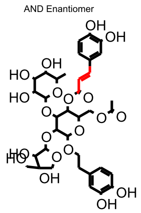 [*]\C=C\C(=[*])[*]	0.479	21 out of 48
FCFP_6	-2132692875	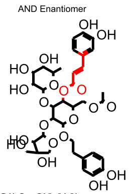 [*]OC(=O)\C=C\[c](:[*]):[*]	0.460	1 out of 1

Top Features for negative contribution

Fingerprint	Bit/Smiles	Feature Structure	Score	Carcinogen in training set
FCFP_6	-497728148	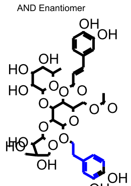 [*]CC[c]1:[cH]:[cH]:[*]:[c]([*]):[cH]:1	-0.960	2 out of 26
FCFP_6	699500266	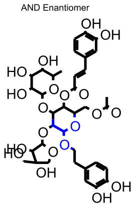 [*]OC(O[*])C([*])[*]	-0.850	1 out of 14
FCFP_6	-2005207466	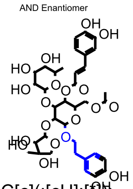 [*]OCC[c](:[cH]:[*]):[cH]:[*]	-0.582	0 out of 3

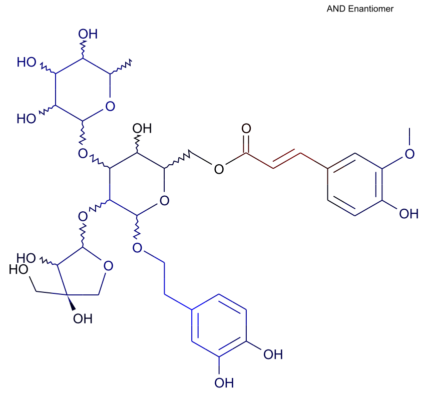

$C_{35}H_{46}O_{19}$

Molecular Weight: 770.72833

ALogP: -0.699

Rotatable Bonds: 15

Acceptors: 19

Donors: 10

Model Prediction

Prediction: Non-Carcinogen

Probability: 0.105

Enrichment: 0.358

Bayesian Score: -13.549

Mahalanobis Distance: 16.254

Mahalanobis Distance p-value: 2.64e-010

Prediction: Positive if the Bayesian score is above the estimated best cutoff value from minimizing the false positive and false negative rate.
Probability: The esimated probability that the sample is in the positive category. This assumes that the Bayesian score follows a normal distribution and is different from the prediction using a cutoff.
Enrichment: An estimate of enrichment, that is, the increased likelihood (versus random) of this sample being in the category.
Bayesian Score: The standard Laplacian-modified Bayesian score.
Mahalanobis Distance: The Mahalanobis distance (MD) is the distance to the center of the training data. The larger the MD, the less trustworthy the prediction.
Mahalanobis Distance p-value: The p-value gives the fraction of training data with an MD greater than or equal to the one for the given sample, assuming normally distributed data. The smaller the p-value, the less trustworthy the prediciton. For highly non-normal X properties (e.g., fingerprints), the MD p-value is wildly inaccurate.

Structural Similar Compounds

Name	Netilmicin	Rifampin	Rifabutin
Structure	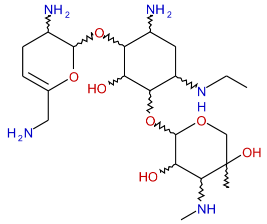	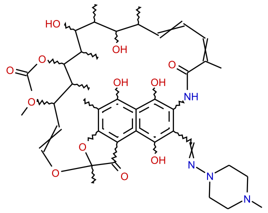	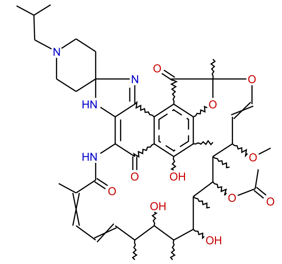
Actual Endpoint	Non-Carcinogen	Non-Carcinogen	Non-Carcinogen
Predicted Endpoint	Non-Carcinogen	Non-Carcinogen	Non-Carcinogen
Distance	1.402	1.437	1.586
Reference	US FDA (Centre for Drug Eval.& Res./Off. Testing & Res.) Sept. 1997	US FDA (Centre for Drug Eval.& Res./Off. Testing & Res.) Sept. 1997	US FDA (Centre for Drug Eval.& Res./Off. Testing & Res.) Sept. 1997

Model Applicability

Unknown features are fingerprint features in the query molecule, but not found in the training set.

1. Num_H_Donors out of range. Value: 10. Training min, max, mean, SD: 0, 8, 1.7342, 1.449.
2. Num_H_Acceptors out of range. Value: 19. Training min, max, mean, SD: 0, 15, 4.2627, 2.435.
3. OPS PC3 out of range. Value: 6.2187. Training min, max, SD, explained variance: -6.8858, 5.9444, 2.309, 0.0559.

Feature Contribution

Top features for positive contribution

Fingerprint	Bit/Smiles	Feature Structure	Score	Carcinogen in training set
FCFP_6	-146015125	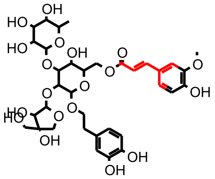 [*]C(=[*])\C=C\[c](:[cH]:[*]):[cH]:[*]	0.676	2 out of 2

FCFP_6	451847724	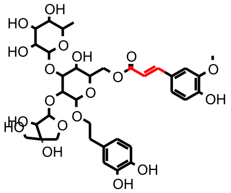 [*]\C=C\C(=[*])[*]	0.479	21 out of 48
FCFP_6	-1251367201	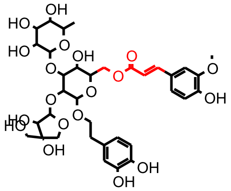 [*]\C=C\C(=O)OC([*])[*]	0.460	1 out of 1
**Top Features for negative contribution**				
**Fingerprint**	**Bit/Smiles**	**Feature Structure**	**Score**	**Carcinogen in training set**
FCFP_6	-497728148	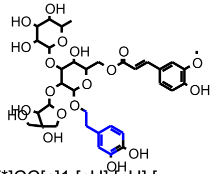 [*]CC[c]1:[cH]:[cH]:[*]:[c]([*]):[cH]:1	-0.960	2 out of 26
FCFP_6	699500266	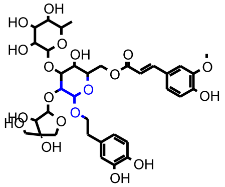 [*]OC(O[*])C([*])[*]	-0.850	1 out of 14
FCFP_6	745786291	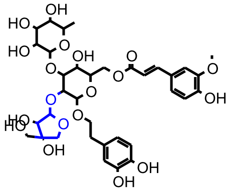 [*]OC1OCC([*])([*])C1 [*]	-0.582	0 out of 3

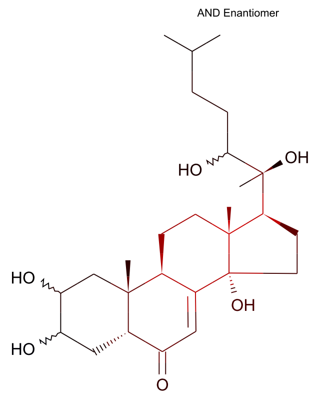

$C_{27}H_{44}O_6$
Molecular Weight: 464.63466
ALogP: 2.489
Rotatable Bonds: 5
Acceptors: 6
Donors: 5

Structural Similar Compounds

Name	Triamcinolone	Budesonide	Glimepride
Structure	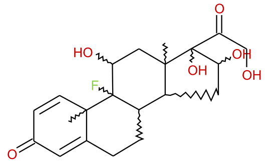	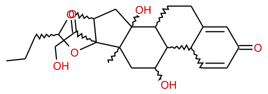	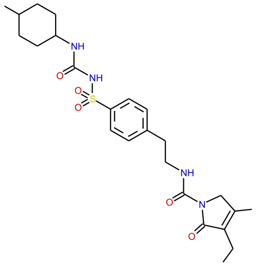
Actual Endpoint	Carcinogen	Non-Carcinogen	Carcinogen
Predicted Endpoint	Carcinogen	Non-Carcinogen	Carcinogen
Distance	0.721	0.761	0.793
Reference	US FDA (Centre for Drug Eval.& Res./Off. Testing & Res.) Sept. 1997	US FDA (Centre for Drug Eval.& Res./Off. Testing & Res.) Sept. 1997	US FDA (Centre for Drug Eval.& Res./Off. Testing & Res.) Sept. 1997

Model Applicability

Unknown features are fingerprint features in the query molecule, but not found in the training set.

1. All properties and OPS components are within expected ranges.

Feature Contribution

Top features for positive contribution

Fingerprint	Bit/Smiles	Feature Structure	Score	Carcinogen in training set
FCFP_6	-1742546106	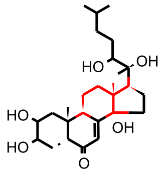 [*][C@@H]1[*][C@]2([*])[*][*][C@H]([*])[C@@]2(C)CC1	0.748	10 out of 16

Model Prediction

Prediction: Carcinogen
Probability: 0.457
Enrichment: 1.551
Bayesian Score: 4.542
Mahalanobis Distance: 10.822
Mahalanobis Distance p-value: 0.107

Prediction: Positive if the Bayesian score is above the estimated best cutoff value from minimizing the false positive and false negative rate.
Probability: The esimated probability that the sample is in the positive category. This assumes that the Bayesian score follows a normal distribution and is different from the prediction using a cutoff.
Enrichment: An estimate of enrichment, that is, the increased likelihood (versus random) of this sample being in the category.
Bayesian Score: The standard Laplacian-modified Bayesian score.
Mahalanobis Distance: The Mahalanobis distance (MD) is the distance to the center of the training data. The larger the MD, the less trustworthy the prediction.
Mahalanobis Distance p-value: The p-value gives the fraction of training data with an MD greater than or equal to the one for the given sample, assuming normally distributed data. The smaller the p-value, the less trustworthy the prediciton. For highly non-normal X properties (e.g., fingerprints), the MD p-value is wildly inaccurate.

FCFP_6	-415245925	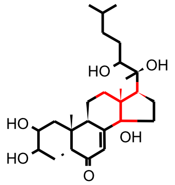 [*]C[C@]1(C)[C@@H]([*])[*][*]C1([*])[*]	0.710	16 out of 28
FCFP_6	-145742489	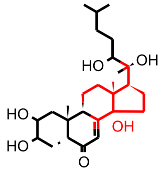 [*]C([*])([*])[C@H]1C C[C@@]2(O)C(=[*])[*] CC[C@]12C	0.668	3 out of 4
**Top Features for negative contribution**				
**Fingerprint**	**Bit/Smiles**	**Feature Structure**	**Score**	**Carcinogen in training set**
FCFP_6	420514984	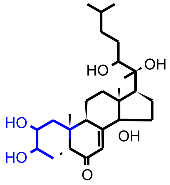 [*]C1([*])[*]CC(O)C(O)C1	-0.233	0 out of 1
FCFP_6	-415156552	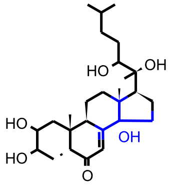 [*]C[C@@]1(O)C[*][*]C 1[*]	-0.217	7 out of 34
FCFP_6	-1043250487	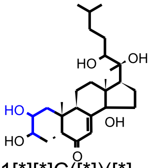 [*]C1[*][*]C([*])([*])C1O	-0.073	19 out of 78

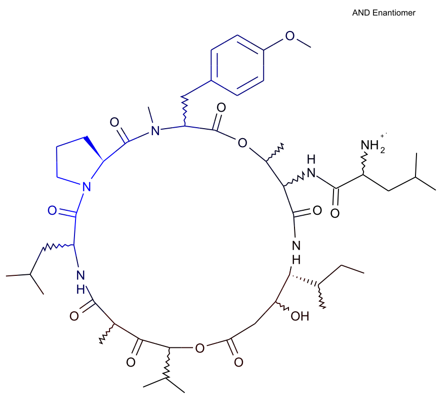

$C_{49}H_{79}N_6O_{12}$
Molecular Weight: 944.18455
ALogP: 3.176
Rotatable Bonds: 13
Acceptors: 12
Donors: 5

Model Prediction

Prediction: Non-Carcinogen
Probability: 0.121
Enrichment: 0.411
Bayesian Score: -12.039
Mahalanobis Distance: 15.647
Mahalanobis Distance p-value: 4.82e-009

Prediction: Positive if the Bayesian score is above the estimated best cutoff value from minimizing the false positive and false negative rate.
Probability: The esimated probability that the sample is in the positive category. This assumes that the Bayesian score follows a normal distribution and is different from the prediction using a cutoff.
Enrichment: An estimate of enrichment, that is, the increased likelihood (versus random) of this sample being in the category.
Bayesian Score: The standard Laplacian-modified Bayesian score.
Mahalanobis Distance: The Mahalanobis distance (MD) is the distance to the center of the training data. The larger the MD, the less trustworthy the prediction.
Mahalanobis Distance p-value: The p-value gives the fraction of training data with an MD greater than or equal to the one for the given sample, assuming normally distributed data. The smaller the p-value, the less trustworthy the prediciton. For highly non-normal X properties (e.g., fingerprints), the MD p-value is wildly inaccurate.

Structural Similar Compounds

Name	Erythromycin	Rifabutin	Rifampin
Structure	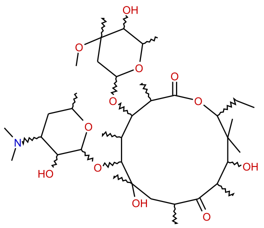	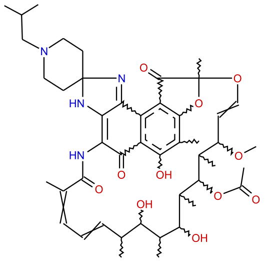	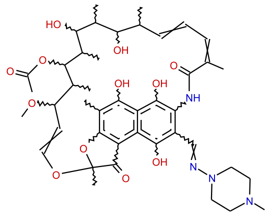
Actual Endpoint	Non-Carcinogen	Non-Carcinogen	Non-Carcinogen
Predicted Endpoint	Non-Carcinogen	Non-Carcinogen	Non-Carcinogen
Distance	0.996	0.996	1.057
Reference	US FDA (Centre for Drug Eval.& Res./Off. Testing & Res.) Sept. 1997	US FDA (Centre for Drug Eval.& Res./Off. Testing & Res.) Sept. 1997	US FDA (Centre for Drug Eval.& Res./Off. Testing & Res.) Sept. 1997

Model Applicability

Unknown features are fingerprint features in the query molecule, but not found in the training set.

1. Molecular_Weight out of range. Value: 944.18. Training min, max, mean, SD: 46.068, 847, 304.63, 118.9.
2. Unknown FCFP_2 feature: 10: [*][NH2+][*]
3. Unknown FCFP_2 feature: 18028531: [*]C([*])[NH2+]C
4. Unknown FCFP_2 feature: -1817836174: [*]CC([NH2+][*])C(=[*])[*]
5. Unknown FCFP_2 feature: 136418580: [*][NH2+]C

Feature Contribution

Top features for positive contribution

Fingerprint	Bit/Smiles	Feature Structure	Score	Carcinogen in training set
FCFP_6	261084607	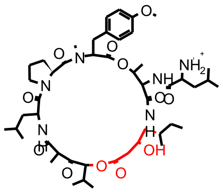 [*]OC(=O)CC(O)C([*])[*]	0.676	2 out of 2

FCFP_6	61186801	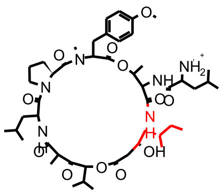<br>[*]N[C@@H](C([*])[*])<br>C(C)CC	0.460	1 out of 1
FCFP_6	566058135	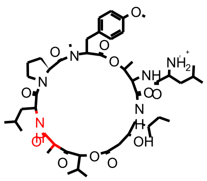<br>[*]NC(=O)C([*])[*]	0.447	17 out of 40
**Top Features for negative contribution**				
**Fingerprint**	**Bit/Smiles**	**Feature Structure**	**Score**	**Carcinogen in training set**
FCFP_6	393220684	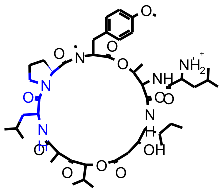<br>[*]CC(N[*])C(=O)N1C[*<br>][*][C@H]1[*]	-1.133	0 out of 8
FCFP_6	-497728148	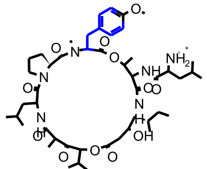<br>[*]CC[c]1:[cH]:[cH]:[<br>*]:[c]([*]):[cH]:1	-0.960	2 out of 26
FCFP_6	-1423737326	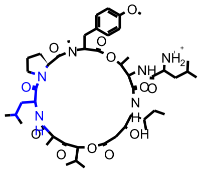<br>[*]C([*])CC(NC(=[*])[<br>*])C(=O)N([*])[*]	-0.947	0 out of 6

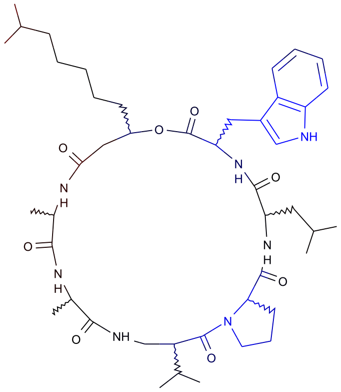

$C_{45}H_{69}N_7O_8$
Molecular Weight: 836.07145
ALogP: 4.986
Rotatable Bonds: 11
Acceptors: 8
Donors: 6

Model Prediction

Prediction: Non-Carcinogen
Probability: 0.098
Enrichment: 0.331
Bayesian Score: -14.368
Mahalanobis Distance: 15.128
Mahalanobis Distance p-value: 5.24e-008

Prediction: Positive if the Bayesian score is above the estimated best cutoff value from minimizing the false positive and false negative rate.
Probability: The esimated probability that the sample is in the positive category. This assumes that the Bayesian score follows a normal distribution and is different from the prediction using a cutoff.
Enrichment: An estimate of enrichment, that is, the increased likelihood (versus random) of this sample being in the category.
Bayesian Score: The standard Laplacian-modified Bayesian score.
Mahalanobis Distance: The Mahalanobis distance (MD) is the distance to the center of the training data. The larger the MD, the less trustworthy the prediction.
Mahalanobis Distance p-value: The p-value gives the fraction of training data with an MD greater than or equal to the one for the given sample, assuming normally distributed data. The smaller the p-value, the less trustworthy the prediciton. For highly non-normal X properties (e.g., fingerprints), the MD p-value is wildly inaccurate.

Structural Similar Compounds

Name	Bromocriptine	Erythromycin	Rifabutin
Structure	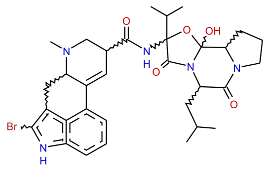	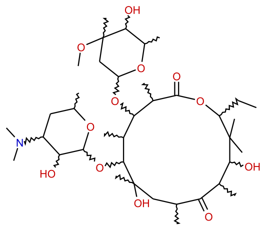	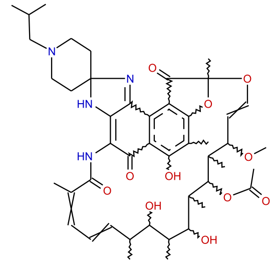
Actual Endpoint	Non-Carcinogen	Non-Carcinogen	Non-Carcinogen
Predicted Endpoint	Non-Carcinogen	Non-Carcinogen	Non-Carcinogen
Distance	1.067	1.082	1.106
Reference	US FDA (Centre for Drug Eval.& Res./Off. Testing & Res.) Sept. 1997	US FDA (Centre for Drug Eval.& Res./Off. Testing & Res.) Sept. 1997	US FDA (Centre for Drug Eval.& Res./Off. Testing & Res.) Sept. 1997

Model Applicability

Unknown features are fingerprint features in the query molecule, but not found in the training set.

1. All properties and OPS components are within expected ranges.

Feature Contribution

Top features for positive contribution

Fingerprint	Bit/Smiles	Feature Structure	Score	Carcinogen in training set
FCFP_6	-55265897	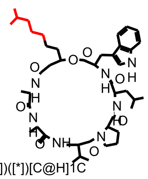 [*]C([*])([*])[C@H]1C CC([*])([*])[*]C1=[*]	0.594	17 out of 34

FCFP_6	-1367722585	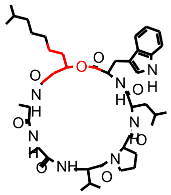 [*]CCC(CC(=[*])[*])OC(=[*])[*]	0.547	3 out of 5
FCFP_6	-387072142	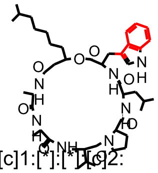 [*][c]1:[*]:[*]:[c]2:[cH]:[cH]:[cH]:[cH]:[c]:1:2	0.477	4 out of 8

Top Features for negative contribution

Fingerprint	Bit/Smiles	Feature Structure	Score	Carcinogen in training set
FCFP_6	-1525101452	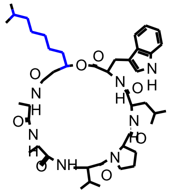 [*]C([*])CCCCCC([*])[*]	-1.133	0 out of 8
FCFP_6	392412010	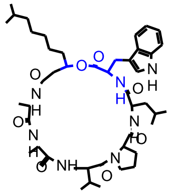 [*]CC(N[*])C(=O)OC([*])[*]	-0.947	0 out of 6
FCFP_6	852937099	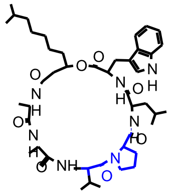 [*]C([*])C(=O)N1CCC[C@H]1C(=[*])[*]	-0.839	0 out of 5

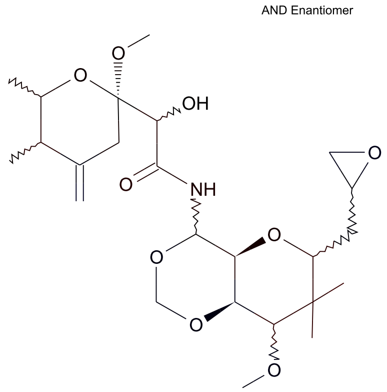

$C_{24}H_{39}NO_9$
Molecular Weight: 485.56776
ALogP: 0.267
Rotatable Bonds: 7
Acceptors: 9
Donors: 2

Structural Similar Compounds

Name	Bacampicillin	Budesonide	Amlodipine
Structure	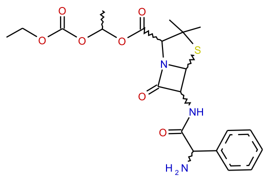	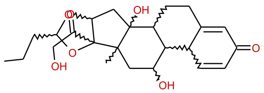	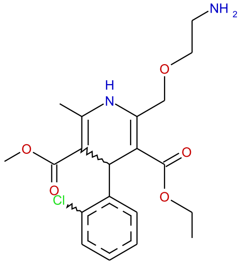
Actual Endpoint	Non-Carcinogen	Non-Carcinogen	Non-Carcinogen
Predicted Endpoint	Non-Carcinogen	Non-Carcinogen	Non-Carcinogen
Distance	0.706	0.754	0.756
Reference	US FDA (Centre for Drug Eval.& Res./Off. Testing & Res.) Sept. 1997	US FDA (Centre for Drug Eval.& Res./Off. Testing & Res.) Sept. 1997	US FDA (Centre for Drug Eval.& Res./Off. Testing & Res.) Sept. 1997

Model Prediction

Prediction: Carcinogen
Probability: 0.283
Enrichment: 0.963
Bayesian Score: -0.860
Mahalanobis Distance: 12.892
Mahalanobis Distance p-value: 0.000406

Prediction: Positive if the Bayesian score is above the estimated best cutoff value from minimizing the false positive and false negative rate.
Probability: The esimated probability that the sample is in the positive category. This assumes that the Bayesian score follows a normal distribution and is different from the prediction using a cutoff.
Enrichment: An estimate of enrichment, that is, the increased likelihood (versus random) of this sample being in the category.
Bayesian Score: The standard Laplacian-modified Bayesian score.
Mahalanobis Distance: The Mahalanobis distance (MD) is the distance to the center of the training data. The larger the MD, the less trustworthy the prediction.
Mahalanobis Distance p-value: The p-value gives the fraction of training data with an MD greater than or equal to the one for the given sample, assuming normally distributed data. The smaller the p-value, the less trustworthy the prediciton. For highly non-normal X properties (e.g., fingerprints), the MD p-value is wildly inaccurate.

Model Applicability

Unknown features are fingerprint features in the query molecule, but not found in the training set.

1. All properties and OPS components are within expected ranges.

Feature Contribution

Top features for positive contribution

Fingerprint	Bit/Smiles	Feature Structure	Score	Carcinogen in training set
FCFP_6	-415245925	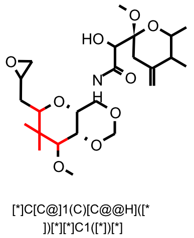	0.710	16 out of 28

FCFP_6	566058135	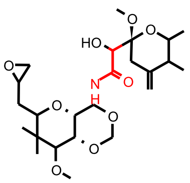<br>[*]NC(=O)C([*])[*]	0.447	17 out of 40
FCFP_6	-1043339860	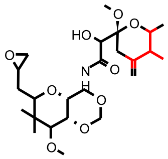<br>[*]C([*])([*])[C@H]1C<br>[*][*]C1([*])[*]	0.383	24 out of 61

Top Features for negative contribution

Fingerprint	Bit/Smiles	Feature Structure	Score	Carcinogen in training set
FCFP_6	129344189	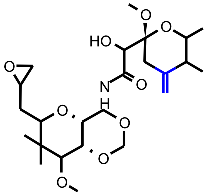<br>[*]C(=C)[*]	-0.719	0 out of 4
FCFP_6	470041467	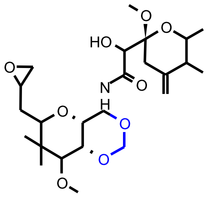<br>[*]OCO[*]	-0.582	0 out of 3
FCFP_6	-1043310069	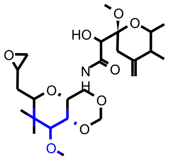<br>[*]OC(C([*])[*])C([*]<br>)[*]	-0.406	10 out of 59

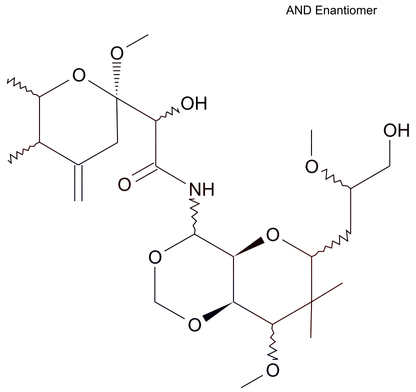

$C_{25}H_{43}NO_{10}$
Molecular Weight: 517.60962
ALogP: -9.1e-002
Rotatable Bonds: 9
Acceptors: 10
Donors: 3

Model Prediction

Prediction: Carcinogen
Probability: 0.273
Enrichment: 0.927
Bayesian Score: -1.285
Mahalanobis Distance: 12.566
Mahalanobis Distance p-value: 0.0012

Prediction: Positive if the Bayesian score is above the estimated best cutoff value from minimizing the false positive and false negative rate.
Probability: The esimated probability that the sample is in the positive category. This assumes that the Bayesian score follows a normal distribution and is different from the prediction using a cutoff.
Enrichment: An estimate of enrichment, that is, the increased likelihood (versus random) of this sample being in the category.
Bayesian Score: The standard Laplacian-modified Bayesian score.
Mahalanobis Distance: The Mahalanobis distance (MD) is the distance to the center of the training data. The larger the MD, the less trustworthy the prediction.
Mahalanobis Distance p-value: The p-value gives the fraction of training data with an MD greater than or equal to the one for the given sample, assuming normally distributed data. The smaller the p-value, the less trustworthy the prediciton. For highly non-normal X properties (e.g., fingerprints), the MD p-value is wildly inaccurate.

Structural Similar Compounds

Name	Bacampicillin	Amlodipine	Dipyridamole
Structure	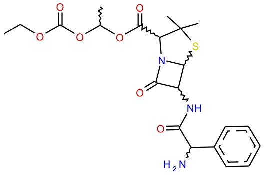	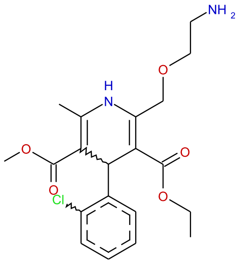	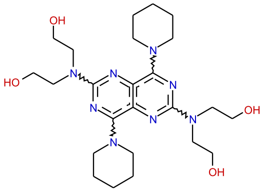
Actual Endpoint	Non-Carcinogen	Non-Carcinogen	Non-Carcinogen
Predicted Endpoint	Non-Carcinogen	Non-Carcinogen	Non-Carcinogen
Distance	0.728	0.832	0.844
Reference	US FDA (Centre for Drug Eval.& Res./Off. Testing & Res.) Sept. 1997	US FDA (Centre for Drug Eval.& Res./Off. Testing & Res.) Sept. 1997	US FDA (Centre for Drug Eval.& Res./Off. Testing & Res.) Sept. 1997

Model Applicability

Unknown features are fingerprint features in the query molecule, but not found in the training set.

1. All properties and OPS components are within expected ranges.

Feature Contribution

Top features for positive contribution

Fingerprint	Bit/Smiles	Feature Structure	Score	Carcinogen in training set
FCFP_6	-415245925	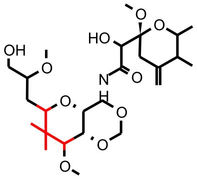 [*]C[C@]1(C)[C@@H]([*])[*][*]C1([*])[*]	0.710	16 out of 28

FCFP_6	566058135	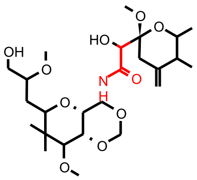 [*]NC(=O)C([*])[*]	0.447	17 out of 40
FCFP_6	-1043339860	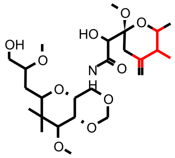 [*]C([*])([*])[C@H]1C [*][*]C1([*])[*]	0.383	24 out of 61

Top Features for negative contribution

Fingerprint	Bit/Smiles	Feature Structure	Score	Carcinogen in training set
FCFP_6	129344189	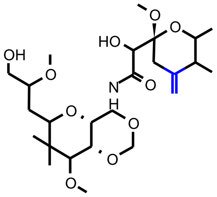 [*]C(=C)[*]	-0.719	0 out of 4
FCFP_6	470041467	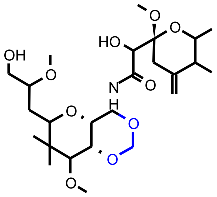 [*]OCO[*]	-0.582	0 out of 3
FCFP_6	-1043310069	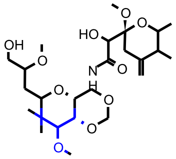 [*]OC(C([*])[*])C([*])[*]	-0.406	10 out of 59

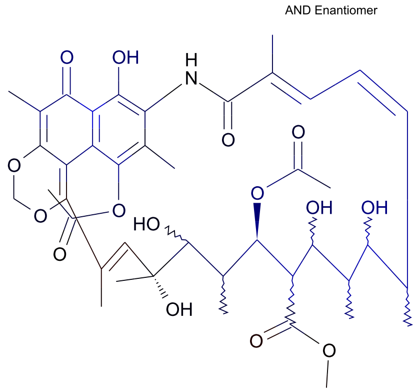

$C_{42}H_{53}NO_{15}$
Molecular Weight: 811.86791
ALogP: 2.745
Rotatable Bonds: 6
Acceptors: 15
Donors: 6

Model Prediction

Prediction: Non-Carcinogen
Probability: 0.123
Enrichment: 0.418
Bayesian Score: -11.857
Mahalanobis Distance: 13.906
Mahalanobis Distance p-value: 9.48e-006

Prediction: Positive if the Bayesian score is above the estimated best cutoff value from minimizing the false positive and false negative rate.
Probability: The esimated probability that the sample is in the positive category. This assumes that the Bayesian score follows a normal distribution and is different from the prediction using a cutoff.
Enrichment: An estimate of enrichment, that is, the increased likelihood (versus random) of this sample being in the category.
Bayesian Score: The standard Laplacian-modified Bayesian score.
Mahalanobis Distance: The Mahalanobis distance (MD) is the distance to the center of the training data. The larger the MD, the less trustworthy the prediction.
Mahalanobis Distance p-value: The p-value gives the fraction of training data with an MD greater than or equal to the one for the given sample, assuming normally distributed data. The smaller the p-value, the less trustworthy the prediciton. For highly non-normal X properties (e.g., fingerprints), the MD p-value is wildly inaccurate.

Structural Similar Compounds

Name	Rifampin	Rifabutin	Erythromycin
Structure	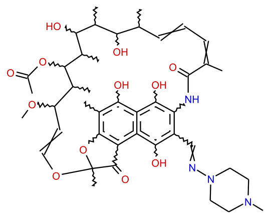	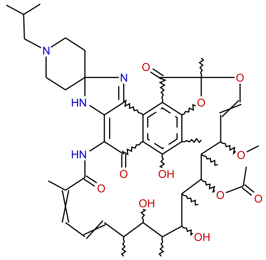	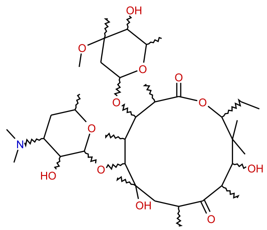
Actual Endpoint	Non-Carcinogen	Non-Carcinogen	Non-Carcinogen
Predicted Endpoint	Non-Carcinogen	Non-Carcinogen	Non-Carcinogen
Distance	0.431	0.594	0.827
Reference	US FDA (Centre for Drug Eval.& Res./Off. Testing & Res.) Sept. 1997	US FDA (Centre for Drug Eval.& Res./Off. Testing & Res.) Sept. 1997	US FDA (Centre for Drug Eval.& Res./Off. Testing & Res.) Sept. 1997

Model Applicability

Unknown features are fingerprint features in the query molecule, but not found in the training set.

1. All properties and OPS components are within expected ranges.

Feature Contribution

Top features for positive contribution

Fingerprint	Bit/Smiles	Feature Structure	Score	Carcinogen in training set
FCFP_6	1175665944	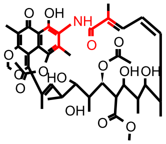 [*]C(=[*])C(=O)N[c](:[c]([*]):[*]):[c]([*]):[*]	0.655	7 out of 12

FCFP_6	451847724	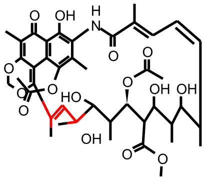 [*]\C=C\C(=[*])[*]	0.479	21 out of 48
FCFP_6	566058135	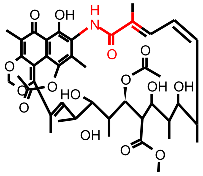 [*]NC(=O)C([*])[*]	0.447	17 out of 40
**Top Features for negative contribution**				
**Fingerprint**	**Bit/Smiles**	**Feature Structure**	**Score**	**Carcinogen in training set**
FCFP_6	-1096767684	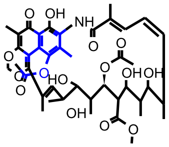 [*]C(=[*])O[c]1:[c](C):[c]([*]):[*]:[c]([*]):[c]:1C(=[*])[*]	-0.947	0 out of 6
FCFP_6	449814859	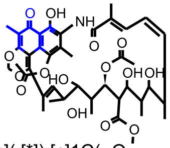 [*][c](:[*]):[c]1C(=O)C(=C([*])[*][c]:1:[*])C	-0.947	0 out of 6
FCFP_6	524083277	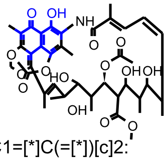 [*]C1=[*]C(=[*])[c]2: [c]([*]):[*]:[c]([*]):[c](O):[c]:2C1=O	-0.839	0 out of 5

Simeprevir

TOPKAT_Mouse_Male_FDA_None_vs_Carcinogen

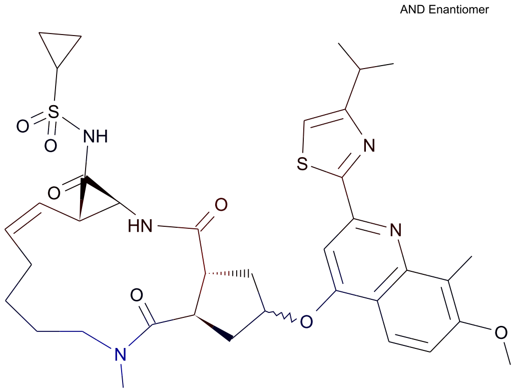

$C_{38}H_{47}N_5O_7S_2$

Molecular Weight: 749.93907

ALogP: 4.796

Rotatable Bonds: 8

Acceptors: 9

Donors: 2

Structural Similar Compounds

Name	Reserpine	Bromocriptine	Itraconazole
Structure	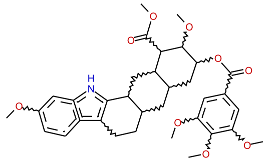	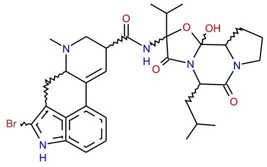	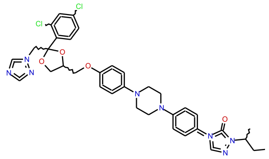
Actual Endpoint	Carcinogen	Non-Carcinogen	Non-Carcinogen
Predicted Endpoint	Carcinogen	Non-Carcinogen	Non-Carcinogen
Distance	0.775	0.832	0.865
Reference	US FDA (Centre for Drug Eval.& Res./Off. Testing & Res.) Sept. 1997	US FDA (Centre for Drug Eval.& Res./Off. Testing & Res.) Sept. 1997	US FDA (Centre for Drug Eval.& Res./Off. Testing & Res.) Sept. 1997

Model Prediction

Prediction: Non-Carcinogen

Probability: 0.258

Enrichment: 0.877

Bayesian Score: -1.921

Mahalanobis Distance: 18.337

Mahalanobis Distance p-value: 6.22e-015

Prediction: Positive if the Bayesian score is above the estimated best cutoff value from minimizing the false positive and false negative rate.
Probability: The esimated probability that the sample is in the positive category. This assumes that the Bayesian score follows a normal distribution and is different from the prediction using a cutoff.
Enrichment: An estimate of enrichment, that is, the increased likelihood (versus random) of this sample being in the category.
Bayesian Score: The standard Laplacian-modified Bayesian score.
Mahalanobis Distance: The Mahalanobis distance (MD) is the distance to the center of the training data. The larger the MD, the less trustworthy the prediction.
Mahalanobis Distance p-value: The p-value gives the fraction of training data with an MD greater than or equal to the one for the given sample, assuming normally distributed data. The smaller the p-value, the less trustworthy the prediciton. For highly non-normal X properties (e.g., fingerprints), the MD p-value is wildly inaccurate.

Model Applicability

Unknown features are fingerprint features in the query molecule, but not found in the training set.

1. All properties and OPS components are within expected ranges.
2. Unknown FCFP_2 feature: 690481386: [*]:[c](:[*])[c]1:n:[*]:[*]:s:1

Feature Contribution

Top features for positive contribution

Fingerprint	Bit/Smiles	Feature Structure	Score	Carcinogen in training set
FCFP_6	-55265897	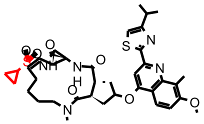 [*]C([*])([*])[C@H]1CCC([*])([*])[*]C1=[*]	0.594	17 out of 34

FCFP_6	-1289661876	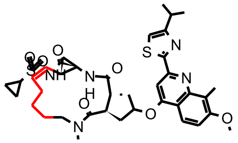 [*]CCC\C=C/[*]	0.517	2 out of 3
FCFP_6	451847724	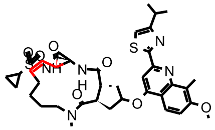 [*]\C=C\C(=[*])[*]	0.479	21 out of 48

Top Features for negative contribution

Fingerprint	Bit/Smiles	Feature Structure	Score	Carcinogen in training set
FCFP_6	-98332825	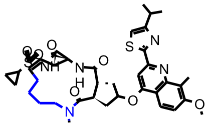 [*][C@@H]1CCCN1[*]	-0.793	1 out of 13
FCFP_6	-1972798083	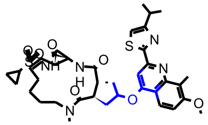 [*]:[cH]:[c](OC1C[*][*]C1):[c](:[*]):[*]	-0.582	0 out of 3
FCFP_6	-1553874037	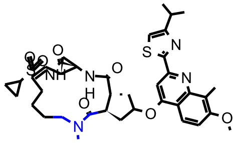 [*][C@@H]1[*][*]CN1C(=[*])[*]	-0.450	5 out of 32

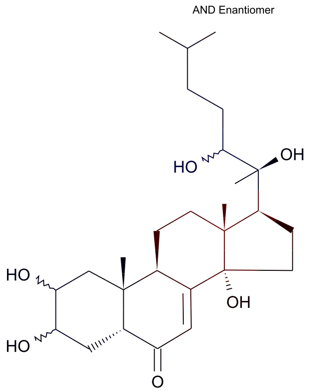

$C_{27}H_{44}O_6$
Molecular Weight: 464.63466
ALogP: 2.489
Rotatable Bonds: 5
Acceptors: 6
Donors: 5

Structural Similar Compounds

Name	Triamcinolone	Glimepride	Sulfasalazine
Structure	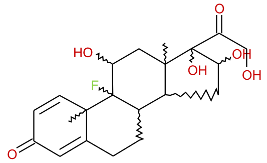	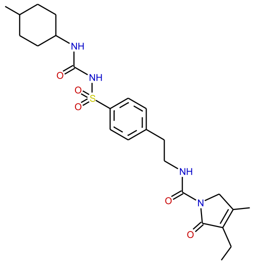	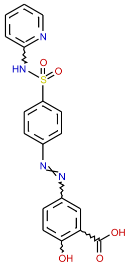
Actual Endpoint	Single-Carcinogen	Single-Carcinogen	Single-Carcinogen
Predicted Endpoint	Single-Carcinogen	Single-Carcinogen	Single-Carcinogen
Distance	0.787	0.898	0.987
Reference	US FDA (Centre for Drug Eval.& Res./Off. Testing & Res.) Sept. 1997	US FDA (Centre for Drug Eval.& Res./Off. Testing & Res.) Sept. 1997	US FDA (Centre for Drug Eval.& Res./Off. Testing & Res.) Sept. 1997

Model Prediction

Prediction: Multiple-Carcinogen
Probability: 0.242
Enrichment: 0.804
Bayesian Score: -0.642
Mahalanobis Distance: 9.663
Mahalanobis Distance p-value: 0.0309

Prediction: Positive if the Bayesian score is above the estimated best cutoff value from minimizing the false positive and false negative rate.
Probability: The esimated probability that the sample is in the positive category. This assumes that the Bayesian score follows a normal distribution and is different from the prediction using a cutoff.
Enrichment: An estimate of enrichment, that is, the increased likelihood (versus random) of this sample being in the category.
Bayesian Score: The standard Laplacian-modified Bayesian score.
Mahalanobis Distance: The Mahalanobis distance (MD) is the distance to the center of the training data. The larger the MD, the less trustworthy the prediction.
Mahalanobis Distance p-value: The p-value gives the fraction of training data with an MD greater than or equal to the one for the given sample, assuming normally distributed data. The smaller the p-value, the less trustworthy the prediciton. For highly non-normal X properties (e.g., fingerprints), the MD p-value is wildly inaccurate.

Model Applicability

Unknown features are fingerprint features in the query molecule, but not found in the training set.

1. All properties and OPS components are within expected ranges.

Feature Contribution

Top features for positive contribution				
**Fingerprint**	**Bit/Smiles**	**Feature Structure**	**Score**	**Multiple-Carcinogen in training set**
FCFP_12	-1742546106	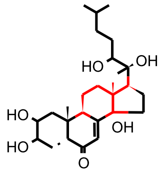 [*][C@@H]1[*][C@]2([*])[*][*][C@H]([*])[C@@]2(C)CC1	0.597	7 out of 10

FCFP_12	-415245925	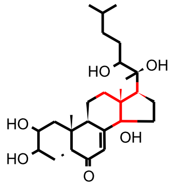 [*]C[C@]1(C)[C@@H]([*])[*][*]C1([*])[*]	0.535	10 out of 16
FCFP_12	-1870530637	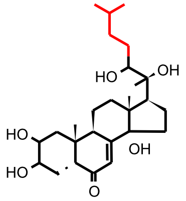 [*]CCC(C)C	0.400	1 out of 1

Top Features for negative contribution

Fingerprint	Bit/Smiles	Feature Structure	Score	Multiple-Carcinogen in training set
FCFP_12	354117335	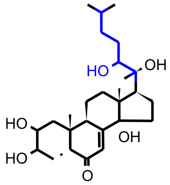 [*]C([*])CCC(O)C([*])([*])[*]	-0.859	0 out of 4
FCFP_12	351491212	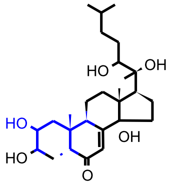 [*]C([*])[C@@]1(C)CC(O)C([*])[*][C@H]1[*]	-0.519	0 out of 2
FCFP_12	1970621279	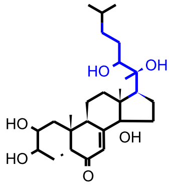 [*]CCC(O)[C@](C)(O)C([*])[*]	-0.293	0 out of 1

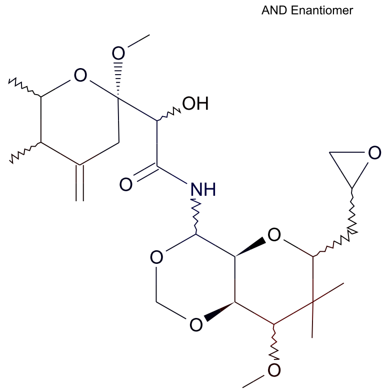

$C_{24}H_{39}NO_9$
Molecular Weight: 485.56776
ALogP: 0.267
Rotatable Bonds: 7
Acceptors: 9
Donors: 2

Structural Similar Compounds

Name	Flunisolide	Sulfasalazine	Dihydroxymethylfuratrizine
Structure	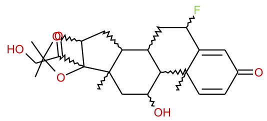	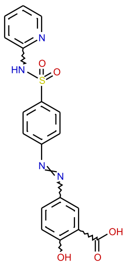	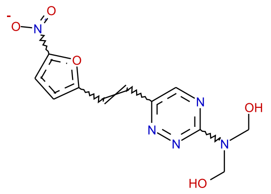
Actual Endpoint	Single-Carcinogen	Single-Carcinogen	Multiple-Carcinogen
Predicted Endpoint	Single-Carcinogen	Single-Carcinogen	Multiple-Carcinogen
Distance	0.918	0.928	0.958
Reference	US FDA (Centre for Drug Eval.& Res./Off. Testing & Res.) Sept. 1997	US FDA (Centre for Drug Eval.& Res./Off. Testing & Res.) Sept. 1997	US FDA (Centre for Drug Eval.& Res./Off. Testing & Res.) Sept. 1997

Model Prediction

Prediction: Multiple-Carcinogen
Probability: 0.234
Enrichment: 0.778
Bayesian Score: -0.949
Mahalanobis Distance: 13.752
Mahalanobis Distance p-value: 2.48e-005

Prediction: Positive if the Bayesian score is above the estimated best cutoff value from minimizing the false positive and false negative rate.
Probability: The esimated probability that the sample is in the positive category. This assumes that the Bayesian score follows a normal distribution and is different from the prediction using a cutoff.
Enrichment: An estimate of enrichment, that is, the increased likelihood (versus random) of this sample being in the category.
Bayesian Score: The standard Laplacian-modified Bayesian score.
Mahalanobis Distance: The Mahalanobis distance (MD) is the distance to the center of the training data. The larger the MD, the less trustworthy the prediction.
Mahalanobis Distance p-value: The p-value gives the fraction of training data with an MD greater than or equal to the one for the given sample, assuming normally distributed data. The smaller the p-value, the less trustworthy the prediciton. For highly non-normal X properties (e.g., fingerprints), the MD p-value is wildly inaccurate.

Model Applicability

Unknown features are fingerprint features in the query molecule, but not found in the training set.

1. All properties and OPS components are within expected ranges.
2. Unknown FCFP_2 feature: 470041467: [*]OCO[*]
3. Unknown FCFP_2 feature: 129344189: [*]C(=C)[*]

Feature Contribution

Top features for positive contribution

Fingerprint	Bit/Smiles	Feature Structure	Score	Multiple-Carcinogen in training set
FCFP_12	-1043310069	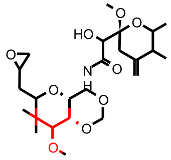 [*]OC(C([*])[*])C([*])([*])[*]	0.597	7 out of 10

FCFP_12	-415245925	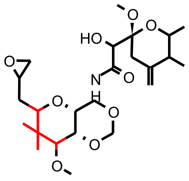 [*]C[C@]1(C)[C@@H]([*])[*][*]C1([*])[*]	0.535	10 out of 16
FCFP_12	346066116	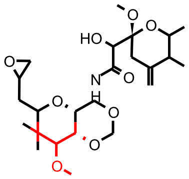 [*]C([*])C(OC)C([*])([*])[*]	0.400	1 out of 1
**Top Features for negative contribution**				
**Fingerprint**	**Bit/Smiles**	**Feature Structure**	**Score**	**Multiple-Carcinogen in training set**
FCFP_12	566058135	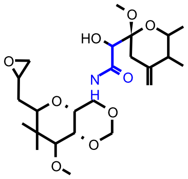 [*]NC(=O)C([*])[*]	-0.528	3 out of 17
FCFP_12	699559848	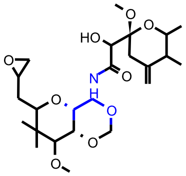 [*]NC(O[*])C([*])[*]	-0.519	0 out of 2
FCFP_12	-1272768868	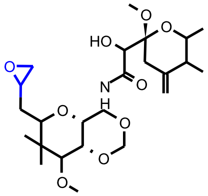 [*]C1CO1	-0.347	5 out of 22

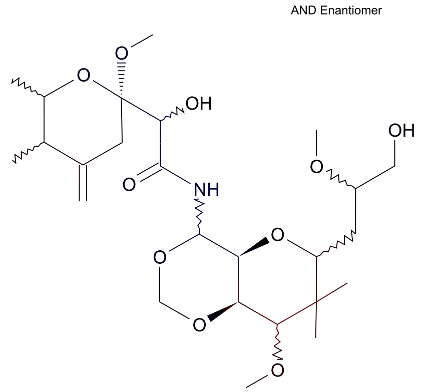

$C_{25}H_{43}NO_{10}$
Molecular Weight: 517.60962
ALogP: -9.1e-002
Rotatable Bonds: 9
Acceptors: 10
Donors: 3

Model Prediction

Prediction: Multiple-Carcinogen
Probability: 0.256
Enrichment: 0.850
Bayesian Score: -0.144
Mahalanobis Distance: 11.664
Mahalanobis Distance p-value: 0.0011

Prediction: Positive if the Bayesian score is above the estimated best cutoff value from minimizing the false positive and false negative rate.
Probability: The esimated probability that the sample is in the positive category. This assumes that the Bayesian score follows a normal distribution and is different from the prediction using a cutoff.
Enrichment: An estimate of enrichment, that is, the increased likelihood (versus random) of this sample being in the category.
Bayesian Score: The standard Laplacian-modified Bayesian score.
Mahalanobis Distance: The Mahalanobis distance (MD) is the distance to the center of the training data. The larger the MD, the less trustworthy the prediction.
Mahalanobis Distance p-value: The p-value gives the fraction of training data with an MD greater than or equal to the one for the given sample, assuming normally distributed data. The smaller the p-value, the less trustworthy the prediciton. For highly non-normal X properties (e.g., fingerprints), the MD p-value is wildly inaccurate.

Structural Similar Compounds

Name	Methotrexate	Sulfasalazine	Primidolol
Structure	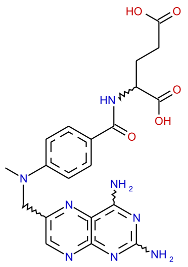	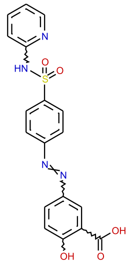	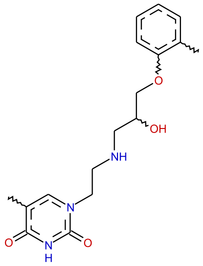
Actual Endpoint	Single-Carcinogen	Single-Carcinogen	Single-Carcinogen
Predicted Endpoint	Single-Carcinogen	Single-Carcinogen	Single-Carcinogen
Distance	0.993	0.996	1.083
Reference	US FDA (Centre for Drug Eval.& Res./Off. Testing & Res.) Sept. 1997	US FDA (Centre for Drug Eval.& Res./Off. Testing & Res.) Sept. 1997	US FDA (Centre for Drug Eval.& Res./Off. Testing & Res.) Sept. 1997

Model Applicability

Unknown features are fingerprint features in the query molecule, but not found in the training set.

1. All properties and OPS components are within expected ranges.
2. Unknown FCFP_2 feature: 470041467: [*]OCO[*]
3. Unknown FCFP_2 feature: 129344189: [*]C(=C)[*]

Feature Contribution

Top features for positive contribution

Fingerprint	Bit/Smiles	Feature Structure	Score	Multiple-Carcinogen in training set
FCFP_12	-1043310069	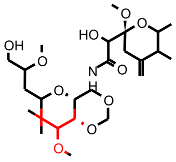 [*]OC(C([*])[*])C([*])([*])[*]	0.597	7 out of 10

FCFP_12	-415245925	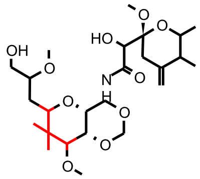 [*]C[C@]1(C)[C@@H]([*])[*][*]C1([*])[*]	0.535	10 out of 16
FCFP_12	346066116	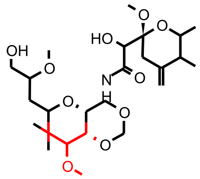 [*]C([*])C(OC)C([*])([*])[*]	0.400	1 out of 1

Top Features for negative contribution

Fingerprint	Bit/Smiles	Feature Structure	Score	Multiple-Carcinogen in training set
FCFP_12	566058135	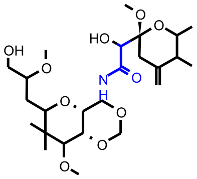 [*]NC(=O)C([*])[*]	-0.528	3 out of 17
FCFP_12	699559848	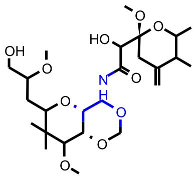 [*]NC(O[*])C([*])[*]	-0.519	0 out of 2
FCFP_12	-675291618	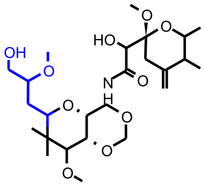 [*]C([*])CC(CO)OC	-0.293	0 out of 1

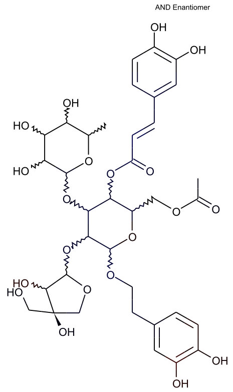

$C_{36}H_{46}O_{20}$
Molecular Weight: 798.73843
ALogP: -0.546
Rotatable Bonds: 16
Acceptors: 20
Donors: 10

Structural Similar Compounds

Name	Methanol; (s-triazine-2;4;6-triyltrinitrilo)hexa-	2-Naphthalenesulfonic acid; 5;6'-iminobis(1-hydroxy-	1;3;6-NAPHTHALENE TRISULFONIC ACID;7-AMINO-
Structure	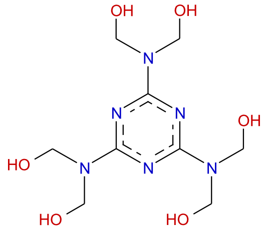	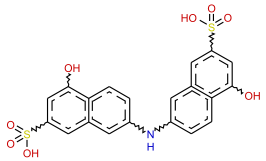	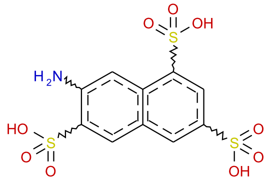
Actual Endpoint	Moderate_Severe	Mild	Mild
Predicted Endpoint	Moderate_Severe	Mild	Mild
Distance	1.957	2.188	2.360
Reference	Prehled Prumyslove Toxikologie; Organicke Latky; Marhold; J. -;876;86	Prehled Prumyslove Toxikologie; Organicke Latky; Marhold; J. pp 1065;86	28ZPAK-;190;72

Model Prediction

Prediction: Mild
Probability: 0.781
Enrichment: 1.134
Bayesian Score: -1.654
Mahalanobis Distance: 23.979
Mahalanobis Distance p-value: 4.97e-058

Prediction: Positive if the Bayesian score is above the estimated best cutoff value from minimizing the false positive and false negative rate.
Probability: The esimated probability that the sample is in the positive category. This assumes that the Bayesian score follows a normal distribution and is different from the prediction using a cutoff.
Enrichment: An estimate of enrichment, that is, the increased likelihood (versus random) of this sample being in the category.
Bayesian Score: The standard Laplacian-modified Bayesian score.
Mahalanobis Distance: The Mahalanobis distance (MD) is the distance to the center of the training data. The larger the MD, the less trustworthy the prediction.
Mahalanobis Distance p-value: The p-value gives the fraction of training data with an MD greater than or equal to the one for the given sample, assuming normally distributed data. The smaller the p-value, the less trustworthy the prediciton. For highly non-normal X properties (e.g., fingerprints), the MD p-value is wildly inaccurate.

Model Applicability

Unknown features are fingerprint features in the query molecule, but not found in the training set.

1. Num_H_Donors out of range. Value: 10. Training min, max, mean, SD: 0, 6, 0.71878, 0.8939.
2. Num_H_Acceptors out of range. Value: 20. Training min, max, mean, SD: 0, 19, 2.6994, 2.203.
3. OPS PC8 out of range. Value: -4.4632. Training min, max, SD, explained variance: -3.8477, 8.3204, 1.516, 0.0280.

Feature Contribution

Top features for positive contribution

Fingerprint	Bit/Smiles	Feature Structure	Score	Moderate_Severe in training set
FCFP_10	-497728148	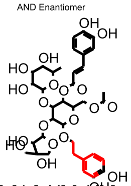 [*]CC[c]1:[cH]:[cH]:[*]:[c]([*]):[cH]:1	0.356	24 out of 25

FCFP_10	-1272709286	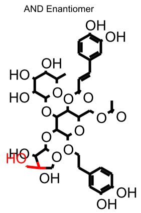<br>[*]C([*])([*])CO	0.285	234 out of 266
FCFP_10	1070061035	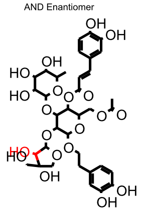<br>[*]C([*])O	0.239	284 out of 338
**Top Features for negative contribution**				
**Fingerprint**	**Bit/Smiles**	**Feature Structure**	**Score**	**Moderate_Severe in training set**
FCFP_10	-428284881	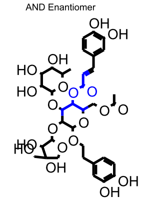<br>[*]C([*])C(OC(=O)C=[*<br>])C([*])[*]	-0.580	4 out of 12
FCFP_10	1132363037	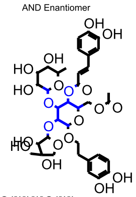<br>[*]OC1C([*])[*]C([*])<br>C(O[*])C1OC([*])[*]	-0.507	0 out of 1
FCFP_10	-146015125	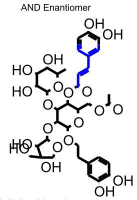<br>[*]C(=[*])\C=C\[c](:[<br>cH]:[*]):[cH]:[*]	-0.507	0 out of 1

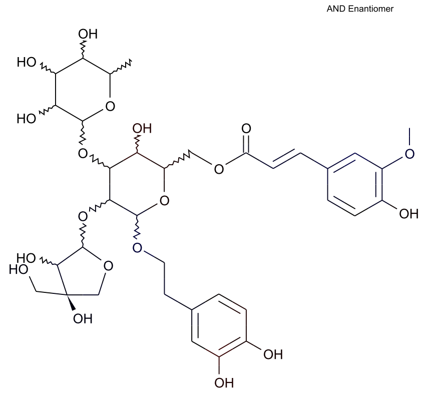

$C_{35}H_{46}O_{19}$
Molecular Weight: 770.72833
ALogP: -0.699
Rotatable Bonds: 15
Acceptors: 19
Donors: 10

Structural Similar Compounds

Name	Methanol; (s-triazine-2;4;6-triyltrinitrilo)hexa-	2-Naphthalenesulfonic acid; 5;6'-iminobis(1-hydroxy-	1;3;6-NAPHTHALENE TRISULFONIC ACID;7-AMINO-
Structure	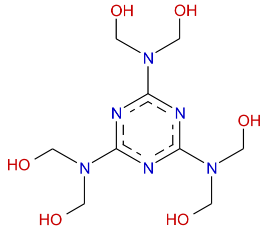	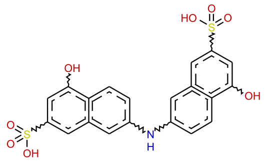	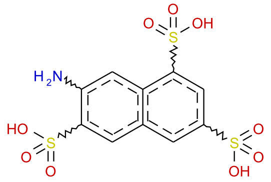
Actual Endpoint	Moderate_Severe	Mild	Mild
Predicted Endpoint	Moderate_Severe	Mild	Mild
Distance	1.882	2.119	2.297
Reference	Prehled Prumyslove Toxikologie; Organicke Latky; Marhold; J. -;876;86	Prehled Prumyslove Toxikologie; Organicke Latky; Marhold; J. pp 1065;86	28ZPAK-;190;72

Model Prediction

Prediction: Mild
Probability: 0.789
Enrichment: 1.145
Bayesian Score: -1.397
Mahalanobis Distance: 23.079
Mahalanobis Distance p-value: 1.07e-052

Prediction: Positive if the Bayesian score is above the estimated best cutoff value from minimizing the false positive and false negative rate.
Probability: The esimated probability that the sample is in the positive category. This assumes that the Bayesian score follows a normal distribution and is different from the prediction using a cutoff.
Enrichment: An estimate of enrichment, that is, the increased likelihood (versus random) of this sample being in the category.
Bayesian Score: The standard Laplacian-modified Bayesian score.
Mahalanobis Distance: The Mahalanobis distance (MD) is the distance to the center of the training data. The larger the MD, the less trustworthy the prediction.
Mahalanobis Distance p-value: The p-value gives the fraction of training data with an MD greater than or equal to the one for the given sample, assuming normally distributed data. The smaller the p-value, the less trustworthy the prediciton. For highly non-normal X properties (e.g., fingerprints), the MD p-value is wildly inaccurate.

Model Applicability

Unknown features are fingerprint features in the query molecule, but not found in the training set.

1. Num_H_Donors out of range. Value: 10. Training min, max, mean, SD: 0, 6, 0.71878, 0.8939.
2. OPS PC8 out of range. Value: -4.2603. Training min, max, SD, explained variance: -3.8477, 8.3204, 1.516, 0.0280.

Feature Contribution

Top features for positive contribution

Fingerprint	Bit/Smiles	Feature Structure	Score	Moderate_Severe in training set
FCFP_10	-497728148	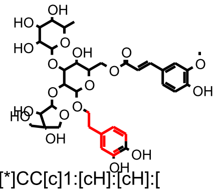 [*]CC[c]1:[cH]:[cH]:[*]:[c]([*]):[cH]:1	0.356	24 out of 25

FCFP_10	-2106701143	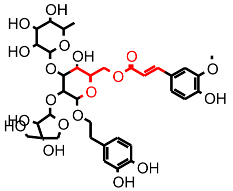 [*]OC(COC(=O)\C=C\[*])C([*])[*]	0.294	3 out of 3
FCFP_10	-1272709286	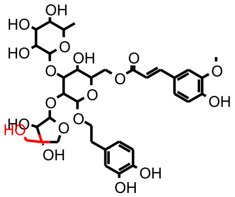 [*]C([*])([*])CO	0.285	234 out of 266
**Top Features for negative contribution**				
**Fingerprint**	**Bit/Smiles**	**Feature Structure**	**Score**	**Moderate_Severe in training set**
FCFP_10	-1977641857	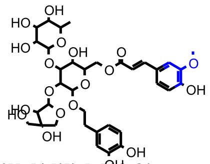 [*][c](:[*]):[c](OC): [cH]:[*]	-0.780	4 out of 15
FCFP_10	-2005207466	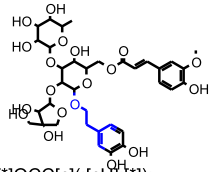 [*]OCC[c](:[cH]:[*]): [cH]:[*]	-0.507	0 out of 1
FCFP_10	205247261	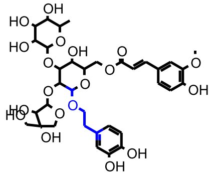 [*]C([*])OCC[c](:[*]) :[*]	-0.507	0 out of 1

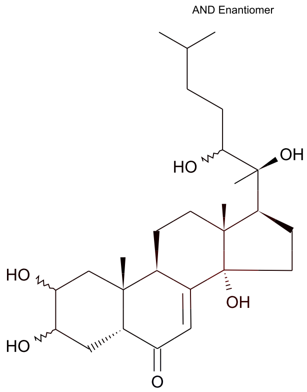

$C_{27}H_{44}O_6$
Molecular Weight: 464.63466
ALogP: 2.489
Rotatable Bonds: 5
Acceptors: 6
Donors: 5

Model Prediction

Prediction: Moderate_Severe
Probability: 0.850
Enrichment: 1.233
Bayesian Score: 1.869
Mahalanobis Distance: 13.747
Mahalanobis Distance p-value: 1.98e-009

Prediction: Positive if the Bayesian score is above the estimated best cutoff value from minimizing the false positive and false negative rate.
Probability: The esimated probability that the sample is in the positive category. This assumes that the Bayesian score follows a normal distribution and is different from the prediction using a cutoff.
Enrichment: An estimate of enrichment, that is, the increased likelihood (versus random) of this sample being in the category.
Bayesian Score: The standard Laplacian-modified Bayesian score.
Mahalanobis Distance: The Mahalanobis distance (MD) is the distance to the center of the training data. The larger the MD, the less trustworthy the prediction.
Mahalanobis Distance p-value: The p-value gives the fraction of training data with an MD greater than or equal to the one for the given sample, assuming normally distributed data. The smaller the p-value, the less trustworthy the prediciton. For highly non-normal X properties (e.g., fingerprints), the MD p-value is wildly inaccurate.

Structural Similar Compounds

Name	ANTHRAQUINONE; 1;5-DIAMINO-4;8-DIHYDROXY-3-(p-METHOXYPHENYL)-	2-Naphthalenesulfonic acid; 5;6'-iminobis(1-hydroxy-	4;4'-DIAMINO-1;1'-DIANTHRIMIDE
Structure	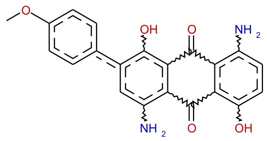	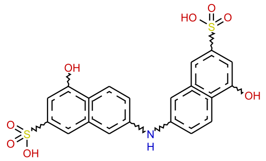	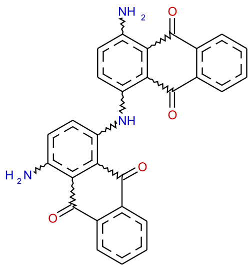
Actual Endpoint	Mild	Mild	Mild
Predicted Endpoint	Mild	Mild	Mild
Distance	0.847	0.902	0.990
Reference	28ZPAK 245;72	Prehled Prumyslove Toxikologie; Organicke Latky; Marhold; J. pp 1065;86	28ZPAK-;125;72

Model Applicability

Unknown features are fingerprint features in the query molecule, but not found in the training set.

1. All properties and OPS components are within expected ranges.

Feature Contribution

Top features for positive contribution				
Fingerprint	Bit/Smiles	Feature Structure	Score	Moderate_Severe in training set
FCFP_10	-1742546106	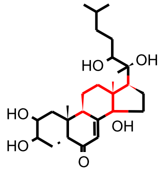 [*][C@@H]1[*][C@]2([*])[*][*][C@H]([*])[C@@]2(C)CC1	0.317	4 out of 4

FCFP_10	1904223630	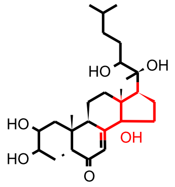 [*][C@H]1CC[C@@](O)(C(=[*])[*])C1([*])[*]	0.294	3 out of 3
FCFP_10	1002543125	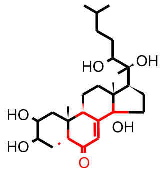 [*][C@@H]1[*][C@@H]([*])C(=CC1=O)C([*])([*])[*]	0.256	2 out of 2

Top Features for negative contribution

Fingerprint	Bit/Smiles	Feature Structure	Score	Moderate_Severe in training set
FCFP_10	565968762	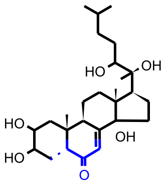 [*]C([*])C(=O)C=[*]	-0.372	17 out of 38
FCFP_10	1872154524	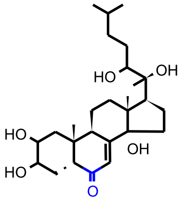 [*]C(=O)[*]	-0.078	344 out of 563
FCFP_10	136597326	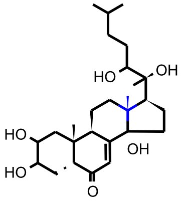 [*]C([*])C	0.000	422 out of 612

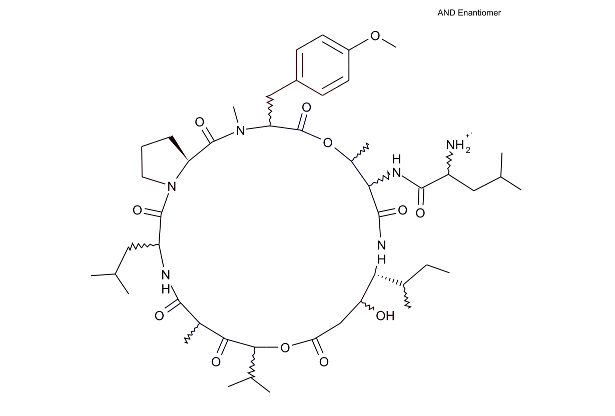

$C_{49}H_{79}N_6O_{12}$
Molecular Weight: 944.18455
ALogP: 3.176
Rotatable Bonds: 13
Acceptors: 12
Donors: 5

Model Prediction

Prediction: Moderate_Severe
Probability: 0.817
Enrichment: 1.186
Bayesian Score: -0.256
Mahalanobis Distance: 23.061
Mahalanobis Distance p-value: 1.36e-052

Prediction: Positive if the Bayesian score is above the estimated best cutoff value from minimizing the false positive and false negative rate.
Probability: The esimated probability that the sample is in the positive category. This assumes that the Bayesian score follows a normal distribution and is different from the prediction using a cutoff.
Enrichment: An estimate of enrichment, that is, the increased likelihood (versus random) of this sample being in the category.
Bayesian Score: The standard Laplacian-modified Bayesian score.
Mahalanobis Distance: The Mahalanobis distance (MD) is the distance to the center of the training data. The larger the MD, the less trustworthy the prediction.
Mahalanobis Distance p-value: The p-value gives the fraction of training data with an MD greater than or equal to the one for the given sample, assuming normally distributed data. The smaller the p-value, the less trustworthy the prediciton. For highly non-normal X properties (e.g., fingerprints), the MD p-value is wildly inaccurate.

Structural Similar Compounds

Name	2-Naphthalenesulfonic acid; 5;6'-iminobis(1-hydroxy-	2;7-NAPHTHALENE DISULFONIC ACID;4-AMINO-5-HYDROXY-;P-TOLUENE SULFONATE (ESTER)	2;7-Naphthalenedisulfonic acid;
Structure	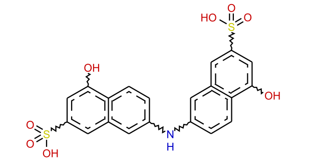	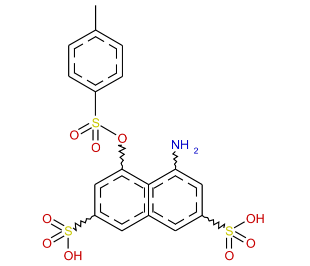	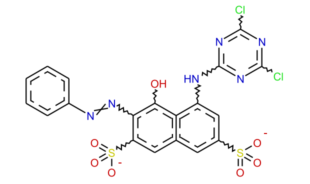
Actual Endpoint	Mild	Moderate_Severe	Moderate_Severe
Predicted Endpoint	Mild	Moderate_Severe	Moderate_Severe
Distance	1.447	1.525	1.527
Reference	Prehled Prumyslove Toxikologie; Organicke Latky; Marhold; J. pp 1065;86	28ZPAK-;194;72	Prehled Prumyslove Toxikologie; Organicke Latky; Marhold; J. - ;1300;86

Model Applicability

Unknown features are fingerprint features in the query molecule, but not found in the training set.

1. OPS PC11 out of range. Value: 5.5126. Training min, max, SD, explained variance: -5.8277, 5.3336, 1.291, 0.0203.
2. OPS PC18 out of range. Value: -4.5689. Training min, max, SD, explained variance: -4.1571, 3.8727, 1.069, 0.0139.
3. Unknown FCFP_2 feature: 10: [*][NH2+][*]
4. Unknown FCFP_2 feature: 18028531: [*]C([*])[NH2+]C
5. Unknown FCFP_2 feature: -1817836174: [*]CC([NH2+][*])C(=[*])[*]
6. Unknown FCFP_2 feature: 136418580: [*][NH2+]C

Feature Contribution

Top features for positive contribution

Fingerprint	Bit/Smiles	Feature Structure	Score	Moderate_Severe in training set

FCFP_10	1985089045	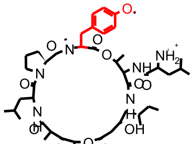 [*]O[c]1:[cH]:[cH]:[c](CC([*])[*]):[cH]:[cH]:1	0.385	16 out of 16
FCFP_10	-497728148	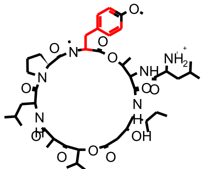 [*]CC[c]1:[cH]:[cH]:[*]:[c]([*]):[cH]:1	0.356	24 out of 25
FCFP_10	-1034142694	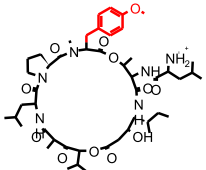 [*]C[c]1:[cH]:[cH]:[c](OC):[cH]:[cH]:1	0.338	18 out of 19

Top Features for negative contribution

Fingerprint	Bit/Smiles	Feature Structure	Score	Moderate_Severe in training set
FCFP_10	-1977641857	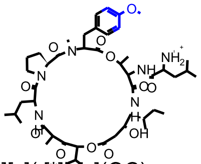 [*][c](:[*]):[c](OC):[cH]:[*]	-0.780	4 out of 15
FCFP_10	-428284881	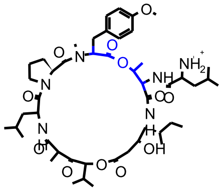 [*]C([*])C(OC(=O)C=[*])C([*])[*]	-0.580	4 out of 12

FCFP_10	-551279842	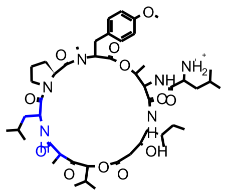 [*]CC(NC(=O)C([*])[*])C(=[*])[*]	-0.507	0 out of 1

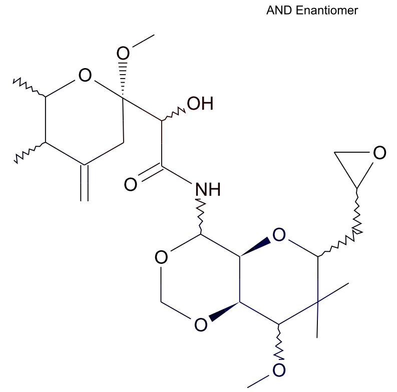

$C_{24}H_{39}NO_9$
Molecular Weight: 485.56776
ALogP: 0.267
Rotatable Bonds: 7
Acceptors: 9
Donors: 2

Structural Similar Compounds

Name	Fumaric acid; bis(3-allyloxy-2-hydroxypropyl) ester	COLCHICINE	2H-Naphtho(1;2-d)triazole-6;8-disulfonic acid;
Structure	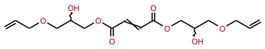	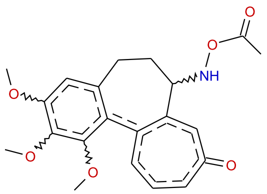	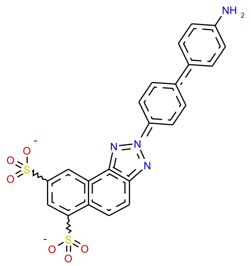
Actual Endpoint	Moderate_Severe	Moderate_Severe	Mild
Predicted Endpoint	Moderate_Severe	Moderate_Severe	Mild
Distance	0.841	0.876	0.883
Reference	Prehled Prumyslove Toxikologie; Organicke Latky; Marhold; J. -;646;86	AJOPAA 31;837;48	Prehled Prumyslove Toxikologie; Organicke Latky; Marhold; J. pp 1065;86

Model Prediction

Prediction: Mild
Probability: 0.807
Enrichment: 1.171
Bayesian Score: -0.741
Mahalanobis Distance: 14.994
Mahalanobis Distance p-value: 1.61e-013

Prediction: Positive if the Bayesian score is above the estimated best cutoff value from minimizing the false positive and false negative rate.
Probability: The esimated probability that the sample is in the positive category. This assumes that the Bayesian score follows a normal distribution and is different from the prediction using a cutoff.
Enrichment: An estimate of enrichment, that is, the increased likelihood (versus random) of this sample being in the category.
Bayesian Score: The standard Laplacian-modified Bayesian score.
Mahalanobis Distance: The Mahalanobis distance (MD) is the distance to the center of the training data. The larger the MD, the less trustworthy the prediction.
Mahalanobis Distance p-value: The p-value gives the fraction of training data with an MD greater than or equal to the one for the given sample, assuming normally distributed data. The smaller the p-value, the less trustworthy the prediciton. For highly non-normal X properties (e.g., fingerprints), the MD p-value is wildly inaccurate.

Model Applicability

Unknown features are fingerprint features in the query molecule, but not found in the training set.

1. All properties and OPS components are within expected ranges.

Feature Contribution

Top features for positive contribution				
**Fingerprint**	**Bit/Smiles**	**Feature Structure**	**Score**	**Moderate_Severe in training set**
FCFP_10	1657756157	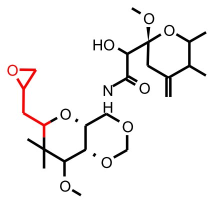 [*]C([*])CC1CO1	0.294	3 out of 3

FCFP_10	-885550502	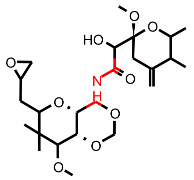 [*]C([*])NC(=[*])[*]	0.239	54 out of 64
FCFP_10	1070061035	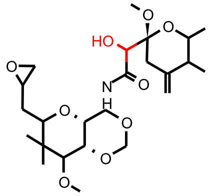 [*]C([*])O	0.239	284 out of 338
**Top Features for negative contribution**				
**Fingerprint**	**Bit/Smiles**	**Feature Structure**	**Score**	**Moderate_Severe in training set**
FCFP_10	1132363037	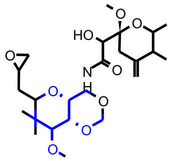 [*]OC1C([*])[*]C([*]) C(O[*])C1OC([*])[*]	-0.507	0 out of 1
FCFP_10	638583477	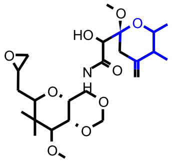 [*]C1([*])[*]C(=[*])C (C)C(C)O1	-0.507	0 out of 1
FCFP_10	-987676025	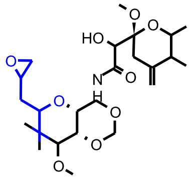 [*]OC(CC1CO1)C([*])([*])[*]	-0.507	0 out of 1

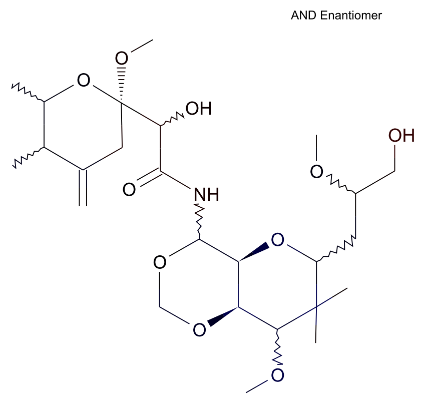

$C_{25}H_{43}NO_{10}$
Molecular Weight: 517.60962
ALogP: -9.1e-002
Rotatable Bonds: 9
Acceptors: 10
Donors: 3

Model Prediction

Prediction: Mild
Probability: 0.809
Enrichment: 1.174
Bayesian Score: -0.646
Mahalanobis Distance: 14.434
Mahalanobis Distance p-value: 1.34e-011

Prediction: Positive if the Bayesian score is above the estimated best cutoff value from minimizing the false positive and false negative rate.
Probability: The esimated probability that the sample is in the positive category. This assumes that the Bayesian score follows a normal distribution and is different from the prediction using a cutoff.
Enrichment: An estimate of enrichment, that is, the increased likelihood (versus random) of this sample being in the category.
Bayesian Score: The standard Laplacian-modified Bayesian score.
Mahalanobis Distance: The Mahalanobis distance (MD) is the distance to the center of the training data. The larger the MD, the less trustworthy the prediction.
Mahalanobis Distance p-value: The p-value gives the fraction of training data with an MD greater than or equal to the one for the given sample, assuming normally distributed data. The smaller the p-value, the less trustworthy the prediciton. For highly non-normal X properties (e.g., fingerprints), the MD p-value is wildly inaccurate.

Structural Similar Compounds

Name	Fumaric acid; bis(3-allyloxy-2-hydroxypropyl) ester	2;7-NAPHTHALENE DISULFONIC ACID;4-AMINO-5-HYDROXY-;P-TOLUENE SULFONATE (ESTER)	2;7-Naphthalenedisulfonic acid;
Structure	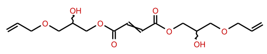	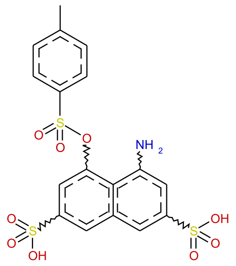	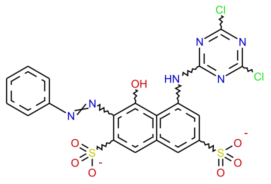
Actual Endpoint	Moderate_Severe	Moderate_Severe	Moderate_Severe
Predicted Endpoint	Moderate_Severe	Moderate_Severe	Moderate_Severe
Distance	0.942	0.943	0.994
Reference	Prehled Prumyslove Toxikologie; Organicke Latky; Marhold; J. -;646;86	28ZPAK-;194;72	Prehled Prumyslove Toxikologie; Organicke Latky; Marhold; J. -;1300;86

Model Applicability

Unknown features are fingerprint features in the query molecule, but not found in the training set.

1. All properties and OPS components are within expected ranges.

Feature Contribution

Top features for positive contribution

Fingerprint	Bit/Smiles	Feature Structure	Score	Moderate_Severe in training set
FCFP_10	-1272709286	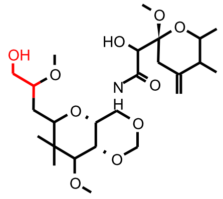 [*]C([*])([*])CO	0.285	234 out of 266

FCFP_10	-885550502	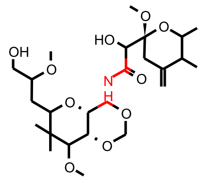<br>[*]C([*])NC(=[*])[*]	0.239	54 out of 64
FCFP_10	1070061035	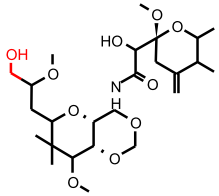<br>[*]C([*])O	0.239	284 out of 338
**Top Features for negative contribution**				
**Fingerprint**	**Bit/Smiles**	**Feature Structure**	**Score**	**Moderate_Severe in training set**
FCFP_10	638583477	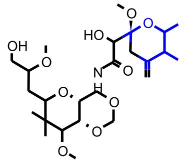<br>[*]C1([*])[*]C(=[*])C<br>(C)C(C)O1	-0.507	0 out of 1
FCFP_10	-987676025	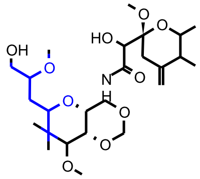<br>[*]OC(CC1CO1)C([*])([<br>*])[*]	-0.507	0 out of 1
FCFP_10	1132363037	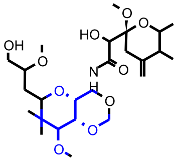<br>[*]OC1C([*])[*]C([*])<br>C(O[*])C1OC([*])[*]	-0.507	0 out of 1

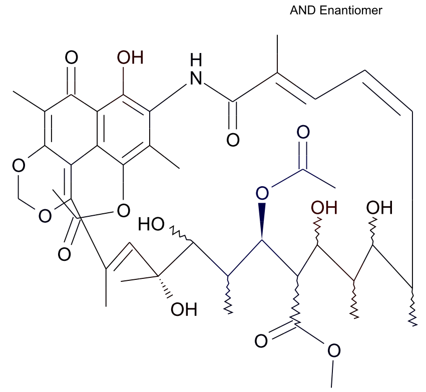

$C_{42}H_{53}NO_{15}$
Molecular Weight: 811.86791
ALogP: 2.745
Rotatable Bonds: 6
Acceptors: 15
Donors: 6

Model Prediction

Prediction: Mild
Probability: 0.796
Enrichment: 1.155
Bayesian Score: -1.163
Mahalanobis Distance: 18.715
Mahalanobis Distance p-value: 3.08e-029

Prediction: Positive if the Bayesian score is above the estimated best cutoff value from minimizing the false positive and false negative rate.
Probability: The esimated probability that the sample is in the positive category. This assumes that the Bayesian score follows a normal distribution and is different from the prediction using a cutoff.
Enrichment: An estimate of enrichment, that is, the increased likelihood (versus random) of this sample being in the category.
Bayesian Score: The standard Laplacian-modified Bayesian score.
Mahalanobis Distance: The Mahalanobis distance (MD) is the distance to the center of the training data. The larger the MD, the less trustworthy the prediction.
Mahalanobis Distance p-value: The p-value gives the fraction of training data with an MD greater than or equal to the one for the given sample, assuming normally distributed data. The smaller the p-value, the less trustworthy the prediciton. For highly non-normal X properties (e.g., fingerprints), the MD p-value is wildly inaccurate.

Structural Similar Compounds

Name	2-Naphthalenesulfonic acid; 5;6'-iminobis(1-hydroxy-	2;7-NAPHTHALENE DISULFONIC ACID;4-AMINO-5-HYDROXY-;P-TOLUENE SULFONATE (ESTER)	Methanol; (s-triazine-2;4;6-triyltrinitrilo)hexa-
Structure	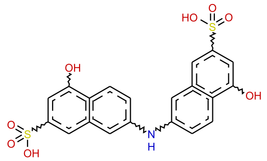	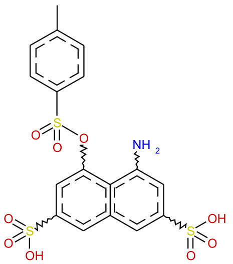	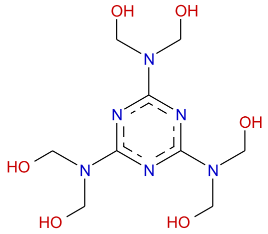
Actual Endpoint	Mild	Moderate_Severe	Moderate_Severe
Predicted Endpoint	Mild	Moderate_Severe	Moderate_Severe
Distance	1.359	1.530	1.538
Reference	Prehled Prumyslove Toxikologie; Organicke Latky; Marhold; J. pp 1065;86	28ZPAK-;194;72	Prehled Prumyslove Toxikologie; Organicke Latky; Marhold; J. -;876;86

Model Applicability

Unknown features are fingerprint features in the query molecule, but not found in the training set.

1. OPS PC20 out of range. Value: -3.8503. Training min, max, SD, explained variance: -3.1294, 4.2413, 1.03, 0.0129.

Feature Contribution

Top features for positive contribution

Fingerprint	Bit/Smiles	Feature Structure	Score	Moderate_Severe in training set
FCFP_10	-525166915	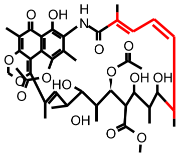 [*]C([*])\C=C/C=C([*])[*]	0.317	4 out of 4

FCFP_10	-1922181751	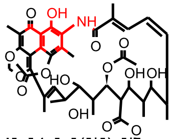 [*]N[c]1:[c]([*]):[*] :[c]([*]):[c](C(=[*])[*]):[c]:1O	0.256	2 out of 2
FCFP_10	56921271	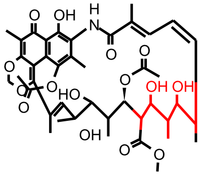 [*]C([*])C(O)C(C)C(O) C([*])[*]	0.256	2 out of 2

Top Features for negative contribution

Fingerprint	Bit/Smiles	Feature Structure	Score	Moderate_Severe in training set
FCFP_10	-528918648	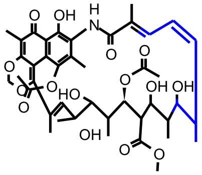 [*]C([*])C(C)\C=C/C=[*]	-0.651	4 out of 13
FCFP_10	-428284881	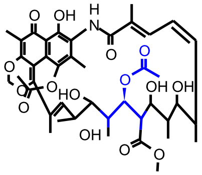 [*]C([*])C(OC(=O)C=[*])C([*])[*]	-0.580	4 out of 12
FCFP_10	-2052862541	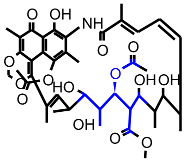 [*]C([*])C(C)[C@@H](O C(=[*])[*])C(C([*])[*])C(=[*])[*]	-0.507	0 out of 1

Simeprevir

TOPKAT_Ocular_Irritancy_Mild_vs_Moderate_Severe

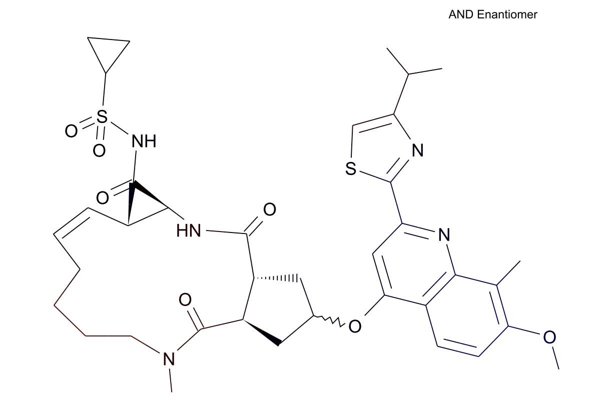

$C_{38}H_{47}N_5O_7S_2$

Molecular Weight: 749.93907

ALogP: 4.796

Rotatable Bonds: 8

Acceptors: 9

Donors: 2

Structural Similar Compounds

Name	Anthraquinone; 1;1'-(anthraquinon-1;5-ylenediimino)di-	Anthraquinone; 1;1'-(anthraquinon-1;4-ylenediimino)di-	4;4'-DIAMINO-1;1'-DIANTHRIMIDE
Structure	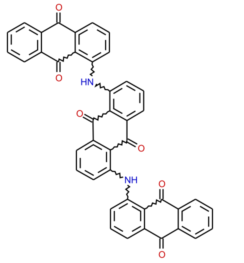	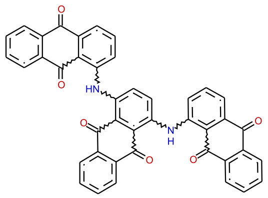	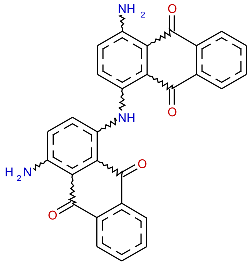
Actual Endpoint	Mild	Mild	Mild
Predicted Endpoint	Mild	Mild	Mild
Distance	0.875	0.875	1.102
Reference	Prehled Prumyslove Toxikologie; Organicke Latky; Marhold; J. pp 736;86	Prehled Prumyslove Toxikologie; Organicke Latky; Marhold; J. pp 736;86	28ZPAK-;125;72

Model Prediction

Prediction: Mild

Probability: 0.777

Enrichment: 1.128

Bayesian Score: -1.771

Mahalanobis Distance: 17.078

Mahalanobis Distance p-value: 9.74e-022

Prediction: Positive if the Bayesian score is above the estimated best cutoff value from minimizing the false positive and false negative rate.
Probability: The esimated probability that the sample is in the positive category. This assumes that the Bayesian score follows a normal distribution and is different from the prediction using a cutoff.
Enrichment: An estimate of enrichment, that is, the increased likelihood (versus random) of this sample being in the category.
Bayesian Score: The standard Laplacian-modified Bayesian score.
Mahalanobis Distance: The Mahalanobis distance (MD) is the distance to the center of the training data. The larger the MD, the less trustworthy the prediction.
Mahalanobis Distance p-value: The p-value gives the fraction of training data with an MD greater than or equal to the one for the given sample, assuming normally distributed data. The smaller the p-value, the less trustworthy the prediciton. For highly non-normal X properties (e.g., fingerprints), the MD p-value is wildly inaccurate.

Model Applicability

Unknown features are fingerprint features in the query molecule, but not found in the training set.

1. OPS PC15 out of range. Value: 5.5676. Training min, max, SD, explained variance: -4.4071, 5.1624, 1.138, 0.0158.

Feature Contribution

Top features for positive contribution

Fingerprint	Bit/Smiles	Feature Structure	Score	Moderate_Severe in training set
FCFP_10	-2015019763	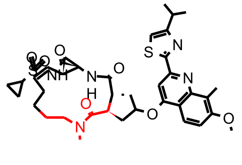 [*]CCN(C)C(=O)C([*])[*]	0.294	3 out of 3

FCFP_10	-124655670	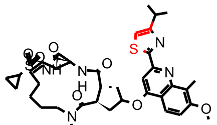 [*][c]1:[*]:[*]:s:[cH]:1	0.259	14 out of 16
FCFP_10	-1474971978	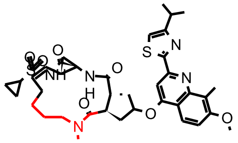 [*][C@@H]1CCCN1C(=[*])[*]	0.259	14 out of 16

Top Features for negative contribution

Fingerprint	Bit/Smiles	Feature Structure	Score	Moderate_Severe in training set
FCFP_10	-1977641857	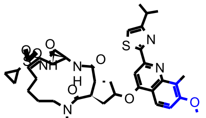 [*][c](:[*]):[c](OC): [cH]:[*]	-0.780	4 out of 15
FCFP_10	-528918648	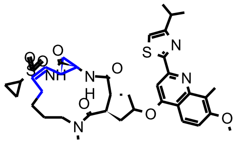 [*]C([*])C(C)\C=C/C=[*]	-0.651	4 out of 13
FCFP_10	690481386	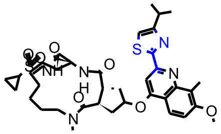 [*]:[c](:[*])[c]1:n:[*]:[*]:s:1	-0.600	1 out of 4

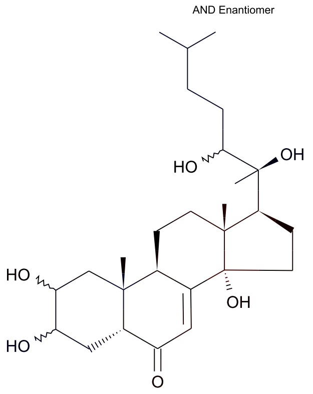

$C_{27}H_{44}O_6$
Molecular Weight: 464.63466
ALogP: 2.489
Rotatable Bonds: 5
Acceptors: 6
Donors: 5

Model Prediction

Prediction: Moderate
Probability: 0.664
Enrichment: 1.071
Bayesian Score: -0.774
Mahalanobis Distance: 14.969
Mahalanobis Distance p-value: 6.15e-014

Prediction: Positive if the Bayesian score is above the estimated best cutoff value from minimizing the false positive and false negative rate.
Probability: The esimated probability that the sample is in the positive category. This assumes that the Bayesian score follows a normal distribution and is different from the prediction using a cutoff.
Enrichment: An estimate of enrichment, that is, the increased likelihood (versus random) of this sample being in the category.
Bayesian Score: The standard Laplacian-modified Bayesian score.
Mahalanobis Distance: The Mahalanobis distance (MD) is the distance to the center of the training data. The larger the MD, the less trustworthy the prediction.
Mahalanobis Distance p-value: The p-value gives the fraction of training data with an MD greater than or equal to the one for the given sample, assuming normally distributed data. The smaller the p-value, the less trustworthy the prediciton. For highly non-normal X properties (e.g., fingerprints), the MD p-value is wildly inaccurate.

Structural Similar Compounds

Name	SULFIDE; BIS(DIHYDROXYPHENYL)	ANTHRACENE-9;10-DIOL;1;4-DIAMINO-	ANTHRAQUINONE;1;5-DIAMINO-4;8-DIHYDROXY-
Structure	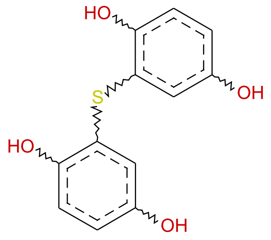	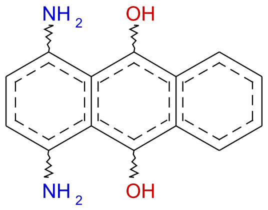	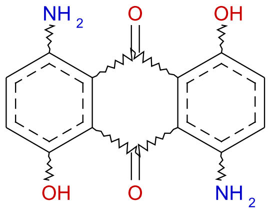
Actual Endpoint	Severe	Moderate	Moderate
Predicted Endpoint	Severe	Moderate	Moderate
Distance	1.045	1.058	1.085
Reference	IHFCAY 6;1;67	28ZPAK-;112;72	28ZPAK-;103;72

Model Applicability

Unknown features are fingerprint features in the query molecule, but not found in the training set.

1. All properties and OPS components are within expected ranges.

Feature Contribution

Top features for positive contribution				
**Fingerprint**	**Bit/Smiles**	**Feature Structure**	**Score**	**Severe in training set**
SCFP_12	-1742546106	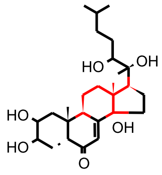 [*][C@@H]1[*][C@]2([*])[*][*][C@H]([*])[C@@]2(C)CC1	0.348	3 out of 3

SCFP_12	-415245925	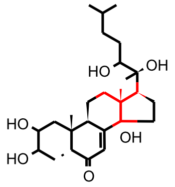 [*]C[C@]1(C)[C@@H]([*])[*][*]C1([*])[*]	0.312	12 out of 14
SCFP_12	571795252	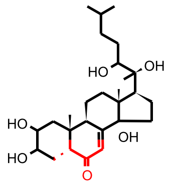 [*]C([*])C(=O)C=[*]	0.303	2 out of 2

Top Features for negative contribution

**Fingerprint**	**Bit/Smiles**	**Feature Structure**	**Score**	**Severe in training set**
SCFP_12	-1870530637	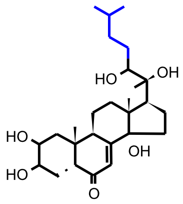 [*]CCC(C)C	-0.653	3 out of 11
SCFP_12	-1903488337	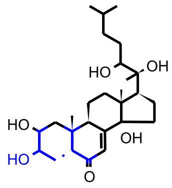 [*]C1[*]C([*])([*])[C @@H](CC1O)C(=[*])[*]	-0.475	0 out of 1
SCFP_12	1416196903	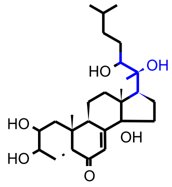 [*]C([*])C(C)(O)C([*])[*]	-0.383	3 out of 8

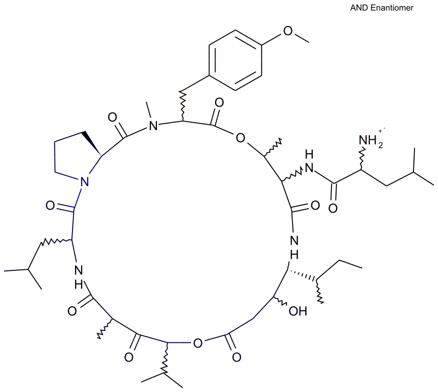

$C_{49}H_{79}N_6O_{12}$
Molecular Weight: 944.18455
ALogP: 3.176
Rotatable Bonds: 13
Acceptors: 12
Donors: 5

Model Prediction

Prediction: Moderate
Probability: 0.325
Enrichment: 0.524
Bayesian Score: -6.874
Mahalanobis Distance: 22.527
Mahalanobis Distance p-value: 5.24e-047

Prediction: Positive if the Bayesian score is above the estimated best cutoff value from minimizing the false positive and false negative rate.
Probability: The esimated probability that the sample is in the positive category. This assumes that the Bayesian score follows a normal distribution and is different from the prediction using a cutoff.
Enrichment: An estimate of enrichment, that is, the increased likelihood (versus random) of this sample being in the category.
Bayesian Score: The standard Laplacian-modified Bayesian score.
Mahalanobis Distance: The Mahalanobis distance (MD) is the distance to the center of the training data. The larger the MD, the less trustworthy the prediction.
Mahalanobis Distance p-value: The p-value gives the fraction of training data with an MD greater than or equal to the one for the given sample, assuming normally distributed data. The smaller the p-value, the less trustworthy the prediciton. For highly non-normal X properties (e.g., fingerprints), the MD p-value is wildly inaccurate.

Structural Similar Compounds

Name	2;7-NAPHTHALENE DISULFONIC ACID;4-AMINO-5-HYDROXY-;P-TOLUENE SULFONATE (ESTER)	2;7-Naphthalenedisulfonic acid;	Nonoxynol-10
Structure	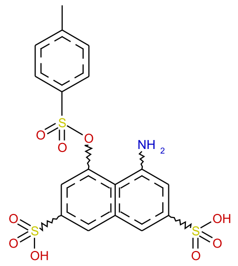	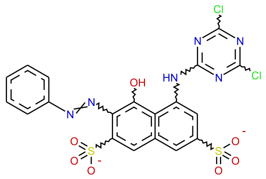	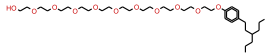
Actual Endpoint	Moderate	Moderate	Severe
Predicted Endpoint	Moderate	Moderate	Severe
Distance	1.539	1.555	1.717
Reference	28ZPAK-;194;72	Prehled Prumyslove Toxikologie; Organicke Latky; Marhold; J. -;1300;86	J. Am. Coll. Toxicol. 2(7):35;1983

Model Applicability

Unknown features are fingerprint features in the query molecule, but not found in the training set.

1. Molecular_Weight out of range. Value: 944.18. Training min, max, mean, SD: 30.026, 925.19, 182.4, 118.7.
2. OPS PC13 out of range. Value: 3.858. Training min, max, SD, explained variance: -4.8039, 3.7347, 1.133, 0.0169.
3. OPS PC17 out of range. Value: -3.6159. Training min, max, SD, explained variance: -3.5376, 3.4565, 1.013, 0.0135.

Feature Contribution

Top features for positive contribution				
Fingerprint	Bit/Smiles	Feature Structure	Score	Severe in training set

SCFP_12	-1580903393	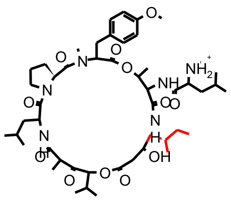 [*]C([*])C(C)CC	0.268	17 out of 21
SCFP_12	-1272709286	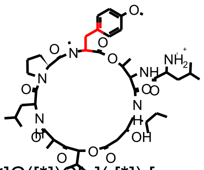 [*]C([*])C[c](:[*]):[*]	0.231	24 out of 31
SCFP_12	136448371	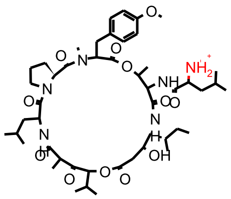 [*][NH2+]C	0.218	1 out of 1

Top Features for negative contribution

Fingerprint	Bit/Smiles	Feature Structure	Score	Severe in training set
SCFP_12	571765461	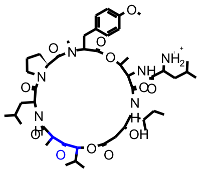 [*]C([*])C(=O)C([*])[*]	-1.017	2 out of 12
SCFP_12	-1109046624	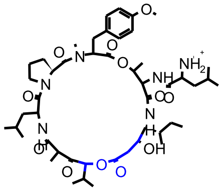 [*]C([*])CC(=O)OC([*])[*]	-0.769	2 out of 9

SCFP_12	-1343150366	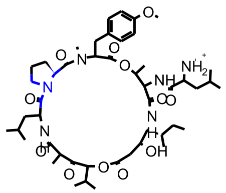 [*][C@@H]1[*][*]CN1C(=[*])[*]	-0.667	7 out of 24

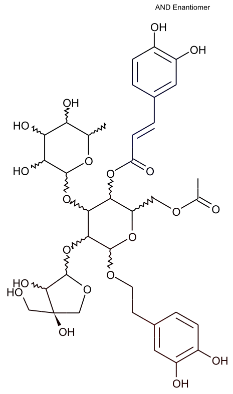

$C_{36}H_{46}O_{20}$
Molecular Weight: 798.73843
ALogP: -0.546
Rotatable Bonds: 16
Acceptors: 20
Donors: 10

Structural Similar Compounds

Name	Methanol; (s-triazine-2;4;6-triyltrinitrilo)hexa-	2-Naphthalenesulfonic acid; 5;6'-iminobis(1-hydroxy-	1;3;6-NAPHTHALENE TRISULFONIC ACID;7-AMINO-
Structure	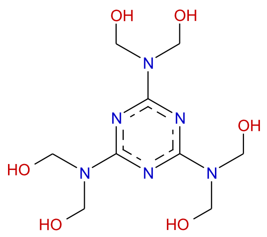	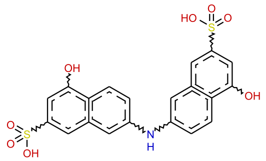	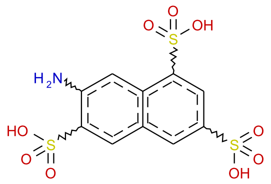
Actual Endpoint	Irritant	Irritant	Irritant
Predicted Endpoint	Irritant	Irritant	Irritant
Distance	1.918	2.143	2.327
Reference	Prehled Prumyslove Toxikologie; Organicke Latky; Marhold; J. -;876;86	Prehled Prumyslove Toxikologie; Organicke Latky; Marhold; J. pp 1065;86	28ZPAK-;190;72

Model Prediction

Prediction: Irritant
Probability: 1.000
Enrichment: 1.175
Bayesian Score: 0.772
Mahalanobis Distance: 22.937
Mahalanobis Distance p-value: 1.22e-053

Prediction: Positive if the Bayesian score is above the estimated best cutoff value from minimizing the false positive and false negative rate.
Probability: The esimated probability that the sample is in the positive category. This assumes that the Bayesian score follows a normal distribution and is different from the prediction using a cutoff.
Enrichment: An estimate of enrichment, that is, the increased likelihood (versus random) of this sample being in the category.
Bayesian Score: The standard Laplacian-modified Bayesian score.
Mahalanobis Distance: The Mahalanobis distance (MD) is the distance to the center of the training data. The larger the MD, the less trustworthy the prediction.
Mahalanobis Distance p-value: The p-value gives the fraction of training data with an MD greater than or equal to the one for the given sample, assuming normally distributed data. The smaller the p-value, the less trustworthy the prediciton. For highly non-normal X properties (e.g., fingerprints), the MD p-value is wildly inaccurate.

Model Applicability

Unknown features are fingerprint features in the query molecule, but not found in the training set.

1. Num_H_Donors out of range. Value: 10. Training min, max, mean, SD: 0, 6, 0.68472, 0.8931.
2. OPS PC7 out of range. Value: 9.7097. Training min, max, SD, explained variance: -6.07, 6.0201, 1.57, 0.0301.

Feature Contribution

Top features for positive contribution

Fingerprint	Bit/Smiles	Feature Structure	Score	Irritant in training set
FCFP_12	-415156552	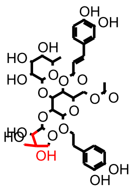 AND Enantiomer [*]C[C@@]1(O)C[*][*]C1[*]	0.184	7 out of 7

FCFP_12	-204034463	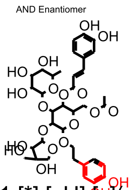 [*][c]1:[*]:[cH]:[c](O):[c](O):[cH]:1	0.175	5 out of 5
FCFP_12	750347608	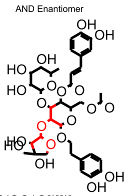 [*]C([*])C(OC1O[*][*]C1[*])C([*])[*]	0.156	3 out of 3
**Top Features for negative contribution**				
**Fingerprint**	**Bit/Smiles**	**Feature Structure**	**Score**	**Irritant in training set**
FCFP_12	-1078052987	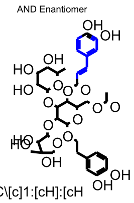 [*]\C=C\[c]1:[cH]:[cH]:[*]:[c]([*]):[cH]:1	-0.344	2 out of 4
FCFP_12	-146015125	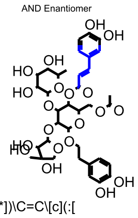 [*]C(=[*])\C=C\[c](:[cH]:[*]):[cH]:[*]	-0.268	1 out of 2
FCFP_12	-432846198	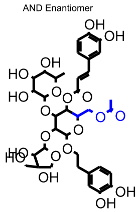 [*]C([*])COC(=O)C	-0.229	91 out of 142

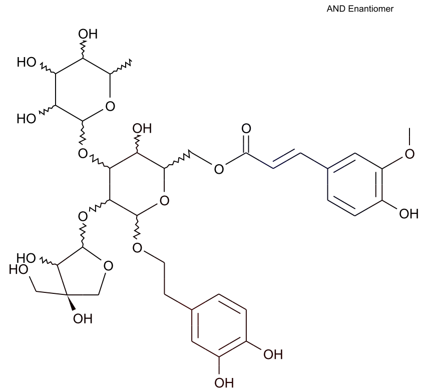

$C_{35}H_{46}O_{19}$
Molecular Weight: 770.72833
ALogP: -0.699
Rotatable Bonds: 15
Acceptors: 19
Donors: 10

Structural Similar Compounds

Name	Methanol; (s-triazine-2;4;6-triyltrinitrilo)hexa-	2-Naphthalenesulfonic acid; 5;6'-iminobis(1-hydroxy-	1;3;6-NAPHTHALENE TRISULFONIC ACID;7-AMINO-
Structure	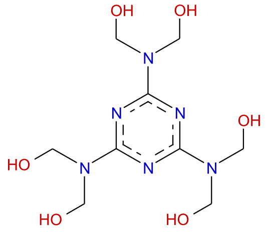	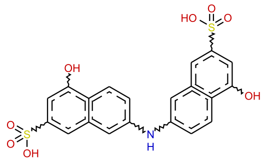	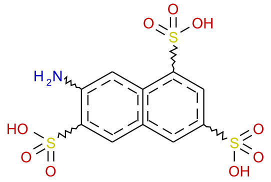
Actual Endpoint	Irritant	Irritant	Irritant
Predicted Endpoint	Irritant	Irritant	Irritant
Distance	1.848	2.078	2.269
Reference	Prehled Prumyslove Toxikologie; Organicke Latky; Marhold; J. -;876;86	Prehled Prumyslove Toxikologie; Organicke Latky; Marhold; J. pp 1065;86	28ZPAK-;190;72

Model Prediction

Prediction: Irritant
Probability: 1.000
Enrichment: 1.175
Bayesian Score: 1.187
Mahalanobis Distance: 21.625
Mahalanobis Distance p-value: 8.39e-046

Prediction: Positive if the Bayesian score is above the estimated best cutoff value from minimizing the false positive and false negative rate.
Probability: The esimated probability that the sample is in the positive category. This assumes that the Bayesian score follows a normal distribution and is different from the prediction using a cutoff.
Enrichment: An estimate of enrichment, that is, the increased likelihood (versus random) of this sample being in the category.
Bayesian Score: The standard Laplacian-modified Bayesian score.
Mahalanobis Distance: The Mahalanobis distance (MD) is the distance to the center of the training data. The larger the MD, the less trustworthy the prediction.
Mahalanobis Distance p-value: The p-value gives the fraction of training data with an MD greater than or equal to the one for the given sample, assuming normally distributed data. The smaller the p-value, the less trustworthy the prediciton. For highly non-normal X properties (e.g., fingerprints), the MD p-value is wildly inaccurate.

Model Applicability

Unknown features are fingerprint features in the query molecule, but not found in the training set.

1. Num_H_Donors out of range. Value: 10. Training min, max, mean, SD: 0, 6, 0.68472, 0.8931.
2. OPS PC7 out of range. Value: 9.2397. Training min, max, SD, explained variance: -6.07, 6.0201, 1.57, 0.0301.
3. OPS PC15 out of range. Value: 5.2325. Training min, max, SD, explained variance: -4.342, 4.1881, 1.08, 0.0142.

Feature Contribution

Top features for positive contribution				
Fingerprint	Bit/Smiles	Feature Structure	Score	Irritant in training set

FCFP_12	-415156552	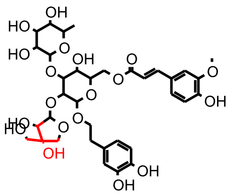<br>[*]C[C@@]1(O)C[*][*]C<br>1[*]	0.184	7 out of 7
FCFP_12	-204034463	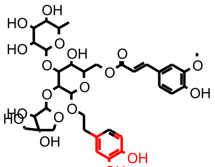<br>[*][c]1:[*]:[cH]:[c](<br>O):[c](O):[cH]:1	0.175	5 out of 5
FCFP_12	750347608	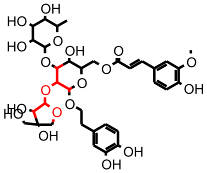<br>[*]C([*])C(OC1O[*][*]<br>C1[*])C([*])[*]	0.156	3 out of 3
**Top Features for negative contribution**				
**Fingerprint**	**Bit/Smiles**	**Feature Structure**	**Score**	**Irritant in training set**
FCFP_12	-1078052987	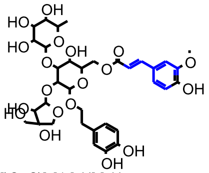<br>[*]\C=C\[c]1:[cH]:[cH<br>]:[*]:[c]([*]):[cH]:<br>1	-0.344	2 out of 4
FCFP_12	-146015125	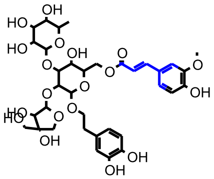<br>[*]C(=[*])\C=C\[c](:[<br>cH]:[*]):[cH]:[*]	-0.268	1 out of 2

FCFP_12	-432846198	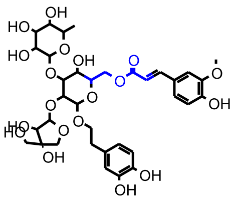 [*]C([*])COC(=O)C	-0.229	91 out of 142

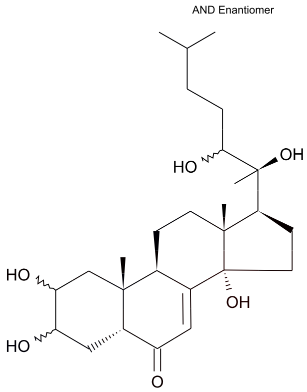

$C_{27}H_{44}O_6$
Molecular Weight: 464.63466
ALogP: 2.489
Rotatable Bonds: 5
Acceptors: 6
Donors: 5

Structural Similar Compounds

Name	ANTHRAQUINONE; 1;5-DIAMINO-4;8-DIHYDROXY-3-(p-METHOXYPHENYL)-	2-Naphthalenesulfonic acid; 5;6'-iminobis(1-hydroxy-	4;4'-DIAMINO-1;1'-DIANTHRIMIDE
Structure	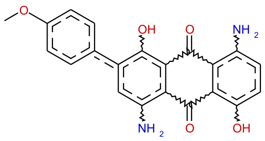	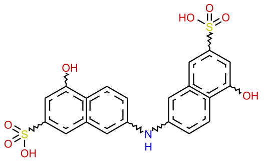	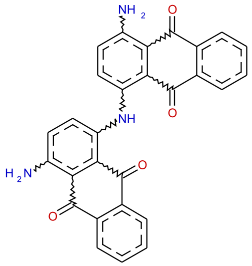
Actual Endpoint	Irritant	Irritant	Irritant
Predicted Endpoint	Irritant	Irritant	Irritant
Distance	0.843	0.897	0.986
Reference	28ZPAK 245;72	Prehled Prumyslove Toxikologie; Organicke Latky; Marhold; J. pp 1065;86	28ZPAK-;125;72

Model Prediction

Prediction: Irritant
Probability: 1.000
Enrichment: 1.175
Bayesian Score: 0.431
Mahalanobis Distance: 13.037
Mahalanobis Distance p-value: 1.63e-007

Prediction: Positive if the Bayesian score is above the estimated best cutoff value from minimizing the false positive and false negative rate.
Probability: The esimated probability that the sample is in the positive category. This assumes that the Bayesian score follows a normal distribution and is different from the prediction using a cutoff.
Enrichment: An estimate of enrichment, that is, the increased likelihood (versus random) of this sample being in the category.
Bayesian Score: The standard Laplacian-modified Bayesian score.
Mahalanobis Distance: The Mahalanobis distance (MD) is the distance to the center of the training data. The larger the MD, the less trustworthy the prediction.
Mahalanobis Distance p-value: The p-value gives the fraction of training data with an MD greater than or equal to the one for the given sample, assuming normally distributed data. The smaller the p-value, the less trustworthy the prediciton. For highly non-normal X properties (e.g., fingerprints), the MD p-value is wildly inaccurate.

Model Applicability

Unknown features are fingerprint features in the query molecule, but not found in the training set.

1. All properties and OPS components are within expected ranges.

Feature Contribution

Top features for positive contribution

Fingerprint	Bit/Smiles	Feature Structure	Score	Irritant in training set
FCFP_12	-415156552	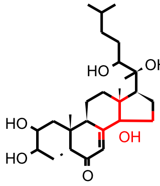 [*]C[C@@]1(O)C[*][*]C 1[*]	0.184	7 out of 7

FCFP_12	1904223630	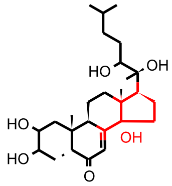 [*][C@H]1CC[C@@](O)(C(=[*])[*])C1([*])[*]	0.156	3 out of 3
FCFP_12	1002543125	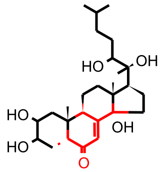 [*][C@@H]1[*][C@@H]([*])C(=CC1=O)C([*])([*])[*]	0.137	2 out of 2

Top Features for negative contribution

**Fingerprint**	**Bit/Smiles**	**Feature Structure**	**Score**	**Irritant in training set**
FCFP_12	-59531427	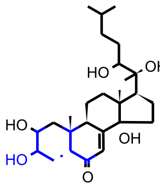 [*]C1[*]C([*])([*])[C@@H](CC1O)C(=[*])[*]	-0.156	4 out of 6
FCFP_12	-55265897	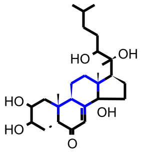 [*]C([*])([*])[C@H]1CCC([*])([*])[*]C1=[*]	-0.114	77 out of 107
FCFP_12	-1870530637	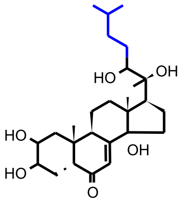 [*]CCC(C)C	-0.070	18 out of 24

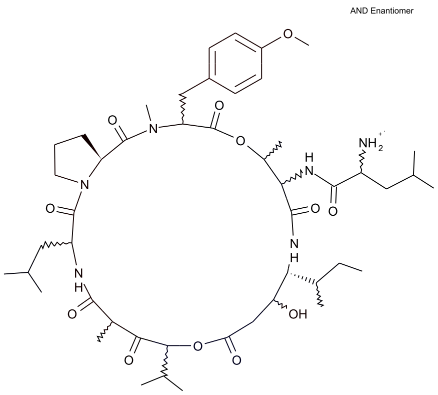

$C_{49}H_{79}N_6O_{12}$
Molecular Weight: 944.18455
ALogP: 3.176
Rotatable Bonds: 13
Acceptors: 12
Donors: 5

Model Prediction

Prediction: Irritant
Probability: 0.976
Enrichment: 1.147
Bayesian Score: 0.320
Mahalanobis Distance: 24.094
Mahalanobis Distance p-value: 7.42e-061

Prediction: Positive if the Bayesian score is above the estimated best cutoff value from minimizing the false positive and false negative rate.
Probability: The esimated probability that the sample is in the positive category. This assumes that the Bayesian score follows a normal distribution and is different from the prediction using a cutoff.
Enrichment: An estimate of enrichment, that is, the increased likelihood (versus random) of this sample being in the category.
Bayesian Score: The standard Laplacian-modified Bayesian score.
Mahalanobis Distance: The Mahalanobis distance (MD) is the distance to the center of the training data. The larger the MD, the less trustworthy the prediction.
Mahalanobis Distance p-value: The p-value gives the fraction of training data with an MD greater than or equal to the one for the given sample, assuming normally distributed data. The smaller the p-value, the less trustworthy the prediciton. For highly non-normal X properties (e.g., fingerprints), the MD p-value is wildly inaccurate.

Structural Similar Compounds

Name	2-Naphthalenesulfonic acid; 5;6'-iminobis(1-hydroxy-	2;7-NAPHTHALENE DISULFONIC ACID;4-AMINO-5-HYDROXY-;P-TOLUENE SULFONATE (ESTER)	2;7-Naphthalenedisulfonic acid;
Structure	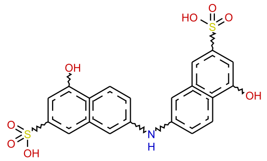	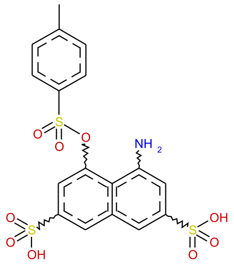	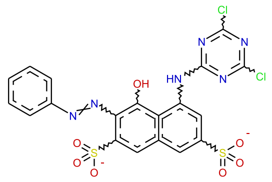
Actual Endpoint	Irritant	Irritant	Irritant
Predicted Endpoint	Irritant	Irritant	Irritant
Distance	1.404	1.491	1.501
Reference	Prehled Prumyslove Toxikologie; Organicke Latky; Marhold; J. pp 1065;86	28ZPAK-;194;72	Prehled Prumyslove Toxikologie; Organicke Latky; Marhold; J. - ;1300;86

Model Applicability

Unknown features are fingerprint features in the query molecule, but not found in the training set.

1. OPS PC15 out of range. Value: 4.7944. Training min, max, SD, explained variance: -4.342, 4.1881, 1.08, 0.0142.
2. Unknown FCFP_2 feature: 10: [*][NH2+][*]
3. Unknown FCFP_2 feature: 18028531: [*]C([*])[NH2+]C
4. Unknown FCFP_2 feature: -1817836174: [*]CC([NH2+][*])C(=[*])[*]
5. Unknown FCFP_2 feature: 136418580: [*][NH2+]C

Feature Contribution

Top features for positive contribution				
Fingerprint	Bit/Smiles	Feature Structure	Score	Irritant in training set

FCFP_12	-885550502	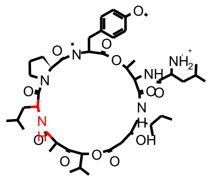 [*]C([*])NC(=[*])[*]	0.180	64 out of 66
FCFP_12	566058135	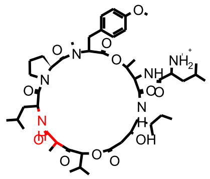 [*]NC(=O)C([*])[*]	0.163	23 out of 24
FCFP_12	-1553874037	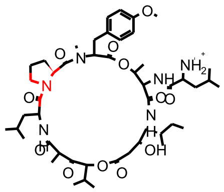 [*][C@@H]1[*][*]CN1C(=[*])[*]	0.163	42 out of 44

Top Features for negative contribution

Fingerprint	Bit/Smiles	Feature Structure	Score	Irritant in training set
FCFP_12	1213932708	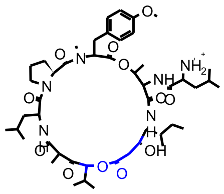 [*]C([*])CC(=O)OC([*])[*]	-0.671	28 out of 69
FCFP_12	-1580903393	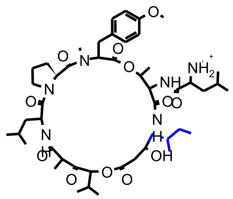 [*]C([*])C(C)CC	-0.284	35 out of 58

FCFP_12	-428284881	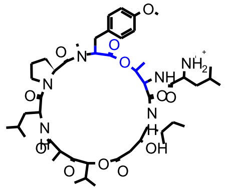<br>[*]C([*])C(OC(=O)C=[*<br>])C([*])[*]	-0.229	12 out of 19

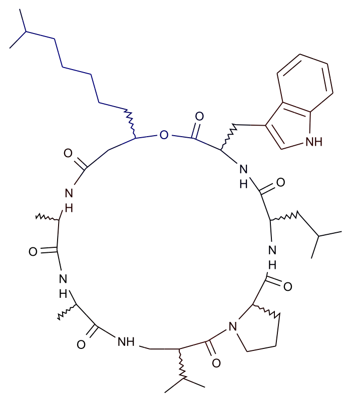

$C_{45}H_{69}N_7O_8$
Molecular Weight: 836.07145
ALogP: 4.986
Rotatable Bonds: 11
Acceptors: 8
Donors: 6

Model Prediction

Prediction: Non-Irritant
Probability: 0.969
Enrichment: 1.140
Bayesian Score: -1.238
Mahalanobis Distance: 21.131
Mahalanobis Distance p-value: 5.83e-043

Prediction: Positive if the Bayesian score is above the estimated best cutoff value from minimizing the false positive and false negative rate.
Probability: The esimated probability that the sample is in the positive category. This assumes that the Bayesian score follows a normal distribution and is different from the prediction using a cutoff.
Enrichment: An estimate of enrichment, that is, the increased likelihood (versus random) of this sample being in the category.
Bayesian Score: The standard Laplacian-modified Bayesian score.
Mahalanobis Distance: The Mahalanobis distance (MD) is the distance to the center of the training data. The larger the MD, the less trustworthy the prediction.
Mahalanobis Distance p-value: The p-value gives the fraction of training data with an MD greater than or equal to the one for the given sample, assuming normally distributed data. The smaller the p-value, the less trustworthy the prediciton. For highly non-normal X properties (e.g., fingerprints), the MD p-value is wildly inaccurate.

Structural Similar Compounds

Name	2-Naphthalenesulfonic acid; 5;6'-iminobis(1-hydroxy-	4;4'-DIAMINO-1;1'-DIANTHRIMIDE	ANTHRAQUINONE; 1;5-DIAMINO-4;8-DIHYDROXY-3-(p-METHOXYPHENYL)-
Structure	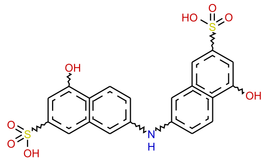	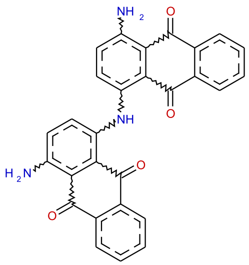	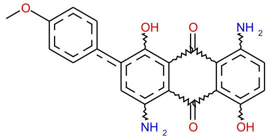
Actual Endpoint	Irritant	Irritant	Irritant
Predicted Endpoint	Irritant	Irritant	Irritant
Distance	1.247	1.451	1.454
Reference	Prehled Prumyslove Toxikologie; Organicke Latky; Marhold; J. pp 1065;86	28ZPAK-;125;72	28ZPAK 245;72

Model Applicability

Unknown features are fingerprint features in the query molecule, but not found in the training set.

1. All properties and OPS components are within expected ranges.

Feature Contribution

Top features for positive contribution

Fingerprint	Bit/Smiles	Feature Structure	Score	Irritant in training set
FCFP_12	-16162548	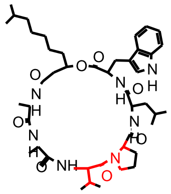 [*]CC(C([*])[*])C(=O)N1C[*][*]C1[*]	0.187	8 out of 8

FCFP_12	-885550502	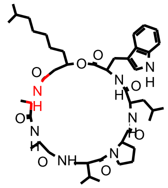 [*]C([*])NC(=[*])[*]	0.180	64 out of 66
FCFP_12	-416918913	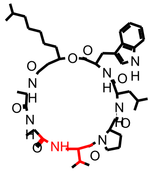 [*]C([*])C(CNC(=[*])[*])C(=[*])[*]	0.175	5 out of 5

Top Features for negative contribution

Fingerprint	Bit/Smiles	Feature Structure	Score	Irritant in training set
FCFP_12	-631749136	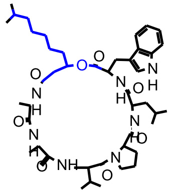 [*]C([*])CCCCCC(CC(=[*])[*])OC(=[*])[*]	-0.961	0 out of 2
FCFP_12	392412010	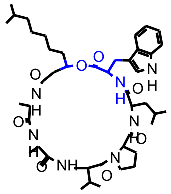 [*]CC(N[*])C(=O)OC([*])[*]	-0.537	1 out of 3
FCFP_12	-154166589	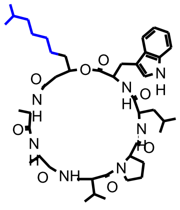 [*]CCCCC(C)C	-0.344	2 out of 4

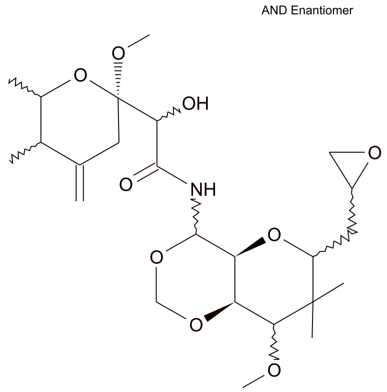

$C_{24}H_{39}NO_9$
Molecular Weight: 485.56776
ALogP: 0.267
Rotatable Bonds: 7
Acceptors: 9
Donors: 2

Structural Similar Compounds

Name	Fumaric acid; bis(3-allyloxy-2-hydroxypropyl) ester	COLCHICINE	2H-Naphtho(1;2-d)triazole-6;8-disulfonic acid;
Structure	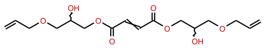	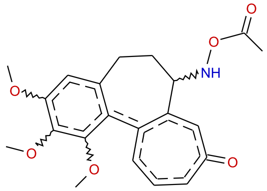	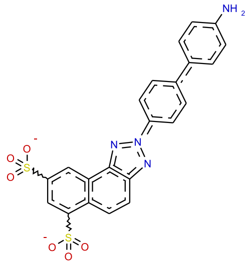
Actual Endpoint	Irritant	Irritant	Irritant
Predicted Endpoint	Irritant	Irritant	Irritant
Distance	0.818	0.858	0.879
Reference	Prehled Prumyslove Toxikologie; Organicke Latky; Marhold; J. -;646;86	AJOPAA 31;837;48	Prehled Prumyslove Toxikologie; Organicke Latky; Marhold; J. pp 1065;86

Model Prediction

Prediction: Irritant
Probability: 1.000
Enrichment: 1.176
Bayesian Score: 1.658
Mahalanobis Distance: 14.637
Mahalanobis Distance p-value: 1.73e-012

Prediction: Positive if the Bayesian score is above the estimated best cutoff value from minimizing the false positive and false negative rate.
Probability: The esimated probability that the sample is in the positive category. This assumes that the Bayesian score follows a normal distribution and is different from the prediction using a cutoff.
Enrichment: An estimate of enrichment, that is, the increased likelihood (versus random) of this sample being in the category.
Bayesian Score: The standard Laplacian-modified Bayesian score.
Mahalanobis Distance: The Mahalanobis distance (MD) is the distance to the center of the training data. The larger the MD, the less trustworthy the prediction.
Mahalanobis Distance p-value: The p-value gives the fraction of training data with an MD greater than or equal to the one for the given sample, assuming normally distributed data. The smaller the p-value, the less trustworthy the prediciton. For highly non-normal X properties (e.g., fingerprints), the MD p-value is wildly inaccurate.

Model Applicability

Unknown features are fingerprint features in the query molecule, but not found in the training set.

1. All properties and OPS components are within expected ranges.

Feature Contribution

Top features for positive contribution

Fingerprint	Bit/Smiles	Feature Structure	Score	Irritant in training set
FCFP_12	470041467	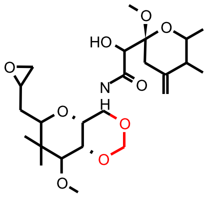 [*]OCO[*]	0.187	8 out of 8

FCFP_12	346066116	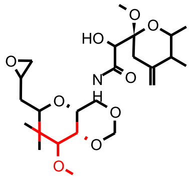<br>[*]C([*])C(OC)C([*])(<br>[*])[*]	0.187	8 out of 8
FCFP_12	648139160	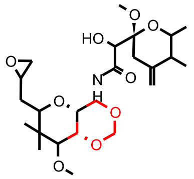<br>[*][C@@H]1[*]C([*])OC<br>O1	0.180	6 out of 6
**Top Features for negative contribution**				
**Fingerprint**	**Bit/Smiles**	**Feature Structure**	**Score**	**Irritant in training set**
FCFP_12	1327594201	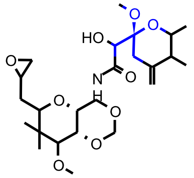<br>[*]CC(O[*])(O[*])C([*<br>])[*]	-0.140	9 out of 13
FCFP_12	-1682848062	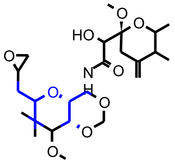<br>[*]CC1O[C@H](C([*])[*<br>])[C@H]([*])[*]C1([*<br>])[*]	-0.086	14 out of 19
FCFP_12	136597326	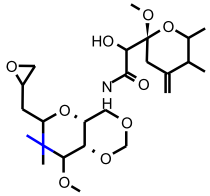<br>[*]C([*])C	0.000	612 out of 753

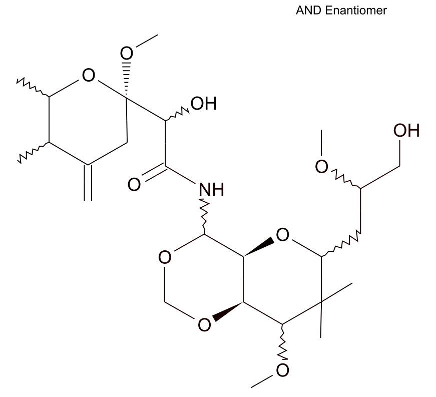

$C_{25}H_{43}NO_{10}$
Molecular Weight: 517.60962
ALogP: -9.1e-002
Rotatable Bonds: 9
Acceptors: 10
Donors: 3

Model Prediction

Prediction: Irritant
Probability: 1.000
Enrichment: 1.176
Bayesian Score: 1.690
Mahalanobis Distance: 15.146
Mahalanobis Distance p-value: 2.57e-014

Prediction: Positive if the Bayesian score is above the estimated best cutoff value from minimizing the false positive and false negative rate.
Probability: The esimated probability that the sample is in the positive category. This assumes that the Bayesian score follows a normal distribution and is different from the prediction using a cutoff.
Enrichment: An estimate of enrichment, that is, the increased likelihood (versus random) of this sample being in the category.
Bayesian Score: The standard Laplacian-modified Bayesian score.
Mahalanobis Distance: The Mahalanobis distance (MD) is the distance to the center of the training data. The larger the MD, the less trustworthy the prediction.
Mahalanobis Distance p-value: The p-value gives the fraction of training data with an MD greater than or equal to the one for the given sample, assuming normally distributed data. The smaller the p-value, the less trustworthy the prediciton. For highly non-normal X properties (e.g., fingerprints), the MD p-value is wildly inaccurate.

Structural Similar Compounds

Name	Fumaric acid; bis(3-allyloxy-2-hydroxypropyl) ester	2;7-NAPHTHALENE DISULFONIC ACID;4-AMINO-5-HYDROXY-;P-TOLUENE SULFONATE (ESTER)	4;4'-DIAMINO-1;1'-DIANTHRIMIDE
Structure	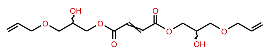	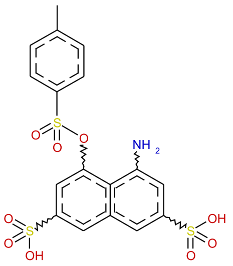	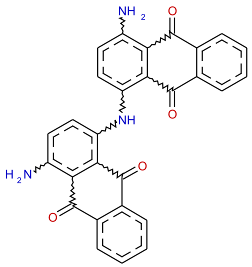
Actual Endpoint	Irritant	Irritant	Irritant
Predicted Endpoint	Irritant	Irritant	Irritant
Distance	0.924	0.929	0.985
Reference	Prehled Prumyslove Toxikologie; Organicke Latky; Marhold; J. -;646;86	28ZPAK-;194;72	28ZPAK-;125;72

Model Applicability

Unknown features are fingerprint features in the query molecule, but not found in the training set.

1. All properties and OPS components are within expected ranges.

Feature Contribution

Top features for positive contribution				
Fingerprint	Bit/Smiles	Feature Structure	Score	Irritant in training set
FCFP_12	-2091721556	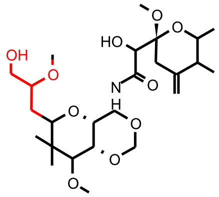 [*]CC(CO)O[*]	0.187	8 out of 8

FCFP_12	470041467	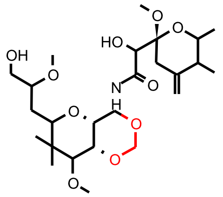 [*]OCO[*]	0.187	8 out of 8
FCFP_12	346066116	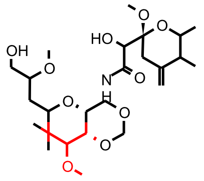 [*]C([*])C(OC)C([*])([*])[*]	0.187	8 out of 8
**Top Features for negative contribution**				
**Fingerprint**	**Bit/Smiles**	**Feature Structure**	**Score**	**Irritant in training set**
FCFP_12	1327594201	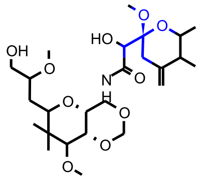 [*]CC(O[*])(O[*])C([*])[*]	-0.140	9 out of 13
FCFP_12	-1682848062	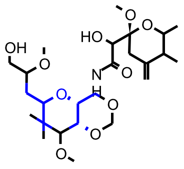 [*]CC1O[C@H](C([*])[*])[C@H]([*])[*]C1([*])[*]	-0.086	14 out of 19
FCFP_12	1	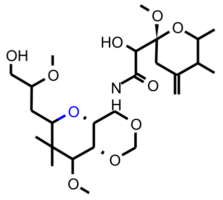 [*]O[*]	0.000	872 out of 1051

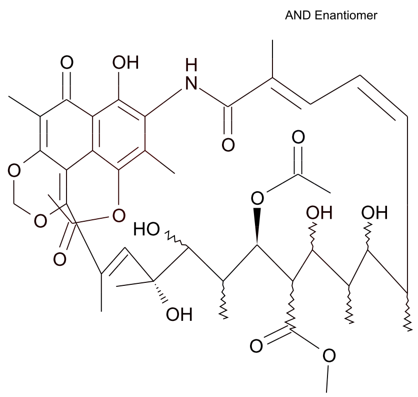

$C_{42}H_{53}NO_{15}$

Molecular Weight: 811.86791

ALogP: 2.745

Rotatable Bonds: 6

Acceptors: 15

Donors: 6

Model Prediction

Prediction: Irritant

Probability: 1.000

Enrichment: 1.176

Bayesian Score: 3.446

Mahalanobis Distance: 20.122

Mahalanobis Distance p-value: 2.44e-037

Prediction: Positive if the Bayesian score is above the estimated best cutoff value from minimizing the false positive and false negative rate.
Probability: The esimated probability that the sample is in the positive category. This assumes that the Bayesian score follows a normal distribution and is different from the prediction using a cutoff.
Enrichment: An estimate of enrichment, that is, the increased likelihood (versus random) of this sample being in the category.
Bayesian Score: The standard Laplacian-modified Bayesian score.
Mahalanobis Distance: The Mahalanobis distance (MD) is the distance to the center of the training data. The larger the MD, the less trustworthy the prediction.
Mahalanobis Distance p-value: The p-value gives the fraction of training data with an MD greater than or equal to the one for the given sample, assuming normally distributed data. The smaller the p-value, the less trustworthy the prediciton. For highly non-normal X properties (e.g., fingerprints), the MD p-value is wildly inaccurate.

Structural Similar Compounds

Name	2-Naphthalenesulfonic acid; 5;6'-iminobis(1-hydroxy-	Methanol; (s-triazine-2;4;6-triyltrinitrilo)hexa-	2;7-NAPHTHALENE DISULFONIC ACID;4-AMINO-5-HYDROXY-;P-TOLUENE SULFONATE (ESTER)
Structure	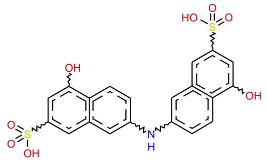	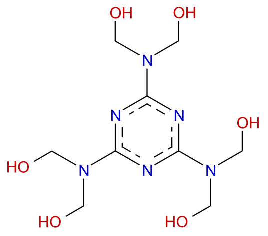	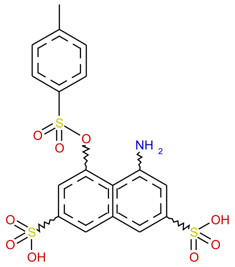
Actual Endpoint	Irritant	Irritant	Irritant
Predicted Endpoint	Irritant	Irritant	Irritant
Distance	1.329	1.480	1.511
Reference	Prehled Prumyslove Toxikologie; Organicke Latky; Marhold; J. pp 1065;86	Prehled Prumyslove Toxikologie; Organicke Latky; Marhold; J. -;876;86	28ZPAK-;194;72

Model Applicability

Unknown features are fingerprint features in the query molecule, but not found in the training set.

1. OPS PC7 out of range. Value: 6.0687. Training min, max, SD, explained variance: -6.07, 6.0201, 1.57, 0.0301.

Feature Contribution

Top features for positive contribution

Fingerprint	Bit/Smiles	Feature Structure	Score	Irritant in training set
FCFP_12	1175665944	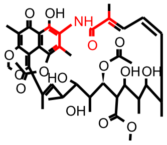 [*]C(=[*])C(=O)N[c](:[c]([*]):[*]):[c]([*]):[*]	0.198	14 out of 14

FCFP_12	-1979033238	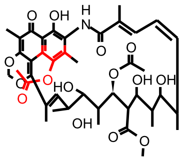 [*][c](:[*]):[c](OC(=O)C):[c]([*]):[*]	0.192	10 out of 10
FCFP_12	-1539730963	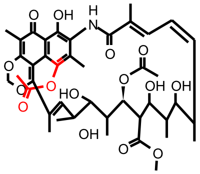 [*]:[c](:[*])OC(=O)C	0.190	9 out of 9

Top Features for negative contribution

Fingerprint	Bit/Smiles	Feature Structure	Score	Irritant in training set
FCFP_12	-428284881	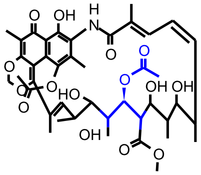 [*]C([*])C(OC(=O)C=[*])C([*])[*]	-0.229	12 out of 19
FCFP_12	565998553	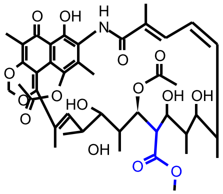 [*]OC(=O)C=[*]	-0.066	198 out of 262
FCFP_12	-1678275541	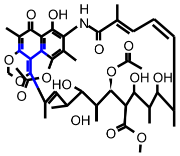 [*]C(=C(C(=[*])[*])[c](:[*]):[*])[*]	-0.056	3 out of 4

Simeprevir

TOPKAT_Ocular_Irritancy_None_vs_Irritant

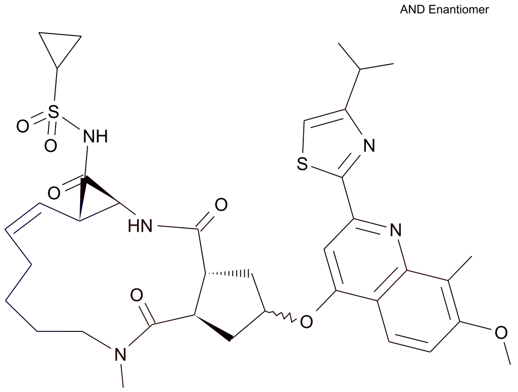

$C_{38}H_{47}N_5O_7S_2$

Molecular Weight: 749.93907

ALogP: 4.796

Rotatable Bonds: 8

Acceptors: 9

Donors: 2

Structural Similar Compounds

Name	Anthraquinone; 1;1'-(anthraquinon-1;5-ylenediimino)di-	Anthraquinone; 1;1'-(anthraquinon-1;4-ylenediimino)di-	ANILINE;N;N'-1;4-ANTHRAQUINONYLENEBIS(4-PHENOXY-
Structure	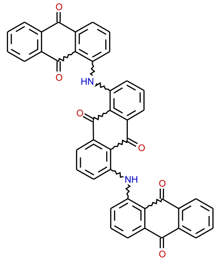	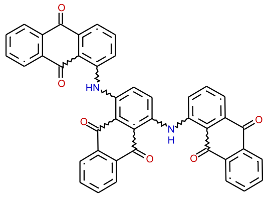	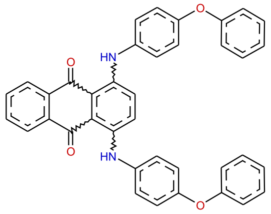
Actual Endpoint	Irritant	Irritant	Non-Irritant
Predicted Endpoint	Irritant	Irritant	Non-Irritant
Distance	0.837	0.838	1.053
Reference	Prehled Prumyslove Toxikologie; Organicke Latky; Marhold; J. pp 736;86	Prehled Prumyslove Toxikologie; Organicke Latky; Marhold; J. pp 736;86	28ZPAK-;114;72

Model Prediction

Prediction: Irritant

Probability: 1.000

Enrichment: 1.176

Bayesian Score: 1.806

Mahalanobis Distance: 16.456

Mahalanobis Distance p-value: 1.62e-019

Prediction: Positive if the Bayesian score is above the estimated best cutoff value from minimizing the false positive and false negative rate.
Probability: The esimated probability that the sample is in the positive category. This assumes that the Bayesian score follows a normal distribution and is different from the prediction using a cutoff.
Enrichment: An estimate of enrichment, that is, the increased likelihood (versus random) of this sample being in the category.
Bayesian Score: The standard Laplacian-modified Bayesian score.
Mahalanobis Distance: The Mahalanobis distance (MD) is the distance to the center of the training data. The larger the MD, the less trustworthy the prediction.
Mahalanobis Distance p-value: The p-value gives the fraction of training data with an MD greater than or equal to the one for the given sample, assuming normally distributed data. The smaller the p-value, the less trustworthy the prediciton. For highly non-normal X properties (e.g., fingerprints), the MD p-value is wildly inaccurate.

Model Applicability

Unknown features are fingerprint features in the query molecule, but not found in the training set.

1. All properties and OPS components are within expected ranges.

Feature Contribution

Top features for positive contribution

Fingerprint	Bit/Smiles	Feature Structure	Score	Irritant in training set
FCFP_12	1747237384	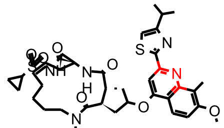 [*][c](:[*]):n:[c](:[*]):[*]	0.208	44 out of 44

FCFP_12	178336375	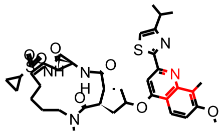 [*][c](:[*]):[c](:n:[*]):[c](:[*]):[*]	0.202	19 out of 19
FCFP_12	-124655670	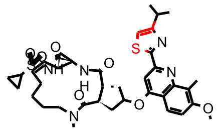 [*][c]1:[*]:[*]:s:[cH]:1	0.200	16 out of 16
**Top Features for negative contribution**				
**Fingerprint**	**Bit/Smiles**	**Feature Structure**	**Score**	**Irritant in training set**
FCFP_12	-1812846456	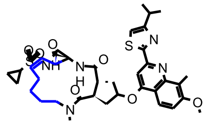 [*]CCCC\C=C/C1[*][*]1	-1.014	2 out of 9
FCFP_12	690511177	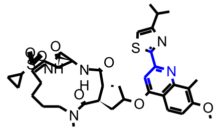 [*]:[cH]:[c](:n:[*])[c](:[*]):[*]	-0.268	1 out of 2
FCFP_12	1175638033	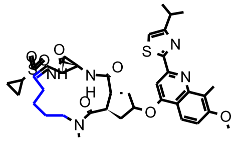 [*]CCCCC([*])[*]	-0.133	207 out of 293

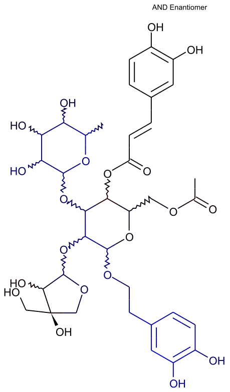

$C_{36}H_{46}O_{20}$
Molecular Weight: 798.73843
ALogP: -0.546
Rotatable Bonds: 16
Acceptors: 20
Donors: 10

Model Prediction

Prediction: Non-Carcinogen
Probability: 0.180
Enrichment: 0.559
Bayesian Score: -10.360
Mahalanobis Distance: 19.004
Mahalanobis Distance p-value: 1.85e-018

Prediction: Positive if the Bayesian score is above the estimated best cutoff value from minimizing the false positive and false negative rate.
Probability: The esimated probability that the sample is in the positive category. This assumes that the Bayesian score follows a normal distribution and is different from the prediction using a cutoff.
Enrichment: An estimate of enrichment, that is, the increased likelihood (versus random) of this sample being in the category.
Bayesian Score: The standard Laplacian-modified Bayesian score.
Mahalanobis Distance: The Mahalanobis distance (MD) is the distance to the center of the training data. The larger the MD, the less trustworthy the prediction.
Mahalanobis Distance p-value: The p-value gives the fraction of training data with an MD greater than or equal to the one for the given sample, assuming normally distributed data. The smaller the p-value, the less trustworthy the prediciton. For highly non-normal X properties (e.g., fingerprints), the MD p-value is wildly inaccurate.

Structural Similar Compounds

Name	Rifampin	Natamycin	Netilmicin
Structure	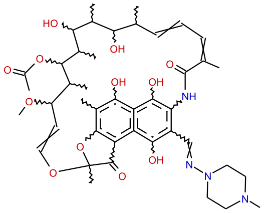	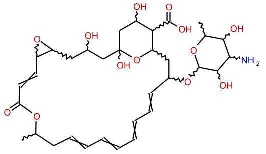	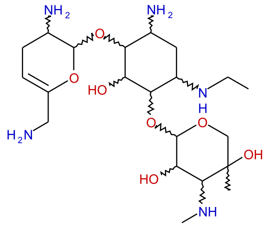
Actual Endpoint	Non-Carcinogen	Non-Carcinogen	Non-Carcinogen
Predicted Endpoint	Non-Carcinogen	Non-Carcinogen	Non-Carcinogen
Distance	1.538	1.559	1.576
Reference	US FDA (Centre for Drug Eval.& Res./Off. Testing & Res.) Sept. 1997	US FDA (Centre for Drug Eval.& Res./Off. Testing & Res.) Sept. 1997	US FDA (Centre for Drug Eval.& Res./Off. Testing & Res.) Sept. 1997

Model Applicability

Unknown features are fingerprint features in the query molecule, but not found in the training set.

1. Num_H_Acceptors out of range. Value: 20. Training min, max, mean, SD: 0, 15, 4.2304, 2.314.
2. OPS PC3 out of range. Value: 5.9163. Training min, max, SD, explained variance: -7.6052, 5.6498, 2.401, 0.0605.
3. OPS PC4 out of range. Value: 5.1678. Training min, max, SD, explained variance: -5.3573, 4.9476, 2.055, 0.0443.
4. Unknown ECFP_2 feature: 1953003528: [*]C[C@@]1(O)C[*][*]C1[*]
5. Unknown ECFP_2 feature: -176483725: [*]=C[c](:c:[*]):c:[*]

Feature Contribution

Top features for positive contribution				
Fingerprint	Bit/Smiles	Feature Structure	Score	Carcinogen in training set

ECFP_12	-553149446	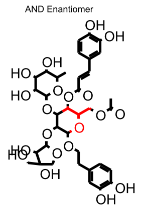<br>[*]CC(O[*])C([*])[*]	0.575	3 out of 4
ECFP_12	-521596699	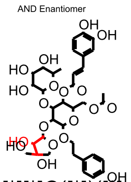<br>[*]C1[*][*]C([*])([*])C1O	0.445	3 out of 5
ECFP_12	-1925046727	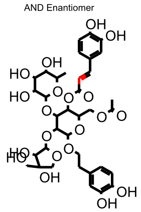<br>[*]C=[*]	0.407	16 out of 33
**Top Features for negative contribution**				
**Fingerprint**	**Bit/Smiles**	**Feature Structure**	**Score**	**Carcinogen in training set**
ECFP_12	-2060414325	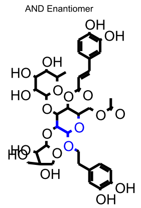<br>[*]OC(O[*])C([*])[*]	-0.811	0 out of 4
ECFP_12	456242574	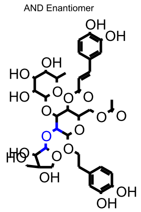<br>[*]C([*])OC([*])[*]	-0.811	0 out of 4

ECFP_12	-770374390	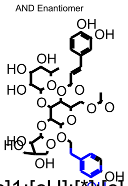 [*]C[c]1:[cH]:[*]:[c]([*]):[c](O):[cH]:1	-0.661	0 out of 3

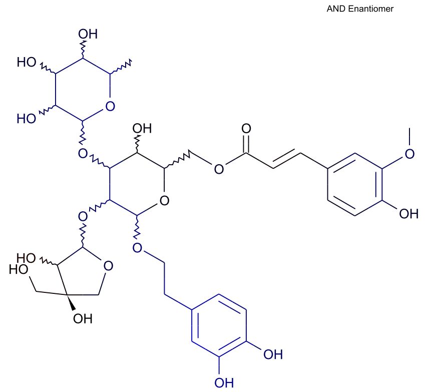

$C_{35}H_{46}O_{19}$
Molecular Weight: 770.72833
ALogP: -0.699
Rotatable Bonds: 15
Acceptors: 19
Donors: 10

Model Prediction

Prediction: Non-Carcinogen
Probability: 0.171
Enrichment: 0.532
Bayesian Score: -12.945
Mahalanobis Distance: 19.251
Mahalanobis Distance p-value: 3.88e-019

Prediction: Positive if the Bayesian score is above the estimated best cutoff value from minimizing the false positive and false negative rate.
Probability: The esimated probability that the sample is in the positive category. This assumes that the Bayesian score follows a normal distribution and is different from the prediction using a cutoff.
Enrichment: An estimate of enrichment, that is, the increased likelihood (versus random) of this sample being in the category.
Bayesian Score: The standard Laplacian-modified Bayesian score.
Mahalanobis Distance: The Mahalanobis distance (MD) is the distance to the center of the training data. The larger the MD, the less trustworthy the prediction.
Mahalanobis Distance p-value: The p-value gives the fraction of training data with an MD greater than or equal to the one for the given sample, assuming normally distributed data. The smaller the p-value, the less trustworthy the prediciton. For highly non-normal X properties (e.g., fingerprints), the MD p-value is wildly inaccurate.

Structural Similar Compounds

Name	Natamycin	Netilmicin	Rifampin
Structure	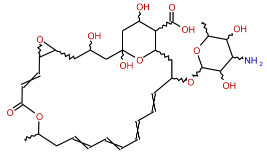	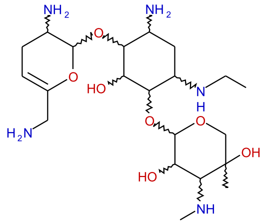	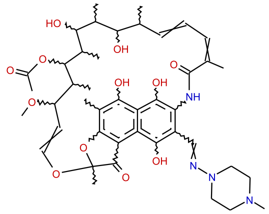
Actual Endpoint	Non-Carcinogen	Non-Carcinogen	Non-Carcinogen
Predicted Endpoint	Non-Carcinogen	Non-Carcinogen	Non-Carcinogen
Distance	1.460	1.466	1.475
Reference	US FDA (Centre for Drug Eval.& Res./Off. Testing & Res.) Sept. 1997	US FDA (Centre for Drug Eval.& Res./Off. Testing & Res.) Sept. 1997	US FDA (Centre for Drug Eval.& Res./Off. Testing & Res.) Sept. 1997

Model Applicability

Unknown features are fingerprint features in the query molecule, but not found in the training set.

1. Num_H_Acceptors out of range. Value: 19. Training min, max, mean, SD: 0, 15, 4.2304, 2.314.
2. OPS PC3 out of range. Value: 5.741. Training min, max, SD, explained variance: -7.6052, 5.6498, 2.401, 0.0605.
3. OPS PC4 out of range. Value: 5.2611. Training min, max, SD, explained variance: -5.3573, 4.9476, 2.055, 0.0443.
4. OPS PC13 out of range. Value: -3.0474. Training min, max, SD, explained variance: -2.9848, 6.2909, 1.429, 0.0214.
5. Unknown ECFP_2 feature: 1953003528: [*]C[C@@]1(O)C[*][*]C1[*]
6. Unknown ECFP_2 feature: -176483725: [*]=C[c](:c:[*]):c:[*]

Feature Contribution

Top features for positive contribution				
**Fingerprint**	**Bit/Smiles**	**Feature Structure**	**Score**	**Carcinogen in training set**

ECFP_12	-553149446	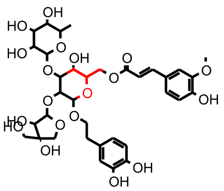<br>[*]CC(O[*])C([*])[*]	0.575	3 out of 4
ECFP_12	-521596699	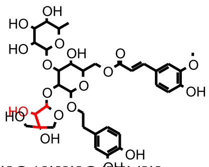<br>[*]C1[*][*]C([*])([*])C1O	0.445	3 out of 5
ECFP_12	-1925046727	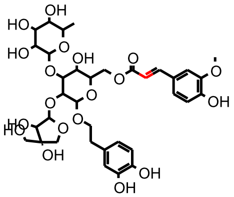<br>[*]C=[*]	0.407	16 out of 33

Top Features for negative contribution

Fingerprint	Bit/Smiles	Feature Structure	Score	Carcinogen in training set
ECFP_12	-2060414325	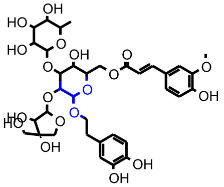<br>[*]OC(O[*])C([*])[*]	-0.811	0 out of 4
ECFP_12	456242574	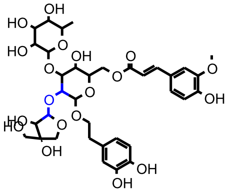<br>[*]C([*])OC([*])[*]	-0.811	0 out of 4

ECFP_12	-770374390	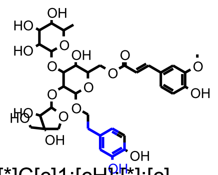 [*]C[c]1:[cH]:[*]:[c]([*]):[c](O):[cH]:1	-0.661	0 out of 3

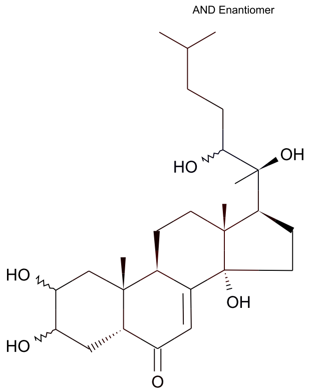

$C_{27}H_{44}O_6$
Molecular Weight: 464.63466
ALogP: 2.489
Rotatable Bonds: 5
Acceptors: 6
Donors: 5

Model Prediction

Prediction: Carcinogen
Probability: 0.335
Enrichment: 1.041
Bayesian Score: 1.667
Mahalanobis Distance: 15.692
Mahalanobis Distance p-value: 5.3e-010

Prediction: Positive if the Bayesian score is above the estimated best cutoff value from minimizing the false positive and false negative rate.
Probability: The esimated probability that the sample is in the positive category. This assumes that the Bayesian score follows a normal distribution and is different from the prediction using a cutoff.
Enrichment: An estimate of enrichment, that is, the increased likelihood (versus random) of this sample being in the category.
Bayesian Score: The standard Laplacian-modified Bayesian score.
Mahalanobis Distance: The Mahalanobis distance (MD) is the distance to the center of the training data. The larger the MD, the less trustworthy the prediction.
Mahalanobis Distance p-value: The p-value gives the fraction of training data with an MD greater than or equal to the one for the given sample, assuming normally distributed data. The smaller the p-value, the less trustworthy the prediciton. For highly non-normal X properties (e.g., fingerprints), the MD p-value is wildly inaccurate.

Structural Similar Compounds

Name	Triamcinolone	Mesuprine	Budesonide
Structure	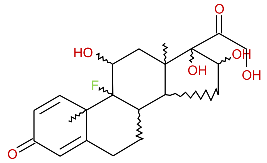	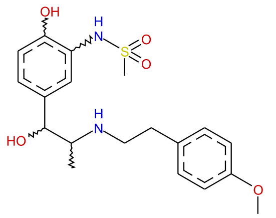	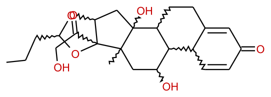
Actual Endpoint	Non-Carcinogen	Carcinogen	Non-Carcinogen
Predicted Endpoint	Non-Carcinogen	Carcinogen	Non-Carcinogen
Distance	0.789	0.798	0.808
Reference	US FDA (Centre for Drug Eval.& Res./Off. Testing & Res.) Sept. 1997	US FDA (Centre for Drug Eval.& Res./Off. Testing & Res.) Sept. 1997	US FDA (Centre for Drug Eval.& Res./Off. Testing & Res.) Sept. 1997

Model Applicability

Unknown features are fingerprint features in the query molecule, but not found in the training set.

1. All properties and OPS components are within expected ranges.
2. Unknown ECFP_2 feature: -483166673: [*]C(=[*])[C@]1(O)C[*][*]C1([*])[*]
3. Unknown ECFP_2 feature: -1049278438: [*]C([*])([*])[C@H]1C[*][*]C1([*])[*]
4. Unknown ECFP_2 feature: 1195714366: [*]C([*])C(C)(O)C([*])[*]
5. Unknown ECFP_2 feature: -211745668: [*]C([*])C(=O)C=[*]
6. Unknown ECFP_2 feature: 80071435: [*]CC(O)C([*])([*])[*]

Feature Contribution

Top features for positive contribution

Fingerprint	Bit/Smiles	Feature Structure	Score	Carcinogen in training set
ECFP_12	1035165602	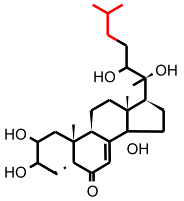 [*]CC(C)C	0.575	3 out of 4

ECFP_12	-84975114	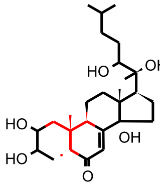<br>[*]CC(C)(C([*])[*])C([*])[*]	0.539	5 out of 8
ECFP_12	1908695621	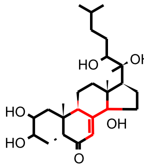<br>[*]\C=C(/C([*])[*])\C([*])([*])[*]	0.421	1 out of 1

Top Features for negative contribution

Fingerprint	Bit/Smiles	Feature Structure	Score	Carcinogen in training set
ECFP_12	333674688	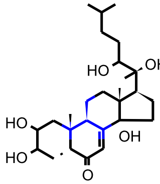<br>[*]C[C@@H](C(=[*])[*])C([*])([*])[*]	-0.272	0 out of 1
ECFP_12	2023785560	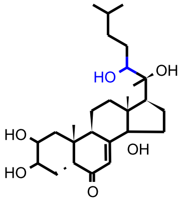<br>[*]C([*])O	-0.253	9 out of 38
ECFP_12	-1910270391	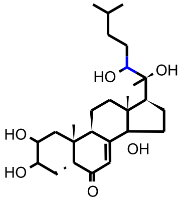<br>[*]C([*])[*]	-0.216	26 out of 104

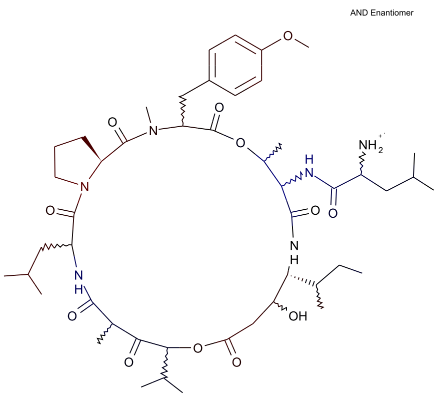

$C_{49}H_{79}N_6O_{12}$

Molecular Weight: 944.18455

ALogP: 3.176

Rotatable Bonds: 13

Acceptors: 12

Donors: 5

Model Prediction

Prediction: Non-Carcinogen

Probability: 0.253

Enrichment: 0.784

Bayesian Score: -2.607

Mahalanobis Distance: 16.345

Mahalanobis Distance p-value: 1.49e-011

Prediction: Positive if the Bayesian score is above the estimated best cutoff value from minimizing the false positive and false negative rate.
Probability: The esimated probability that the sample is in the positive category. This assumes that the Bayesian score follows a normal distribution and is different from the prediction using a cutoff.
Enrichment: An estimate of enrichment, that is, the increased likelihood (versus random) of this sample being in the category.
Bayesian Score: The standard Laplacian-modified Bayesian score.
Mahalanobis Distance: The Mahalanobis distance (MD) is the distance to the center of the training data. The larger the MD, the less trustworthy the prediction.
Mahalanobis Distance p-value: The p-value gives the fraction of training data with an MD greater than or equal to the one for the given sample, assuming normally distributed data. The smaller the p-value, the less trustworthy the prediciton. For highly non-normal X properties (e.g., fingerprints), the MD p-value is wildly inaccurate.

Structural Similar Compounds

Name	Erythromycin	Rifabutin	Rifampin
Structure	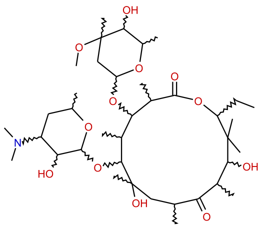	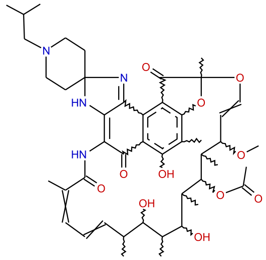	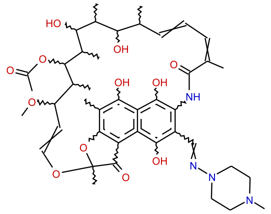
Actual Endpoint	Non-Carcinogen	Non-Carcinogen	Non-Carcinogen
Predicted Endpoint	Non-Carcinogen	Non-Carcinogen	Non-Carcinogen
Distance	1.025	1.034	1.090
Reference	US FDA (Centre for Drug Eval.& Res./Off. Testing & Res.) Sept. 1997	US FDA (Centre for Drug Eval.& Res./Off. Testing & Res.) Sept. 1997	US FDA (Centre for Drug Eval.& Res./Off. Testing & Res.) Sept. 1997

Model Applicability

Unknown features are fingerprint features in the query molecule, but not found in the training set.

1. Molecular_Weight out of range. Value: 944.18. Training min, max, mean, SD: 46.068, 847, 306.62, 116.4.
2. Unknown ECFP_2 feature: -591526139: [*][NH2+][*]
3. Unknown ECFP_2 feature: 1352327988: [*]C([*])[NH2+]C
4. Unknown ECFP_2 feature: 1691770380: [*]NC(C([*])[*])C([*])[*]
5. Unknown ECFP_2 feature: 771121623: [*]C([*])C[c](:[*]):[*]
6. Unknown ECFP_2 feature: -714938792: [*]C(=[*])C(C)C(=[*])[*]
7. Unknown ECFP_2 feature: 20550775: [*]CC([NH2+][*])C(=[*])[*]
8. Unknown ECFP_2 feature: 866401773: [*][NH2+]C

Feature Contribution

Top features for positive contribution				
**Fingerprint**	**Bit/Smiles**	**Feature Structure**	**Score**	**Carcinogen in training set**

ECFP_12	1038798692	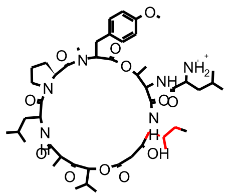 [*]CC(C)C([*])[*]	0.613	2 out of 2
ECFP_12	1035165602	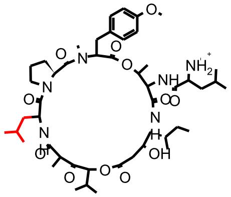 [*]CC(C)C	0.575	3 out of 4
ECFP_12	53207596	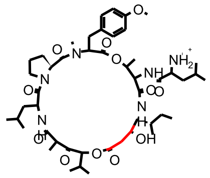 [*]C([*])CC(=[*])[*]	0.459	8 out of 15
**Top Features for negative contribution**				
**Fingerprint**	**Bit/Smiles**	**Feature Structure**	**Score**	**Carcinogen in training set**
ECFP_12	-1567199489	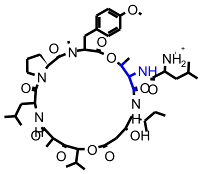 [*]NC(C([*])[*])C(=[*])[*]	-0.811	0 out of 4
ECFP_12	-949992060	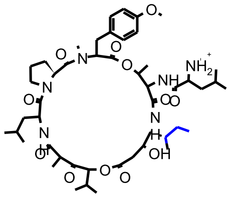 [*]C([*])CC	-0.811	0 out of 4

ECFP_12	-1693599735	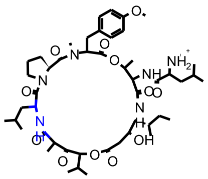<br>[*]C([*])NC(=[*])[*]	-0.661	0 out of 3

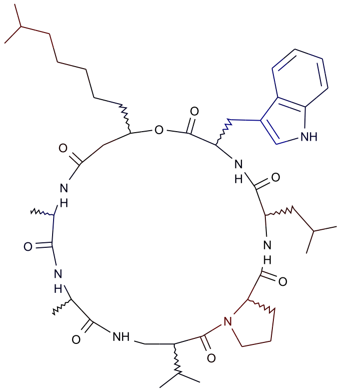

$C_{45}H_{69}N_{7}O_{8}$
Molecular Weight: 836.07145
ALogP: 4.986
Rotatable Bonds: 11
Acceptors: 8
Donors: 6

Model Prediction

Prediction: Carcinogen
Probability: 0.305
Enrichment: 0.949
Bayesian Score: 0.338
Mahalanobis Distance: 14.665
Mahalanobis Distance p-value: 1e-007

Prediction: Positive if the Bayesian score is above the estimated best cutoff value from minimizing the false positive and false negative rate.
Probability: The esimated probability that the sample is in the positive category. This assumes that the Bayesian score follows a normal distribution and is different from the prediction using a cutoff.
Enrichment: An estimate of enrichment, that is, the increased likelihood (versus random) of this sample being in the category.
Bayesian Score: The standard Laplacian-modified Bayesian score.
Mahalanobis Distance: The Mahalanobis distance (MD) is the distance to the center of the training data. The larger the MD, the less trustworthy the prediction.
Mahalanobis Distance p-value: The p-value gives the fraction of training data with an MD greater than or equal to the one for the given sample, assuming normally distributed data. The smaller the p-value, the less trustworthy the prediciton. For highly non-normal X properties (e.g., fingerprints), the MD p-value is wildly inaccurate.

Structural Similar Compounds

Name	Erythromycin	Bromocriptine	Rifabutin
Structure	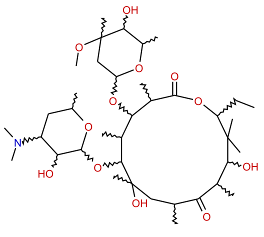	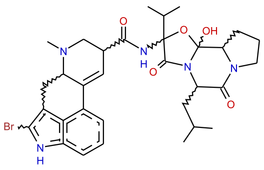	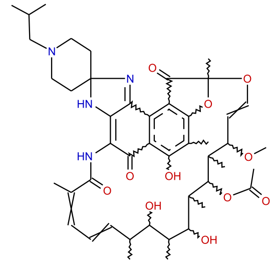
Actual Endpoint	Non-Carcinogen	Carcinogen	Non-Carcinogen
Predicted Endpoint	Non-Carcinogen	Carcinogen	Non-Carcinogen
Distance	1.120	1.132	1.144
Reference	US FDA (Centre for Drug Eval.& Res./Off. Testing & Res.) Sept. 1997	US FDA (Centre for Drug Eval.& Res./Off. Testing & Res.) Sept. 1997	US FDA (Centre for Drug Eval.& Res./Off. Testing & Res.) Sept. 1997

Model Applicability

Unknown features are fingerprint features in the query molecule, but not found in the training set.

1. All properties and OPS components are within expected ranges.
2. Unknown ECFP_2 feature: 771121623: [*]C([*])C[c](:[*]):[*]
3. Unknown ECFP_2 feature: 1863041499: [*]NC(C)C(=[*])[*]
4. Unknown ECFP_2 feature: -2096927833: [*]CC(C([*])[*])C(=[*])[*]

Feature Contribution

Top features for positive contribution				
Fingerprint	Bit/Smiles	Feature Structure	Score	Carcinogen in training set
ECFP_12	1035165602	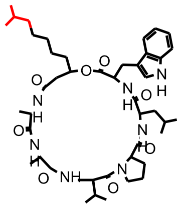 [*]CC(C)C	0.575	3 out of 4

ECFP_12	53207596	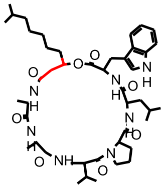 [*]C([*])CC(=[*])[*]	0.459	8 out of 15
ECFP_12	1099224616	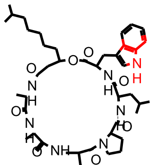 [*]:[cH]:[c]1:[nH]:[*]:[*]:[c]:1:[*]	0.456	6 out of 11
**Top Features for negative contribution**				
**Fingerprint**	**Bit/Smiles**	**Feature Structure**	**Score**	**Carcinogen in training set**
ECFP_12	-1659633832	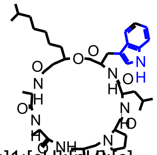 [*][c]1:[cH]:[nH]:[c]2:[cH]:[cH]:[*]:[cH]:[c]:1:2	-0.661	0 out of 3
ECFP_12	-1693599735	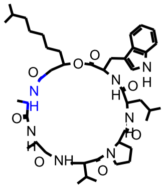 [*]C([*])NC(=[*])[*]	-0.661	0 out of 3
ECFP_12	-867777309	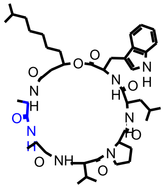 [*]NC(=O)C([*])[*]	-0.661	0 out of 3

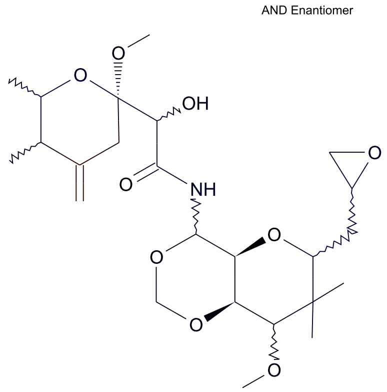

$C_{24}H_{39}NO_9$
Molecular Weight: 485.56776
ALogP: 0.267
Rotatable Bonds: 7
Acceptors: 9
Donors: 2

Structural Similar Compounds

Name	Bacampicillin	Lasiocarpine	Budesonide
Structure	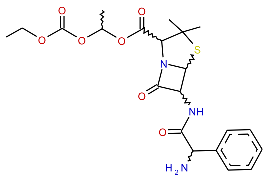	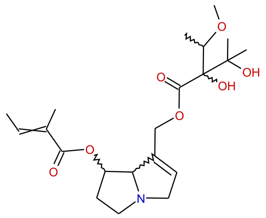	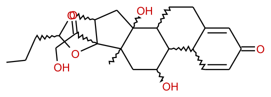
Actual Endpoint	Non-Carcinogen	Carcinogen	Non-Carcinogen
Predicted Endpoint	Non-Carcinogen	Carcinogen	Non-Carcinogen
Distance	0.728	0.735	0.784
Reference	US FDA (Centre for Drug Eval.& Res./Off. Testing & Res.) Sept. 1997	US FDA (Centre for Drug Eval.& Res./Off. Testing & Res.) Sept. 1997	US FDA (Centre for Drug Eval.& Res./Off. Testing & Res.) Sept. 1997

Model Prediction

Prediction: Non-Carcinogen
Probability: 0.215
Enrichment: 0.668
Bayesian Score: -5.561
Mahalanobis Distance: 14.930
Mahalanobis Distance p-value: 2.72e-008

Prediction: Positive if the Bayesian score is above the estimated best cutoff value from minimizing the false positive and false negative rate.
Probability: The esimated probability that the sample is in the positive category. This assumes that the Bayesian score follows a normal distribution and is different from the prediction using a cutoff.
Enrichment: An estimate of enrichment, that is, the increased likelihood (versus random) of this sample being in the category.
Bayesian Score: The standard Laplacian-modified Bayesian score.
Mahalanobis Distance: The Mahalanobis distance (MD) is the distance to the center of the training data. The larger the MD, the less trustworthy the prediction.
Mahalanobis Distance p-value: The p-value gives the fraction of training data with an MD greater than or equal to the one for the given sample, assuming normally distributed data. The smaller the p-value, the less trustworthy the prediciton. For highly non-normal X properties (e.g., fingerprints), the MD p-value is wildly inaccurate.

Model Applicability

Unknown features are fingerprint features in the query molecule, but not found in the training set.

1. All properties and OPS components are within expected ranges.
2. Unknown ECFP_2 feature: -457637478: [*]NC(O[*])C([*])[*]
3. Unknown ECFP_2 feature: 837844420: [*]CC(O[*])(O[*])C([*])[*]
4. Unknown ECFP_2 feature: 825070174: [*]C(=[*])C(O)C([*])([*])[*]

Feature Contribution

Top features for positive contribution				
**Fingerprint**	**Bit/Smiles**	**Feature Structure**	**Score**	**Carcinogen in training set**
ECFP_12	-2085781978	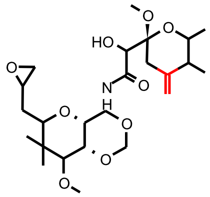 [*]C(=C)[*]	0.613	2 out of 2

ECFP_12	1377749300	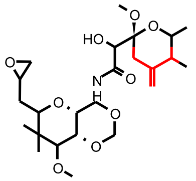<br>[*]CC(=C)C([*])[*]	0.421	1 out of 1
ECFP_12	683445015	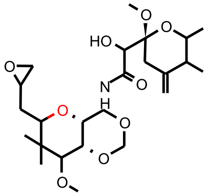<br>[*]O[*]	0.294	28 out of 66
**Top Features for negative contribution**				
**Fingerprint**	**Bit/Smiles**	**Feature Structure**	**Score**	**Carcinogen in training set**
ECFP_12	1535429263	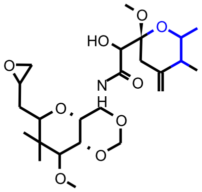<br>[*]OC(C)C([*])[*]	-0.485	0 out of 2
ECFP_12	1067196438	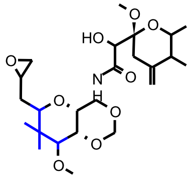<br>[*]C([*])C(C)(C)C([*]<br>)[*]	-0.485	0 out of 2
ECFP_12	-194719409	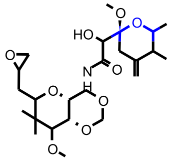<br>[*]C([*])OC([*])([*])<br>[*]	-0.466	1 out of 7

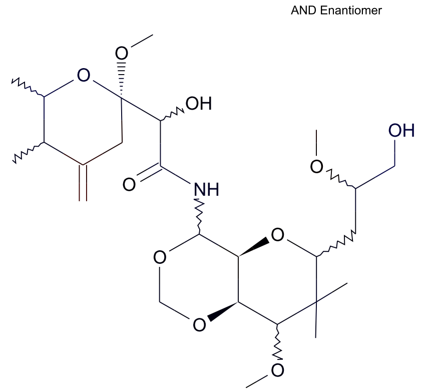

$C_{25}H_{43}NO_{10}$
Molecular Weight: 517.60962
ALogP: -9.1e-002
Rotatable Bonds: 9
Acceptors: 10
Donors: 3

Model Prediction

Prediction: Non-Carcinogen
Probability: 0.220
Enrichment: 0.682
Bayesian Score: -5.128
Mahalanobis Distance: 14.718
Mahalanobis Distance p-value: 7.74e-008

Prediction: Positive if the Bayesian score is above the estimated best cutoff value from minimizing the false positive and false negative rate.
Probability: The esimated probability that the sample is in the positive category. This assumes that the Bayesian score follows a normal distribution and is different from the prediction using a cutoff.
Enrichment: An estimate of enrichment, that is, the increased likelihood (versus random) of this sample being in the category.
Bayesian Score: The standard Laplacian-modified Bayesian score.
Mahalanobis Distance: The Mahalanobis distance (MD) is the distance to the center of the training data. The larger the MD, the less trustworthy the prediction.
Mahalanobis Distance p-value: The p-value gives the fraction of training data with an MD greater than or equal to the one for the given sample, assuming normally distributed data. The smaller the p-value, the less trustworthy the prediciton. For highly non-normal X properties (e.g., fingerprints), the MD p-value is wildly inaccurate.

Structural Similar Compounds

Name	Bacampicillin	Lasiocarpine	Dipyridamole
Structure	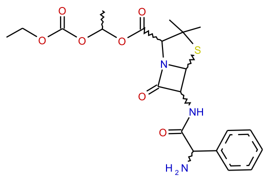	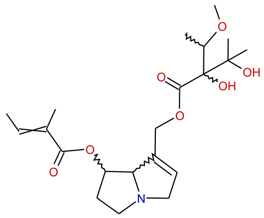	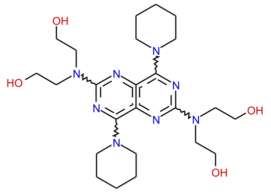
Actual Endpoint	Non-Carcinogen	Carcinogen	Non-Carcinogen
Predicted Endpoint	Non-Carcinogen	Carcinogen	Non-Carcinogen
Distance	0.748	0.800	0.867
Reference	US FDA (Centre for Drug Eval.& Res./Off. Testing & Res.) Sept. 1997	US FDA (Centre for Drug Eval.& Res./Off. Testing & Res.) Sept. 1997	US FDA (Centre for Drug Eval.& Res./Off. Testing & Res.) Sept. 1997

Model Applicability

Unknown features are fingerprint features in the query molecule, but not found in the training set.

1. All properties and OPS components are within expected ranges.
2. Unknown ECFP_2 feature: -457637478: [*]NC(O[*])C([*])[*]
3. Unknown ECFP_2 feature: 837844420: [*]CC(O[*])(O[*])C([*])[*]
4. Unknown ECFP_2 feature: 825070174: [*]C(=[*])C(O)C([*])([*])[*]

Feature Contribution

Top features for positive contribution				
Fingerprint	Bit/Smiles	Feature Structure	Score	Carcinogen in training set
ECFP_12	-2085781978	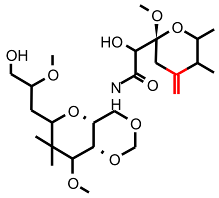 [*]C(=C)[*]	0.613	2 out of 2

ECFP_12	1377749300	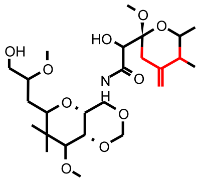 [*]CC(=C)C([*])[*]	0.421	1 out of 1
ECFP_12	-410173153	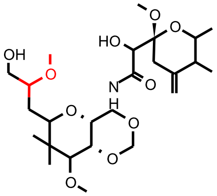 [*]C([*])OC	0.421	1 out of 1

Top Features for negative contribution

Fingerprint	Bit/Smiles	Feature Structure	Score	Carcinogen in training set
ECFP_12	-1909093651	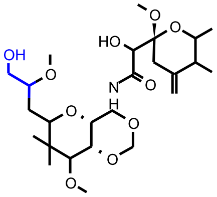 [*]C([*])CO	-1.056	0 out of 6
ECFP_12	1535429263	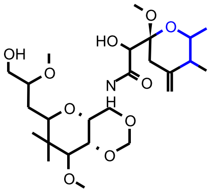 [*]OC(C)C([*])[*]	-0.485	0 out of 2
ECFP_12	1067196438	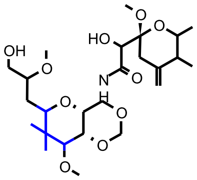 [*]C([*])C(C)(C)C([*])[*]	-0.485	0 out of 2

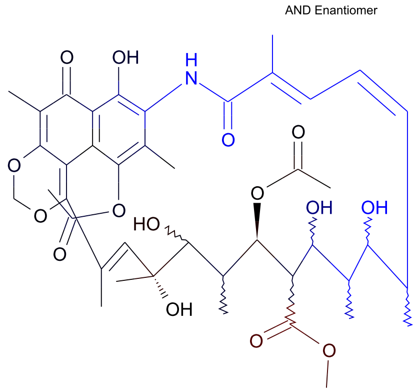

$C_{42}H_{53}NO_{15}$
Molecular Weight: 811.86791
ALogP: 2.745
Rotatable Bonds: 6
Acceptors: 15
Donors: 6

Model Prediction

Prediction: Non-Carcinogen
Probability: 0.152
Enrichment: 0.471
Bayesian Score: -16.239
Mahalanobis Distance: 11.987
Mahalanobis Distance p-value: 0.00495

Prediction: Positive if the Bayesian score is above the estimated best cutoff value from minimizing the false positive and false negative rate.
Probability: The esimated probability that the sample is in the positive category. This assumes that the Bayesian score follows a normal distribution and is different from the prediction using a cutoff.
Enrichment: An estimate of enrichment, that is, the increased likelihood (versus random) of this sample being in the category.
Bayesian Score: The standard Laplacian-modified Bayesian score.
Mahalanobis Distance: The Mahalanobis distance (MD) is the distance to the center of the training data. The larger the MD, the less trustworthy the prediction.
Mahalanobis Distance p-value: The p-value gives the fraction of training data with an MD greater than or equal to the one for the given sample, assuming normally distributed data. The smaller the p-value, the less trustworthy the prediciton. For highly non-normal X properties (e.g., fingerprints), the MD p-value is wildly inaccurate.

Structural Similar Compounds

Name	Rifampin	Rifabutin	Erythromycin
Structure	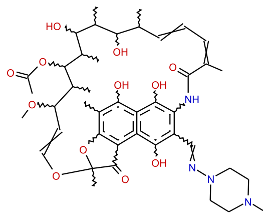	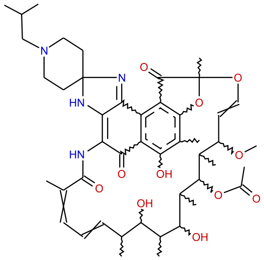	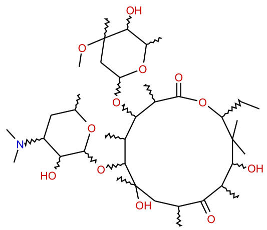
Actual Endpoint	Non-Carcinogen	Non-Carcinogen	Non-Carcinogen
Predicted Endpoint	Non-Carcinogen	Non-Carcinogen	Non-Carcinogen
Distance	0.505	0.679	0.854
Reference	US FDA (Centre for Drug Eval.& Res./Off. Testing & Res.) Sept. 1997	US FDA (Centre for Drug Eval.& Res./Off. Testing & Res.) Sept. 1997	US FDA (Centre for Drug Eval.& Res./Off. Testing & Res.) Sept. 1997

Model Applicability

Unknown features are fingerprint features in the query molecule, but not found in the training set.

1. All properties and OPS components are within expected ranges.
2. Unknown ECFP_2 feature: -40769921: [*]C([*])[C@](C)(O)C=[*]
3. Unknown ECFP_2 feature: 464446479: [*]C(=CC([*])([*])[*])[*]

Feature Contribution

Top features for positive contribution				
Fingerprint	Bit/Smiles	Feature Structure	Score	Carcinogen in training set
ECFP_12	-954757448	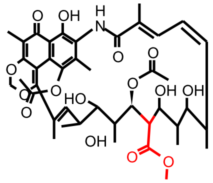 [*]C([*])C(=O)OC	0.613	2 out of 2

ECFP_12	706080645	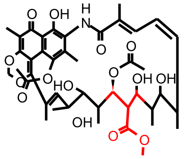<br>[*]C([*])C(C(C([*])[*])<br>C(=O)OC	0.613	2 out of 2
ECFP_12	-1531301414	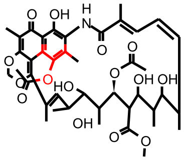<br>[*]O[c](:[c]([*]):[*]<br>):[c]([*]):[*]	0.454	5 out of 9
**Top Features for negative contribution**				
**Fingerprint**	**Bit/Smiles**	**Feature Structure**	**Score**	**Carcinogen in training set**
ECFP_12	-1660913849	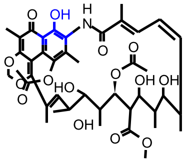<br>[*][c](:[*]):[c](O):[<br>c]([*]):[*]	-0.941	0 out of 5
ECFP_12	1311676480	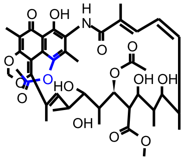<br>[*]C(=[*])O[c](:[*]):<br>[*]	-0.811	0 out of 4
ECFP_12	824248976	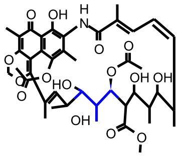<br>[*]C([*])C(C)C([*])[*<br>]	-0.661	0 out of 3

Simeprevir

TOPKAT_Rat_Female_FDA_None_vs_Carcinogen

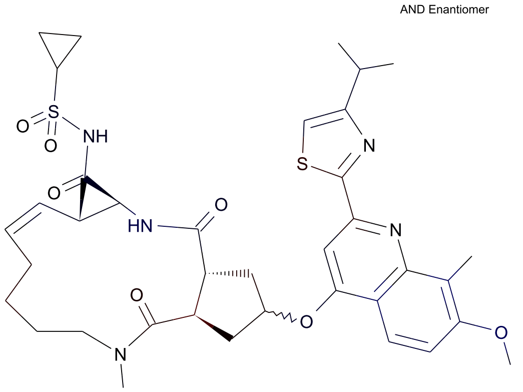

$C_{38}H_{47}N_5O_7S_2$
Molecular Weight: 749.93907
ALogP: 4.796
Rotatable Bonds: 8
Acceptors: 9
Donors: 2

Structural Similar Compounds

Name	Reserpine	Deserpidine	Bromocriptine
Structure	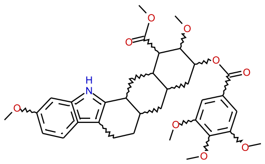	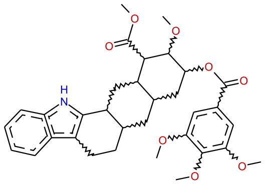	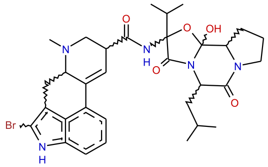
Actual Endpoint	Carcinogen	Carcinogen	Carcinogen
Predicted Endpoint	Carcinogen	Carcinogen	Carcinogen
Distance	0.807	0.846	0.874
Reference	US FDA (Centre for Drug Eval.& Res./Off. Testing & Res.) Sept. 1997	US FDA (Centre for Drug Eval.& Res./Off. Testing & Res.) Sept. 1997	US FDA (Centre for Drug Eval.& Res./Off. Testing & Res.) Sept. 1997

Model Prediction

Prediction: Non-Carcinogen
Probability: 0.231
Enrichment: 0.719
Bayesian Score: -4.130
Mahalanobis Distance: 14.568
Mahalanobis Distance p-value: 1.6e-007

Prediction: Positive if the Bayesian score is above the estimated best cutoff value from minimizing the false positive and false negative rate.
Probability: The esimated probability that the sample is in the positive category. This assumes that the Bayesian score follows a normal distribution and is different from the prediction using a cutoff.
Enrichment: An estimate of enrichment, that is, the increased likelihood (versus random) of this sample being in the category.
Bayesian Score: The standard Laplacian-modified Bayesian score.
Mahalanobis Distance: The Mahalanobis distance (MD) is the distance to the center of the training data. The larger the MD, the less trustworthy the prediction.
Mahalanobis Distance p-value: The p-value gives the fraction of training data with an MD greater than or equal to the one for the given sample, assuming normally distributed data. The smaller the p-value, the less trustworthy the prediciton. For highly non-normal X properties (e.g., fingerprints), the MD p-value is wildly inaccurate.

Model Applicability

Unknown features are fingerprint features in the query molecule, but not found in the training set.

1. All properties and OPS components are within expected ranges.
2. Unknown ECFP_2 feature: -309676563: [*]N[C@]1(C[C@H]1[*])C(=[*])[*]
3. Unknown ECFP_2 feature: -1049290660: [*]C1([*])C[C@H]1C=[*]
4. Unknown ECFP_2 feature: 1616402542: [*]CN(C)C(=[*])[*]
5. Unknown ECFP_2 feature: -1818486371: [*]NC(=O)C1([*])[*][*]1
6. Unknown ECFP_2 feature: 1310940530: [*]C([*])O[c](:[*]):[*]
7. Unknown ECFP_2 feature: 733491677: [*]:[c](:[*])C(C)C
8. Unknown ECFP_2 feature: 2121941848: [*]NS(=O)(=O)C1[*][*]1
9. Unknown ECFP_2 feature: -622223421: [*]S(=[*])(=[*])C1CC1

Feature Contribution

Top features for positive contribution				
Fingerprint	Bit/Smiles	Feature Structure	Score	Carcinogen in training set

ECFP_12	-1331088410	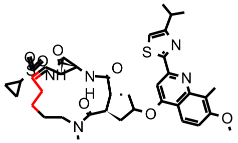 [*]CCC=[*]	0.725	3 out of 3
ECFP_12	-2095963820	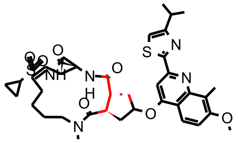 [*][C@@H]1[*][*]C[C@H]1C(=[*])[*]	0.722	12 out of 17
ECFP_12	914325265	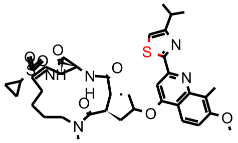 [*]:s:[*]	0.516	8 out of 14
**Top Features for negative contribution**				
**Fingerprint**	**Bit/Smiles**	**Feature Structure**	**Score**	**Carcinogen in training set**
ECFP_12	2077607946	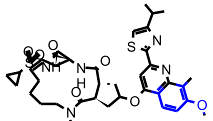 [*]O[c]1:[cH]:[cH]:[c](:[*]):[*]:[c]:1[*]	-1.253	0 out of 8
ECFP_12	-867777309	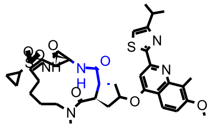 [*]NC(=O)C([*])[*]	-0.661	0 out of 3

ECFP_12	1408898974	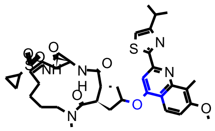 [*]O[c](:[cH]:[*]):[c]([*]):[*]	-0.517	5 out of 29

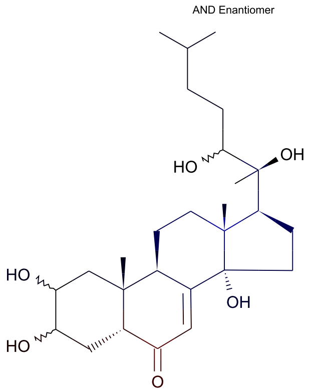

$C_{27}H_{44}O_6$
Molecular Weight: 464.63466
ALogP: 2.489
Rotatable Bonds: 5
Acceptors: 6
Donors: 5

Model Prediction

Prediction: Single-Carcinogen
Probability: 0.425
Enrichment: 1.138
Bayesian Score: -2.582
Mahalanobis Distance: 15.663
Mahalanobis Distance p-value: 6.91e-007

Prediction: Positive if the Bayesian score is above the estimated best cutoff value from minimizing the false positive and false negative rate.
Probability: The esimated probability that the sample is in the positive category. This assumes that the Bayesian score follows a normal distribution and is different from the prediction using a cutoff.
Enrichment: An estimate of enrichment, that is, the increased likelihood (versus random) of this sample being in the category.
Bayesian Score: The standard Laplacian-modified Bayesian score.
Mahalanobis Distance: The Mahalanobis distance (MD) is the distance to the center of the training data. The larger the MD, the less trustworthy the prediction.
Mahalanobis Distance p-value: The p-value gives the fraction of training data with an MD greater than or equal to the one for the given sample, assuming normally distributed data. The smaller the p-value, the less trustworthy the prediciton. For highly non-normal X properties (e.g., fingerprints), the MD p-value is wildly inaccurate.

Structural Similar Compounds

Name	Mesuprine	Ursodiol	Minocycline
Structure	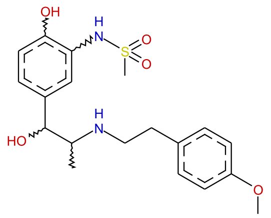	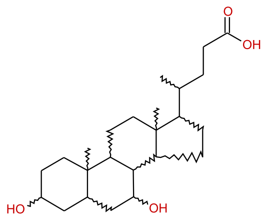	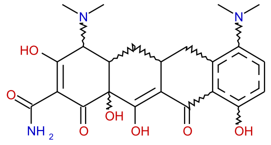
Actual Endpoint	Single-Carcinogen	Single-Carcinogen	Single-Carcinogen
Predicted Endpoint	Single-Carcinogen	Single-Carcinogen	Single-Carcinogen
Distance	0.798	0.821	0.860
Reference	US FDA (Centre for Drug Eval.& Res./Off. Testing & Res.) Sept. 1997	US FDA (Centre for Drug Eval.& Res./Off. Testing & Res.) Sept. 1997	US FDA (Centre for Drug Eval.& Res./Off. Testing & Res.) Sept. 1997

Model Applicability

Unknown features are fingerprint features in the query molecule, but not found in the training set.

1. All properties and OPS components are within expected ranges.

Feature Contribution

Top features for positive contribution				
**Fingerprint**	**Bit/Smiles**	**Feature Structure**	**Score**	**Multiple-Carcinogen in training set**
SCFP_4	-973999932	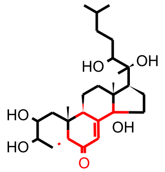 [*][C@@H]1[*][C@@H]([*])C(=CC1=O)C([*])([*])[*]	0.610	2 out of 2

SCFP_4	-1903488337	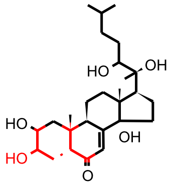<br>[*]C1[*]C([*])([*])[C<br>@@H](CC1O)C(=[*])[*]	0.419	1 out of 1
SCFP_4	-1971196727	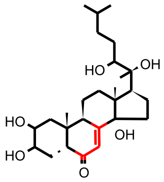<br>[*]C(=CC(=[*])[*])[*]	0.295	5 out of 11
**Top Features for negative contribution**				
**Fingerprint**	**Bit/Smiles**	**Feature Structure**	**Score**	**Multiple-Carcinogen in training set**
SCFP_4	-1043339860	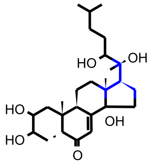<br>[*]C([*])([*])[C@H]1C<br>[*][*]C1([*])[*]	-0.675	4 out of 28
SCFP_4	-1870530637	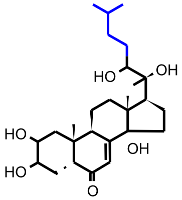<br>[*]CCC(C)C	-0.489	0 out of 2
SCFP_4	-511672165	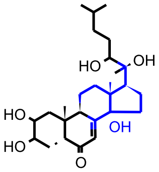<br>[*]C([*])([*])[C@H]1C<br>C[C@@]2(O)C(=[*])[*]<br>CC[C@]12C	-0.489	0 out of 2

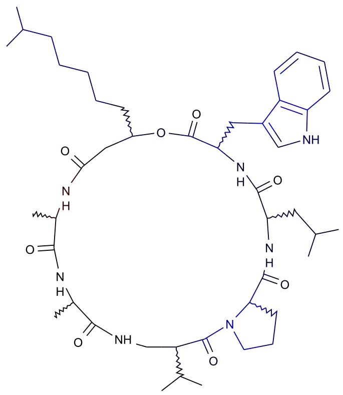

$C_{45}H_{69}N_7O_8$
Molecular Weight: 836.07145
ALogP: 4.986
Rotatable Bonds: 11
Acceptors: 8
Donors: 6

Model Prediction

Prediction: Single-Carcinogen
Probability: 0.018
Enrichment: 0.048
Bayesian Score: -13.077
Mahalanobis Distance: 20.115
Mahalanobis Distance p-value: 4.33e-011

Prediction: Positive if the Bayesian score is above the estimated best cutoff value from minimizing the false positive and false negative rate.
Probability: The esimated probability that the sample is in the positive category. This assumes that the Bayesian score follows a normal distribution and is different from the prediction using a cutoff.
Enrichment: An estimate of enrichment, that is, the increased likelihood (versus random) of this sample being in the category.
Bayesian Score: The standard Laplacian-modified Bayesian score.
Mahalanobis Distance: The Mahalanobis distance (MD) is the distance to the center of the training data. The larger the MD, the less trustworthy the prediction.
Mahalanobis Distance p-value: The p-value gives the fraction of training data with an MD greater than or equal to the one for the given sample, assuming normally distributed data. The smaller the p-value, the less trustworthy the prediciton. For highly non-normal X properties (e.g., fingerprints), the MD p-value is wildly inaccurate.

Structural Similar Compounds

Name	Bromocriptine	Prednimustine	Salmeterol
Structure	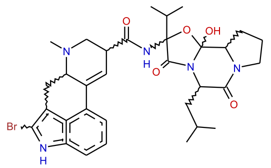	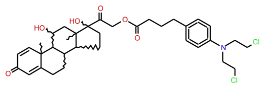	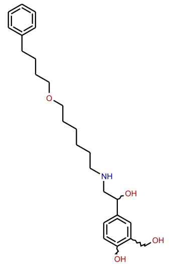
Actual Endpoint	Single-Carcinogen	Single-Carcinogen	Single-Carcinogen
Predicted Endpoint	Single-Carcinogen	Single-Carcinogen	Single-Carcinogen
Distance	1.103	1.232	1.366
Reference	US FDA (Centre for Drug Eval.& Res./Off. Testing & Res.) Sept. 1997	US FDA (Centre for Drug Eval.& Res./Off. Testing & Res.) Sept. 1997	US FDA (Centre for Drug Eval.& Res./Off. Testing & Res.) Sept. 1997

Model Applicability

Unknown features are fingerprint features in the query molecule, but not found in the training set.

1. Molecular_Weight out of range. Value: 836.07. Training min, max, mean, SD: 59.067, 654.59, 292.9, 119.1.
2. Num_H_Donors out of range. Value: 6. Training min, max, mean, SD: 0, 5, 1.3902, 1.143.
3. OPS PC8 out of range. Value: 5.542. Training min, max, SD, explained variance: -3.3768, 4.2738, 1.554, 0.0358.

Feature Contribution

Top features for positive contribution

Fingerprint	Bit/Smiles	Feature Structure	Score	Multiple-Carcinogen in training set
SCFP_4	-111024397	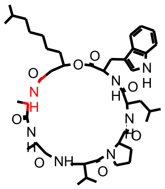 [*]C([*])NC(=[*])[*]	0.351	4 out of 8

SCFP_4	1256995004	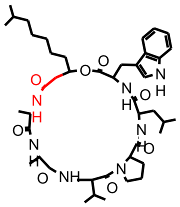 [*]CC(=O)N[*]	0.327	10 out of 22
SCFP_4	149003983	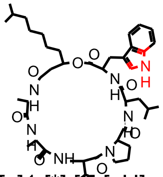 [*]:[c]1:[*]:[*]:[cH] :[nH]:1	0.266	11 out of 26

Top Features for negative contribution

**Fingerprint**	**Bit/Smiles**	**Feature Structure**	**Score**	**Multiple-Carcinogen in training set**
SCFP_4	1175638033	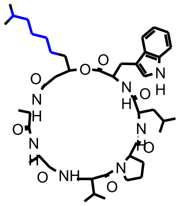 [*]CCCCC([*])[*]	-1.165	0 out of 7
SCFP_4	-1272709286	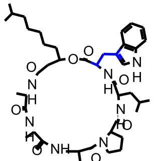 [*]C([*])C[c](:[*]):[*]	-1.157	1 out of 17
SCFP_4	-1343150366	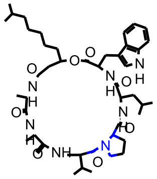 [*]C1[*][*]CN1C(=[*]) [*]	-0.946	0 out of 5

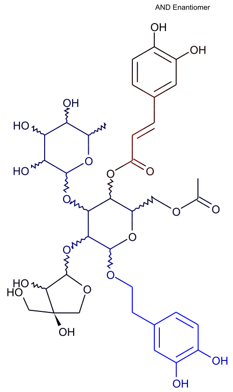

$C_{36}H_{46}O_{20}$
Molecular Weight: 798.73843
ALogP: -0.546
Rotatable Bonds: 16
Acceptors: 20
Donors: 10

Model Prediction

Prediction: Non-Carcinogen
Probability: 0.139
Enrichment: 0.416
Bayesian Score: -11.931
Mahalanobis Distance: 19.254
Mahalanobis Distance p-value: 8.18e-017

Prediction: Positive if the Bayesian score is above the estimated best cutoff value from minimizing the false positive and false negative rate.
Probability: The esimated probability that the sample is in the positive category. This assumes that the Bayesian score follows a normal distribution and is different from the prediction using a cutoff.
Enrichment: An estimate of enrichment, that is, the increased likelihood (versus random) of this sample being in the category.
Bayesian Score: The standard Laplacian-modified Bayesian score.
Mahalanobis Distance: The Mahalanobis distance (MD) is the distance to the center of the training data. The larger the MD, the less trustworthy the prediction.
Mahalanobis Distance p-value: The p-value gives the fraction of training data with an MD greater than or equal to the one for the given sample, assuming normally distributed data. The smaller the p-value, the less trustworthy the prediciton. For highly non-normal X properties (e.g., fingerprints), the MD p-value is wildly inaccurate.

Structural Similar Compounds

Name	Rifampin	Natamycin	Netilmicin
Structure	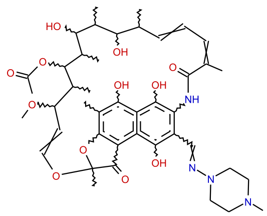	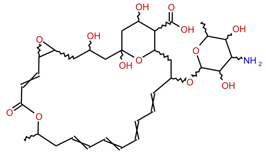	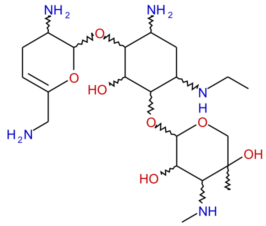
Actual Endpoint	Non-Carcinogen	Non-Carcinogen	Non-Carcinogen
Predicted Endpoint	Non-Carcinogen	Non-Carcinogen	Non-Carcinogen
Distance	1.506	1.523	1.547
Reference	US FDA (Centre for Drug Eval.& Res./Off. Testing & Res.) Sept. 1997	US FDA (Centre for Drug Eval.& Res./Off. Testing & Res.) Sept. 1997	US FDA (Centre for Drug Eval.& Res./Off. Testing & Res.) Sept. 1997

Model Applicability

Unknown features are fingerprint features in the query molecule, but not found in the training set.

1. Num_H_Acceptors out of range. Value: 20. Training min, max, mean, SD: 0, 15, 4.1723, 2.348.
2. OPS PC10 out of range. Value: -5.0703. Training min, max, SD, explained variance: -4.2502, 5.657, 1.784, 0.0297.

Feature Contribution

Top features for positive contribution

Fingerprint	Bit/Smiles	Feature Structure	Score	Carcinogen in training set
SCFP_6	1590423958	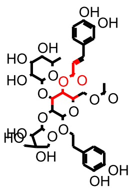 AND Enantiomer [*]C([*])C(OC(=O)C=[*])C([*])[*]	0.675	6 out of 8

SCFP_6	-1971137145	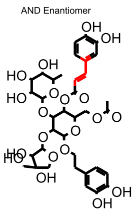 [*]\C=C\[c](:[*]):[*]	0.434	5 out of 9
SCFP_6	-1977229858	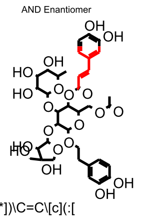 [*]C(=[*])\C=C\[c](:[cH]:[*]):[cH]:[*]	0.425	2 out of 3

Top Features for negative contribution

Fingerprint	Bit/Smiles	Feature Structure	Score	Carcinogen in training set
SCFP_6	-1642341584	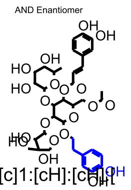 [*]CC[c]1:[cH]:[cH]:[c](O):[c]([*]):[cH]:1	-1.271	0 out of 8
SCFP_6	-1211866396	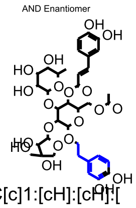 [*]CC[c]1:[cH]:[cH]:[*]:[c]([*]):[cH]:1	-1.101	2 out of 25
SCFP_6	-820970991	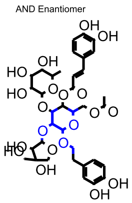 [*]COC1OC([*])[*]C([*])C1O[*]	-0.825	0 out of 4

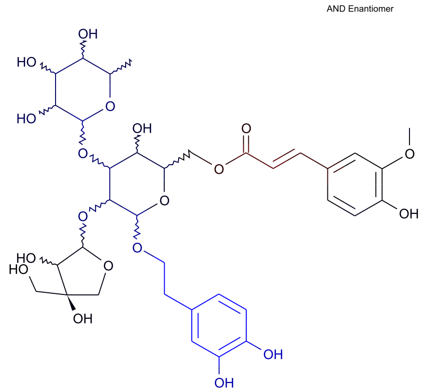

$C_{35}H_{46}O_{19}$
Molecular Weight: 770.72833
ALogP: -0.699
Rotatable Bonds: 15
Acceptors: 19
Donors: 10

Model Prediction

Prediction: Non-Carcinogen
Probability: 0.135
Enrichment: 0.405
Bayesian Score: -12.223
Mahalanobis Distance: 18.658
Mahalanobis Distance p-value: 2.73e-015

Prediction: Positive if the Bayesian score is above the estimated best cutoff value from minimizing the false positive and false negative rate.
Probability: The esimated probability that the sample is in the positive category. This assumes that the Bayesian score follows a normal distribution and is different from the prediction using a cutoff.
Enrichment: An estimate of enrichment, that is, the increased likelihood (versus random) of this sample being in the category.
Bayesian Score: The standard Laplacian-modified Bayesian score.
Mahalanobis Distance: The Mahalanobis distance (MD) is the distance to the center of the training data. The larger the MD, the less trustworthy the prediction.
Mahalanobis Distance p-value: The p-value gives the fraction of training data with an MD greater than or equal to the one for the given sample, assuming normally distributed data. The smaller the p-value, the less trustworthy the prediciton. For highly non-normal X properties (e.g., fingerprints), the MD p-value is wildly inaccurate.

Structural Similar Compounds

Name	Natamycin	Netilmicin	Rifampin
Structure	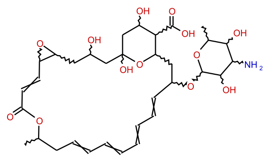	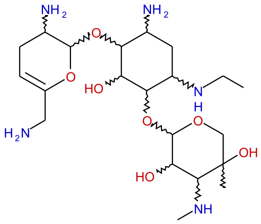	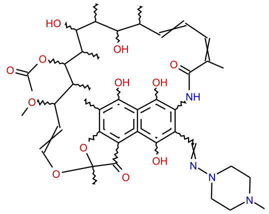
Actual Endpoint	Non-Carcinogen	Non-Carcinogen	Non-Carcinogen
Predicted Endpoint	Non-Carcinogen	Non-Carcinogen	Non-Carcinogen
Distance	1.431	1.436	1.443
Reference	US FDA (Centre for Drug Eval.& Res./Off. Testing & Res.) Sept. 1997	US FDA (Centre for Drug Eval.& Res./Off. Testing & Res.) Sept. 1997	US FDA (Centre for Drug Eval.& Res./Off. Testing & Res.) Sept. 1997

Model Applicability

Unknown features are fingerprint features in the query molecule, but not found in the training set.

1. Num_H_Acceptors out of range. Value: 19. Training min, max, mean, SD: 0, 15, 4.1723, 2.348.
2. OPS PC10 out of range. Value: -4.967. Training min, max, SD, explained variance: -4.2502, 5.657, 1.784, 0.0297.

Feature Contribution

Top features for positive contribution

Fingerprint	Bit/Smiles	Feature Structure	Score	Carcinogen in training set
SCFP_6	-1971137145	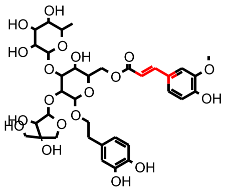 [*]\C=C\[c](:[*]):[*]	0.434	5 out of 9

SCFP_6	-1977229858	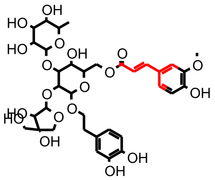 [*]C(=[*])\C=C\[c](:[cH]:[*]):[cH]:[*]	0.425	2 out of 3
SCFP_6	1284965536	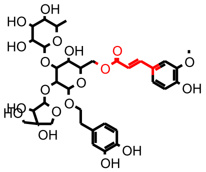 [*]OC(=O)\C=C\[c](:[*]):[*]	0.425	2 out of 3
**Top Features for negative contribution**				
**Fingerprint**	**Bit/Smiles**	**Feature Structure**	**Score**	**Carcinogen in training set**
SCFP_6	-1642341584	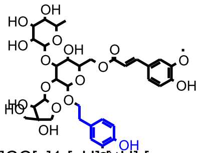 [*]CC[c]1:[cH]:[cH]:[c](O):[c]([*]):[cH]:1	-1.271	0 out of 8
SCFP_6	-1211866396	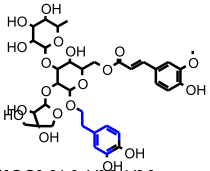 [*]CC[c]1:[cH]:[cH]:[*]:[c]([*]):[cH]:1	-1.101	2 out of 25
SCFP_6	2116304939	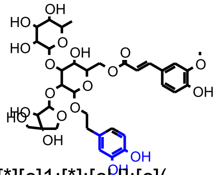 [*][c]1:[*]:[cH]:[c](O):[c](O):[cH]:1	-0.825	0 out of 4

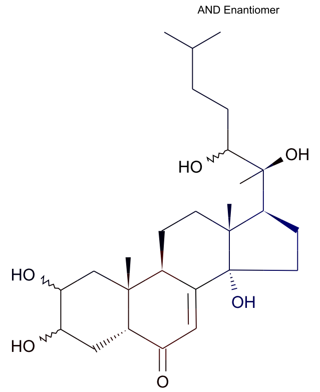

$C_{27}H_{44}O_6$
Molecular Weight: 464.63466
ALogP: 2.489
Rotatable Bonds: 5
Acceptors: 6
Donors: 5

Model Prediction

Prediction: Non-Carcinogen
Probability: 0.298
Enrichment: 0.893
Bayesian Score: -2.193
Mahalanobis Distance: 16.132
Mahalanobis Distance p-value: 2.72e-009

Prediction: Positive if the Bayesian score is above the estimated best cutoff value from minimizing the false positive and false negative rate.
Probability: The esimated probability that the sample is in the positive category. This assumes that the Bayesian score follows a normal distribution and is different from the prediction using a cutoff.
Enrichment: An estimate of enrichment, that is, the increased likelihood (versus random) of this sample being in the category.
Bayesian Score: The standard Laplacian-modified Bayesian score.
Mahalanobis Distance: The Mahalanobis distance (MD) is the distance to the center of the training data. The larger the MD, the less trustworthy the prediction.
Mahalanobis Distance p-value: The p-value gives the fraction of training data with an MD greater than or equal to the one for the given sample, assuming normally distributed data. The smaller the p-value, the less trustworthy the prediciton. For highly non-normal X properties (e.g., fingerprints), the MD p-value is wildly inaccurate.

Structural Similar Compounds

Name	Triamcinolone	Budesonide	Beclomethasone
Structure	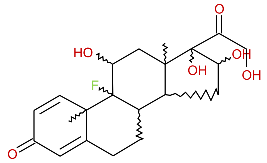	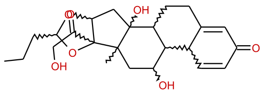	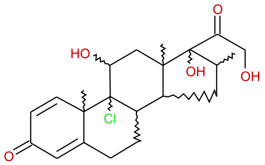
Actual Endpoint	Non-Carcinogen	Carcinogen	Non-Carcinogen
Predicted Endpoint	Non-Carcinogen	Carcinogen	Non-Carcinogen
Distance	0.756	0.771	0.783
Reference	US FDA (Centre for Drug Eval.& Res./Off. Testing & Res.) Sept. 1997	US FDA (Centre for Drug Eval.& Res./Off. Testing & Res.) Sept. 1997	US FDA (Centre for Drug Eval.& Res./Off. Testing & Res.) Sept. 1997

Model Applicability

Unknown features are fingerprint features in the query molecule, but not found in the training set.

1. All properties and OPS components are within expected ranges.

Feature Contribution

Top features for positive contribution

Fingerprint	Bit/Smiles	Feature Structure	Score	Carcinogen in training set
SCFP_6	-1903488337	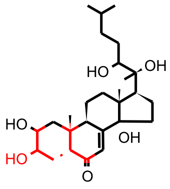 [*]C1[*]C([*])([*])[C@@H](CC1O)C(=[*])[*]	0.603	2 out of 2

SCFP_6	1416226694	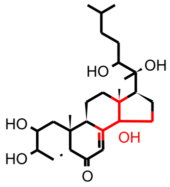<br>[*]C(=[*])[C@]1(O)C[*<br>][*]C1([*])[*]	0.438	8 out of 15
SCFP_6	-1971196727	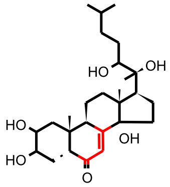<br>[*]\C=C\C(=[*])[*]	0.361	17 out of 36
**Top Features for negative contribution**				
**Fingerprint**	**Bit/Smiles**	**Feature Structure**	**Score**	**Carcinogen in training set**
SCFP_6	-511672165	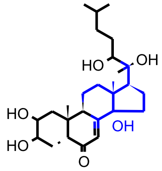<br>[*]C([*])([*])[C@H]1C<br>C[C@@]2(O)C(=[*])[*]<br>CC[C@]12C	-1.177	0 out of 7
SCFP_6	-1546614078	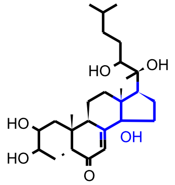<br>[*][C@H]1CC[C@@](O)(C<br>(=[*])[*])C1([*])[*]	-0.825	0 out of 4
SCFP_6	-1870530637	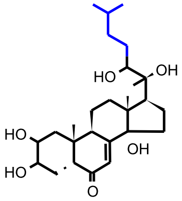<br>[*]CCC(C)C	-0.674	0 out of 3

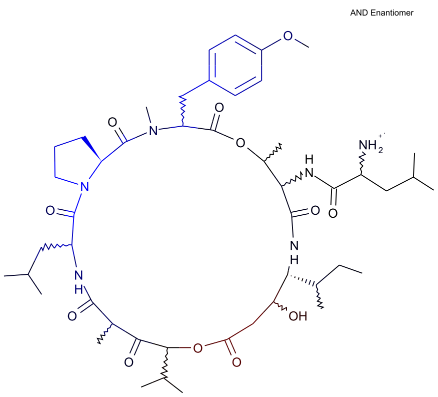

$C_{49}H_{79}N_6O_{12}$
Molecular Weight: 944.18455
ALogP: 3.176
Rotatable Bonds: 13
Acceptors: 12
Donors: 5

Model Prediction

Prediction: Non-Carcinogen
Probability: 0.099
Enrichment: 0.295
Bayesian Score: -15.536
Mahalanobis Distance: 16.154
Mahalanobis Distance p-value: 2.43e-009

Prediction: Positive if the Bayesian score is above the estimated best cutoff value from minimizing the false positive and false negative rate.
Probability: The esimated probability that the sample is in the positive category. This assumes that the Bayesian score follows a normal distribution and is different from the prediction using a cutoff.
Enrichment: An estimate of enrichment, that is, the increased likelihood (versus random) of this sample being in the category.
Bayesian Score: The standard Laplacian-modified Bayesian score.
Mahalanobis Distance: The Mahalanobis distance (MD) is the distance to the center of the training data. The larger the MD, the less trustworthy the prediction.
Mahalanobis Distance p-value: The p-value gives the fraction of training data with an MD greater than or equal to the one for the given sample, assuming normally distributed data. The smaller the p-value, the less trustworthy the prediciton. For highly non-normal X properties (e.g., fingerprints), the MD p-value is wildly inaccurate.

Structural Similar Compounds

Name	Rifabutin	Erythromycin	Rifampin
Structure	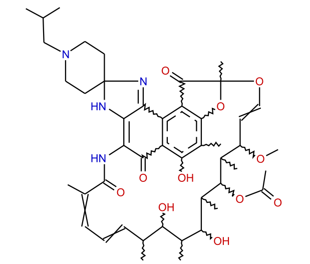	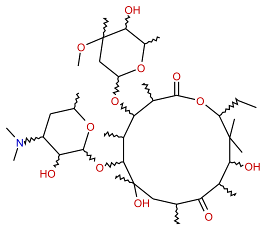	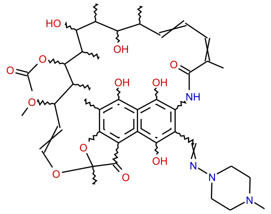
Actual Endpoint	Non-Carcinogen	Non-Carcinogen	Non-Carcinogen
Predicted Endpoint	Non-Carcinogen	Non-Carcinogen	Non-Carcinogen
Distance	1.003	1.008	1.064
Reference	US FDA (Centre for Drug Eval.& Res./Off. Testing & Res.) Sept. 1997	US FDA (Centre for Drug Eval.& Res./Off. Testing & Res.) Sept. 1997	US FDA (Centre for Drug Eval.& Res./Off. Testing & Res.) Sept. 1997

Model Applicability

Unknown features are fingerprint features in the query molecule, but not found in the training set.

1. Molecular_Weight out of range. Value: 944.18. Training min, max, mean, SD: 46.068, 847, 305.01, 115.4.

Feature Contribution

Top features for positive contribution

Fingerprint	Bit/Smiles	Feature Structure	Score	Carcinogen in training set
SCFP_6	2140026027	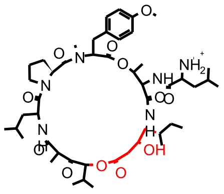 [*]OC(=O)CC(O)C([*])[*]	0.784	4 out of 4

SCFP_6	-1109046624	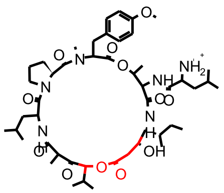 [*]C([*])CC(=O)OC([*])[*]	0.675	6 out of 8
SCFP_6	276223760	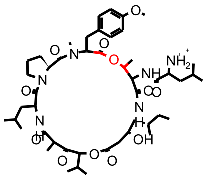 [*]C([*])OC(=[*])[*]	0.243	24 out of 58

Top Features for negative contribution

Fingerprint	Bit/Smiles	Feature Structure	Score	Carcinogen in training set
SCFP_6	-1642341584	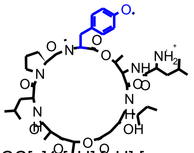 [*]CC[c]1:[cH]:[cH]:[c](O):[c]([*]):[cH]: 1	-1.271	0 out of 8
SCFP_6	-1211866396	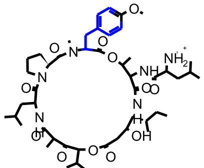 [*]CC[c]1:[cH]:[cH]:[*]:[c]([*]):[cH]:1	-1.101	2 out of 25
SCFP_6	-1316367761	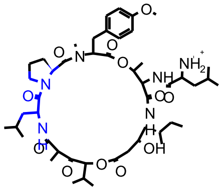 [*]CC(N[*])C(=O)N1C[*][*][C@H]1[*]	-0.957	0 out of 5

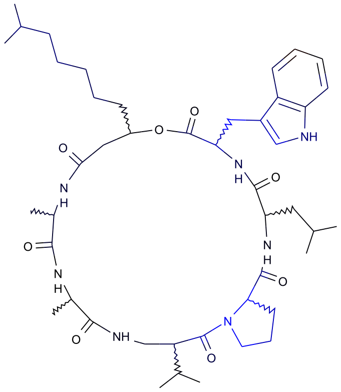

$C_{45}H_{69}N_7O_8$
Molecular Weight: 836.07145
ALogP: 4.986
Rotatable Bonds: 11
Acceptors: 8
Donors: 6

Model Prediction

Prediction: Non-Carcinogen
Probability: 0.141
Enrichment: 0.423
Bayesian Score: -11.745
Mahalanobis Distance: 16.365
Mahalanobis Distance p-value: 8.3e-010

Prediction: Positive if the Bayesian score is above the estimated best cutoff value from minimizing the false positive and false negative rate.
Probability: The esimated probability that the sample is in the positive category. This assumes that the Bayesian score follows a normal distribution and is different from the prediction using a cutoff.
Enrichment: An estimate of enrichment, that is, the increased likelihood (versus random) of this sample being in the category.
Bayesian Score: The standard Laplacian-modified Bayesian score.
Mahalanobis Distance: The Mahalanobis distance (MD) is the distance to the center of the training data. The larger the MD, the less trustworthy the prediction.
Mahalanobis Distance p-value: The p-value gives the fraction of training data with an MD greater than or equal to the one for the given sample, assuming normally distributed data. The smaller the p-value, the less trustworthy the prediciton. For highly non-normal X properties (e.g., fingerprints), the MD p-value is wildly inaccurate.

Structural Similar Compounds

Name	Bromocriptine	Erythromycin	Rifabutin
Structure	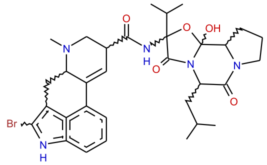	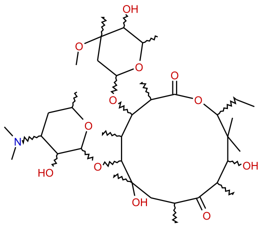	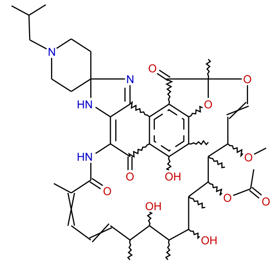
Actual Endpoint	Non-Carcinogen	Non-Carcinogen	Non-Carcinogen
Predicted Endpoint	Non-Carcinogen	Non-Carcinogen	Non-Carcinogen
Distance	1.080	1.090	1.122
Reference	US FDA (Centre for Drug Eval.& Res./Off. Testing & Res.) Sept. 1997	US FDA (Centre for Drug Eval.& Res./Off. Testing & Res.) Sept. 1997	US FDA (Centre for Drug Eval.& Res./Off. Testing & Res.) Sept. 1997

Model Applicability

Unknown features are fingerprint features in the query molecule, but not found in the training set.

1. All properties and OPS components are within expected ranges.

Feature Contribution

Top features for positive contribution

Fingerprint	Bit/Smiles	Feature Structure	Score	Carcinogen in training set
SCFP_6	1651620003	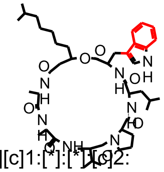 [*][c]1:[*]:[*]:[c]2:[cH]:[cH]:[cH]:[cH]:[c]:1:2	0.643	7 out of 10

SCFP_6	-1379673609	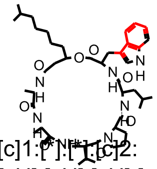 [*][c]1:[*]:[*]:[c]2:[*]:[cH]:[cH]:[cH]:[c]:1:2	0.526	11 out of 19
SCFP_6	1655199790	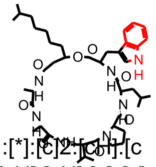 [*]1:[*]:[c]2:[cH]:[cH]:[cH]:[cH]:[c]:2:[nH]:1	0.520	5 out of 8

Top Features for negative contribution

Fingerprint	Bit/Smiles	Feature Structure	Score	Carcinogen in training set
SCFP_6	-1946889102	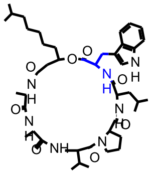 [*]N1[*][*]C[C@H]1C(=[*])[*]	-0.885	1 out of 12
SCFP_6	2002926168	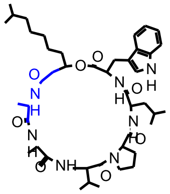 [*]CC(NC(=O)C([*])[*])C(=[*])[*]	-0.825	0 out of 4
SCFP_6	306578635	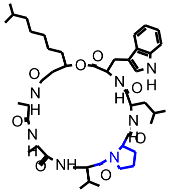 [*][C@@H]1CCCN1C(=[*])[*]	-0.825	0 out of 4

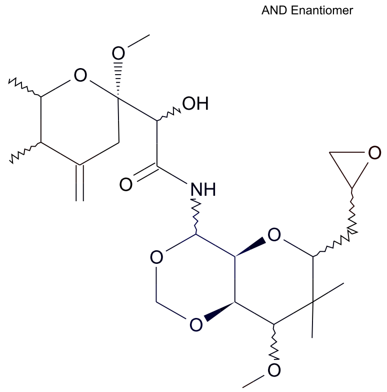

$C_{24}H_{39}NO_9$
Molecular Weight: 485.56776
ALogP: 0.267
Rotatable Bonds: 7
Acceptors: 9
Donors: 2

Model Prediction

Prediction: Non-Carcinogen
Probability: 0.300
Enrichment: 0.898
Bayesian Score: -2.113
Mahalanobis Distance: 17.231
Mahalanobis Distance p-value: 8.52e-012

Prediction: Positive if the Bayesian score is above the estimated best cutoff value from minimizing the false positive and false negative rate.
Probability: The esimated probability that the sample is in the positive category. This assumes that the Bayesian score follows a normal distribution and is different from the prediction using a cutoff.
Enrichment: An estimate of enrichment, that is, the increased likelihood (versus random) of this sample being in the category.
Bayesian Score: The standard Laplacian-modified Bayesian score.
Mahalanobis Distance: The Mahalanobis distance (MD) is the distance to the center of the training data. The larger the MD, the less trustworthy the prediction.
Mahalanobis Distance p-value: The p-value gives the fraction of training data with an MD greater than or equal to the one for the given sample, assuming normally distributed data. The smaller the p-value, the less trustworthy the prediciton. For highly non-normal X properties (e.g., fingerprints), the MD p-value is wildly inaccurate.

Structural Similar Compounds

Name	Lasiocarpine	Bacampicillin	Budesonide
Structure	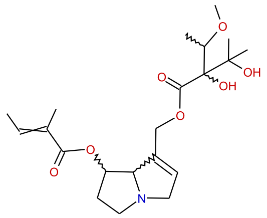	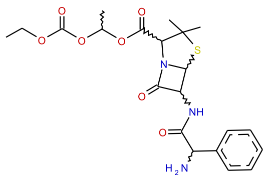	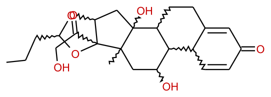
Actual Endpoint	Carcinogen	Non-Carcinogen	Carcinogen
Predicted Endpoint	Carcinogen	Non-Carcinogen	Carcinogen
Distance	0.707	0.718	0.760
Reference	US FDA (Centre for Drug Eval.& Res./Off. Testing & Res.) Sept. 1997	US FDA (Centre for Drug Eval.& Res./Off. Testing & Res.) Sept. 1997	US FDA (Centre for Drug Eval.& Res./Off. Testing & Res.) Sept. 1997

Model Applicability

Unknown features are fingerprint features in the query molecule, but not found in the training set.

1. All properties and OPS components are within expected ranges.

Feature Contribution

Top features for positive contribution

Fingerprint	Bit/Smiles	Feature Structure	Score	Carcinogen in training set
SCFP_6	-37603133	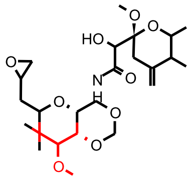 [*]C([*])C(OC)C([*])([*])[*]	0.561	3 out of 4

SCFP_6	-1905051945	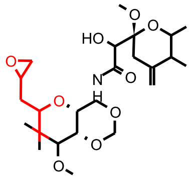 [*]OC(CC1CO1)C([*])([*])[*]	0.561	3 out of 4
SCFP_6	55434585	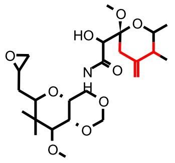 [*]\C=C(/C([*])[*])\C ([*])([*])[*]	0.331	12 out of 26
**Top Features for negative contribution**				
**Fingerprint**	**Bit/Smiles**	**Feature Structure**	**Score**	**Carcinogen in training set**
SCFP_6	-1485821968	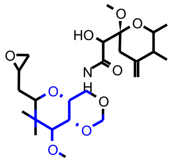 [*]OC1C([*])[*]C([*]) C(O[*])C1OC([*])[*]	-0.674	0 out of 3
SCFP_6	-712043691	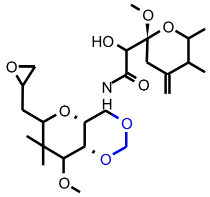 [*]OCO[*]	-0.278	0 out of 1
SCFP_6	312169906	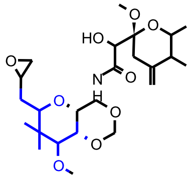 [*]CC1O[*][C@H]([*])C (O[*])C1(C)C	-0.278	0 out of 1

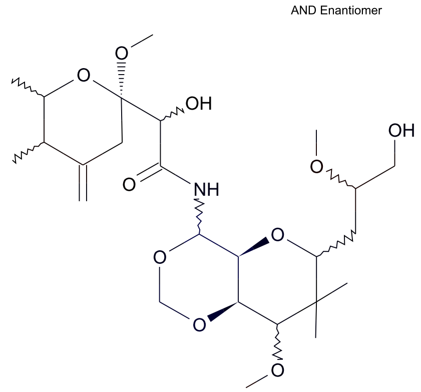

$C_{25}H_{43}NO_{10}$
Molecular Weight: 517.60962
ALogP: -9.1e-002
Rotatable Bonds: 9
Acceptors: 10
Donors: 3

Model Prediction

Prediction: Non-Carcinogen
Probability: 0.291
Enrichment: 0.871
Bayesian Score: -2.496
Mahalanobis Distance: 17.143
Mahalanobis Distance p-value: 1.37e-011

Prediction: Positive if the Bayesian score is above the estimated best cutoff value from minimizing the false positive and false negative rate.
Probability: The esimated probability that the sample is in the positive category. This assumes that the Bayesian score follows a normal distribution and is different from the prediction using a cutoff.
Enrichment: An estimate of enrichment, that is, the increased likelihood (versus random) of this sample being in the category.
Bayesian Score: The standard Laplacian-modified Bayesian score.
Mahalanobis Distance: The Mahalanobis distance (MD) is the distance to the center of the training data. The larger the MD, the less trustworthy the prediction.
Mahalanobis Distance p-value: The p-value gives the fraction of training data with an MD greater than or equal to the one for the given sample, assuming normally distributed data. The smaller the p-value, the less trustworthy the prediciton. For highly non-normal X properties (e.g., fingerprints), the MD p-value is wildly inaccurate.

Structural Similar Compounds

Name	Bacampicillin	Lasiocarpine	Dipyridamole
Structure	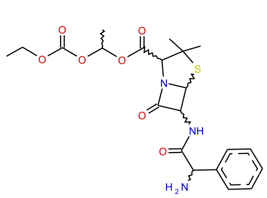	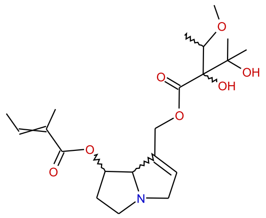	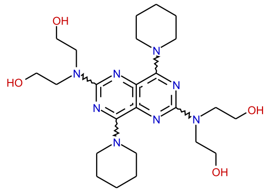
Actual Endpoint	Non-Carcinogen	Carcinogen	Non-Carcinogen
Predicted Endpoint	Non-Carcinogen	Carcinogen	Non-Carcinogen
Distance	0.736	0.772	0.853
Reference	US FDA (Centre for Drug Eval.& Res./Off. Testing & Res.) Sept. 1997	US FDA (Centre for Drug Eval.& Res./Off. Testing & Res.) Sept. 1997	US FDA (Centre for Drug Eval.& Res./Off. Testing & Res.) Sept. 1997

Model Applicability

Unknown features are fingerprint features in the query molecule, but not found in the training set.

1. All properties and OPS components are within expected ranges.

Feature Contribution

Top features for positive contribution				
**Fingerprint**	**Bit/Smiles**	**Feature Structure**	**Score**	**Carcinogen in training set**
SCFP_6	-37603133	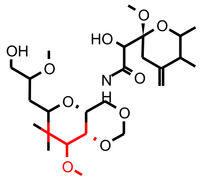 [*]C([*])C(OC)C([*])([*])[*]	0.561	3 out of 4

SCFP_6	-1905051945	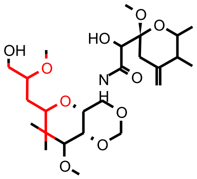 [*]OC(CC1CO1)C([*])([*])[*]	0.561	3 out of 4
SCFP_6	55434585	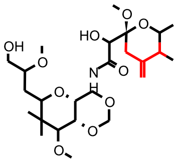 [*]\C=C(/C([*])[*])\C([*])([*])[*]	0.331	12 out of 26
**Top Features for negative contribution**				
**Fingerprint**	**Bit/Smiles**	**Feature Structure**	**Score**	**Carcinogen in training set**
SCFP_6	-1485821968	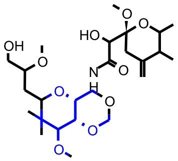 [*]OC1C([*])[*]C([*])C(O[*])C1OC([*])[*]	-0.674	0 out of 3
SCFP_6	312169906	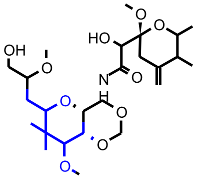 [*]CC1O[*][C@H]([*])C(O[*])C1(C)C	-0.278	0 out of 1
SCFP_6	-503761529	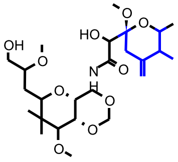 [*]C1[*]C([*])([*])CC(=C)C1C	-0.278	0 out of 1

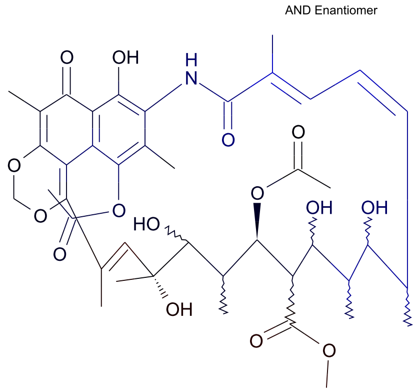

$C_{42}H_{53}NO_{15}$
Molecular Weight: 811.86791
ALogP: 2.745
Rotatable Bonds: 6
Acceptors: 15
Donors: 6

Model Prediction

Prediction: Non-Carcinogen
Probability: 0.184
Enrichment: 0.550
Bayesian Score: -8.510
Mahalanobis Distance: 16.141
Mahalanobis Distance p-value: 2.6e-009

Prediction: Positive if the Bayesian score is above the estimated best cutoff value from minimizing the false positive and false negative rate.
Probability: The esimated probability that the sample is in the positive category. This assumes that the Bayesian score follows a normal distribution and is different from the prediction using a cutoff.
Enrichment: An estimate of enrichment, that is, the increased likelihood (versus random) of this sample being in the category.
Bayesian Score: The standard Laplacian-modified Bayesian score.
Mahalanobis Distance: The Mahalanobis distance (MD) is the distance to the center of the training data. The larger the MD, the less trustworthy the prediction.
Mahalanobis Distance p-value: The p-value gives the fraction of training data with an MD greater than or equal to the one for the given sample, assuming normally distributed data. The smaller the p-value, the less trustworthy the prediciton. For highly non-normal X properties (e.g., fingerprints), the MD p-value is wildly inaccurate.

Structural Similar Compounds

Name	Rifampin	Rifabutin	Erythromycin
Structure	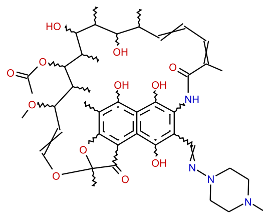	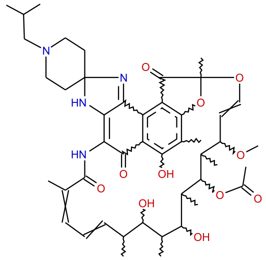	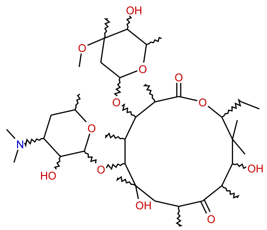
Actual Endpoint	Non-Carcinogen	Non-Carcinogen	Non-Carcinogen
Predicted Endpoint	Non-Carcinogen	Non-Carcinogen	Non-Carcinogen
Distance	0.461	0.615	0.829
Reference	US FDA (Centre for Drug Eval.& Res./Off. Testing & Res.) Sept. 1997	US FDA (Centre for Drug Eval.& Res./Off. Testing & Res.) Sept. 1997	US FDA (Centre for Drug Eval.& Res./Off. Testing & Res.) Sept. 1997

Model Applicability

Unknown features are fingerprint features in the query molecule, but not found in the training set.

1. All properties and OPS components are within expected ranges.

Feature Contribution

Top features for positive contribution

Fingerprint	Bit/Smiles	Feature Structure	Score	Carcinogen in training set
SCFP_6	-1104485307	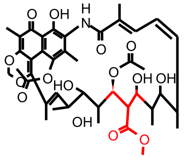 [*]C([*])C(C([*])[*]) C(=O)OC	0.615	5 out of 7

SCFP_6	97110830	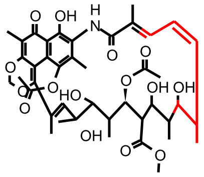 [*]C([*])C(C)\C=C/C=[<br>*]	0.536	4 out of 6
SCFP_6	1416226694	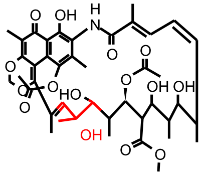 [*]C(=[*])[C@]1(O)C[*<br>][*]C1([*])[*]	0.438	8 out of 15

Top Features for negative contribution

**Fingerprint**	**Bit/Smiles**	**Feature Structure**	**Score**	**Carcinogen in training set**
SCFP_6	-92192314	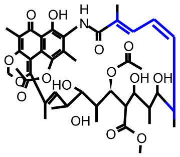 [*]C([*])\C=C/C=C([*]<br>)[*]	-0.674	0 out of 3
SCFP_6	-2042331623	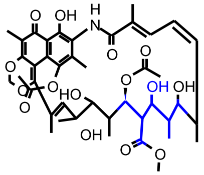 [*]C([*])C(C)C(O)C(C(<br>[*])[*])C(=[*])[*]	-0.674	0 out of 3
SCFP_6	876914673	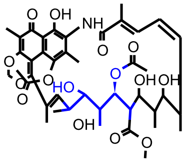 [*]O[C@@H](C([*])[*])<br>C(C)C(O)C([*])([*])[<br>*]	-0.674	0 out of 3

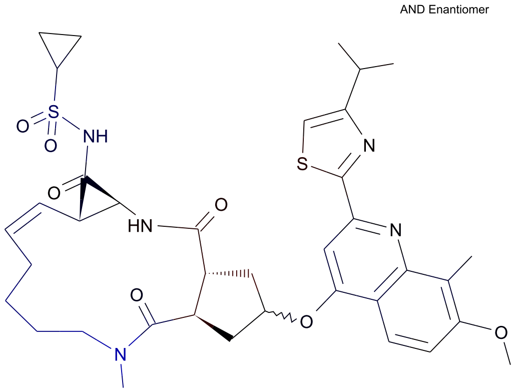

$C_{38}H_{47}N_5O_7S_2$
Molecular Weight: 749.93907
ALogP: 4.796
Rotatable Bonds: 8
Acceptors: 9
Donors: 2

Structural Similar Compounds

Name	Reserpine	Deserpidine	Bromocriptine
Structure	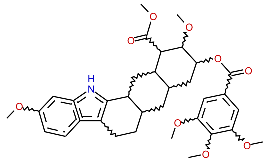	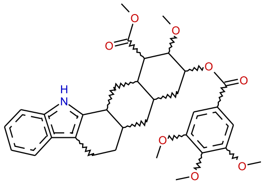	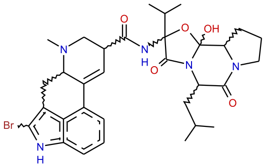
Actual Endpoint	Carcinogen	Carcinogen	Non-Carcinogen
Predicted Endpoint	Carcinogen	Carcinogen	Non-Carcinogen
Distance	0.771	0.812	0.842
Reference	US FDA (Centre for Drug Eval.& Res./Off. Testing & Res.) Sept. 1997	US FDA (Centre for Drug Eval.& Res./Off. Testing & Res.) Sept. 1997	US FDA (Centre for Drug Eval.& Res./Off. Testing & Res.) Sept. 1997

Model Prediction

Prediction: Non-Carcinogen
Probability: 0.231
Enrichment: 0.690
Bayesian Score: -5.429
Mahalanobis Distance: 20.335
Mahalanobis Distance p-value: 1.2e-019

Prediction: Positive if the Bayesian score is above the estimated best cutoff value from minimizing the false positive and false negative rate.
Probability: The esimated probability that the sample is in the positive category. This assumes that the Bayesian score follows a normal distribution and is different from the prediction using a cutoff.
Enrichment: An estimate of enrichment, that is, the increased likelihood (versus random) of this sample being in the category.
Bayesian Score: The standard Laplacian-modified Bayesian score.
Mahalanobis Distance: The Mahalanobis distance (MD) is the distance to the center of the training data. The larger the MD, the less trustworthy the prediction.
Mahalanobis Distance p-value: The p-value gives the fraction of training data with an MD greater than or equal to the one for the given sample, assuming normally distributed data. The smaller the p-value, the less trustworthy the prediciton. For highly non-normal X properties (e.g., fingerprints), the MD p-value is wildly inaccurate.

Model Applicability

Unknown features are fingerprint features in the query molecule, but not found in the training set.

1. OPS PC5 out of range. Value: 7.7766. Training min, max, SD, explained variance: -4.0702, 6.5927, 2.146, 0.0429.
2. OPS PC22 out of range. Value: 3.5288. Training min, max, SD, explained variance: -3.2994, 3.2573, 1.197, 0.0134.

Feature Contribution

Top features for positive contribution

Fingerprint	Bit/Smiles	Feature Structure	Score	Carcinogen in training set
SCFP_6	-1903488337	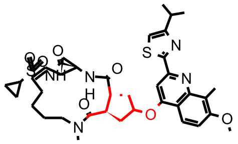 [*]C1[*]C([*])([*])[C@@H](CC1O)C(=[*])[*]	0.603	2 out of 2

SCFP_6	-1379673609	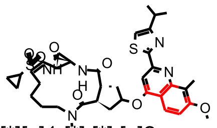 [*][c]1:[*]:[*]:[c]2:[*]:[cH]:[cH]:[cH]:[c]:1:2	0.526	11 out of 19
SCFP_6	1310748454	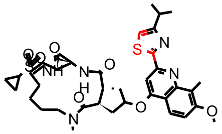 [*][c]1:[*]:[*]:[cH]:s:1	0.437	7 out of 13
**Top Features for negative contribution**				
**Fingerprint**	**Bit/Smiles**	**Feature Structure**	**Score**	**Carcinogen in training set**
SCFP_6	182902497	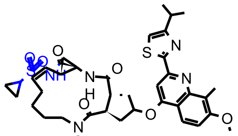 [*]NS(=O)(=O)C1[*][*]1	-0.825	0 out of 4
SCFP_6	306578635	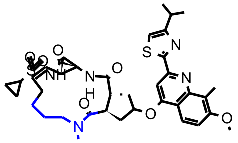 [*][C@@H]1CCCN1C(=[*])[*]	-0.825	0 out of 4
SCFP_6	1260369147	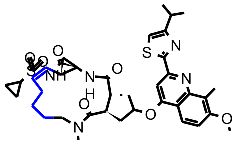 [*]CCC\C=C/[*]	-0.674	0 out of 3

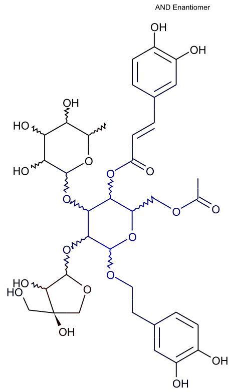

$C_{36}H_{46}O_{20}$

Molecular Weight: 798.73843

ALogP: -0.546

Rotatable Bonds: 16

Acceptors: 20

Donors: 10

Model Prediction

Prediction: Mild

Probability: 0.063

Enrichment: 0.172

Bayesian Score: -10.100

Mahalanobis Distance: 20.805

Mahalanobis Distance p-value: 9.44e-040

Prediction: Positive if the Bayesian score is above the estimated best cutoff value from minimizing the false positive and false negative rate.
Probability: The esimated probability that the sample is in the positive category. This assumes that the Bayesian score follows a normal distribution and is different from the prediction using a cutoff.
Enrichment: An estimate of enrichment, that is, the increased likelihood (versus random) of this sample being in the category.
Bayesian Score: The standard Laplacian-modified Bayesian score.
Mahalanobis Distance: The Mahalanobis distance (MD) is the distance to the center of the training data. The larger the MD, the less trustworthy the prediction.
Mahalanobis Distance p-value: The p-value gives the fraction of training data with an MD greater than or equal to the one for the given sample, assuming normally distributed data. The smaller the p-value, the less trustworthy the prediciton. For highly non-normal X properties (e.g., fingerprints), the MD p-value is wildly inaccurate.

Structural Similar Compounds

Name	2,2'-Stilbenedisulfonic acid, 4,4'-bis((4-(2-hydroxyethyl)amino-6-(p-sulfoanilino)-s- triazin-2-yl)amino)-, tetrasodium salt	1,5-Naphthalenedisulfonic acid, 2-(6-(4,6-dichloro-s-triazinyl)meth ylamino-1-hydroxy-3-sulfonaphthylazo)-	2,7-Naphthalenedisulfonic acid, 4-(4,6-dichloro-s-triazin-2-ylamino)-5-hydroxy-6-(2-hydroxy- 5-nitrophenylazo)-
Structure	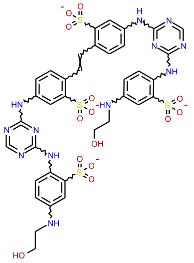	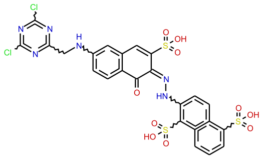	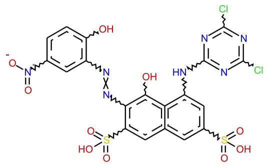
Actual Endpoint	Mild	Mild	Mild
Predicted Endpoint	Mild	Mild	Mild
Distance	1.634	1.729	1.827
Reference	MVCRB3 MVC-Report. (Stockholm, Sweden) No.1-2, 1972-73. Discontinued. Volu me(issue)/page/year: 2,193,1973	28ZPAK "Sbornik Vysledku Toxixologickeho Vysetreni Latek A Pripravku," Marhol d, J.V., Institut Pro Vychovu Vedoucicn Pracovniku Chemickeho Prumyclu Praha, Cz echoslovakia, 1972 Volume(issue)/page/year: -,237,1	28ZPAK "Sbornik Vysledku Toxixologickeho Vysetreni Latek A Pripravku," Marhol d, J.V., Institut Pro Vychovu Vedoucicn Pracovniku Chemickeho Prumyclu Praha, Cz echoslovakia, 1972 Volume(issue)/page/year: -,234,1

Model Applicability

Unknown features are fingerprint features in the query molecule, but not found in the training set.

1. OPS PC3 out of range. Value: 9.2098. Training min, max, SD, explained variance: -6.5795, 8.8354, 2.007, 0.0544.
2. OPS PC7 out of range. Value: 7.2108. Training min, max, SD, explained variance: -4.7717, 5.3182, 1.704, 0.0392.

Feature Contribution

Top features for positive contribution

Fingerprint	Bit/Smiles	Feature Structure	Score	Moderate_Severe in training set

FCFP_12	699500266	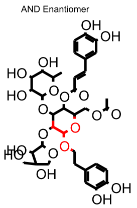 [*]OC(O[*])C([*])[*]	0.441	14 out of 24
FCFP_12	-428284881	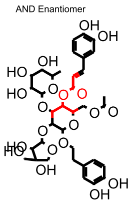 [*]C([*])C(OC(=O)C=[*])C([*])[*]	0.384	19 out of 35
FCFP_12	-415156552	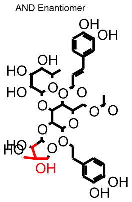 [*]C[C@@]1(O)C[*][*]C 1[*]	0.338	9 out of 17
**Top Features for negative contribution**				
**Fingerprint**	**Bit/Smiles**	**Feature Structure**	**Score**	**Moderate_Severe in training set**
FCFP_12	205247261	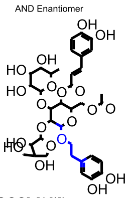 [*]C([*])OCC[c](:[*]) :[*]	-0.664	1 out of 8
FCFP_12	-145972974	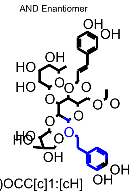 [*]C([*])OCC[c]1:[cH] :[cH]:[*]:[c]([*]):[cH]:1	-0.664	1 out of 8

FCFP_12	2023243256	AND Enantiomer 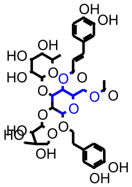 [*]OCC1OC([*])[*]C([*])C1O[*]	-0.543	0 out of 2

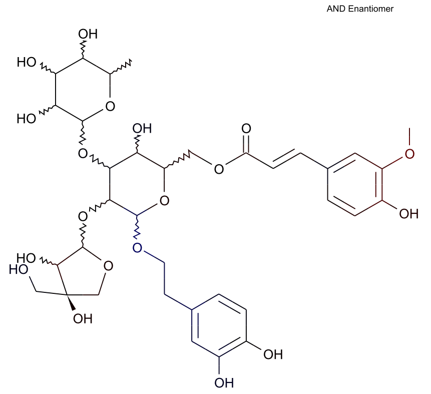

$C_{35}H_{46}O_{19}$

Molecular Weight: 770.72833

ALogP: -0.699

Rotatable Bonds: 15

Acceptors: 19

Donors: 10

Model Prediction

Prediction: Mild

Probability: 0.208

Enrichment: 0.564

Bayesian Score: -4.955

Mahalanobis Distance: 20.760

Mahalanobis Distance p-value: 1.58e-039

Prediction: Positive if the Bayesian score is above the estimated best cutoff value from minimizing the false positive and false negative rate.
Probability: The esimated probability that the sample is in the positive category. This assumes that the Bayesian score follows a normal distribution and is different from the prediction using a cutoff.
Enrichment: An estimate of enrichment, that is, the increased likelihood (versus random) of this sample being in the category.
Bayesian Score: The standard Laplacian-modified Bayesian score.
Mahalanobis Distance: The Mahalanobis distance (MD) is the distance to the center of the training data. The larger the MD, the less trustworthy the prediction.
Mahalanobis Distance p-value: The p-value gives the fraction of training data with an MD greater than or equal to the one for the given sample, assuming normally distributed data. The smaller the p-value, the less trustworthy the prediciton. For highly non-normal X properties (e.g., fingerprints), the MD p-value is wildly inaccurate.

Structural Similar Compounds

**Name**	**1,5-Naphthalenedisulfonic acid, 2-(6-(4,6-dichloro-s-triazinyl)meth ylamino-1-hydroxy-3-sulfonaphthylazo)-**	**2,2'-Stilbenedisulfonic acid, 4,4'-bis((4-(2-hydroxyethyl)amino-6-(p-sulfoanilino)-s- triazin-2-yl)amino)-, tetrasodium salt**	**2,7-Naphthalenedisulfonic acid, 4-(4,6-dichloro-s-triazin-2-ylamino)-5-hydroxy-6-(2-hydroxy- 5-nitrophenylazo)-**
Structure	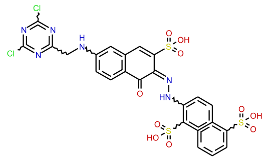	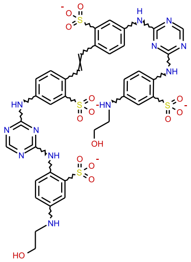	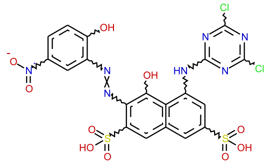
Actual Endpoint	Mild	Mild	Mild
Predicted Endpoint	Mild	Mild	Mild
Distance	1.686	1.758	1.780
Reference	28ZPAK "Sbornik Vysledku Toxixologickeho Vysetreni Latek A Pripravku," Marhol d, J.V., Institut Pro Vychovu Vedoucicn Pracovniku Chemickeho Prumyclu Praha, Cz echoslovakia, 1972 Volume(issue)/page/year: -,237,1	MVCRB3 MVC-Report. (Stockholm, Sweden) No.1-2, 1972-73. Discontinued. Volu me(issue)/page/year: 2,193,1973	28ZPAK "Sbornik Vysledku Toxixologickeho Vysetreni Latek A Pripravku," Marhol d, J.V., Institut Pro Vychovu Vedoucicn Pracovniku Chemickeho Prumyclu Praha, Cz echoslovakia, 1972 Volume(issue)/page/year: -,234,1

Model Applicability

Unknown features are fingerprint features in the query molecule, but not found in the training set.

1. All properties and OPS components are within expected ranges.

Feature Contribution

Top features for positive contribution

Fingerprint	Bit/Smiles	Feature Structure	Score	Moderate_Severe in training set

FCFP_12	699500266	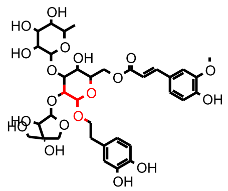 [*]OC(O[*])C([*])[*]	0.441	14 out of 24
FCFP_12	-1977641857	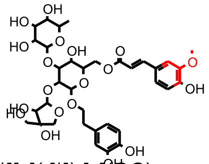 [*][c](:[*]):[c](OC):[cH]:[*]	0.416	18 out of 32
FCFP_12	-2106701143	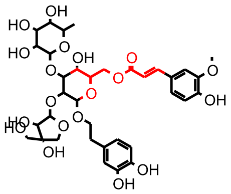 [*]OC(COC(=O)\C=C\[*])C([*])[*]	0.385	1 out of 1

Top Features for negative contribution

**Fingerprint**	**Bit/Smiles**	**Feature Structure**	**Score**	**Moderate_Severe in training set**
FCFP_12	-145972974	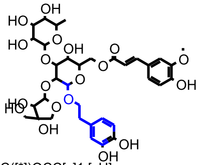 [*]C([*])OCC[c]1:[cH]:[cH]:[*]:[c]([*]):[cH]:1	-0.664	1 out of 8
FCFP_12	205247261	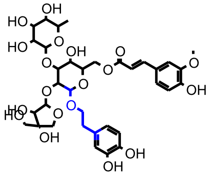 [*]C([*])OCC[c](:[*]):[*]	-0.664	1 out of 8

FCFP_12	-1272709286	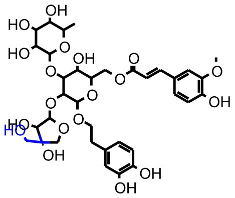 [*]C([*])([*])CO	-0.475	18 out of 82

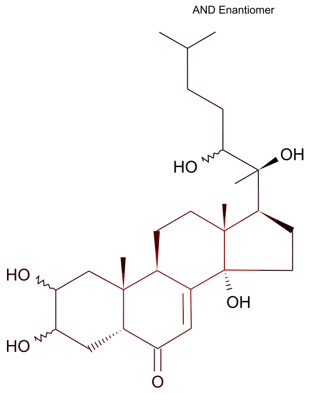

$C_{27}H_{44}O_6$
Molecular Weight: 464.63466
ALogP: 2.489
Rotatable Bonds: 5
Acceptors: 6
Donors: 5

Model Prediction

Prediction: Moderate_Severe
Probability: 0.535
Enrichment: 1.452
Bayesian Score: 2.989
Mahalanobis Distance: 15.122
Mahalanobis Distance p-value: 4.78e-015

Prediction: Positive if the Bayesian score is above the estimated best cutoff value from minimizing the false positive and false negative rate.
Probability: The esimated probability that the sample is in the positive category. This assumes that the Bayesian score follows a normal distribution and is different from the prediction using a cutoff.
Enrichment: An estimate of enrichment, that is, the increased likelihood (versus random) of this sample being in the category.
Bayesian Score: The standard Laplacian-modified Bayesian score.
Mahalanobis Distance: The Mahalanobis distance (MD) is the distance to the center of the training data. The larger the MD, the less trustworthy the prediction.
Mahalanobis Distance p-value: The p-value gives the fraction of training data with an MD greater than or equal to the one for the given sample, assuming normally distributed data. The smaller the p-value, the less trustworthy the prediciton. For highly non-normal X properties (e.g., fingerprints), the MD p-value is wildly inaccurate.

Structural Similar Compounds

Name	Anthraquinone, 2-bromo-1,8-diamino-4,5-dihydroxy-	Anthraquinone, 1,4,5,8-tetrahydroxy-	C.I. Fluorescent Brightening Agent 24
Structure	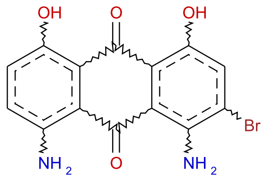	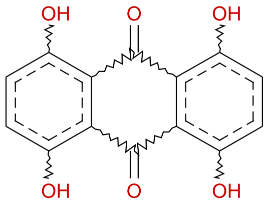	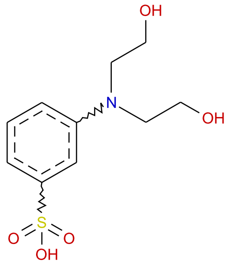
Actual Endpoint	Mild	Mild	Mild
Predicted Endpoint	Mild	Mild	Mild
Distance	0.978	1.076	1.146
Reference	28ZPAK "Sbornik Vysledku Toxixologickeho Vysetreni Latek A Pripravku," Marhol d, J.V., Institut Pro Vychovu Vedoucicn Pracovniku Chemickeho Prumyclu Praha, Cz echoslovakia, 1972 Volume(issue)/page/year: -,244,1	85JCAE "Prehled Prumyslove Toxikologie; Organicke Latky," Marhold, J., Prague , Czechoslovakia, Avicenum, 1986 Volume(issue)/page/year: -,655,1986	MVCRB3 MVC-Report. (Stockholm, Sweden) No.1-2, 1972-73. Discontinued. Volu me(issue)/page/year: 2,193,1973

Model Applicability

Unknown features are fingerprint features in the query molecule, but not found in the training set.

1. OPS PC7 out of range. Value: 5.8386. Training min, max, SD, explained variance: -4.7717, 5.3182, 1.704, 0.0392.

Feature Contribution

Top features for positive contribution

Fingerprint	Bit/Smiles	Feature Structure	Score	Moderate_Severe in training set

FCFP_12	1544504884	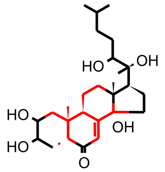 [*]C[C@@]1(C)[C@@H]([*])[*]C=C2[C@@H]1CC[*]C2([*])[*]	0.579	4 out of 5
FCFP_12	436886043	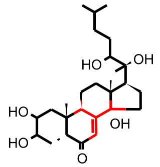 [*]\C=C(/C([*])[*])\C([*])([*])[*]	0.503	68 out of 113
FCFP_12	-415245925	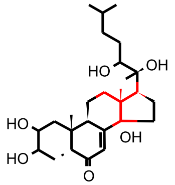 [*]C[C@]1(C)[C@@H]([*])[*][*]C1([*])[*]	0.498	22 out of 36
**Top Features for negative contribution**				
**Fingerprint**	**Bit/Smiles**	**Feature Structure**	**Score**	**Moderate_Severe in training set**
FCFP_12	3	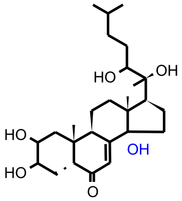 [*]O	-0.234	58 out of 204
FCFP_12	1070061035	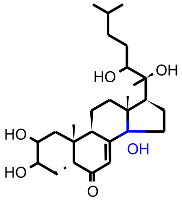 [*]C([*])O	-0.144	47 out of 151

FCFP_12	1	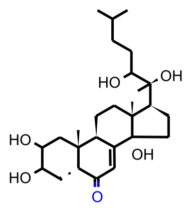[*]O[*]	0.000	269 out of 741

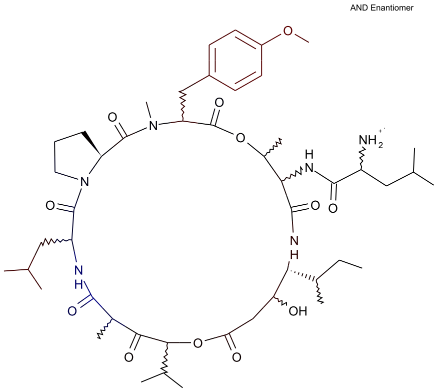

$C_{49}H_{79}N_6O_{12}$
Molecular Weight: 944.18455
ALogP: 3.176
Rotatable Bonds: 13
Acceptors: 12
Donors: 5

Model Prediction

Prediction: Mild
Probability: 0.331
Enrichment: 0.900
Bayesian Score: -1.964
Mahalanobis Distance: 19.791
Mahalanobis Distance p-value: 8.38e-035

Prediction: Positive if the Bayesian score is above the estimated best cutoff value from minimizing the false positive and false negative rate.
Probability: The esimated probability that the sample is in the positive category. This assumes that the Bayesian score follows a normal distribution and is different from the prediction using a cutoff.
Enrichment: An estimate of enrichment, that is, the increased likelihood (versus random) of this sample being in the category.
Bayesian Score: The standard Laplacian-modified Bayesian score.
Mahalanobis Distance: The Mahalanobis distance (MD) is the distance to the center of the training data. The larger the MD, the less trustworthy the prediction.
Mahalanobis Distance p-value: The p-value gives the fraction of training data with an MD greater than or equal to the one for the given sample, assuming normally distributed data. The smaller the p-value, the less trustworthy the prediciton. For highly non-normal X properties (e.g., fingerprints), the MD p-value is wildly inaccurate.

Structural Similar Compounds

Name	1,5-Naphthalenedisulfonic acid, 2-(6-(4,6-dichloro-s-triazinyl)meth ylamino-1-hydroxy-3-sulfonaphthylazo)-	2,7-Naphthalenedisulfonic acid, 4-(4,6-dichloro-s-triazin-2-ylamino)-5-hydroxy-6-(2-hydroxy- 5-nitrophenylazo)-	Anthraquinone, 3-methoxy-5,4'-iminobis(1-benzamido-
Structure	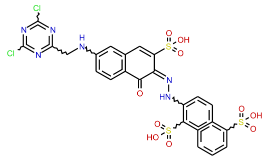	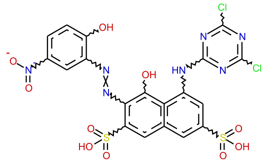	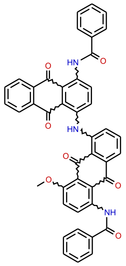
Actual Endpoint	Mild	Mild	Moderate_Severe
Predicted Endpoint	Mild	Mild	Moderate_Severe
Distance	1.212	1.403	1.467
Reference	28ZPAK "Sbornik Vysledku Toxixologickeho Vysetreni Latek A Pripravku," Marhol d, J.V., Institut Pro Vychovu Vedoucicn Pracovniku Chemickeho Prumyclu Praha, Cz echoslovakia, 1972 Volume(issue)/page/year: -,237,1	28ZPAK "Sbornik Vysledku Toxixologickeho Vysetreni Latek A Pripravku," Marhol d, J.V., Institut Pro Vychovu Vedoucicn Pracovniku Chemickeho Prumyclu Praha, Cz echoslovakia, 1972 Volume(issue)/page/year: -,234,1	28ZPAK "Sbornik Vysledku Toxixologickeho Vysetreni Latek A Pripravku," Marhol d, J.V., Institut Pro Vychovu Vedoucicn Pracovniku Chemickeho Prumyclu Praha, Cz echoslovakia, 1972 Volume(issue)/page/year: -,114,1

Model Applicability

Unknown features are fingerprint features in the query molecule, but not found in the training set.

1. OPS PC7 out of range. Value: 6.967. Training min, max, SD, explained variance: -4.7717, 5.3182, 1.704, 0.0392.
2. OPS PC23 out of range. Value: 4.2396. Training min, max, SD, explained variance: -3.6258, 3.7453, 0.9755, 0.0128.
3. Unknown FCFP_2 feature: 10: [*][NH2+][*]
4. Unknown FCFP_2 feature: 18028531: [*]C([*])[NH2+]C
5. Unknown FCFP_2 feature: -1817836174: [*]CC([NH2+][*])C(=[*])[*]
6. Unknown FCFP_2 feature: 136418580: [*][NH2+]C

Feature Contribution

Top features for positive contribution

Fingerprint	Bit/Smiles	Feature Structure	Score	Moderate_Severe in training set
FCFP_12	-1870530637	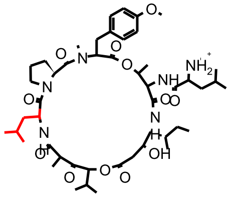<br>[*]CCC(C)C	0.448	22 out of 38
FCFP_12	1985089045	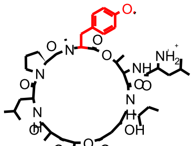<br>[*]O[c]1:[cH]:[cH]:[c](CC([*])[*]):[cH]:[cH]:1	0.435	5 out of 8
FCFP_12	-9847677	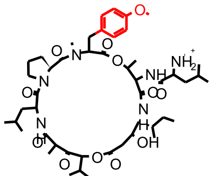<br>[*][c]1:[cH]:[cH]:[c] (OC):[cH]:[cH]:1	0.419	6 out of 10

Top Features for negative contribution

Fingerprint	Bit/Smiles	Feature Structure	Score	Moderate_Severe in training set
FCFP_12	-885550502	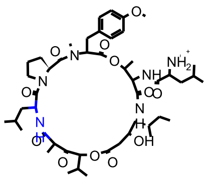<br>[*]C([*])NC(=[*])[*]	-1.050	2 out of 21

FCFP_12	566058135	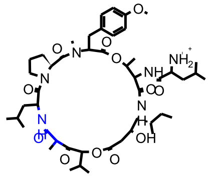 [*]NC(=O)C([*])[*]	-0.909	1 out of 11
FCFP_12	-950918369	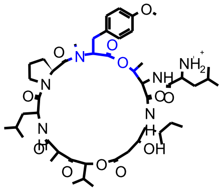 [*]CC(N([*])[*])C(=O)OC([*])[*]	-0.543	0 out of 2

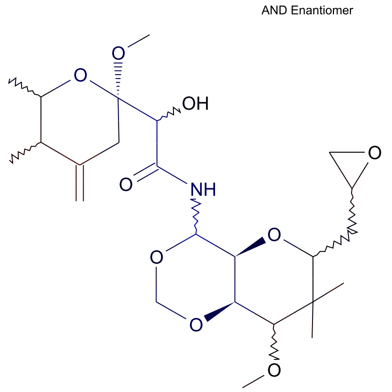

$C_{24}H_{39}NO_9$
Molecular Weight: 485.56776
ALogP: 0.267
Rotatable Bonds: 7
Acceptors: 9
Donors: 2

Model Prediction

Prediction: Mild
Probability: 0.143
Enrichment: 0.390
Bayesian Score: -6.802
Mahalanobis Distance: 13.695
Mahalanobis Distance p-value: 2.47e-010

Prediction: Positive if the Bayesian score is above the estimated best cutoff value from minimizing the false positive and false negative rate.
Probability: The esimated probability that the sample is in the positive category. This assumes that the Bayesian score follows a normal distribution and is different from the prediction using a cutoff.
Enrichment: An estimate of enrichment, that is, the increased likelihood (versus random) of this sample being in the category.
Bayesian Score: The standard Laplacian-modified Bayesian score.
Mahalanobis Distance: The Mahalanobis distance (MD) is the distance to the center of the training data. The larger the MD, the less trustworthy the prediction.
Mahalanobis Distance p-value: The p-value gives the fraction of training data with an MD greater than or equal to the one for the given sample, assuming normally distributed data. The smaller the p-value, the less trustworthy the prediciton. For highly non-normal X properties (e.g., fingerprints), the MD p-value is wildly inaccurate.

Structural Similar Compounds

Name	Pregna-1,4-diene-3,20-dione, 21-(acetyloxy)-11-hydroxy-6-methyl-17- (1-oxopropoxy)-, (6-alpha,11-beta)-	2-Anthracenesulfonic acid, 1-amino-9,10-dihydro-9,10-dioxo-4-(2,4,6 -trimethylanilino)-, monosodium salt	Benzenesulfonic acid, 2,2'-(1,4-anthraquinonylenediimino)bis(5-meth yl-, disodium salt
Structure	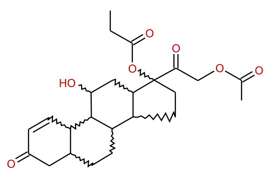	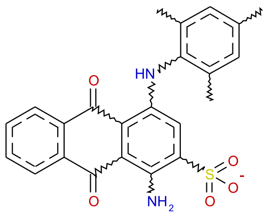	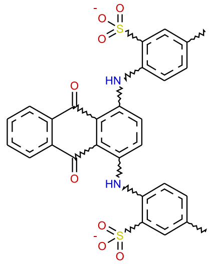
Actual Endpoint	Mild	Mild	Mild
Predicted Endpoint	Mild	Mild	Mild
Distance	0.815	0.852	0.951
Reference	YACHDS Yakuri to Chiryo. Pharmacology and Therapeutics. (Raifu Saiensu Shup pan K.K., 2-5-13, Yaesu, Chuo-ku, Tokyo 104, Japan) V.1- 1972- Volume(issue) /page/year: 19,3103,1991	85JCAE "Prehled Prumyslove Toxikologie; Organicke Latky," Marhold, J., Prague , Czechoslovakia, Avicenum, 1986 Volume(issue)/page/year: -,1327,1986	85JCAE "Prehled Prumyslove Toxikologie; Organicke Latky," Marhold, J., Prague , Czechoslovakia, Avicenum, 1986 Volume(issue)/page/year: -,1330,1986

Model Applicability

Unknown features are fingerprint features in the query molecule, but not found in the training set.

1. OPS PC23 out of range. Value: 5.354. Training min, max, SD, explained variance: -3.6258, 3.7453, 0.9755, 0.0128.
2. Unknown FCFP_2 feature: 699559848: [*]NC(O[*])C([*])[*]

Feature Contribution

Top features for positive contribution

Fingerprint	Bit/Smiles	Feature Structure	Score	Moderate_Severe in training set

FCFP_12	436886043	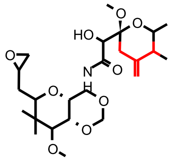 [*]\C=C(/C([*])[*])\C([*])([*])[*]	0.503	68 out of 113
FCFP_12	-415245925	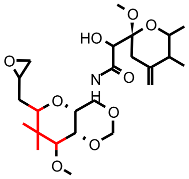 [*]C[C@]1(C)[C@@H]([*])[*][*]C1([*])[*]	0.498	22 out of 36
FCFP_12	-1218582944	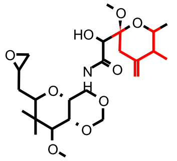 [*]C1[*]C([*])([*])CC(=C)C1C	0.385	1 out of 1

Top Features for negative contribution

Fingerprint	Bit/Smiles	Feature Structure	Score	Moderate_Severe in training set
FCFP_12	1327594201	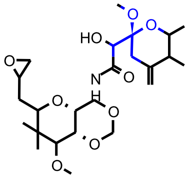 [*]CC(O[*])(O[*])C([*])[*]	-1.260	0 out of 7
FCFP_12	-885550502	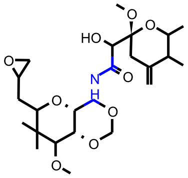 [*]C([*])NC(=[*])[*]	-1.050	2 out of 21

FCFP_12	566058135	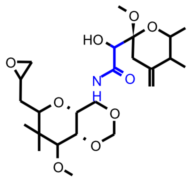 [*]NC(=O)C([*])[*]	-0.909	1 out of 11

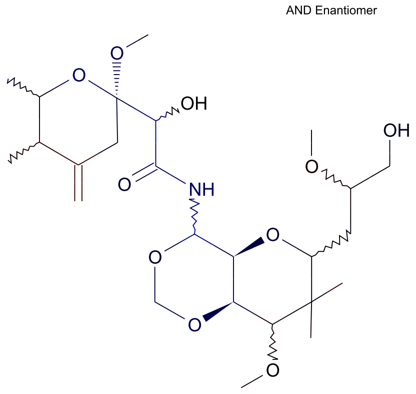

$C_{25}H_{43}NO_{10}$
Molecular Weight: 517.60962
ALogP: -9.1e-002
Rotatable Bonds: 9
Acceptors: 10
Donors: 3

Structural Similar Compounds

Name	Benzenesulfonic acid, 2,2'-(1,4-anthraquinonylenediimino)bis(5-meth yl-, disodium salt	2-Anthracenesulfonic acid, 1-amino-9,10-dihydro-9,10-dioxo-4-(2,4,6 -trimethylanilino)-, monosodium salt	Fumaric acid, bis(3-allyloxy-2-hydroxypropyl) ester
Structure	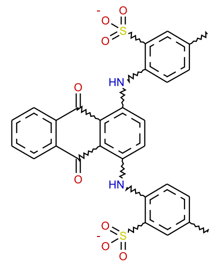	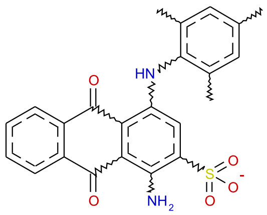	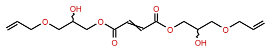
Actual Endpoint	Mild	Mild	Mild
Predicted Endpoint	Mild	Mild	Mild
Distance	0.927	1.010	1.028
Reference	85JCAE "Prehled Prumyslove Toxikologie; Organicke Latky," Marhold, J., Prague , Czechoslovakia, Avicenum, 1986 Volume(issue)/page/year: -,1330,1986	85JCAE "Prehled Prumyslove Toxikologie; Organicke Latky," Marhold, J., Prague , Czechoslovakia, Avicenum, 1986 Volume(issue)/page/year: -,1327,1986	85JCAE "Prehled Prumyslove Toxikologie; Organicke Latky," Marhold, J., Prague , Czechoslovakia, Avicenum, 1986 Volume(issue)/page/year: -,646,1986

Model Prediction

Prediction: Mild
Probability: 0.122
Enrichment: 0.332
Bayesian Score: -7.509
Mahalanobis Distance: 14.139
Mahalanobis Distance p-value: 1.02e-011

Prediction: Positive if the Bayesian score is above the estimated best cutoff value from minimizing the false positive and false negative rate.
Probability: The esimated probability that the sample is in the positive category. This assumes that the Bayesian score follows a normal distribution and is different from the prediction using a cutoff.
Enrichment: An estimate of enrichment, that is, the increased likelihood (versus random) of this sample being in the category.
Bayesian Score: The standard Laplacian-modified Bayesian score.
Mahalanobis Distance: The Mahalanobis distance (MD) is the distance to the center of the training data. The larger the MD, the less trustworthy the prediction.
Mahalanobis Distance p-value: The p-value gives the fraction of training data with an MD greater than or equal to the one for the given sample, assuming normally distributed data. The smaller the p-value, the less trustworthy the prediciton. For highly non-normal X properties (e.g., fingerprints), the MD p-value is wildly inaccurate.

Model Applicability

Unknown features are fingerprint features in the query molecule, but not found in the training set.

1. OPS PC7 out of range. Value: 5.4806. Training min, max, SD, explained variance: -4.7717, 5.3182, 1.704, 0.0392.
2. OPS PC23 out of range. Value: 4.0785. Training min, max, SD, explained variance: -3.6258, 3.7453, 0.9755, 0.0128.
3. Unknown FCFP_2 feature: 699559848: [*]NC(O[*])C([*])[*]

Feature Contribution

Top features for positive contribution

Fingerprint	Bit/Smiles	Feature Structure	Score	Moderate_Severe in training set

FCFP_12	436886043	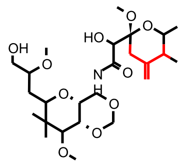 [*]\C=C(/C([*])[*])\C([*])([*])[*]	0.503	68 out of 113
FCFP_12	-415245925	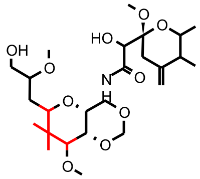 [*]C[C@]1(C)[C@@H]([*])[*][*]C1([*])[*]	0.498	22 out of 36
FCFP_12	-1218582944	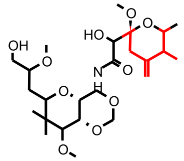 [*]C1[*]C([*])([*])CC(=C)C1C	0.385	1 out of 1

Top Features for negative contribution

Fingerprint	Bit/Smiles	Feature Structure	Score	Moderate_Severe in training set
FCFP_12	1327594201	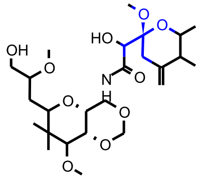 [*]CC(O[*])(O[*])C([*])[*]	-1.260	0 out of 7
FCFP_12	-885550502	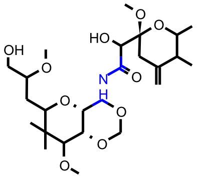 [*]C([*])NC(=[*])[*]	-1.050	2 out of 21

FCFP_12	566058135	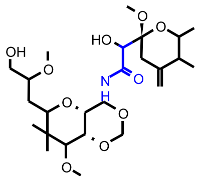<br>[*]NC(=O)C([*])[*]	-0.909	1 out of 11

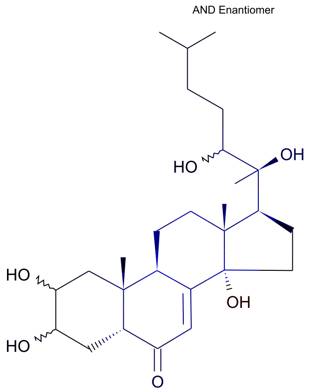

$C_{27}H_{44}O_6$
Molecular Weight: 464.63466
ALogP: 2.489
Rotatable Bonds: 5
Acceptors: 6
Donors: 5

Structural Similar Compounds

Name	Benzenesulfonamide, 4-amino-N-(5,6-dimethoxy-4-pyrimidinyl)-	1,2-Ethanediamine, N-(2-aminoethyl)-N'-(2-((2-aminoethyl)amino)ethy l)-	Anthraquinone, 3-methoxy-5,4'-iminobis(1-benzamido-
Structure	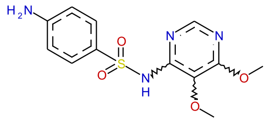	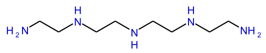	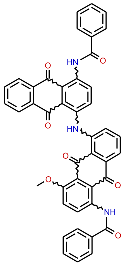
Actual Endpoint	Moderate	Severe	Moderate
Predicted Endpoint	Moderate	Severe	Moderate
Distance	1.387	1.389	1.406
Reference	FCTXAV 14,307,76	85JCAE "Prehled Prumyslove Toxikologie; Organicke Latky," Marhold, J., Prague , Czechoslovakia, Avicenum, 1986 Volume(issue)/page/year: -,446,1986	28ZPAK "Sbornik Vysledku Toxixologickeho Vysetreni Latek A Pripravku," Marhol d, J.V., Institut Pro Vychovu Vedoucicn Pracovniku Chemickeho Prumyclu Praha, Cz echoslovakia, 1972 Volume(issue)/page/year: -,114,1

Model Prediction

Prediction: Moderate

Probability: 0.265

Enrichment: 0.599

Bayesian Score: -6.924

Mahalanobis Distance: 14.170

Mahalanobis Distance p-value: 3.38e-013

Prediction: Positive if the Bayesian score is above the estimated best cutoff value from minimizing the false positive and false negative rate.
Probability: The esimated probability that the sample is in the positive category. This assumes that the Bayesian score follows a normal distribution and is different from the prediction using a cutoff.
Enrichment: An estimate of enrichment, that is, the increased likelihood (versus random) of this sample being in the category.
Bayesian Score: The standard Laplacian-modified Bayesian score.
Mahalanobis Distance: The Mahalanobis distance (MD) is the distance to the center of the training data. The larger the MD, the less trustworthy the prediction.
Mahalanobis Distance p-value: The p-value gives the fraction of training data with an MD greater than or equal to the one for the given sample, assuming normally distributed data. The smaller the p-value, the less trustworthy the prediciton. For highly non-normal X properties (e.g., fingerprints), the MD p-value is wildly inaccurate.

Model Applicability

Unknown features are fingerprint features in the query molecule, but not found in the training set.

1. OPS PC13 out of range. Value: -3.5275. Training min, max, SD, explained variance: -3.4031, 3.5966, 1.166, 0.0215.

Feature Contribution

Top features for positive contribution

Fingerprint	Bit/Smiles	Feature Structure	Score	Severe in training set

SCFP_12	-1546614078	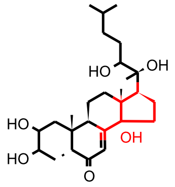 [*][C@H]1CC[C@@](O)(C (=[*])[*])C1([*])[*]	0.366	1 out of 1
SCFP_12	-1971196727	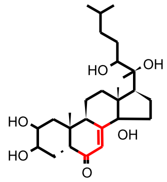 [*]C(=CC(=[*])[*])[*]	0.263	23 out of 45
SCFP_12	1416226694	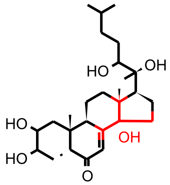 [*]C(=[*])[C@]1(O)C[*][*]C1([*])[*]	0.198	4 out of 8

Top Features for negative contribution

Fingerprint	Bit/Smiles	Feature Structure	Score	Severe in training set
SCFP_12	55434585	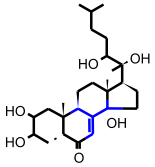 [*]\C=C(/C([*])[*])\C ([*])([*])[*]	-0.948	9 out of 64
SCFP_12	-608397454	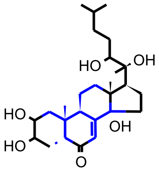 [*]C[C@@]1(C)[C@@H]([*])[*]C=C2[C@@H]1CC[*]C2([*])[*]	-0.936	0 out of 4

SCFP_12	-415245925	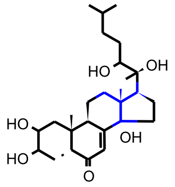<br><br>[*]C[C@]1(C)[C@@H]([*])[*][*]C1([*])[*]	-0.796	4 out of 26

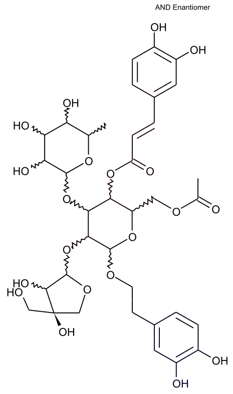

$C_{36}H_{46}O_{20}$
Molecular Weight: 798.73843
ALogP: -0.546
Rotatable Bonds: 16
Acceptors: 20
Donors: 10

Model Prediction

Prediction: Irritant
Probability: 0.978
Enrichment: 1.062
Bayesian Score: -0.216
Mahalanobis Distance: 20.746
Mahalanobis Distance p-value: 8.71e-041

Prediction: Positive if the Bayesian score is above the estimated best cutoff value from minimizing the false positive and false negative rate.
Probability: The esimated probability that the sample is in the positive category. This assumes that the Bayesian score follows a normal distribution and is different from the prediction using a cutoff.
Enrichment: An estimate of enrichment, that is, the increased likelihood (versus random) of this sample being in the category.
Bayesian Score: The standard Laplacian-modified Bayesian score.
Mahalanobis Distance: The Mahalanobis distance (MD) is the distance to the center of the training data. The larger the MD, the less trustworthy the prediction.
Mahalanobis Distance p-value: The p-value gives the fraction of training data with an MD greater than or equal to the one for the given sample, assuming normally distributed data. The smaller the p-value, the less trustworthy the prediciton. For highly non-normal X properties (e.g., fingerprints), the MD p-value is wildly inaccurate.

Structural Similar Compounds

Name	2,2'-Stilbenedisulfonic acid, 4,4'-bis((4-(2-hydroxyethyl)amino-6-(p-sulfoanilino)-s- triazin-2-yl)amino)-, tetrasodium salt	Amipurimycin, hydrate	1,5-Naphthalenedisulfonic acid, 2-(6-(4,6-dichloro-s-triazinyl)meth ylamino-1-hydroxy-3-sulfonaphthylazo)-
Structure	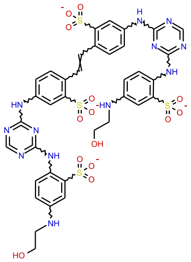	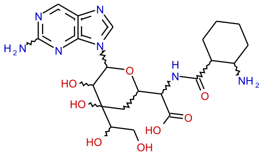	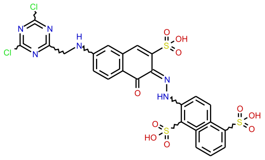
Actual Endpoint	Irritant	Irritant	Irritant
Predicted Endpoint	Non-Irritant	Non-Irritant	Non-Irritant
Distance	1.673	1.687	1.704
Reference	MVCRB3 MVC-Report. (Stockholm, Sweden) No.1-2, 1972-73. Discontinued. Volu me(issue)/page/year: 2,193,1973	JANTAJ Journal of Antibiotics. (Japan Antibiotics Research Assoc., 2-20-8 Ka miosaki, Shinagawa-ku, Tokyo, 141, Japan) V.2-5, 1948-52; V.21- 1968- Volume(i ssue)/page/year: 30,1,1977	28ZPAK "Sbornik Vysledku Toxixologickeho Vysetreni Latek A Pripravku," Marhol d, J.V., Institut Pro Vychovu Vedoucicn Pracovniku Chemickeho Prumyclu Praha, Cz echoslovakia, 1972 Volume(issue)/page/year: -,237,1

Model Applicability

Unknown features are fingerprint features in the query molecule, but not found in the training set.

1. OPS PC3 out of range. Value: 10.001. Training min, max, SD, explained variance: -8.0454, 9.6402, 2.076, 0.0545.

Feature Contribution

Top features for positive contribution				
Fingerprint	Bit/Smiles	Feature Structure	Score	Irritant in training set

FCFP_12	-1251367201	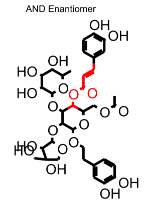<br>[*]\C=C\C(=O)OC([*])[<br>*]	0.086	31 out of 31
FCFP_12	654335567	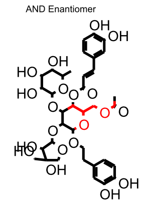<br>[*]OC(COC(=[*])[*])C(<br>[*])[*]	0.086	29 out of 29
FCFP_12	-415156552	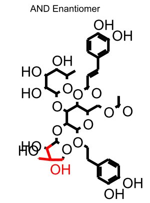<br>[*]C[C@@]1(O)C[*][*]C<br>1[*]	0.085	27 out of 27
**Top Features for negative contribution**				
**Fingerprint**	**Bit/Smiles**	**Feature Structure**	**Score**	**Irritant in training set**
FCFP_12	949015626	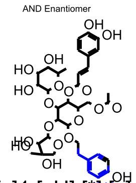<br>[*]C[c]1:[cH]:[*]:[c]<br>([*]):[c](O):[cH]:1	-0.222	2 out of 3
FCFP_12	-204034463	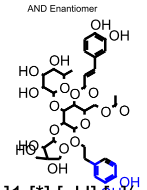<br>[*][c]1:[*]:[cH]:[c](<br>O):[c](O):[cH]:1	-0.222	2 out of 3

FCFP_12	7	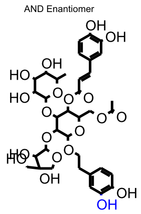 [*]O	-0.118	104 out of 128

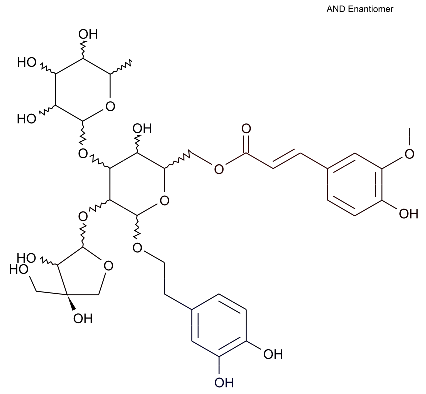

$C_{35}H_{46}O_{19}$
Molecular Weight: 770.72833
ALogP: -0.699
Rotatable Bonds: 15
Acceptors: 19
Donors: 10

Model Prediction

Prediction: Irritant
Probability: 0.996
Enrichment: 1.082
Bayesian Score: 0.219
Mahalanobis Distance: 20.464
Mahalanobis Distance p-value: 2.95e-039

Prediction: Positive if the Bayesian score is above the estimated best cutoff value from minimizing the false positive and false negative rate.
Probability: The esimated probability that the sample is in the positive category. This assumes that the Bayesian score follows a normal distribution and is different from the prediction using a cutoff.
Enrichment: An estimate of enrichment, that is, the increased likelihood (versus random) of this sample being in the category.
Bayesian Score: The standard Laplacian-modified Bayesian score.
Mahalanobis Distance: The Mahalanobis distance (MD) is the distance to the center of the training data. The larger the MD, the less trustworthy the prediction.
Mahalanobis Distance p-value: The p-value gives the fraction of training data with an MD greater than or equal to the one for the given sample, assuming normally distributed data. The smaller the p-value, the less trustworthy the prediciton. For highly non-normal X properties (e.g., fingerprints), the MD p-value is wildly inaccurate.

Structural Similar Compounds

Name	Amipurimycin, hydrate	1,5-Naphthalenedisulfonic acid, 2-(6-(4,6-dichloro-s-triazinyl)meth ylamino-1-hydroxy-3-sulfonaphthylazo)-	2,7-Naphthalenedisulfonic acid, 4-(4,6-dichloro-s-triazin-2-ylamino)-5-hydroxy-6-(2-hydroxy- 5-nitrophenylazo)-
Structure	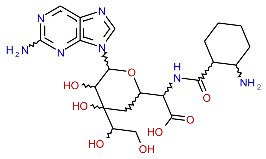	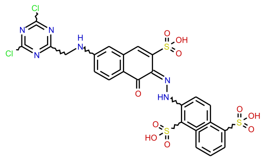	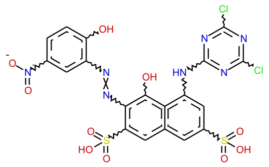
Actual Endpoint	Irritant	Irritant	Irritant
Predicted Endpoint	Non-Irritant	Non-Irritant	Non-Irritant
Distance	1.566	1.654	1.755
Reference	JANTAJ Journal of Antibiotics. (Japan Antibiotics Research Assoc., 2-20-8 Ka miosaki, Shinagawa-ku, Tokyo, 141, Japan) V.2-5, 1948-52; V.21- 1968- Volume(i ssue)/page/year: 30,1,1977	28ZPAK "Sbornik Vysledku Toxixologickeho Vysetreni Latek A Pripravku," Marhol d, J.V., Institut Pro Vychovu Vedoucicn Pracovniku Chemickeho Prumyclu Praha, Cz echoslovakia, 1972 Volume(issue)/page/year: -,237,1	28ZPAK "Sbornik Vysledku Toxixologickeho Vysetreni Latek A Pripravku," Marhol d, J.V., Institut Pro Vychovu Vedoucicn Pracovniku Chemickeho Prumyclu Praha, Cz echoslovakia, 1972 Volume(issue)/page/year: -,234,1

Model Applicability

Unknown features are fingerprint features in the query molecule, but not found in the training set.

1. OPS PC23 out of range. Value: -3.2619. Training min, max, SD, explained variance: -3.0369, 4.9599, 1.007, 0.0128.

Feature Contribution

Top features for positive contribution

Fingerprint	Bit/Smiles	Feature Structure	Score	Irritant in training set

FCFP_12	-1251367201	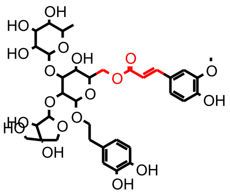 [*]\C=C\C(=O)OC([*])[*]	0.086	31 out of 31
FCFP_12	654335567	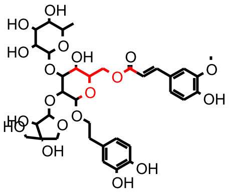 [*]OC(COC(=[*])[*])C([*])[*]	0.086	29 out of 29
FCFP_12	-415156552	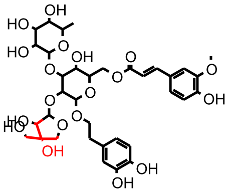 [*]C[C@@]1(O)C[*][*]C1[*]	0.085	27 out of 27

Top Features for negative contribution

Fingerprint	Bit/Smiles	Feature Structure	Score	Irritant in training set
FCFP_12	949015626	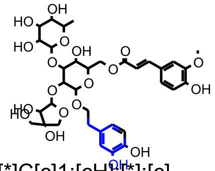 [*]C[c]1:[cH]:[*]:[c]([*]):[c](O):[cH]:1	-0.222	2 out of 3
FCFP_12	-204034463	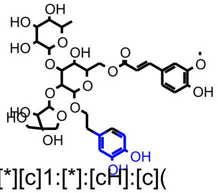 [*][c]1:[*]:[cH]:[c](O):[c](O):[cH]:1	-0.222	2 out of 3

FCFP_12	7	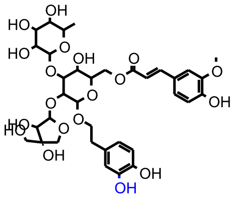 [*]O	-0.118	104 out of 128

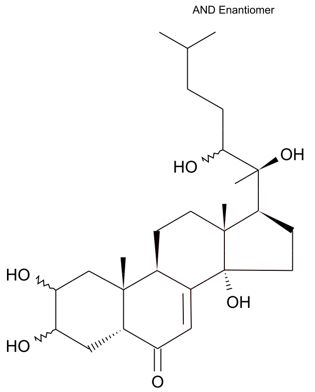

$C_{27}H_{44}O_6$
Molecular Weight: 464.63466
ALogP: 2.489
Rotatable Bonds: 5
Acceptors: 6
Donors: 5

Model Prediction

Prediction: Irritant
Probability: 0.998
Enrichment: 1.083
Bayesian Score: 0.573
Mahalanobis Distance: 15.638
Mahalanobis Distance p-value: 1.31e-016

Prediction: Positive if the Bayesian score is above the estimated best cutoff value from minimizing the false positive and false negative rate.
Probability: The esimated probability that the sample is in the positive category. This assumes that the Bayesian score follows a normal distribution and is different from the prediction using a cutoff.
Enrichment: An estimate of enrichment, that is, the increased likelihood (versus random) of this sample being in the category.
Bayesian Score: The standard Laplacian-modified Bayesian score.
Mahalanobis Distance: The Mahalanobis distance (MD) is the distance to the center of the training data. The larger the MD, the less trustworthy the prediction.
Mahalanobis Distance p-value: The p-value gives the fraction of training data with an MD greater than or equal to the one for the given sample, assuming normally distributed data. The smaller the p-value, the less trustworthy the prediciton. For highly non-normal X properties (e.g., fingerprints), the MD p-value is wildly inaccurate.

Structural Similar Compounds

Name	Anthraquinone, 2-bromo-1,8-diamino-4,5-dihydroxy-	Anthraquinone, 1,4,5,8-tetrahydroxy-	Anthraquinone, 1,5-diamino-4,8-dihydroxy-
Structure	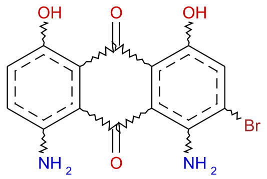	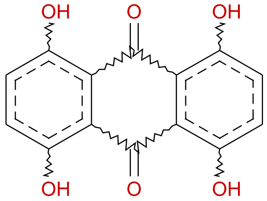	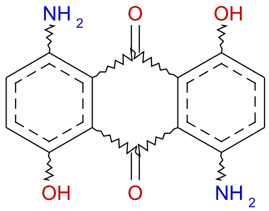
Actual Endpoint	Irritant	Irritant	Non-Irritant
Predicted Endpoint	Non-Irritant	Non-Irritant	Non-Irritant
Distance	0.977	1.082	1.118
Reference	28ZPAK "Sbornik Vysledku Toxixologickeho Vysetreni Latek A Pripravku," Marhol d, J.V., Institut Pro Vychovu Vedoucicn Pracovniku Chemickeho Prumyclu Praha, Cz echoslovakia, 1972 Volume(issue)/page/year: -,244,1	85JCAE "Prehled Prumyslove Toxikologie; Organicke Latky," Marhol, J., Prague , Czechoslovakia, Avicenum, 1986 Volume(issue)/page/year: -,655,1986	28ZPAK -,103,72

Model Applicability

Unknown features are fingerprint features in the query molecule, but not found in the training set.

1. All properties and OPS components are within expected ranges.

Feature Contribution

Top features for positive contribution				
Fingerprint	Bit/Smiles	Feature Structure	Score	Irritant in training set

FCFP_12	-1870530637	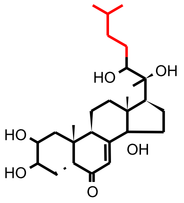<br><br>[*]CCC(C)C	0.087	44 out of 44
FCFP_12	-415156552	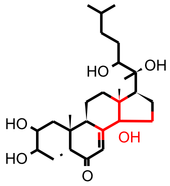<br><br>[*]C[C@@]1(O)C[*][*]C1[*]	0.085	27 out of 27
FCFP_12	-1742546106	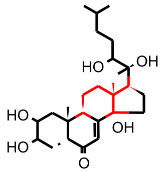<br><br>[*][C@@H]1[*][C@]2([*])[*][*][C@H]([*])[C@@]2(C)CC1	0.084	17 out of 17
**Top Features for negative contribution**				
**Fingerprint**	**Bit/Smiles**	**Feature Structure**	**Score**	**Irritant in training set**
FCFP_12	3	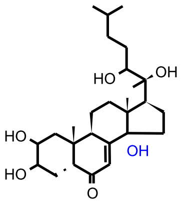<br><br>[*]O	-0.081	291 out of 345
FCFP_12	1002543125	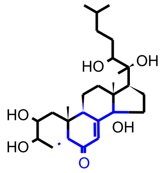<br><br>[*][C@@H]1[*][C@@H]([*])C(=CC1=O)C([*])([*])[*]	0.000	1 out of 1

FCFP_12	351491212	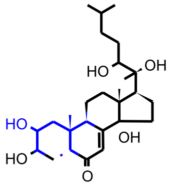 [*]C([*])[C@@]1(C)CC(O)C([*])[*][C@H]1[*]	0.000	1 out of 1

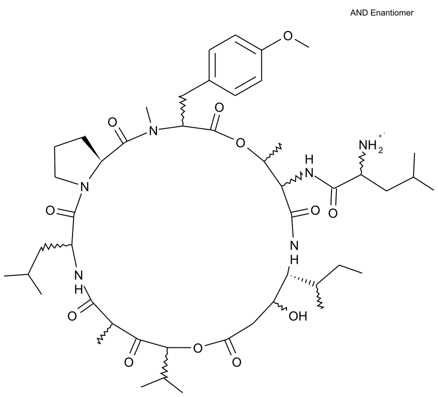

$C_{49}H_{79}N_6O_{12}$
Molecular Weight: 944.18455
ALogP: 3.176
Rotatable Bonds: 13
Acceptors: 12
Donors: 5

Model Prediction

Prediction: Irritant
Probability: 0.976
Enrichment: 1.060
Bayesian Score: -0.424
Mahalanobis Distance: 22.004
Mahalanobis Distance p-value: 7.91e-048

Prediction: Positive if the Bayesian score is above the estimated best cutoff value from minimizing the false positive and false negative rate.
Probability: The esimated probability that the sample is in the positive category. This assumes that the Bayesian score follows a normal distribution and is different from the prediction using a cutoff.
Enrichment: An estimate of enrichment, that is, the increased likelihood (versus random) of this sample being in the category.
Bayesian Score: The standard Laplacian-modified Bayesian score.
Mahalanobis Distance: The Mahalanobis distance (MD) is the distance to the center of the training data. The larger the MD, the less trustworthy the prediction.
Mahalanobis Distance p-value: The p-value gives the fraction of training data with an MD greater than or equal to the one for the given sample, assuming normally distributed data. The smaller the p-value, the less trustworthy the prediciton. For highly non-normal X properties (e.g., fingerprints), the MD p-value is wildly inaccurate.

Structural Similar Compounds

Name	1,5-Naphthalenedisulfonic acid, 2-(6-(4,6-dichloro-s-triazinyl)meth ylamino-1-hydroxy-3-sulfonaphthylazo)-	2,7-Naphthalenedisulfonic acid, 4-(4,6-dichloro-s-triazin-2-ylamino)-5-hydroxy-6-(2-hydroxy- 5-nitrophenylazo)-	Anthraquinone, 3-methoxy-5,4'-iminobis(1-benzamido-
Structure	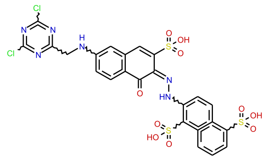	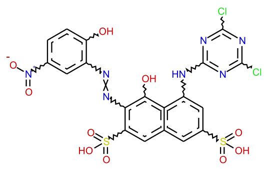	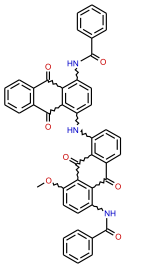
Actual Endpoint	Irritant	Irritant	Irritant
Predicted Endpoint	Non-Irritant	Non-Irritant	Non-Irritant
Distance	1.236	1.430	1.492
Reference	28ZPAK "Sbornik Vysledku Toxixologickeho Vysetreni Latek A Pripravku," Marhol d, J.V., Institut Pro Vychovu Vedoucicn Pracovniku Chemickeho Prumyclu Praha, Cz echoslovakia, 1972 Volume(issue)/page/year: -,237,1	28ZPAK "Sbornik Vysledku Toxixologickeho Vysetreni Latek A Pripravku," Marhol d, J.V., Institut Pro Vychovu Vedoucicn Pracovniku Chemickeho Prumyclu Praha, Cz echoslovakia, 1972 Volume(issue)/page/year: -,234,1	28ZPAK "Sbornik Vysledku Toxixologickeho Vysetreni Latek A Pripravku," Marhol d, J.V., Institut Pro Vychovu Vedoucicn Pracovniku Chemickeho Prumyclu Praha, Cz echoslovakia, 1972 Volume(issue)/page/year: -,114,1

Model Applicability

Unknown features are fingerprint features in the query molecule, but not found in the training set.

1. OPS PC11 out of range. Value: -5.9965. Training min, max, SD, explained variance: -5.6687, 5.5548, 1.412, 0.0252.
2. OPS PC22 out of range. Value: 6.3722. Training min, max, SD, explained variance: -2.9623, 3.7863, 1.016, 0.0131.
3. Unknown FCFP_2 feature: 18028531: [*]C([*])[NH2+]C
4. Unknown FCFP_2 feature: -1817836174: [*]CC([NH2+][*])C(=[*])[*]

Feature Contribution

Top features for positive contribution

Fingerprint	Bit/Smiles	Feature Structure	Score	Irritant in training set
FCFP_12	-1870530637	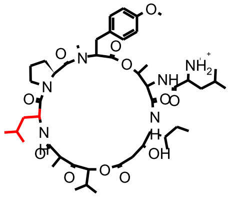 [*]CCC(C)C	0.087	44 out of 44
FCFP_12	-1580903393	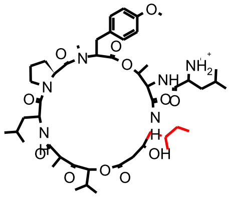 [*]C([*])C(C)CC	0.084	20 out of 20
FCFP_12	1985089045	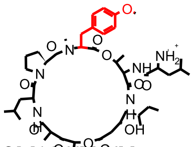 [*]O[c]1:[cH]:[cH]:[c](CC([*])[*]):[cH]:[cH]:1	0.078	8 out of 8

Top Features for negative contribution

Fingerprint	Bit/Smiles	Feature Structure	Score	Irritant in training set
FCFP_12	566058135	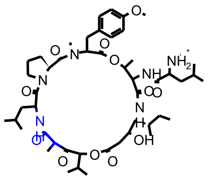 [*]NC(=O)C([*])[*]	-0.367	13 out of 21

FCFP_12	346218766	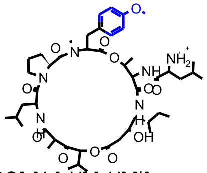 CO[c]1:[cH]:[cH]:[*]:[cH]:[cH]:1	-0.091	35 out of 42
FCFP_12	1674451008	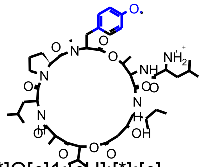 [*]O[c]1:[cH]:[*]:[c]([*]):[cH]:[cH]:1	-0.087	93 out of 111

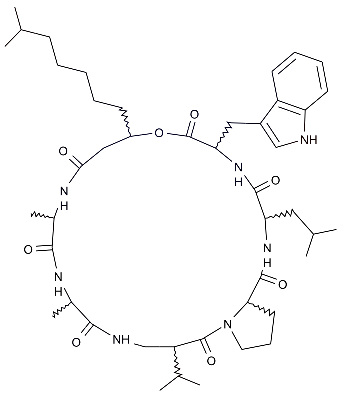

$C_{45}H_{69}N_7O_8$

Molecular Weight: 836.07145

ALogP: 4.986

Rotatable Bonds: 11

Acceptors: 8

Donors: 6

Model Prediction

Prediction: Non-Irritant

Probability: 0.967

Enrichment: 1.051

Bayesian Score: -1.176

Mahalanobis Distance: 21.475

Mahalanobis Distance p-value: 8.03e-045

Prediction: Positive if the Bayesian score is above the estimated best cutoff value from minimizing the false positive and false negative rate.
Probability: The esimated probability that the sample is in the positive category. This assumes that the Bayesian score follows a normal distribution and is different from the prediction using a cutoff.
Enrichment: An estimate of enrichment, that is, the increased likelihood (versus random) of this sample being in the category.
Bayesian Score: The standard Laplacian-modified Bayesian score.
Mahalanobis Distance: The Mahalanobis distance (MD) is the distance to the center of the training data. The larger the MD, the less trustworthy the prediction.
Mahalanobis Distance p-value: The p-value gives the fraction of training data with an MD greater than or equal to the one for the given sample, assuming normally distributed data. The smaller the p-value, the less trustworthy the prediciton. For highly non-normal X properties (e.g., fingerprints), the MD p-value is wildly inaccurate.

Structural Similar Compounds

Name	Anthraquinone, 3-methoxy-5,4'-iminobis(1-benzamido-	1,5-Naphthalenedisulfonic acid, 2-(6-(4,6-dichloro-s-triazinyl)meth ylamino-1-hydroxy-3-sulfonaphthylazo)-	Butanamide, 2,2'-((3,3'-dichloro(1,1'-biphenyl)-4,4'-diyl)bis(azo)) bis(N-(2,4- dimethylphenyl)-3-oxo-
Structure	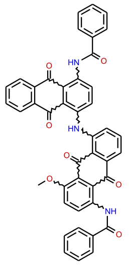	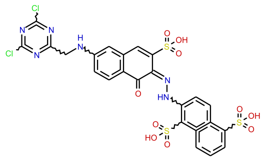	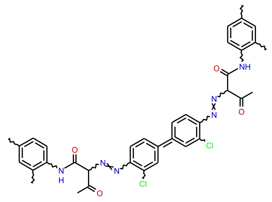
Actual Endpoint	Irritant	Irritant	Irritant
Predicted Endpoint	Non-Irritant	Non-Irritant	Non-Irritant
Distance	1.272	1.487	1.502
Reference	28ZPAK "Sbornik Vysledku Toxixologickeho Vysetreni Latek A Pripravku," Marhol d, J.V., Institut Pro Vychovu Vedoucicn Pracovniku Chemickeho Prumyclu Praha, Cz echoslovakia, 1972 Volume(issue)/page/year: -,114,1	28ZPAK "Sbornik Vysledku Toxixologickeho Vysetreni Latek A Pripravku," Marhol d, J.V., Institut Pro Vychovu Vedoucicn Pracovniku Chemickeho Prumyclu Praha, Cz echoslovakia, 1972 Volume(issue)/page/year: -,237,1	NTIS** National Technical Information Service. (Springfield, VA 22161) Forme rly U.S. Clearinghouse for Scientific & Technical Information. Volume(issue)/pag e/year: OTS0555058

Model Applicability

Unknown features are fingerprint features in the query molecule, but not found in the training set.

1. OPS PC6 out of range. Value: 7.0054. Training min, max, SD, explained variance: -5.7234, 6.8628, 1.786, 0.0403.
2. OPS PC7 out of range. Value: 6.5715. Training min, max, SD, explained variance: -5.5319, 5.3664, 1.751, 0.0388.
3. OPS PC11 out of range. Value: -6.0201. Training min, max, SD, explained variance: -5.6687, 5.5548, 1.412, 0.0252.
4. OPS PC22 out of range. Value: 4.6233. Training min, max, SD, explained variance: -2.9623, 3.7863, 1.016, 0.0131.
5. Unknown FCFP_2 feature: 1618184456: [*][c]1:[*]:[*]:[nH]:c:1

Feature Contribution

Top features for positive contribution				
**Fingerprint**	**Bit/Smiles**	**Feature Structure**	**Score**	**Irritant in training set**
FCFP_12	-1870530637	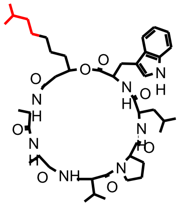 [*]CCC(C)C	0.087	44 out of 44
FCFP_12	-1873833927	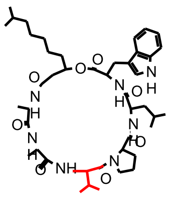 [*]CC(C(C)C)C(=[*])[*]<br>]	0.080	10 out of 10
FCFP_12	-154166589	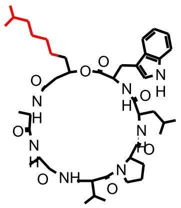 [*]CCCCC(C)C	0.078	8 out of 8

Top Features for negative contribution				
**Fingerprint**	**Bit/Smiles**	**Feature Structure**	**Score**	**Irritant in training set**
FCFP_12	392412010	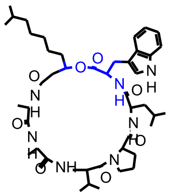 [*]CC(N[*])C(=O)OC([*<br>])[*]	-0.440	2 out of 4

FCFP_12	566058135	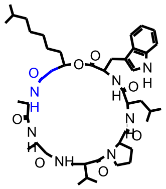 [*]NC(=O)C([*])[*]	-0.367	13 out of 21
FCFP_12	-547731249	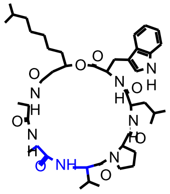 [*]C([*])CNC(=O)C([*])[*]	-0.222	2 out of 3

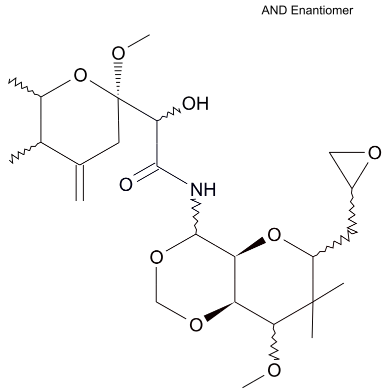

$C_{24}H_{39}NO_9$
Molecular Weight: 485.56776
ALogP: 0.267
Rotatable Bonds: 7
Acceptors: 9
Donors: 2

Model Prediction

Prediction: Irritant
Probability: 0.996
Enrichment: 1.081
Bayesian Score: 0.061
Mahalanobis Distance: 15.663
Mahalanobis Distance p-value: 1.06e-016

Prediction: Positive if the Bayesian score is above the estimated best cutoff value from minimizing the false positive and false negative rate.
Probability: The esimated probability that the sample is in the positive category. This assumes that the Bayesian score follows a normal distribution and is different from the prediction using a cutoff.
Enrichment: An estimate of enrichment, that is, the increased likelihood (versus random) of this sample being in the category.
Bayesian Score: The standard Laplacian-modified Bayesian score.
Mahalanobis Distance: The Mahalanobis distance (MD) is the distance to the center of the training data. The larger the MD, the less trustworthy the prediction.
Mahalanobis Distance p-value: The p-value gives the fraction of training data with an MD greater than or equal to the one for the given sample, assuming normally distributed data. The smaller the p-value, the less trustworthy the prediciton. For highly non-normal X properties (e.g., fingerprints), the MD p-value is wildly inaccurate.

Structural Similar Compounds

Name	Pregna-1,4-diene-3,20-dione, 21-(acetyloxy)-11-hydroxy-6-methyl-17- (1-oxopropoxy)-, (6-alpha,11-beta)-	2-Anthracenesulfonic acid, 1-amino-9,10-dihydro-9,10-dioxo-4-(2,4,6 -trimethylanilino)-, monosodium salt	Benzenesulfonic acid, 2,2'-(1,4-anthraquinonylenediimino)bis(5-meth yl-, disodium salt
Structure	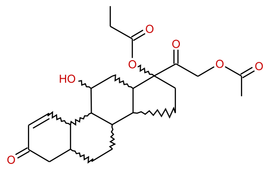	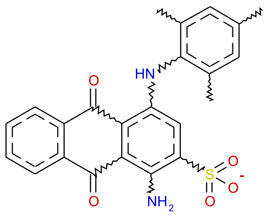	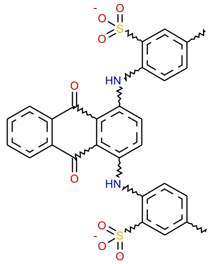
Actual Endpoint	Irritant	Irritant	Irritant
Predicted Endpoint	Irritant	Non-Irritant	Non-Irritant
Distance	0.818	0.863	0.967
Reference	YACHDS Yakuri to Chiryo. Pharmacology and Therapeutics. (Raifu Saiensu Shup pan K.K., 2-5-13, Yaesu, Chuo-ku, Tokyo 104, Japan) V.1-1972- Volume(issue) /page/year: 19,3103,1991	85JCAE "Prehled Prumyslove Toxikologie; Organicke Latky," Marhold, J., Prague , Czechoslovakia, Avicenum, 1986 Volume(issue)/page/year: -,1327,1986	85JCAE "Prehled Prumyslove Toxikologie; Organicke Latky," Marhold, J., Prague , Czechoslovakia, Avicenum, 1986 Volume(issue)/page/year: -,1330,1986

Model Applicability

Unknown features are fingerprint features in the query molecule, but not found in the training set.

1. OPS PC22 out of range. Value: 4.795. Training min, max, SD, explained variance: -2.9623, 3.7863, 1.016, 0.0131.

Feature Contribution

Top features for positive contribution				
Fingerprint	Bit/Smiles	Feature Structure	Score	Irritant in training set

FCFP_12	654335567	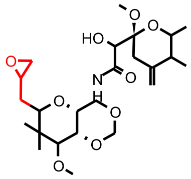 [*]OC(COC(=[*])[*])C([*])[*]	0.086	29 out of 29
FCFP_12	470041467	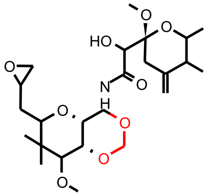 [*]OCO[*]	0.081	11 out of 11
FCFP_12	436886043	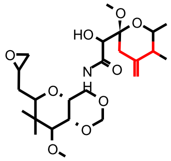 [*]\C=C(/C([*])[*])\C ([*])([*])[*]	0.080	129 out of 130

Top Features for negative contribution

Fingerprint	Bit/Smiles	Feature Structure	Score	Irritant in training set
FCFP_12	566058135	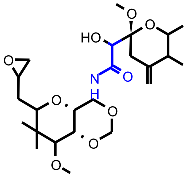 [*]NC(=O)C([*])[*]	-0.367	13 out of 21
FCFP_12	3	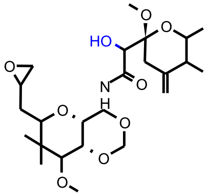 [*]O	-0.081	291 out of 345

FCFP_12	1070061035	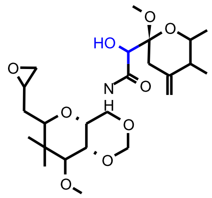 [*]C([*])O	0.000	215 out of 237

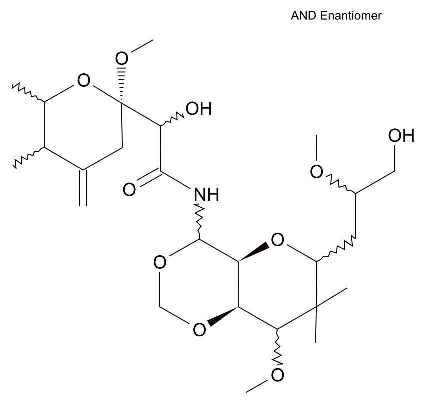

$C_{25}H_{43}NO_{10}$
Molecular Weight: 517.60962
ALogP: -9.1e-002
Rotatable Bonds: 9
Acceptors: 10
Donors: 3

Model Prediction

Prediction: Irritant
Probability: 0.996
Enrichment: 1.082
Bayesian Score: 0.194
Mahalanobis Distance: 15.040
Mahalanobis Distance p-value: 2.38e-014

Prediction: Positive if the Bayesian score is above the estimated best cutoff value from minimizing the false positive and false negative rate.
Probability: The esimated probability that the sample is in the positive category. This assumes that the Bayesian score follows a normal distribution and is different from the prediction using a cutoff.
Enrichment: An estimate of enrichment, that is, the increased likelihood (versus random) of this sample being in the category.
Bayesian Score: The standard Laplacian-modified Bayesian score.
Mahalanobis Distance: The Mahalanobis distance (MD) is the distance to the center of the training data. The larger the MD, the less trustworthy the prediction.
Mahalanobis Distance p-value: The p-value gives the fraction of training data with an MD greater than or equal to the one for the given sample, assuming normally distributed data. The smaller the p-value, the less trustworthy the prediciton. For highly non-normal X properties (e.g., fingerprints), the MD p-value is wildly inaccurate.

Structural Similar Compounds

Name	Benzenesulfonic acid, 2,2'-(1,4-anthraquinonylenediimino)bis(5-meth yl-, disodium salt	2-Anthracenesulfonic acid, 1-amino-9,10-dihydro-9,10-dioxo-4-(2,4,6 -trimethylanilino)-, monosodium salt	Fumaric acid, bis(3-allyloxy-2-hydroxypropyl) ester
Structure	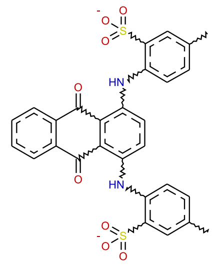	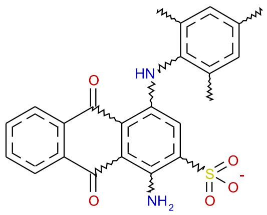	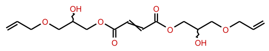
Actual Endpoint	Irritant	Irritant	Irritant
Predicted Endpoint	Non-Irritant	Non-Irritant	Irritant
Distance	0.932	1.022	1.042
Reference	85JCAE "Prehled Prumyslove Toxikologie; Organicke Latky," Marhold, J., Prague , Czechoslovakia, Avicenum, 1986 Volume(issue)/page/year: -,1330,1986	85JCAE "Prehled Prumyslove Toxikologie; Organicke Latky," Marhold, J., Prague , Czechoslovakia, Avicenum, 1986 Volume(issue)/page/year: -,1327,1986	85JCAE "Prehled Prumyslove Toxikologie; Organicke Latky," Marhold, J., Prague , Czechoslovakia, Avicenum, 1986 Volume(issue)/page/year: -,646,1986

Model Applicability

Unknown features are fingerprint features in the query molecule, but not found in the training set.

1. OPS PC22 out of range. Value: 4.7631. Training min, max, SD, explained variance: -2.9623, 3.7863, 1.016, 0.0131.

Feature Contribution

Top features for positive contribution				
Fingerprint	Bit/Smiles	Feature Structure	Score	Irritant in training set

FCFP_12	470041467	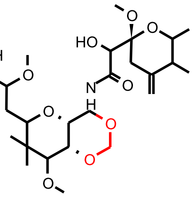 [*]OCO[*]	0.081	11 out of 11
FCFP_12	436886043	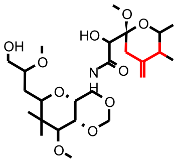 [*]\C=C(/C([*])[*])\C([*])([*])[*]	0.080	129 out of 130
FCFP_12	1327594201	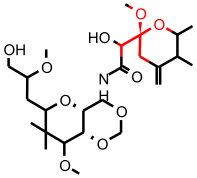 [*]CC(O[*])(O[*])C([*])[*]	0.077	7 out of 7
**Top Features for negative contribution**				
**Fingerprint**	**Bit/Smiles**	**Feature Structure**	**Score**	**Irritant in training set**
FCFP_12	566058135	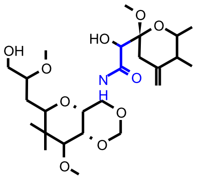 [*]NC(=O)C([*])[*]	-0.367	13 out of 21
FCFP_12	3	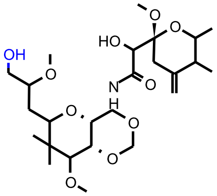 [*]O	-0.081	291 out of 345

FCFP_12	136627117	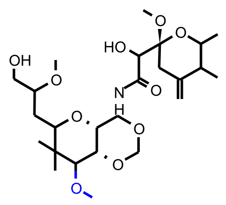 [*]OC	0.000	106 out of 116

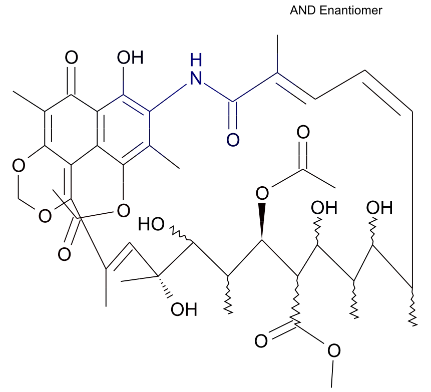

$C_{42}H_{53}NO_{15}$
Molecular Weight: 811.86791
ALogP: 2.745
Rotatable Bonds: 6
Acceptors: 15
Donors: 6

Model Prediction

Prediction: Non-Irritant
Probability: 0.938
Enrichment: 1.019
Bayesian Score: -2.176
Mahalanobis Distance: 17.811
Mahalanobis Distance p-value: 6.54e-026

Prediction: Positive if the Bayesian score is above the estimated best cutoff value from minimizing the false positive and false negative rate.
Probability: The esimated probability that the sample is in the positive category. This assumes that the Bayesian score follows a normal distribution and is different from the prediction using a cutoff.
Enrichment: An estimate of enrichment, that is, the increased likelihood (versus random) of this sample being in the category.
Bayesian Score: The standard Laplacian-modified Bayesian score.
Mahalanobis Distance: The Mahalanobis distance (MD) is the distance to the center of the training data. The larger the MD, the less trustworthy the prediction.
Mahalanobis Distance p-value: The p-value gives the fraction of training data with an MD greater than or equal to the one for the given sample, assuming normally distributed data. The smaller the p-value, the less trustworthy the prediciton. For highly non-normal X properties (e.g., fingerprints), the MD p-value is wildly inaccurate.

Structural Similar Compounds

Name	1,5-Naphthalenedisulfonic acid, 2-(6-(4,6-dichloro-s-triazinyl)meth ylamino-1-hydroxy-3-sulfonaphthylazo)-	2,7-Naphthalenedisulfonic acid, 4-(4,6-dichloro-s-triazin-2-ylamino)-5-hydroxy-6-(2-hydroxy- 5-nitrophenylazo)-	Amipurimycin, hydrate
Structure	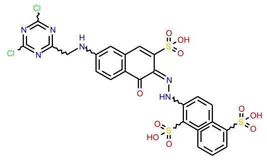	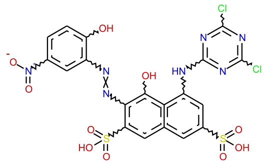	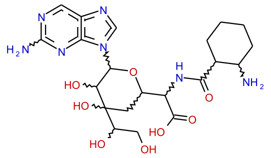
Actual Endpoint	Irritant	Irritant	Irritant
Predicted Endpoint	Non-Irritant	Non-Irritant	Non-Irritant
Distance	0.924	1.111	1.595
Reference	28ZPAK "Sbornik Vysledku Toxixologickeho Vysetreni Latek A Pripravku," Marhol d, J.V., Institut Pro Vychovu Vedoucicn Pracovniku Chemickeho Prumyclu Praha, Cz echoslovakia, 1972 Volume(issue)/page/year: -,237,1	28ZPAK "Sbornik Vysledku Toxixologickeho Vysetreni Latek A Pripravku," Marhol d, J.V., Institut Pro Vychovu Vedoucicn Pracovniku Chemickeho Prumyclu Praha, Cz echoslovakia, 1972 Volume(issue)/page/year: -,234,1	JANTAJ Journal of Antibiotics. (Japan Antibiotics Research Assoc., 2-20-8 Ka miosaki, Shinagawa-ku, Tokyo, 141, Japan) V.2-5, 1948-52; V.21- 1968- Volume(i ssue)/page/year: 30,1,1977

Model Applicability

Unknown features are fingerprint features in the query molecule, but not found in the training set.

1. OPS PC23 out of range. Value: -3.1489. Training min, max, SD, explained variance: -3.0369, 4.9599, 1.007, 0.0128.

Feature Contribution

Top features for positive contribution				
Fingerprint	Bit/Smiles	Feature Structure	Score	Irritant in training set

FCFP_12	-415156552	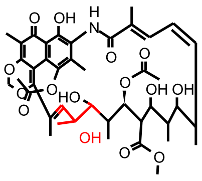 [*]C[C@@]1(O)C[*][*]C 1[*]	0.085	27 out of 27
FCFP_12	-1979033238	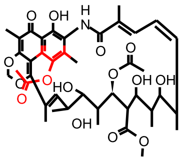 [*][c](:[*]):[c](OC(= O)C):[c]([*]):[*]	0.084	19 out of 19
FCFP_12	470041467	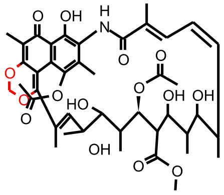 [*]OCO[*]	0.081	11 out of 11

Top Features for negative contribution

**Fingerprint**	**Bit/Smiles**	**Feature Structure**	**Score**	**Irritant in training set**
FCFP_12	1175665944	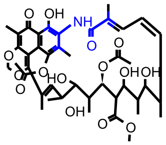 [*]C(=[*])C(=O)N[c](: [c]([*]):[*]):[c]([*]):[*]	-1.020	2 out of 8
FCFP_12	1294255210	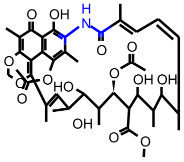 [*]C(=[*])N[c](:[*]): [*]	-0.486	12 out of 22

FCFP_12	590925877	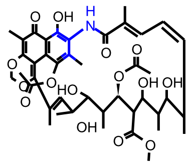<br>[*]N[c](:[c]([*]):[*]<br>):[c]([*]):[*]	-0.434	56 out of 95

Simeprevir

TOPKAT_Skin_Irritancy_None_vs_Irritant

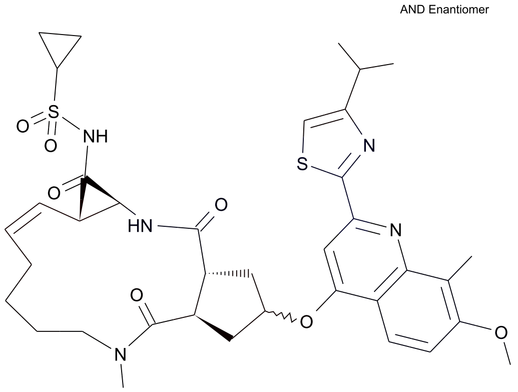

$C_{38}H_{47}N_5O_7S_2$
Molecular Weight: 749.93907
ALogP: 4.796
Rotatable Bonds: 8
Acceptors: 9
Donors: 2

Model Prediction

Prediction: Non-Irritant
Probability: 0.969
Enrichment: 1.053
Bayesian Score: -1.048
Mahalanobis Distance: 16.814
Mahalanobis Distance p-value: 1.94e-021

Prediction: Positive if the Bayesian score is above the estimated best cutoff value from minimizing the false positive and false negative rate.
Probability: The esimated probability that the sample is in the positive category. This assumes that the Bayesian score follows a normal distribution and is different from the prediction using a cutoff.
Enrichment: An estimate of enrichment, that is, the increased likelihood (versus random) of this sample being in the category.
Bayesian Score: The standard Laplacian-modified Bayesian score.
Mahalanobis Distance: The Mahalanobis distance (MD) is the distance to the center of the training data. The larger the MD, the less trustworthy the prediction.
Mahalanobis Distance p-value: The p-value gives the fraction of training data with an MD greater than or equal to the one for the given sample, assuming normally distributed data. The smaller the p-value, the less trustworthy the prediciton. For highly non-normal X properties (e.g., fingerprints), the MD p-value is wildly inaccurate.

Structural Similar Compounds

Name	Anthraquinone, 3-methoxy-5,4'-iminobis(1-benzamido-	Benzenesulfonic acid, 2,2'-(1,4-anthraquinonylenediimino)bis(5-meth yl-, disodium salt	Butanamide, 2,2'-((3,3'-dichloro(1,1'-biphenyl)-4,4'-diyl)bis(azo)) bis(N-(2,4- dimethylphenyl)-3-oxo-
Structure	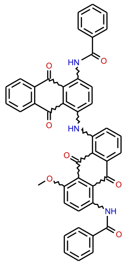	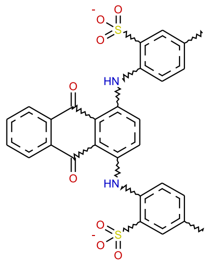	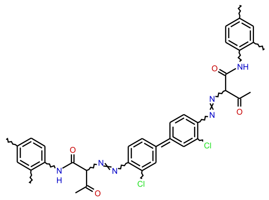
Actual Endpoint	Irritant	Irritant	Irritant
Predicted Endpoint	Non-Irritant	Non-Irritant	Non-Irritant
Distance	0.883	1.017	1.027
Reference	28ZPAK "Sbornik Vysledku Toxixologickeho Vysetreni Latek A Pripravku," Marhol d, J.V., Institut Pro Vychovu Vedoucicn Pracovniku Chemickeho Prumyclu Praha, Cz echoslovakia, 1972 Volume(issue)/page/year: -,114,1	85JCAE "Prehled Prumyslove Toxikologie; Organicke Latky," Marhold, J., Prague , Czechoslovakia, Avicenum, 1986 Volume(issue)/page/year: -,1330,1986	NTIS** National Technical Information Service. (Springfield, VA 22161) Forme rly U.S. Clearinghouse for Scientific & Technical Information. Volume(issue)/pag e/year: OTS0555058

Model Applicability

Unknown features are fingerprint features in the query molecule, but not found in the training set.

1. OPS PC6 out of range. Value: 7.6954. Training min, max, SD, explained variance: -5.7234, 6.8628, 1.786, 0.0403.
2. OPS PC22 out of range. Value: 5.1294. Training min, max, SD, explained variance: -2.9623, 3.7863, 1.016, 0.0131.

Feature Contribution

Top features for positive contribution				
Fingerprint	Bit/Smiles	Feature Structure	Score	Irritant in training set

FCFP_12	-415156552	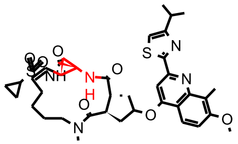 [*]C[C@@]1(O)C[*][*]C 1[*]	0.085	27 out of 27
FCFP_12	-53728878	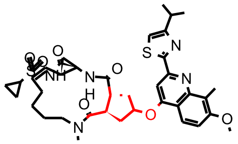 [*]OC1C[C@@H]([*])[C@ @H](C1)C(=[*])[*]	0.085	21 out of 21
FCFP_12	1186303932	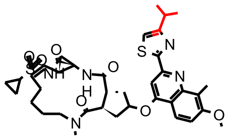 [*]:[c](:[*])C(C)C	0.084	18 out of 18

Top Features for negative contribution

Fingerprint	Bit/Smiles	Feature Structure	Score	Irritant in training set
FCFP_12	690481386	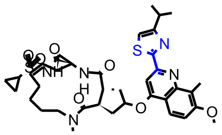 [*]:[c](:[*])[c]1:n:[*]:[*]:s:1	-0.650	0 out of 1
FCFP_12	566058135	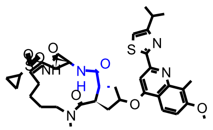 [*]NC(=O)C([*])[*]	-0.367	13 out of 21

FCFP_12	-1320007763	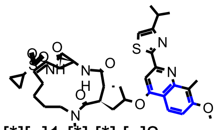 [*][c]1:[*]:[*]:[c]2:[*]:[cH]:[cH]:[cH]:[c]:1:2	-0.089	20 out of 24

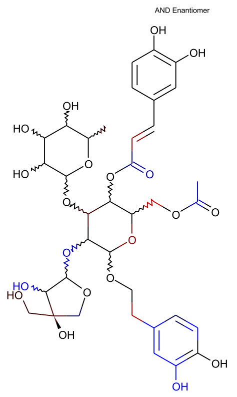

$C_{36}H_{46}O_{20}$

Molecular Weight: 798.73843

ALogP: -0.546

Rotatable Bonds: 16

Acceptors: 20

Donors: 10

Model Prediction

Prediction: 2.871

Unit: mg/kg_body_weight/day

Mahalanobis Distance: 19.229

Mahalanobis Distance p-value: 1.71e-027

Mahalanobis Distance: The Mahalanobis distance (MD) is a generalization of the Euclidean distance that accounts for correlations among the X properties. It is calculated as the distance to the center of the training data. The larger the MD, the less trustworthy the prediction.
Mahalanobis Distance p-value: The p-value gives the fraction of training data with an MD greater than or equal to the one for the given sample, assuming normally distributed data. The smaller the p-value, the less trustworthy the prediciton. For highly non-normal X properties (e.g., fingerprints), the MD p-value is wildly inaccurate.

Structural Similar Compounds

Name	532	380	706
Structure	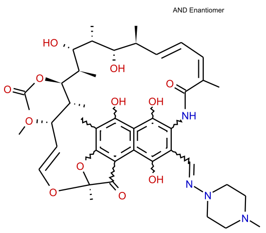	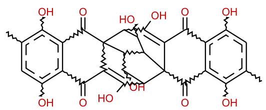	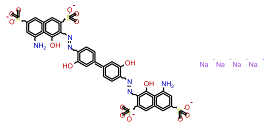
Actual Endpoint (-log C)	4.38903	4.48977	3.00743
Predicted Endpoint (-log C)	5.60554	6.22716	4.81633
Distance	1.398	1.553	1.641
Reference	CPDB	CPDB	CPDB

Model Applicability

Unknown features are fingerprint features in the query molecule, but not found in the training set.

1. Num_H_Donors out of range. Value: 10. Training min, max, mean, SD: 0, 8, 1.1962, 1.306.
2. OPS PC2 out of range. Value: 9.0039. Training min, max, SD, explained variance: -5.652, 8.9166, 2.513, 0.0788.
3. Unknown ECFP_2 feature: 456242574: [*]C([*])OC([*])[*]
4. Unknown ECFP_2 feature: -1250019913: [*]COC([*])[*]
5. Unknown ECFP_2 feature: -2060414325: [*]OC(O[*])C([*])[*]
6. Unknown ECFP_2 feature: 1953003528: [*]C[C@@]1(O)C[*][*]C1[*]
7. Unknown ECFP_2 feature: 1535429263: [*]OC(C)C([*])[*]

Feature Contribution

Top features for positive contribution			
**Fingerprint**	**Bit/Smiles**	**Feature Structure**	**Score**

ECFP_6	1559650422	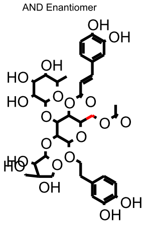 [*]C[*]	0.203
ECFP_6	-2024255407	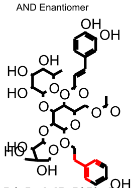 [*]C[c](:[cH]:[*]):[cH]:[*]	0.172
ECFP_6	-1925046727	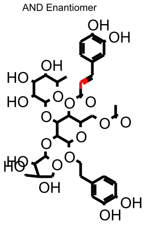 [*]C=[*]	0.145

Top Features for negative contribution

Fingerprint	Bit/Smiles	Feature Structure	Score
ECFP_6	2019062761	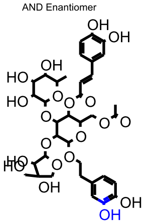 [*]:[c](:[*])O	-0.258
ECFP_6	1996767644	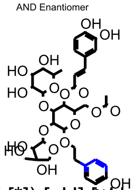 [*][c](:[*]):[cH]:[cH]:[*]	-0.251

ECFP_6	642810091	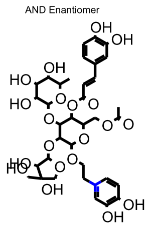 [*][c](:[*]):[*]	-0.247

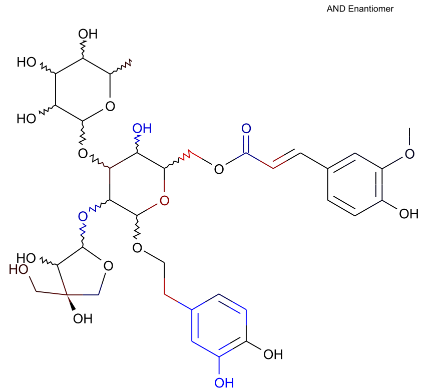

$C_{35}H_{46}O_{19}$
Molecular Weight: 770.72833
ALogP: -0.699
Rotatable Bonds: 15
Acceptors: 19
Donors: 10

Structural Similar Compounds

Name	532	380	Fumonisin B1
Structure	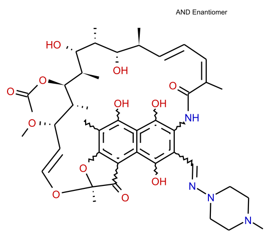	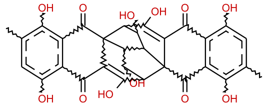	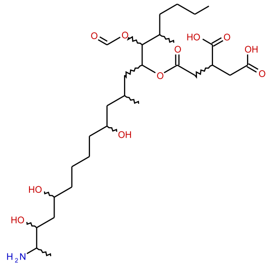
Actual Endpoint (-log C)	4.38903	4.48977	4.94025
Predicted Endpoint (-log C)	5.60554	6.22716	3.434
Distance	1.324	1.463	1.613
Reference	CPDB	CPDB	CPDB

Model Prediction

Prediction: 1.854
Unit: mg/kg_body_weight/day
Mahalanobis Distance: 18.931
Mahalanobis Distance p-value: 2.2e-026

Mahalanobis Distance: The Mahalanobis distance (MD) is a generalization of the Euclidean distance that accounts for correlations among the X properties. It is calculated as the distance to the center of the training data. The larger the MD, the less trustworthy the prediction.
Mahalanobis Distance p-value: The p-value gives the fraction of training data with an MD greater than or equal to the one for the given sample, assuming normally distributed data. The smaller the p-value, the less trustworthy the prediciton. For highly non-normal X properties (e.g., fingerprints), the MD p-value is wildly inaccurate.

Model Applicability

Unknown features are fingerprint features in the query molecule, but not found in the training set.

1. Num_H_Donors out of range. Value: 10. Training min, max, mean, SD: 0, 8, 1.1962, 1.306.
2. Unknown ECFP_2 feature: 456242574: [*]C([*])OC([*])[*]
3. Unknown ECFP_2 feature: -1250019913: [*]COC([*])[*]
4. Unknown ECFP_2 feature: -2060414325: [*]OC(O[*])C([*])[*]
5. Unknown ECFP_2 feature: 1953003528: [*]C[C@@]1(O)C[*][*]C1[*]
6. Unknown ECFP_2 feature: 1535429263: [*]OC(C)C([*])[*]

Feature Contribution

Top features for positive contribution

Fingerprint	Bit/Smiles	Feature Structure	Score
ECFP_6	1559650422	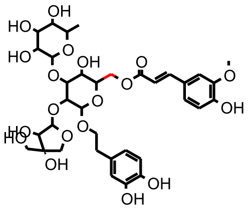 [*]C[*]	0.203

ECFP_6	-2024255407	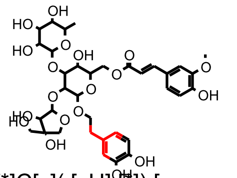 [*]C[c](:[cH]:[*]):[cH]:[*]	0.172
ECFP_6	-1925046727	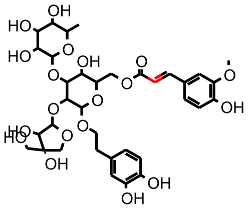 [*]C=[*]	0.145

Top Features for negative contribution

Fingerprint	Bit/Smiles	Feature Structure	Score
ECFP_6	2019062761	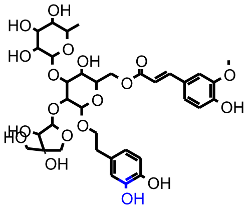 [*]:[c](:[*])O	-0.258
ECFP_6	1996767644	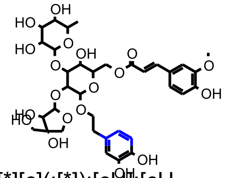 [*][c](:[*]):[cH]:[cH]:[*]	-0.251
ECFP_6	642810091	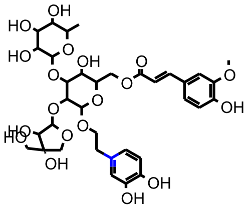 [*][c](:[*]):[*]	-0.247

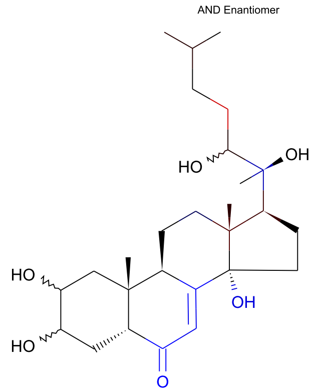

$C_{27}H_{44}O_6$
Molecular Weight: 464.63466
ALogP: 2.489
Rotatable Bonds: 5
Acceptors: 6
Donors: 5

Model Prediction

Prediction: 5.663
Unit: mg/kg_body_weight/day
Mahalanobis Distance: 9.188
Mahalanobis Distance p-value: 0.213

Mahalanobis Distance: The Mahalanobis distance (MD) is a generalization of the Euclidean distance that accounts for correlations among the X properties. It is calculated as the distance to the center of the training data. The larger the MD, the less trustworthy the prediction.
Mahalanobis Distance p-value: The p-value gives the fraction of training data with an MD greater than or equal to the one for the given sample, assuming normally distributed data. The smaller the p-value, the less trustworthy the prediciton. For highly non-normal X properties (e.g., fingerprints), the MD p-value is wildly inaccurate.

Structural Similar Compounds

Name	a-Ecdysone	894	Phorbol
Structure	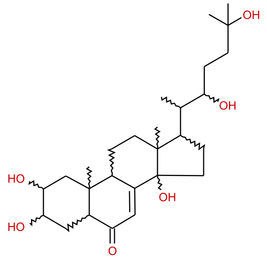	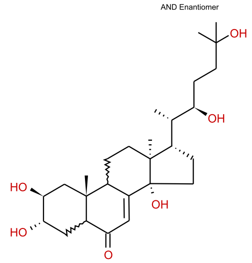	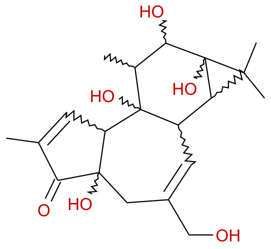
Actual Endpoint (-log C)	7.11323	7.11323	5.21723
Predicted Endpoint (-log C)	4.88091	4.88091	4.6562
Distance	0.244	0.244	0.755
Reference	CPDB	CPDB	CPDB

Model Applicability

Unknown features are fingerprint features in the query molecule, but not found in the training set.

1. All properties and OPS components are within expected ranges.
2. Unknown ECFP_2 feature: -1049278438: [*]C([*])([*])[C@H]1C[*][*]C1([*])[*]
3. Unknown ECFP_2 feature: 1195714366: [*]C([*])C(C)(O)C([*])[*]
4. Unknown ECFP_2 feature: 80071435: [*]CC(O)C([*])([*])[*]

Feature Contribution

Top features for positive contribution			
Fingerprint	Bit/Smiles	Feature Structure	Score
ECFP_6	1559650422	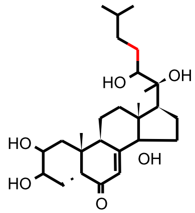 [*]C[*]	0.203

ECFP_6	657586427	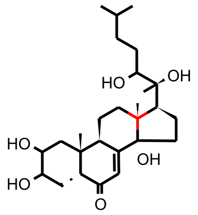 [*]C([*])([*])[*]	0.079
ECFP_6	-167460056	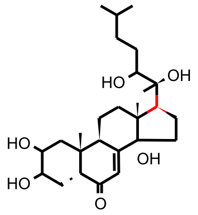 [*]C([*])[*]	0.060

Top Features for negative contribution

Fingerprint	Bit/Smiles	Feature Structure	Score
ECFP_6	2106656448	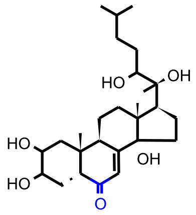 [*]C(=O)[*]	-0.275
ECFP_6	642810091	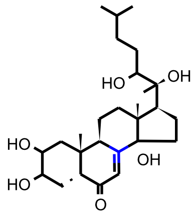 [*][c](:[*]):[*]	-0.247
ECFP_6	-182236392	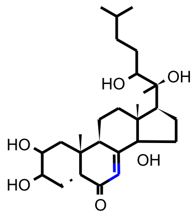 [*]:[cH]:[*]	-0.232

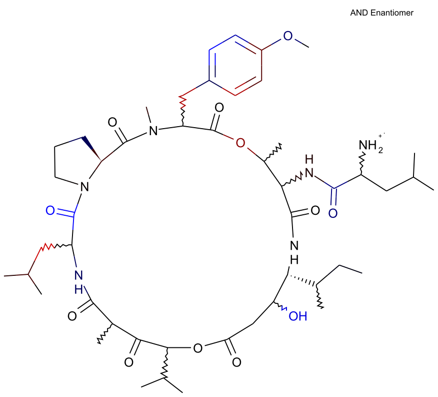

$C_{49}H_{79}N_6O_{12}$
Molecular Weight: 944.18455
ALogP: 3.176
Rotatable Bonds: 13
Acceptors: 12
Donors: 5

Model Prediction

Prediction: 8.687
Unit: mg/kg_body_weight/day
Mahalanobis Distance: 17.537
Mahalanobis Distance p-value: 2.3e-021

Mahalanobis Distance: The Mahalanobis distance (MD) is a generalization of the Euclidean distance that accounts for correlations among the X properties. It is calculated as the distance to the center of the training data. The larger the MD, the less trustworthy the prediction.
Mahalanobis Distance p-value: The p-value gives the fraction of training data with an MD greater than or equal to the one for the given sample, assuming normally distributed data. The smaller the p-value, the less trustworthy the prediciton. For highly non-normal X properties (e.g., fingerprints), the MD p-value is wildly inaccurate.

Structural Similar Compounds

Name	532	338	Leupeptin
Structure	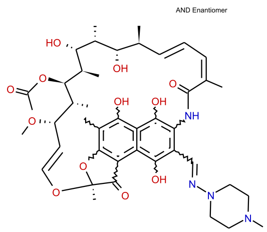	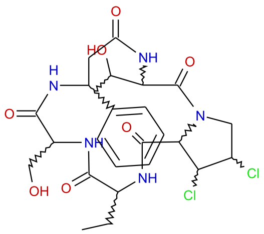	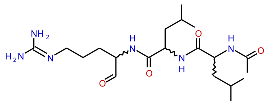
Actual Endpoint (-log C)	4.38903	4.39533	3.88334
Predicted Endpoint (-log C)	5.60554	4.31268	4.28559
Distance	1.011	1.229	1.323
Reference	CPDB	CPDB	CPDB

Model Applicability

Unknown features are fingerprint features in the query molecule, but not found in the training set.

1. Molecular_Weight out of range. Value: 944.18. Training min, max, mean, SD: 30.026, 871.78, 225.83, 133.9.
2. Unknown ECFP_2 feature: -591526139: [*][NH2+][*]
3. Unknown ECFP_2 feature: 1617733200: [*]C([*])N(C)C(=[*])[*]
4. Unknown ECFP_2 feature: 1352327988: [*]C([*])[NH2+]C
5. Unknown ECFP_2 feature: -84540346: [*]C([*])CC([*])[*]
6. Unknown ECFP_2 feature: -1224874527: [*]CC(N([*])[*])C(=[*])[*]
7. Unknown ECFP_2 feature: 1691770380: [*]NC(C([*])[*])C([*])[*]
8. Unknown ECFP_2 feature: 771121623: [*]C([*])C[c](:[*]):[*]
9. Unknown ECFP_2 feature: -1567199489: [*]NC(C([*])[*])C(=[*])[*]
10. Unknown ECFP_2 feature: -1886208901: [*]OC(=O)C([*])[*]
11. Unknown ECFP_2 feature: 1535429263: [*]OC(C)C([*])[*]
12. Unknown ECFP_2 feature: -714938792: [*]C(=[*])C(C)C(=[*])[*]
13. Unknown ECFP_2 feature: -855787384: [*]OC(C([*])[*])C(=[*])[*]
14. Unknown ECFP_2 feature: -212107780: [*]C([*])C(=O)C([*])[*]
15. Unknown ECFP_2 feature: -1167589895: [*]C([*])C(C)C
16. Unknown ECFP_2 feature: 20550775: [*]CC([NH2+][*])C(=[*])[*]
17. Unknown ECFP_2 feature: 866401773: [*][NH2+]C

Feature Contribution

Top features for positive contribution			
**Fingerprint**	**Bit/Smiles**	**Feature Structure**	**Score**
ECFP_6	1559650422	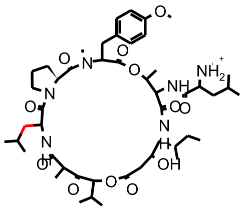 [*]C[*]	0.203
ECFP_6	-2024255407	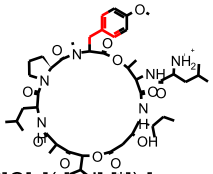 [*]C[c](:[cH]:[*]):[cH]:[*]	0.172
ECFP_6	683445015	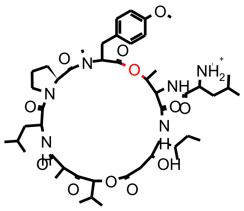 [*]O[*]	0.136

Top Features for negative contribution			
**Fingerprint**	**Bit/Smiles**	**Feature Structure**	**Score**
ECFP_6	2106656448	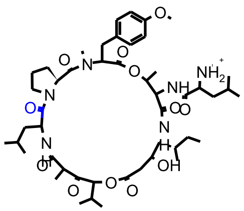 [*]C(=O)[*]	-0.275

ECFP_6	1996767644	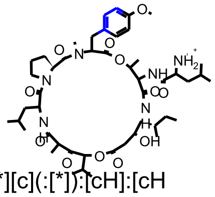 [*][c](:[*]):[cH]:[cH]:[*]	-0.251
ECFP_6	642810091	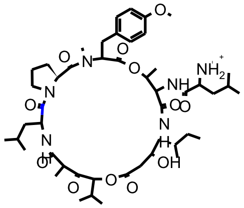 [*][c](:[*]):[*]	-0.247

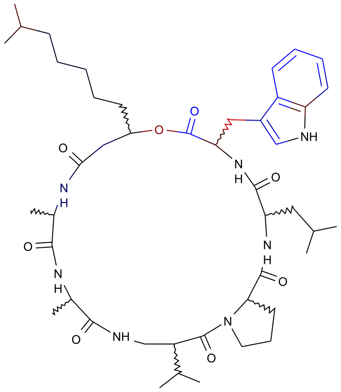

$C_{45}H_{69}N_7O_8$
Molecular Weight: 836.07145
ALogP: 4.986
Rotatable Bonds: 11
Acceptors: 8
Donors: 6

Model Prediction

Prediction: 3.037
Unit: mg/kg_body_weight/day
Mahalanobis Distance: 15.562
Mahalanobis Distance p-value: 8.51e-015

Mahalanobis Distance: The Mahalanobis distance (MD) is a generalization of the Euclidean distance that accounts for correlations among the X properties. It is calculated as the distance to the center of the training data. The larger the MD, the less trustworthy the prediction.
Mahalanobis Distance p-value: The p-value gives the fraction of training data with an MD greater than or equal to the one for the given sample, assuming normally distributed data. The smaller the p-value, the less trustworthy the prediciton. For highly non-normal X properties (e.g., fingerprints), the MD p-value is wildly inaccurate.

Structural Similar Compounds

Name	532	338	Cyclochlorotine
Structure	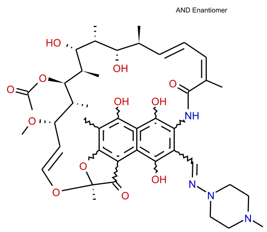	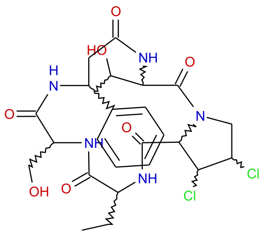	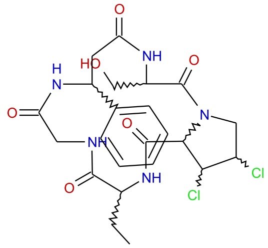
Actual Endpoint (-log C)	4.38903	4.39533	4.36142
Predicted Endpoint (-log C)	5.60554	4.31268	4.0834
Distance	1.049	1.074	1.170
Reference	CPDB	CPDB	CPDB

Model Applicability

Unknown features are fingerprint features in the query molecule, but not found in the training set.

1. All properties and OPS components are within expected ranges.
2. Unknown ECFP_2 feature: -1020449580: [*][c]1:[*]:[*]:[nH]:c:1
3. Unknown ECFP_2 feature: 771121623: [*]C([*])C[c](:[*]):[*]
4. Unknown ECFP_2 feature: -1886208901: [*]OC(=O)C([*])[*]
5. Unknown ECFP_2 feature: 1863041499: [*]NC(C)C(=[*])[*]
6. Unknown ECFP_2 feature: -1457159889: [*]NCC([*])[*]
7. Unknown ECFP_2 feature: -2096927833: [*]CC(C([*])[*])C(=[*])[*]
8. Unknown ECFP_2 feature: -1167589895: [*]C([*])C(C)C
9. Unknown ECFP_2 feature: -84540346: [*]C([*])CC([*])[*]

Feature Contribution

Top features for positive contribution

Fingerprint	Bit/Smiles	Feature Structure	Score

ECFP_6	1559650422	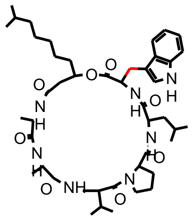 [*]C[*]	0.203
ECFP_6	683445015	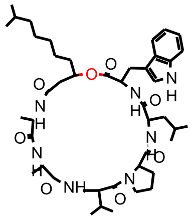 [*]O[*]	0.136
ECFP_6	1333660716	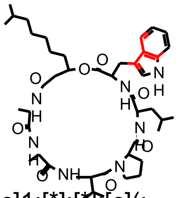 [*][c]1:[*]:[*]:[c](:[*]):[c]:1:[cH]:[*]	0.075

Top Features for negative contribution

Fingerprint	Bit/Smiles	Feature Structure	Score
ECFP_6	2106656448	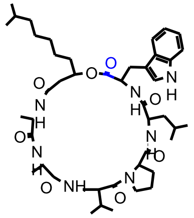 [*]C(=O)[*]	-0.275
ECFP_6	1996767644	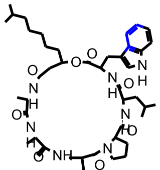 [*][c](:[*]):[cH]:[cH]:[*]	-0.251

ECFP_6	642810091	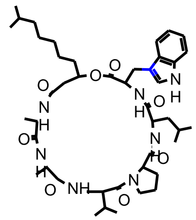 [*][c](:[*]):[*]	-0.247

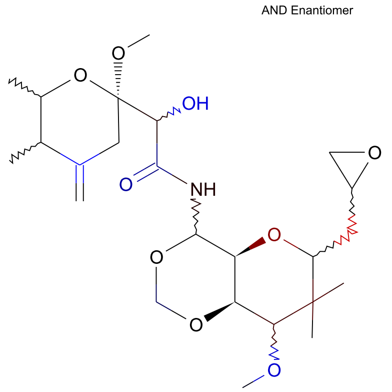

$C_{24}H_{39}NO_9$
Molecular Weight: 485.56776
ALogP: 0.267
Rotatable Bonds: 7
Acceptors: 9
Donors: 2

Structural Similar Compounds

Name	T-2 toxin	Riddelliine	Lovastatin
Structure	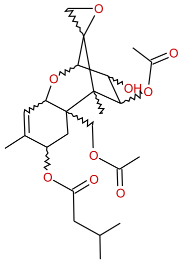	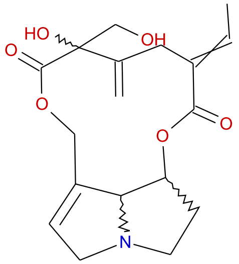	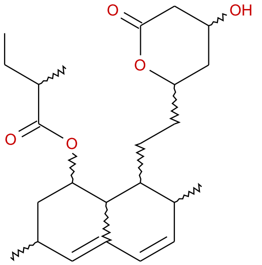
Actual Endpoint (-log C)	5.72291	5.24883	2.89515
Predicted Endpoint (-log C)	3.92553	4.57392	3.9059
Distance	0.620	0.842	0.906
Reference	CPDB	CPDB	CPDB

Model Prediction

Prediction: 7.360
Unit: mg/kg_body_weight/day
Mahalanobis Distance: 15.940
Mahalanobis Distance p-value: 5.38e-016

Mahalanobis Distance: The Mahalanobis distance (MD) is a generalization of the Euclidean distance that accounts for correlations among the X properties. It is calculated as the distance to the center of the training data. The larger the MD, the less trustworthy the prediction.
Mahalanobis Distance p-value: The p-value gives the fraction of training data with an MD greater than or equal to the one for the given sample, assuming normally distributed data. The smaller the p-value, the less trustworthy the prediciton. For highly non-normal X properties (e.g., fingerprints), the MD p-value is wildly inaccurate.

Model Applicability

Unknown features are fingerprint features in the query molecule, but not found in the training set.

1. All properties and OPS components are within expected ranges.
2. Unknown ECFP_2 feature: -194719409: [*]C([*])OC([*])([*])[*]
3. Unknown ECFP_2 feature: 1667686968: [*]C([*])([*])OC
4. Unknown ECFP_2 feature: 1067196438: [*]C([*])C(C)(C)C([*])[*]
5. Unknown ECFP_2 feature: -48623642: [*]CC(O[*])C([*])([*])[*]
6. Unknown ECFP_2 feature: -83576333: [*]C([*])CC1[*][*]1
7. Unknown ECFP_2 feature: -457637478: [*]NC(O[*])C([*])[*]
8. Unknown ECFP_2 feature: 837844420: [*]CC(O[*])(O[*])C([*])[*]
9. Unknown ECFP_2 feature: 825070174: [*]C(=[*])C(O)C([*])([*])[*]
10. Unknown ECFP_2 feature: 1863511924: [*]C([*])C(C)C(=[*])[*]
11. Unknown ECFP_2 feature: 1535429263: [*]OC(C)C([*])[*]
12. Unknown ECFP_2 feature: 1377749300: [*]CC(=C)C([*])[*]

Feature Contribution

Top features for positive contribution			
**Fingerprint**	**Bit/Smiles**	**Feature Structure**	**Score**

ECFP_6	1559650422	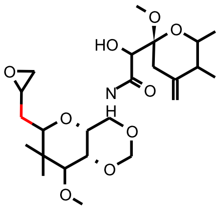 [*]C[*]	0.203
ECFP_6	683445015	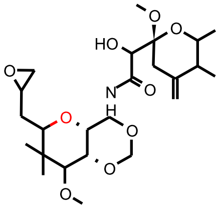 [*]O[*]	0.136
ECFP_6	657586427	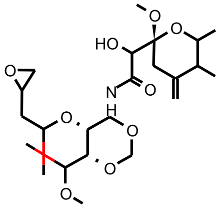 [*]C([*])([*])[*]	0.079
**Top Features for negative contribution**			
**Fingerprint**	**Bit/Smiles**	**Feature Structure**	**Score**
ECFP_6	642810091	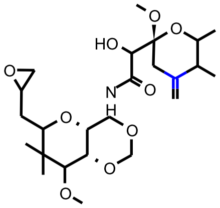 [*][c](:[*]):[*]	-0.247
ECFP_6	-1884411803	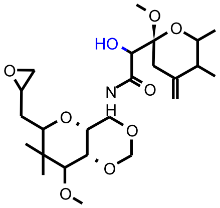 [*]O	-0.217

ECFP_6	-1059365320	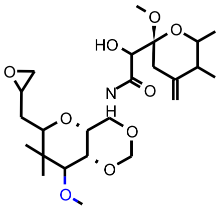<br>[*]O[*]	-0.184

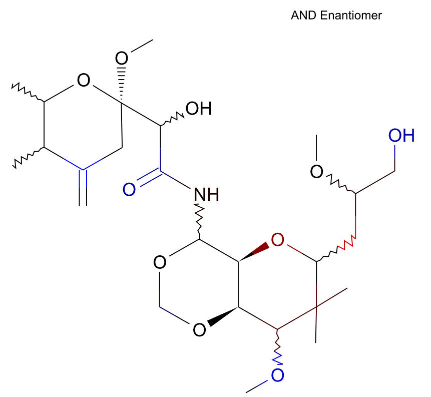

$C_{25}H_{43}NO_{10}$
Molecular Weight: 517.60962
ALogP: -9.1e-002
Rotatable Bonds: 9
Acceptors: 10
Donors: 3

Model Prediction

Prediction: 12.564
Unit: mg/kg_body_weight/day
Mahalanobis Distance: 15.470
Mahalanobis Distance p-value: 1.64e-014

Mahalanobis Distance: The Mahalanobis distance (MD) is a generalization of the Euclidean distance that accounts for correlations among the X properties. It is calculated as the distance to the center of the training data. The larger the MD, the less trustworthy the prediction.
Mahalanobis Distance p-value: The p-value gives the fraction of training data with an MD greater than or equal to the one for the given sample, assuming normally distributed data. The smaller the p-value, the less trustworthy the prediciton. For highly non-normal X properties (e.g., fingerprints), the MD p-value is wildly inaccurate.

Structural Similar Compounds

Name	T-2 toxin	Salicylazosulfapyridine	a-Ecdysone
Structure	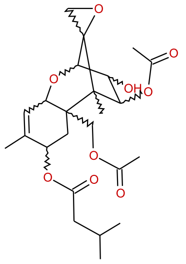	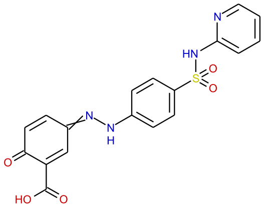	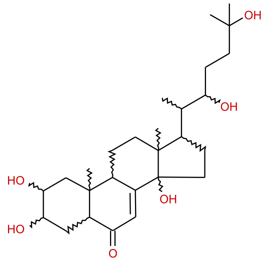
Actual Endpoint (-log C)	5.72291	2.5034	7.11323
Predicted Endpoint (-log C)	3.92553	3.54214	4.88091
Distance	0.743	0.920	0.935
Reference	CPDB	CPDB	CPDB

Model Applicability

Unknown features are fingerprint features in the query molecule, but not found in the training set.

1. All properties and OPS components are within expected ranges.
2. Unknown ECFP_2 feature: -410173153: [*]C([*])OC
3. Unknown ECFP_2 feature: -194719409: [*]C([*])OC([*])([*])[*]
4. Unknown ECFP_2 feature: 1667686968: [*]C([*])([*])OC
5. Unknown ECFP_2 feature: 1067196438: [*]C([*])C(C)(C)C([*])[*]
6. Unknown ECFP_2 feature: -48623642: [*]CC(O[*])C([*])([*])[*]
7. Unknown ECFP_2 feature: -84540346: [*]C([*])CC([*])[*]
8. Unknown ECFP_2 feature: -457637478: [*]NC(O[*])C([*])[*]
9. Unknown ECFP_2 feature: 837844420: [*]CC(O[*])(O[*])C([*])[*]
10. Unknown ECFP_2 feature: 825070174: [*]C(=[*])C(O)C([*])([*])[*]
11. Unknown ECFP_2 feature: 1863511924: [*]C([*])C(C)C(=[*])[*]
12. Unknown ECFP_2 feature: 1535429263: [*]OC(C)C([*])[*]
13. Unknown ECFP_2 feature: 1377749300: [*]CC(=C)C([*])[*]

Feature Contribution

Top features for positive contribution

Fingerprint	Bit/Smiles	Feature Structure	Score

ECFP_6	1559650422	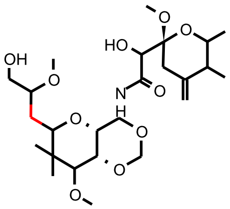 [*]C[*]	0.203
ECFP_6	683445015	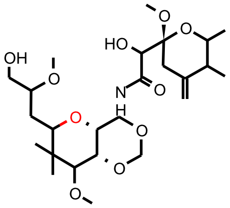 [*]O[*]	0.136
ECFP_6	657586427	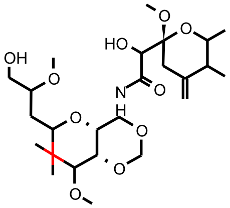 [*]C([*])([*])[*]	0.079

Top Features for negative contribution

Fingerprint	Bit/Smiles	Feature Structure	Score
ECFP_6	642810091	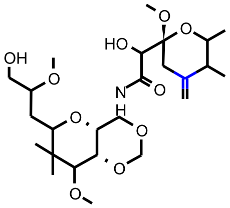 [*][c](:[*]):[*]	-0.247
ECFP_6	-1884411803	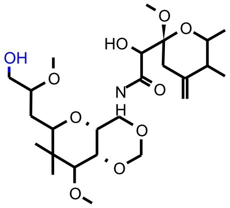 [*]O	-0.217

ECFP_6	-1059365320	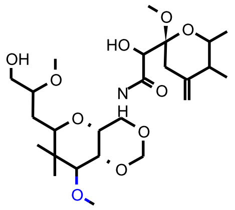 [*]O[*]	-0.184

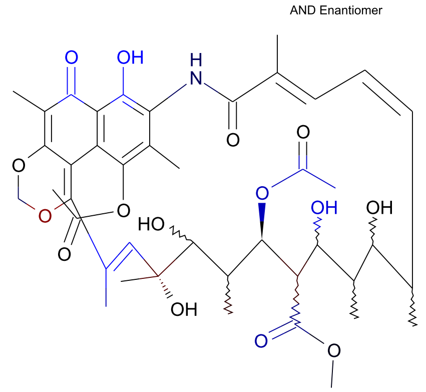

$C_{42}H_{53}NO_{15}$
Molecular Weight: 811.86791
ALogP: 2.745
Rotatable Bonds: 6
Acceptors: 15
Donors: 6

Model Prediction

Prediction: 12.946
Unit: mg/kg_body_weight/day
Mahalanobis Distance: 14.885
Mahalanobis Distance p-value: 9.85e-013

Mahalanobis Distance: The Mahalanobis distance (MD) is a generalization of the Euclidean distance that accounts for correlations among the X properties. It is calculated as the distance to the center of the training data. The larger the MD, the less trustworthy the prediction.
Mahalanobis Distance p-value: The p-value gives the fraction of training data with an MD greater than or equal to the one for the given sample, assuming normally distributed data. The smaller the p-value, the less trustworthy the prediciton. For highly non-normal X properties (e.g., fingerprints), the MD p-value is wildly inaccurate.

Structural Similar Compounds

Name	532	380	338
Structure	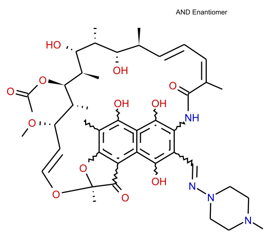	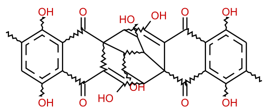	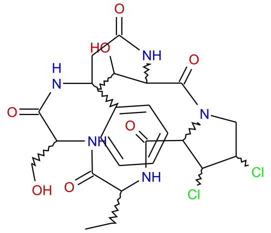
Actual Endpoint (-log C)	4.38903	4.48977	4.39533
Predicted Endpoint (-log C)	5.60554	6.22716	4.31268
Distance	0.615	1.180	1.201
Reference	CPDB	CPDB	CPDB

Model Applicability

Unknown features are fingerprint features in the query molecule, but not found in the training set.

1. All properties and OPS components are within expected ranges.
2. Unknown ECFP_2 feature: -40769921: [*]C([*])[C@](C)(O)C=[*]
3. Unknown ECFP_2 feature: 464446479: [*]C(=CC([*])([*])[*])[*]
4. Unknown ECFP_2 feature: 1796421070: [*]OC(=C([*])[*])C(=[*])[*]
5. Unknown ECFP_2 feature: 1792159373: [*]C(=C(C)C(=[*])[*])[*]

Feature Contribution

Top features for positive contribution

Fingerprint	Bit/Smiles	Feature Structure	Score
ECFP_6	683445015	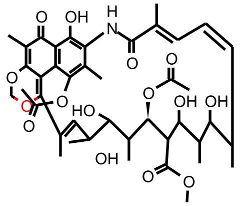 [*]O[*]	0.136

ECFP_6	657586427	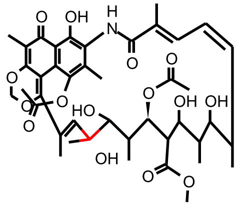 [*]C([*])([*])[*]	0.079
ECFP_6	-167460056	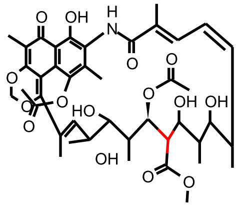 [*]C([*])[*]	0.060

Top Features for negative contribution

Fingerprint	Bit/Smiles	Feature Structure	Score
ECFP_6	2106656448	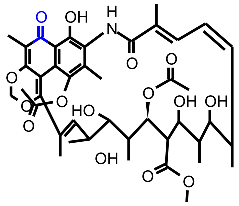 [*]C(=O)[*]	-0.275
ECFP_6	2019062761	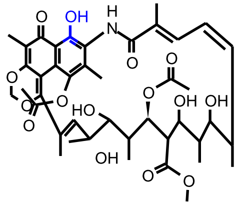 [*]:[c](:[*])O	-0.258
ECFP_6	642810091	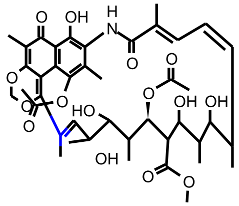 [*][c](:[*]):[*]	-0.247

Simeprevir

TOPKAT_Carcinogenic_Potency_TD50_Mouse

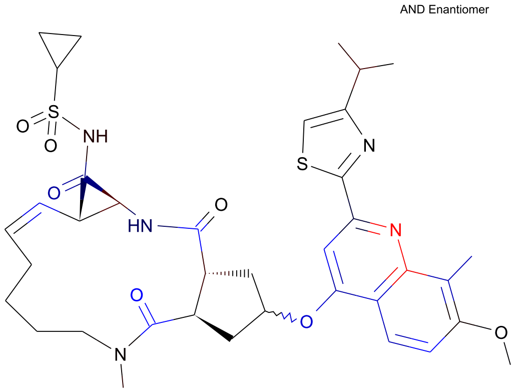

$C_{38}H_{47}N_5O_7S_2$

Molecular Weight: 749.93907

ALogP: 4.796

Rotatable Bonds: 8

Acceptors: 9

Donors: 2

Structural Similar Compounds

Name	223	D & C red no. 5	Salicylazosulfapyridine
Structure	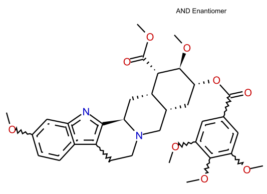	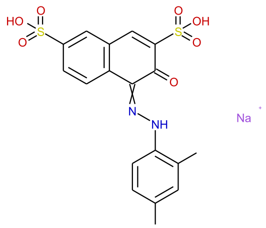	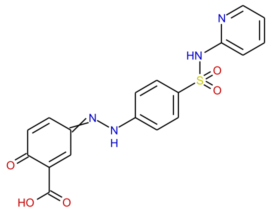
Actual Endpoint (-log C)	5.08368	2.80732	2.5034
Predicted Endpoint (-log C)	5.08273	3.78615	3.54214
Distance	0.926	1.173	1.177
Reference	CPDB	CPDB	CPDB

Model Prediction

Prediction: 2.014

Unit: mg/kg_body_weight/day

Mahalanobis Distance: 16.839

Mahalanobis Distance p-value: 5.81e-019

Mahalanobis Distance: The Mahalanobis distance (MD) is a generalization of the Euclidean distance that accounts for correlations among the X properties. It is calculated as the distance to the center of the training data. The larger the MD, the less trustworthy the prediction.

Mahalanobis Distance p-value: The p-value gives the fraction of training data with an MD greater than or equal to the one for the given sample, assuming normally distributed data. The smaller the p-value, the less trustworthy the prediciton. For highly non-normal X properties (e.g., fingerprints), the MD p-value is wildly inaccurate.

Model Applicability

Unknown features are fingerprint features in the query molecule, but not found in the training set.

1. OPS PC14 out of range. Value: -4.1139. Training min, max, SD, explained variance: -3.6133, 3.7483, 1.312, 0.0215.
2. OPS PC16 out of range. Value: 4.4468. Training min, max, SD, explained variance: -3.1026, 4.016, 1.245, 0.0193.
3. Unknown ECFP_2 feature: -309676563: [*]N[C@]1(C[C@H]1[*])C(=[*])[*]
4. Unknown ECFP_2 feature: -1049290660: [*]C1([*])C[C@H]1C=[*]
5. Unknown ECFP_2 feature: -1818486371: [*]NC(=O)C1([*])[*][*]1
6. Unknown ECFP_2 feature: 946167604: [*]C(=[*])NS(=[*])(=[*])[*]
7. Unknown ECFP_2 feature: 1310940530: [*]C([*])O[c](:[*]):[*]
8. Unknown ECFP_2 feature: 1411720546: [*]C([*])[c]1:n:[*]:[*]:c:1
9. Unknown ECFP_2 feature: 733491677: [*]:[c](:[*])C(C)C
10. Unknown ECFP_2 feature: 2121941848: [*]NS(=O)(=O)C1[*][*]1
11. Unknown ECFP_2 feature: -622223421: [*]S(=[*])(=[*])C1CC1

Feature Contribution

Top features for positive contribution			
**Fingerprint**	**Bit/Smiles**	**Feature Structure**	**Score**

ECFP_6	655739385	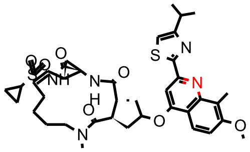 [*]:n:[*]	0.229
ECFP_6	834876373	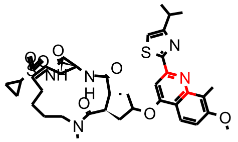 [*][c](:[*]):n:[c](:[*]):[*]	0.163
ECFP_6	657586427	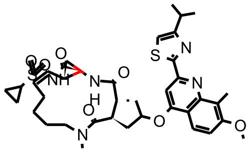 [*]C([*])([*])[*]	0.079

Top Features for negative contribution

Fingerprint	Bit/Smiles	Feature Structure	Score
ECFP_6	2106656448	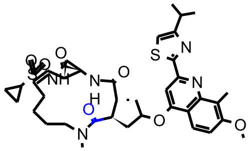 [*]C(=O)[*]	-0.275
ECFP_6	1996767644	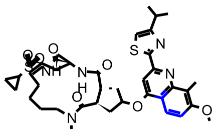 [*][c](:[*]):[cH]:[cH]:[*]	-0.251

ECFP_6	642810091	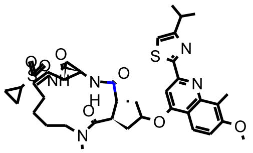 [*][c](:[*]):[*]	-0.247

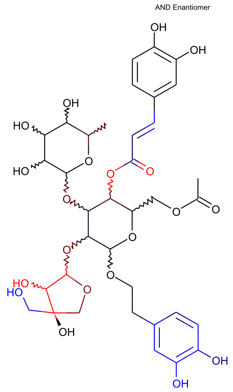

$C_{36}H_{46}O_{20}$
Molecular Weight: 798.73843
ALogP: -0.546
Rotatable Bonds: 16
Acceptors: 20
Donors: 10

Model Prediction

Prediction: 1.086
Unit: mg/kg_body_weight/day
Mahalanobis Distance: 17.246
Mahalanobis Distance p-value: 1.61e-018

Mahalanobis Distance: The Mahalanobis distance (MD) is a generalization of the Euclidean distance that accounts for correlations among the X properties. It is calculated as the distance to the center of the training data. The larger the MD, the less trustworthy the prediction.
Mahalanobis Distance p-value: The p-value gives the fraction of training data with an MD greater than or equal to the one for the given sample, assuming normally distributed data. The smaller the p-value, the less trustworthy the prediciton. For highly non-normal X properties (e.g., fingerprints), the MD p-value is wildly inaccurate.

Structural Similar Compounds

Name	764	706	186
Structure	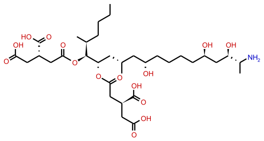	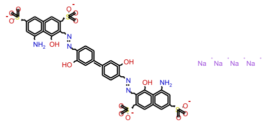	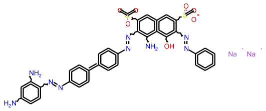
Actual Endpoint (-log C)	5.79398	2.74451	5.72371
Predicted Endpoint (-log C)	4.15405	4.36978	5.06873
Distance	1.386	1.460	1.464
Reference	CPDB	CPDB	CPDB

Model Applicability

Unknown features are fingerprint features in the query molecule, but not found in the training set.

1. Num_H_Donors out of range. Value: 10. Training min, max, mean, SD: 0, 8, 1.0519, 1.223.

Feature Contribution

Top features for positive contribution

Fingerprint	Bit/Smiles	Feature Structure	Score
FCFP_6	-1043250487	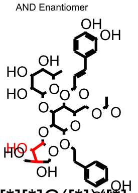 [*]C1[*][*]C([*])([*])C1O	1.153

FCFP_6	565998553	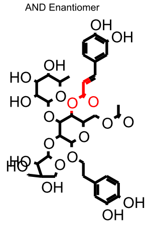 [*]OC(=O)C=[*]	0.357
FCFP_6	1	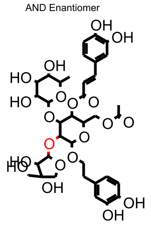 [*]O[*]	0.234

Top Features for negative contribution

Fingerprint	Bit/Smiles	Feature Structure	Score
FCFP_6	-1272709286	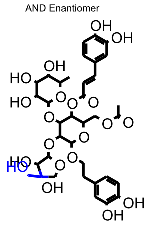 [*]C([*])([*])CO	-0.526
FCFP_6	451847724	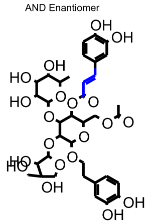 [*]\C=C\C(=[*])[*]	-0.436
FCFP_6	7	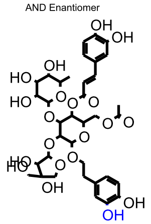 [*]O	-0.372

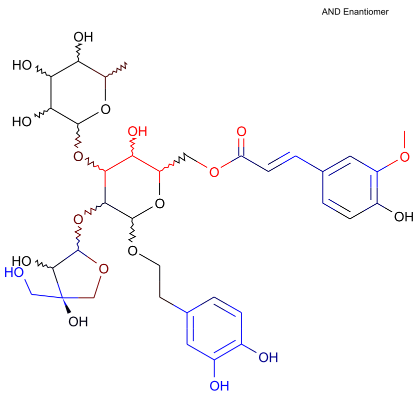

$C_{35}H_{46}O_{19}$

Molecular Weight: 770.72833

ALogP: -0.699

Rotatable Bonds: 15

Acceptors: 19

Donors: 10

Structural Similar Compounds

Name	764	186	706
Structure	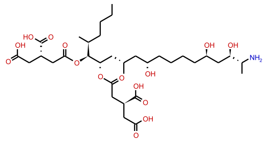	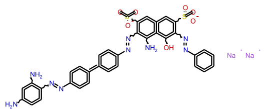	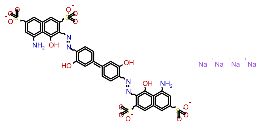
Actual Endpoint (-log C)	5.79398	5.72371	2.74451
Predicted Endpoint (-log C)	4.15405	5.06873	4.36978
Distance	1.402	1.434	1.493
Reference	CPDB	CPDB	CPDB

Model Applicability

Unknown features are fingerprint features in the query molecule, but not found in the training set.

1. Num_H_Donors out of range. Value: 10. Training min, max, mean, SD: 0, 8, 1.0519, 1.223.
2. OPS PC20 out of range. Value: -4.5225. Training min, max, SD, explained variance: -3.9266, 5.5565, 1.236, 0.0147.

Model Prediction

Prediction: 0.340

Unit: mg/kg_body_weight/day

Mahalanobis Distance: 18.842

Mahalanobis Distance p-value: 1.85e-024

Mahalanobis Distance: The Mahalanobis distance (MD) is a generalization of the Euclidean distance that accounts for correlations among the X properties. It is calculated as the distance to the center of the training data. The larger the MD, the less trustworthy the prediction.

Mahalanobis Distance p-value: The p-value gives the fraction of training data with an MD greater than or equal to the one for the given sample, assuming normally distributed data. The smaller the p-value, the less trustworthy the prediciton. For highly non-normal X properties (e.g., fingerprints), the MD p-value is wildly inaccurate.

Feature Contribution

Top features for positive contribution

Fingerprint	Bit/Smiles	Feature Structure	Score
FCFP_6	-1043250487	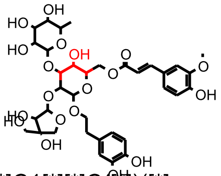 [*]C1[*][*]C([*])([*])C1O	1.153

FCFP_6	136627117	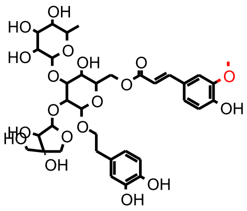 [*]OC	0.690
FCFP_6	565998553	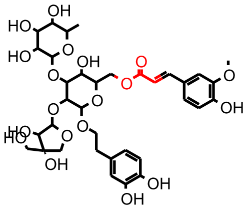 [*]OC(=O)C=[*]	0.357

Top Features for negative contribution

Fingerprint	Bit/Smiles	Feature Structure	Score
FCFP_6	-1272709286	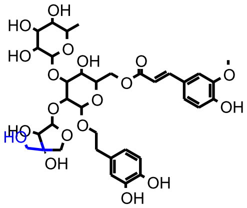 [*]C([*])([*])CO	-0.526
FCFP_6	451847724	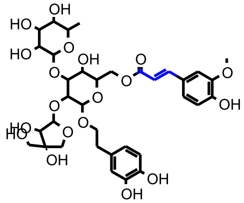 [*]\C=C\C(=[*])[*]	-0.436
FCFP_6	7	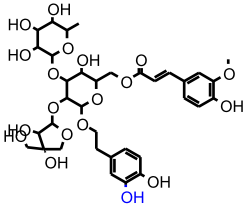 [*]O	-0.372

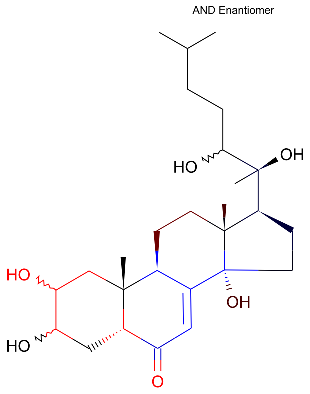

$C_{27}H_{44}O_6$
Molecular Weight: 464.63466
ALogP: 2.489
Rotatable Bonds: 5
Acceptors: 6
Donors: 5

Model Prediction

Prediction: 0.361
Unit: mg/kg_body_weight/day
Mahalanobis Distance: 10.702
Mahalanobis Distance p-value: 0.0425

Mahalanobis Distance: The Mahalanobis distance (MD) is a generalization of the Euclidean distance that accounts for correlations among the X properties. It is calculated as the distance to the center of the training data. The larger the MD, the less trustworthy the prediction.
Mahalanobis Distance p-value: The p-value gives the fraction of training data with an MD greater than or equal to the one for the given sample, assuming normally distributed data. The smaller the p-value, the less trustworthy the prediciton. For highly non-normal X properties (e.g., fingerprints), the MD p-value is wildly inaccurate.

Structural Similar Compounds

Name	1085	Prednisolone	Budesonide
Structure	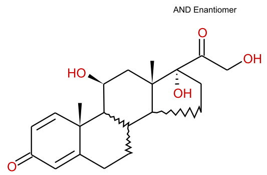	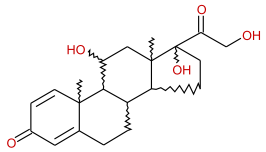	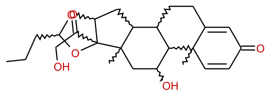
Actual Endpoint (-log C)	5.37215	5.37215	6.17011
Predicted Endpoint (-log C)	5.32932	5.32932	5.6493
Distance	0.729	0.729	0.818
Reference	CPDB	CPDB	CPDB

Model Applicability

Unknown features are fingerprint features in the query molecule, but not found in the training set.

1. All properties and OPS components are within expected ranges.

Feature Contribution

Top features for positive contribution

Fingerprint	Bit/Smiles	Feature Structure	Score
FCFP_6	-1043250487	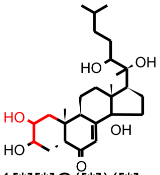 [*]C1[*][*]C([*])([*])C1O	1.153

FCFP_6	565968762	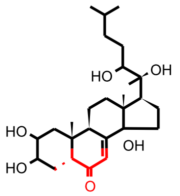 [*]C([*])C(=O)C=[*]	0.266
FCFP_6	1	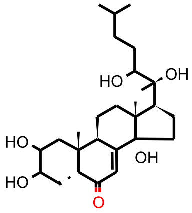 [*]O[*]	0.234

Top Features for negative contribution

**Fingerprint**	**Bit/Smiles**	**Feature Structure**	**Score**
FCFP_6	451847724	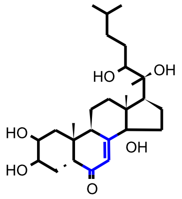 [*]\C=C\C(=[*])[*]	-0.436
FCFP_6	436886043	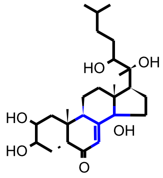 [*]\C=C(/C([*])[*])\C([*])([*])[*]	-0.383
FCFP_6	0	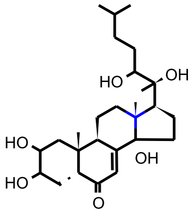 [*]C([*])[*]	-0.115

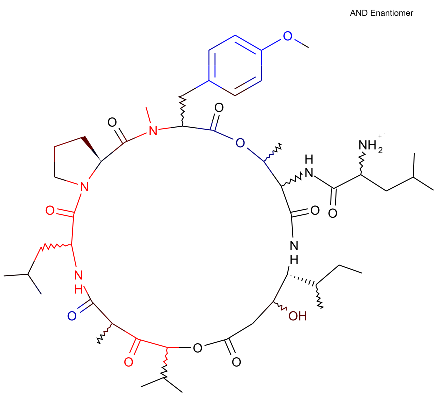

$C_{49}H_{79}N_6O_{12}$
Molecular Weight: 944.18455
ALogP: 3.176
Rotatable Bonds: 13
Acceptors: 12
Donors: 5

Model Prediction

Prediction: 0.053
Unit: mg/kg_body_weight/day
Mahalanobis Distance: 17.444
Mahalanobis Distance p-value: 3.14e-019

Mahalanobis Distance: The Mahalanobis distance (MD) is a generalization of the Euclidean distance that accounts for correlations among the X properties. It is calculated as the distance to the center of the training data. The larger the MD, the less trustworthy the prediction.
Mahalanobis Distance p-value: The p-value gives the fraction of training data with an MD greater than or equal to the one for the given sample, assuming normally distributed data. The smaller the p-value, the less trustworthy the prediciton. For highly non-normal X properties (e.g., fingerprints), the MD p-value is wildly inaccurate.

Structural Similar Compounds

Name	332	186	Fumonisin B1
Structure	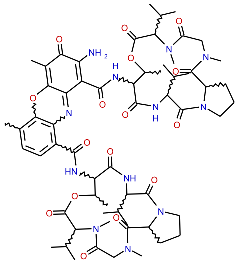	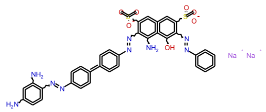	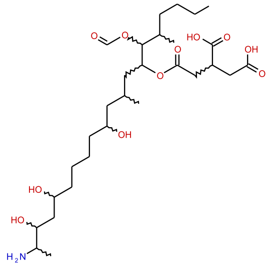
Actual Endpoint (-log C)	9.05347	5.72371	5.59603
Predicted Endpoint (-log C)	8.32884	5.06873	4.17651
Distance	1.305	1.353	1.357
Reference	CPDB	CPDB	CPDB

Model Applicability

Unknown features are fingerprint features in the query molecule, but not found in the training set.

1. All properties and OPS components are within expected ranges.
2. Unknown FCFP_2 feature: 136418580: [*][NH2+]C

Feature Contribution

Top features for positive contribution			
**Fingerprint**	**Bit/Smiles**	**Feature Structure**	**Score**
FCFP_6	-1043250487	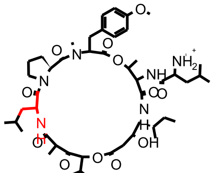 [*]C1[*][*]C([*])([*])C1O	1.153

FCFP_6	136627117	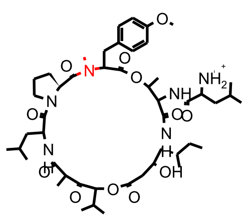 [*]OC	0.690
FCFP_6	565998553	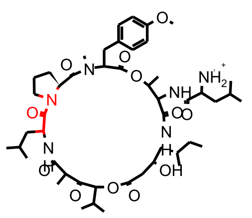 [*]OC(=O)C=[*]	0.357

Top Features for negative contribution

**Fingerprint**	**Bit/Smiles**	**Feature Structure**	**Score**
FCFP_6	16	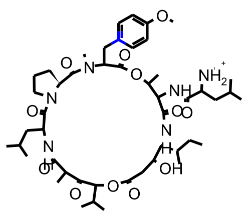 [*][c](:[*]):[*]	-0.354
FCFP_6	1674451008	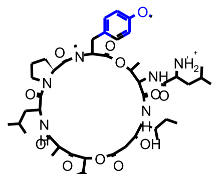 [*]O[c]1:[cH]:[*]:[c] ([*]):[cH]:[cH]:1	-0.233
FCFP_6	566058135	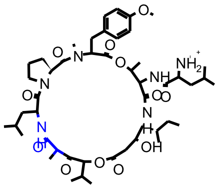 [*]NC(=O)C([*])[*]	-0.182

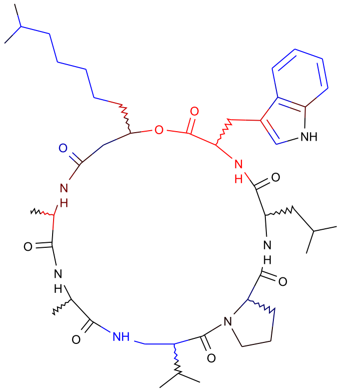

$C_{45}H_{69}N_7O_8$

Molecular Weight: 836.07145

ALogP: 4.986

Rotatable Bonds: 11

Acceptors: 8

Donors: 6

Model Prediction

Prediction: 1.612

Unit: mg/kg_body_weight/day

Mahalanobis Distance: 18.754

Mahalanobis Distance p-value: 4.02e-024

Mahalanobis Distance: The Mahalanobis distance (MD) is a generalization of the Euclidean distance that accounts for correlations among the X properties. It is calculated as the distance to the center of the training data. The larger the MD, the less trustworthy the prediction.
Mahalanobis Distance p-value: The p-value gives the fraction of training data with an MD greater than or equal to the one for the given sample, assuming normally distributed data. The smaller the p-value, the less trustworthy the prediciton. For highly non-normal X properties (e.g., fingerprints), the MD p-value is wildly inaccurate.

Structural Similar Compounds

Name	551	411	188
Structure	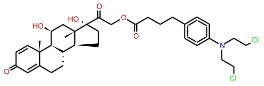	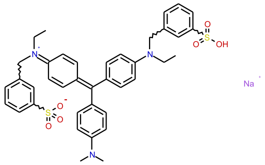	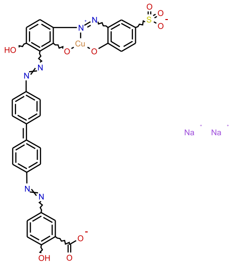
Actual Endpoint (-log C)	4.52736	3.06566	5.5378
Predicted Endpoint (-log C)	6.81053	4.8672	5.71925
Distance	1.234	1.235	1.280
Reference	CPDB	CPDB	CPDB

Model Applicability

Unknown features are fingerprint features in the query molecule, but not found in the training set.

1. All properties and OPS components are within expected ranges.

Feature Contribution

Top features for positive contribution			
**Fingerprint**	**Bit/Smiles**	**Feature Structure**	**Score**
FCFP_6	-1043250487	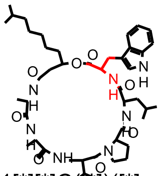 [*]C1[*][*]C([*])([*])C1O	1.153

FCFP_6	565998553	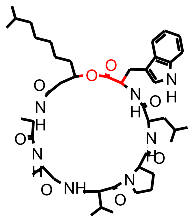<br>[*]OC(=O)C=[*]	0.357
FCFP_6	1	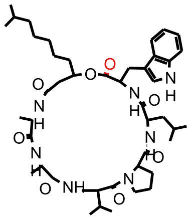<br>[*]O[*]	0.234

Top Features for negative contribution

Fingerprint	Bit/Smiles	Feature Structure	Score
FCFP_6	-1272709286	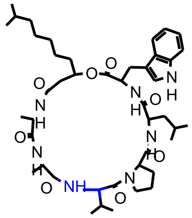<br>[*]C([*])([*])CO	-0.526
FCFP_6	1175638033	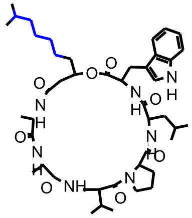<br>[*]CCCCC([*])[*]	-0.512
FCFP_6	991735244	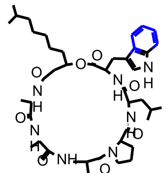<br>[*]:[c]1:[*]:[cH]:[cH<br>]:[cH]:[cH]:1	-0.422

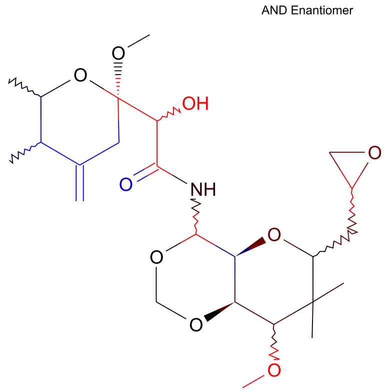

$C_{24}H_{39}NO_9$

Molecular Weight: 485.56776

ALogP: 0.267

Rotatable Bonds: 7

Acceptors: 9

Donors: 2

Model Prediction

Prediction: 0.032

Unit: mg/kg_body_weight/day

Mahalanobis Distance: 13.800

Mahalanobis Distance p-value: 6.77e-008

Mahalanobis Distance: The Mahalanobis distance (MD) is a generalization of the Euclidean distance that accounts for correlations among the X properties. It is calculated as the distance to the center of the training data. The larger the MD, the less trustworthy the prediction.
Mahalanobis Distance p-value: The p-value gives the fraction of training data with an MD greater than or equal to the one for the given sample, assuming normally distributed data. The smaller the p-value, the less trustworthy the prediciton. For highly non-normal X properties (e.g., fingerprints), the MD p-value is wildly inaccurate.

Structural Similar Compounds

Name	199	Lasiocarpine	Actinomycin D
Structure	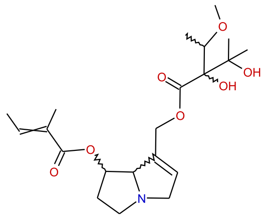	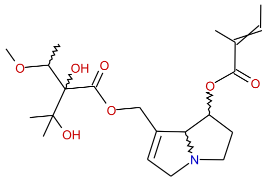	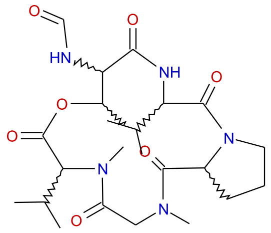
Actual Endpoint (-log C)	5.93675	6.02441	8.6619
Predicted Endpoint (-log C)	5.63437	5.63437	6.62157
Distance	0.731	0.731	0.761
Reference	CPDB	CPDB	CPDB

Model Applicability

Unknown features are fingerprint features in the query molecule, but not found in the training set.

1. All properties and OPS components are within expected ranges.

Feature Contribution

Top features for positive contribution			
**Fingerprint**	**Bit/Smiles**	**Feature Structure**	**Score**
FCFP_6	-1043250487	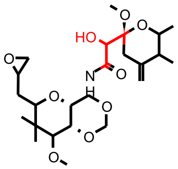 [*]C1[*][*]C([*])([*])C1O	1.153

FCFP_6	136627117	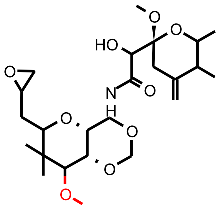 [*]OC	0.690
FCFP_6	1	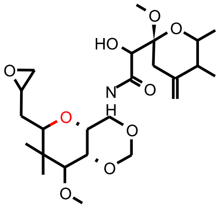 [*]O[*]	0.234
**Top Features for negative contribution**			
**Fingerprint**	**Bit/Smiles**	**Feature Structure**	**Score**
FCFP_6	436886043	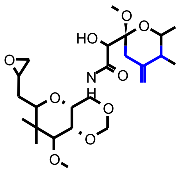 [*]\C=C(/C([*])[*])\C([*])([*])[*]	-0.383
FCFP_6	566058135	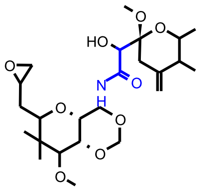 [*]NC(=O)C([*])[*]	-0.182
FCFP_6	-1143715940	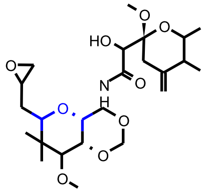 [*]C([*])OC([*])[*]	-0.124

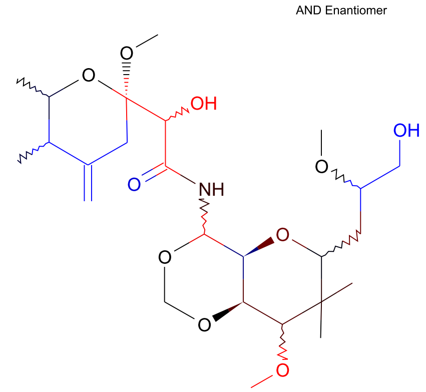

$C_{25}H_{43}NO_{10}$
Molecular Weight: 517.60962
ALogP: -9.1e-002
Rotatable Bonds: 9
Acceptors: 10
Donors: 3

Model Prediction

Prediction: 0.335
Unit: mg/kg_body_weight/day
Mahalanobis Distance: 13.235
Mahalanobis Distance p-value: 1.58e-006

Mahalanobis Distance: The Mahalanobis distance (MD) is a generalization of the Euclidean distance that accounts for correlations among the X properties. It is calculated as the distance to the center of the training data. The larger the MD, the less trustworthy the prediction.
Mahalanobis Distance p-value: The p-value gives the fraction of training data with an MD greater than or equal to the one for the given sample, assuming normally distributed data. The smaller the p-value, the less trustworthy the prediciton. For highly non-normal X properties (e.g., fingerprints), the MD p-value is wildly inaccurate.

Structural Similar Compounds

Name	199	Lasiocarpine	Actinomycin D
Structure	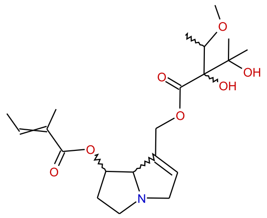	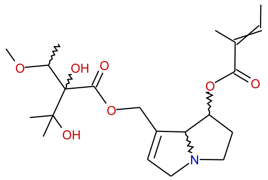	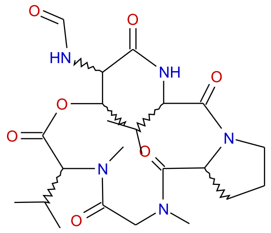
Actual Endpoint (-log C)	5.93675	6.02441	8.6619
Predicted Endpoint (-log C)	5.63437	5.63437	6.62157
Distance	0.749	0.749	0.781
Reference	CPDB	CPDB	CPDB

Model Applicability

Unknown features are fingerprint features in the query molecule, but not found in the training set.

1. All properties and OPS components are within expected ranges.

Feature Contribution

Top features for positive contribution			
Fingerprint	Bit/Smiles	Feature Structure	Score
FCFP_6	-1043250487	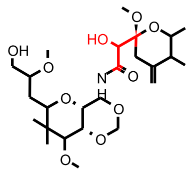 [*]C1[*][*]C([*])([*])C1O	1.153

FCFP_6	136627117	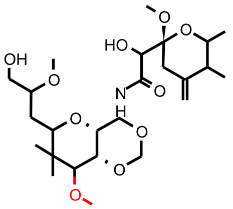 [*]OC	0.690
FCFP_6	1	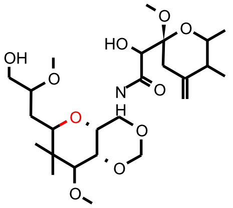 [*]O[*]	0.234

Top Features for negative contribution

**Fingerprint**	**Bit/Smiles**	**Feature Structure**	**Score**
FCFP_6	-1272709286	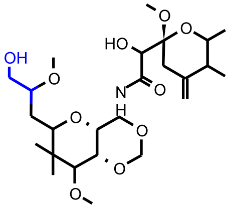 [*]C([*])([*])CO	-0.526
FCFP_6	436886043	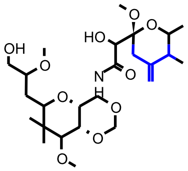 [*]\C=C(/C([*])[*])\C([*])([*])[*]	-0.383
FCFP_6	566058135	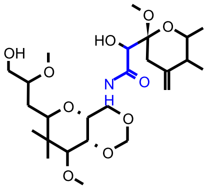 [*]NC(=O)C([*])[*]	-0.182

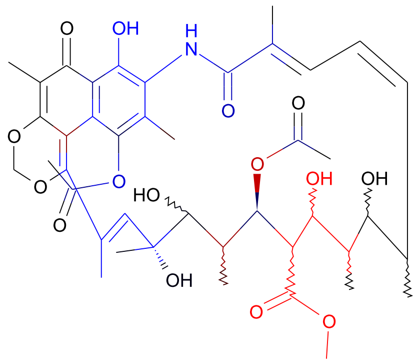

$C_{42}H_{53}NO_{15}$
Molecular Weight: 811.86791
ALogP: 2.745
Rotatable Bonds: 6
Acceptors: 15
Donors: 6

Model Prediction

Prediction: 1.527
Unit: mg/kg_body_weight/day
Mahalanobis Distance: 19.242
Mahalanobis Distance p-value: 4.99e-026

Mahalanobis Distance: The Mahalanobis distance (MD) is a generalization of the Euclidean distance that accounts for correlations among the X properties. It is calculated as the distance to the center of the training data. The larger the MD, the less trustworthy the prediction.
Mahalanobis Distance p-value: The p-value gives the fraction of training data with an MD greater than or equal to the one for the given sample, assuming normally distributed data. The smaller the p-value, the less trustworthy the prediciton. For highly non-normal X properties (e.g., fingerprints), the MD p-value is wildly inaccurate.

Structural Similar Compounds

Name	186	424	188
Structure	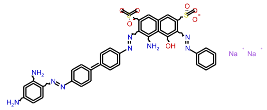	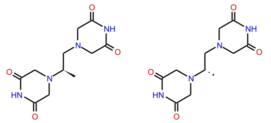	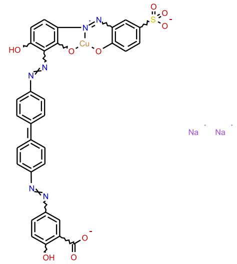
Actual Endpoint (-log C)	5.72371	4.70022	5.5378
Predicted Endpoint (-log C)	5.06873	5.67571	5.71925
Distance	1.229	1.239	1.271
Reference	CPDB	CPDB	CPDB

Model Applicability

Unknown features are fingerprint features in the query molecule, but not found in the training set.

1. All properties and OPS components are within expected ranges.

Feature Contribution

Top features for positive contribution			
**Fingerprint**	**Bit/Smiles**	**Feature Structure**	**Score**
FCFP_6	-1043250487	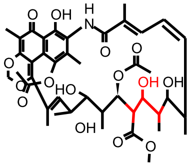 [*]C1[*][*]C([*])([*])C1O	1.153

FCFP_6	136627117	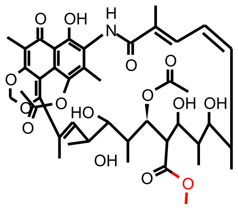 [*]OC	0.690
FCFP_6	565998553	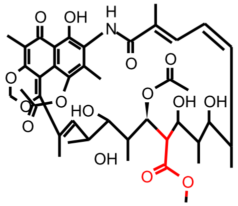 [*]OC(=O)C=[*]	0.357

Top Features for negative contribution

**Fingerprint**	**Bit/Smiles**	**Feature Structure**	**Score**
FCFP_6	451847724	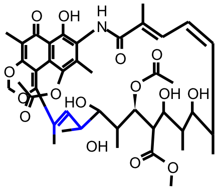 [*]\C=C\C(=[*])[*]	-0.436
FCFP_6	436886043	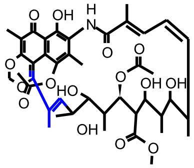 [*]\C=C(/C([*])[*])\C ([*])([*])[*]	-0.383
FCFP_6	7	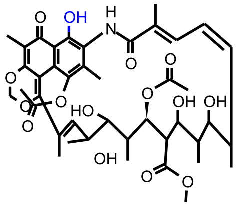 [*]O	-0.372

Simeprevir

TOPKAT_Carcinogenic_Potency_TD50_Rat

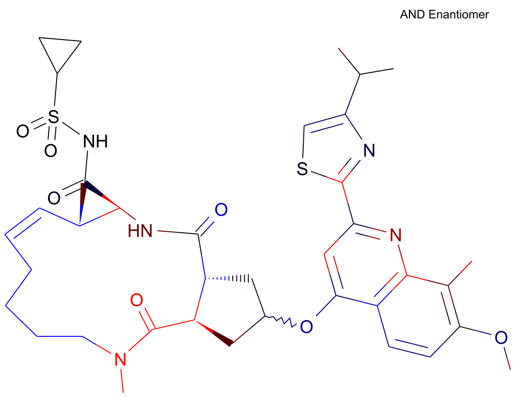

$C_{38}H_{47}N_5O_7S_2$
Molecular Weight: 749.93907
ALogP: 4.796
Rotatable Bonds: 8
Acceptors: 9
Donors: 2

Model Prediction

Prediction: 0.280
Unit: mg/kg_body_weight/day
Mahalanobis Distance: 24.067
Mahalanobis Distance p-value: 1.44e-046

Mahalanobis Distance: The Mahalanobis distance (MD) is a generalization of the Euclidean distance that accounts for correlations among the X properties. It is calculated as the distance to the center of the training data. The larger the MD, the less trustworthy the prediction.
Mahalanobis Distance p-value: The p-value gives the fraction of training data with an MD greater than or equal to the one for the given sample, assuming normally distributed data. The smaller the p-value, the less trustworthy the prediciton. For highly non-normal X properties (e.g., fingerprints), the MD p-value is wildly inaccurate.

Structural Similar Compounds

Name	411	223	188
Structure	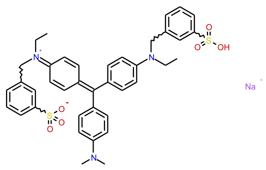	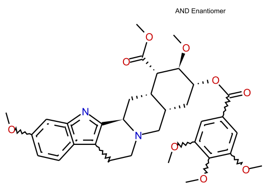	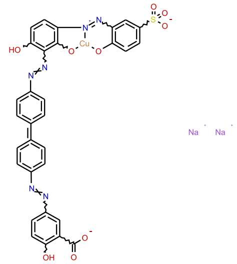
Actual Endpoint (-log C)	3.06566	6.29867	5.5378
Predicted Endpoint (-log C)	4.8672	7.5657	5.71925
Distance	0.846	0.861	0.891
Reference	CPDB	CPDB	CPDB

Model Applicability

Unknown features are fingerprint features in the query molecule, but not found in the training set.

1. OPS PC27 out of range. Value: -3.3094. Training min, max, SD, explained variance: -2.8642, 4.2058, 1.002, 0.0096.

Feature Contribution

Top features for positive contribution

Fingerprint	Bit/Smiles	Feature Structure	Score
FCFP_6	136627117	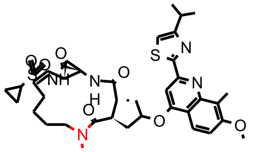 [*]OC	0.690

FCFP_6	565998553	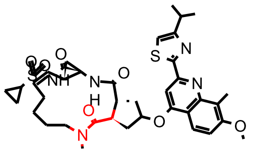<br>[*]OC(=O)C=[*]	0.357
FCFP_6	690511177	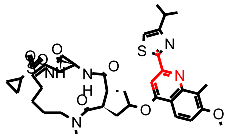<br>[*]:[cH]:[c](:n:[*])[<br>c](:[*]):[*]	0.293
**Top Features for negative contribution**			
**Fingerprint**	**Bit/Smiles**	**Feature Structure**	**Score**
FCFP_6	1175638033	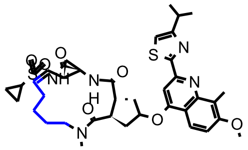<br>[*]CCCCC([*])[*]	-0.512
FCFP_6	451847724	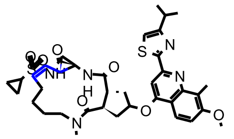<br>[*]\C=C\C(=[*])[*]	-0.436
FCFP_6	16	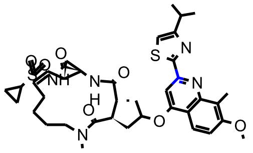<br>[*][c](:[*]):[*]	-0.354

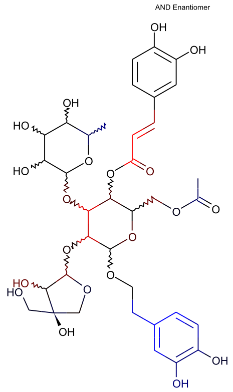

$C_{36}H_{46}O_{20}$

Molecular Weight: 798.73843

ALogP: -0.546

Rotatable Bonds: 16

Acceptors: 20

Donors: 10

Structural Similar Compounds

Name	CARMINE	C.I. DIRECT BLUE 15	OXYTETRACYCLINE .HCL
Structure	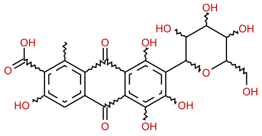	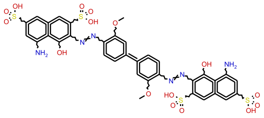	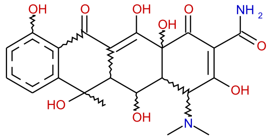
Actual Endpoint (-log C)	3.5162	4.3131	2.56626
Predicted Endpoint (-log C)	3.66829	3.72681	3.75581
Distance	1.385	1.492	1.728
Reference	FOOD.CHEM.TOXICOL.25.897.1987	NTP REPORT # 397	NTP REPORT # 315

Model Prediction

Prediction: 0.040

Unit: g/kg_body_weight

Mahalanobis Distance: 44.607

Mahalanobis Distance p-value: 1.46e-049

Mahalanobis Distance: The Mahalanobis distance (MD) is a generalization of the Euclidean distance that accounts for correlations among the X properties. It is calculated as the distance to the center of the training data. The larger the MD, the less trustworthy the prediction.
Mahalanobis Distance p-value: The p-value gives the fraction of training data with an MD greater than or equal to the one for the given sample, assuming normally distributed data. The smaller the p-value, the less trustworthy the prediciton. For highly non-normal X properties (e.g., fingerprints), the MD p-value is wildly inaccurate.

Model Applicability

Unknown features are fingerprint features in the query molecule, but not found in the training set.

1. Num_H_Donors out of range. Value: 10. Training min, max, mean, SD: 0, 9, 1.0902, 1.316.
2. Unknown ECFP_6 feature: 456242574: [*]C([*])OC([*])[*]
3. Unknown ECFP_6 feature: -1409796893: [*]C([*])OC([*])[*]
4. Unknown ECFP_6 feature: -1939757055: [*]C1[*][*]CO1
5. Unknown ECFP_6 feature: 456978524: [*]C([*])OC(=[*])[*]
6. Unknown ECFP_6 feature: -1250019913: [*]COC([*])[*]
7. Unknown ECFP_6 feature: 2024749573: [*]C([*])O
8. Unknown ECFP_6 feature: 2018700401: [*]C([*])([*])O
9. Unknown ECFP_6 feature: 2022454958: [*]CO
10. Unknown ECFP_6 feature: 2019062761: [*]:[c](:[*])O
11. Unknown ECFP_6 feature: 2011659532: [*]OC(C([*])[*])C([*])[*]
12. Unknown ECFP_6 feature: -553149446: [*]CC(O[*])C([*])[*]
13. Unknown ECFP_6 feature: -2060414325: [*]OC(O[*])C([*])[*]
14. Unknown ECFP_6 feature: -521596699: [*]C1[*][*]C([*])([*])C1O
15. Unknown ECFP_6 feature: 1953003528: [*]C[C@@]1(O)C[*][*]C1[*]
16. Unknown ECFP_6 feature: 305695353: [*]C([*])C(O)C([*])[*]
17. Unknown ECFP_6 feature: 414215731: [*]C1([*])[*][*]OC1
18. Unknown ECFP_6 feature: -1687549011: [*]OCC([*])[*]
19. Unknown ECFP_6 feature: 1535429263: [*]OC(C)C([*])[*]
20. Unknown ECFP_6 feature: 1129597062: [*]C([*])([*])CO

21. Unknown ECFP_6 feature: 865482986: [*]C([*])C
22. Unknown ECFP_6 feature: -1790412586: [*]CCO[*]
23. Unknown ECFP_6 feature: -1684512787: [*]OC(=O)C=[*]
24. Unknown ECFP_6 feature: -1795525632: [*]CC[c](:[*]):[*]
25. Unknown ECFP_6 feature: -473922720: [*]OC(=O)C
26. Unknown ECFP_6 feature: -470416293: [*]\C=C\C(=[*])[*]
27. Unknown ECFP_6 feature: -1831055759: [*]\C=C\[c](:[*]):[*]
28. Unknown ECFP_6 feature: -176483725: [*]=C[c](:[cH]:[*]):[cH]:[*]

Feature Contribution

Top features for positive contribution

Fingerprint	Bit/Smiles	Feature Structure	Score
FCFP_6	451847724	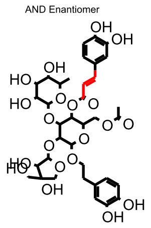 [*]\C=C\C(=[*])[*]	0.160
ECFP_6	-167460056	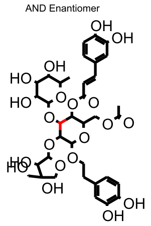 [*]C([*])[*]	0.136
FCFP_6	-1143715940	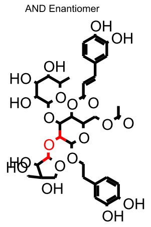 [*]C([*])OC([*])[*]	0.130

Top Features for negative contribution

Fingerprint	Bit/Smiles	Feature Structure	Score

FCFP_6	1	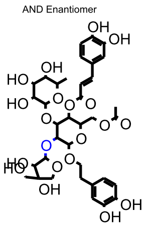 [*]O[*]	-0.102
FCFP_6	-453677277	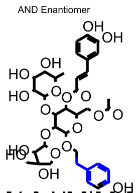 [*]C[c]1:[cH]:[*]:[c]([*]):[cH]:[cH]:1	-0.091
FCFP_6	136597326	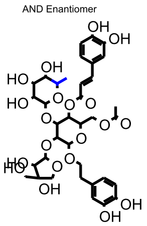 [*]C([*])C	-0.081

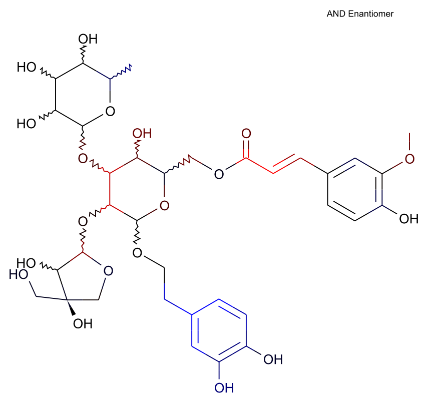

$C_{35}H_{46}O_{19}$
Molecular Weight: 770.72833
ALogP: -0.699
Rotatable Bonds: 15
Acceptors: 19
Donors: 10

Model Prediction

Prediction: 0.017
Unit: g/kg_body_weight
Mahalanobis Distance: 45.301
Mahalanobis Distance p-value: 1.23e-050

Mahalanobis Distance: The Mahalanobis distance (MD) is a generalization of the Euclidean distance that accounts for correlations among the X properties. It is calculated as the distance to the center of the training data. The larger the MD, the less trustworthy the prediction.
Mahalanobis Distance p-value: The p-value gives the fraction of training data with an MD greater than or equal to the one for the given sample, assuming normally distributed data. The smaller the p-value, the less trustworthy the prediciton. For highly non-normal X properties (e.g., fingerprints), the MD p-value is wildly inaccurate.

Structural Similar Compounds

Name	CARMINE	C.I. DIRECT BLUE 15	OXYTETRACYCLINE .HCL
Structure	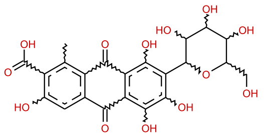	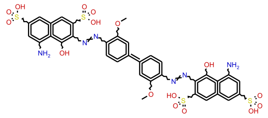	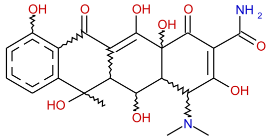
Actual Endpoint (-log C)	3.5162	4.3131	2.56626
Predicted Endpoint (-log C)	3.66829	3.72681	3.75581
Distance	1.278	1.541	1.612
Reference	FOOD.CHEM.TOXICOL.25.897.1987	NTP REPORT # 397	NTP REPORT # 315

Model Applicability

Unknown features are fingerprint features in the query molecule, but not found in the training set.

1. Num_H_Donors out of range. Value: 10. Training min, max, mean, SD: 0, 9, 1.0902, 1.316.
2. Unknown ECFP_6 feature: 456242574: [*]C([*])OC([*])[*]
3. Unknown ECFP_6 feature: -1409796893: [*]C([*])OC([*])[*]
4. Unknown ECFP_6 feature: -1939757055: [*]C1[*][*]CO1
5. Unknown ECFP_6 feature: -1250019913: [*]COC([*])[*]
6. Unknown ECFP_6 feature: 2024749573: [*]C([*])O
7. Unknown ECFP_6 feature: 2018700401: [*]C([*])([*])O
8. Unknown ECFP_6 feature: 2022454958: [*]CO
9. Unknown ECFP_6 feature: 2019062761: [*]:[c](:[*])O
10. Unknown ECFP_6 feature: 1307307440: [*]:[c](:[*])OC
11. Unknown ECFP_6 feature: 2011659532: [*]OC(C([*])[*])C([*])[*]
12. Unknown ECFP_6 feature: 305695353: [*]C([*])C(O)C([*])[*]
13. Unknown ECFP_6 feature: -553149446: [*]CC(O[*])C([*])[*]
14. Unknown ECFP_6 feature: -2060414325: [*]OC(O[*])C([*])[*]
15. Unknown ECFP_6 feature: -521596699: [*]C1[*][*]C([*])([*])C1O
16. Unknown ECFP_6 feature: 1953003528: [*]C[C@@]1(O)C[*][*]C1[*]
17. Unknown ECFP_6 feature: 414215731: [*]C1([*])[*][*]OC1
18. Unknown ECFP_6 feature: 1535429263: [*]OC(C)C([*])[*]
19. Unknown ECFP_6 feature: -1687549011: [*]OCC([*])[*]
20. Unknown ECFP_6 feature: 1129597062: [*]C([*])([*])CO

21. Unknown ECFP_6 feature: 865482986: [*]C([*])C
22. Unknown ECFP_6 feature: -1790412586: [*]CCO[*]
23. Unknown ECFP_6 feature: -1795525632: [*]CC[c](:[*]):[*]
24. Unknown ECFP_6 feature: -1684512787: [*]OC(=O)C=[*]
25. Unknown ECFP_6 feature: -470416293: [*]\C=C\C(=[*])[*]
26. Unknown ECFP_6 feature: -1831055759: [*]\C=C\[c](:[*]):[*]
27. Unknown ECFP_6 feature: -176483725: [*]=C[c](:[cH]:[*]):[cH]:[*]

Feature Contribution

Top features for positive contribution

Fingerprint	Bit/Smiles	Feature Structure	Score
FCFP_6	451847724	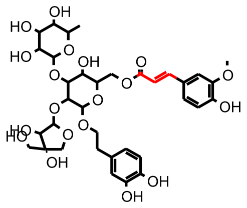 [*]\C=C\C(=[*])[*]	0.160
ECFP_6	-167460056	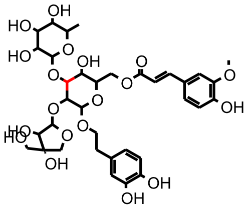 [*]C([*])[*]	0.136
FCFP_6	-1143715940	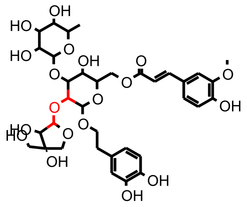 [*]C([*])OC([*])[*]	0.130

Top Features for negative contribution

Fingerprint	Bit/Smiles	Feature Structure	Score

FCFP_6	1	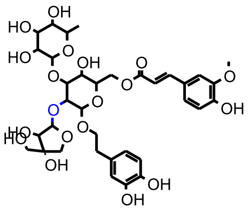<br>[*]O[*]	-0.102
FCFP_6	-453677277	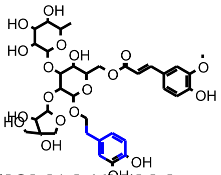<br>[*]C[c]1:[cH]:[*]:[c]([*]):[cH]:[cH]:1	-0.091
FCFP_6	136597326	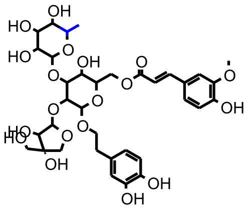<br>[*]C([*])C	-0.081

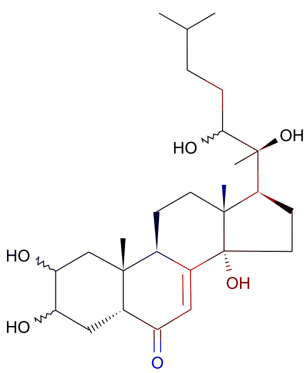

$C_{27}H_{44}O_6$

Molecular Weight: 464.63466

ALogP: 2.489

Rotatable Bonds: 5

Acceptors: 6

Donors: 5

Model Prediction

Prediction: 0.017

Unit: g/kg_body_weight

Mahalanobis Distance: 26.168

Mahalanobis Distance p-value: 1.14e-017

Mahalanobis Distance: The Mahalanobis distance (MD) is a generalization of the Euclidean distance that accounts for correlations among the X properties. It is calculated as the distance to the center of the training data. The larger the MD, the less trustworthy the prediction.
Mahalanobis Distance p-value: The p-value gives the fraction of training data with an MD greater than or equal to the one for the given sample, assuming normally distributed data. The smaller the p-value, the less trustworthy the prediciton. For highly non-normal X properties (e.g., fingerprints), the MD p-value is wildly inaccurate.

Structural Similar Compounds

Name	PRAVASTATIN	THIOPHANATEMETHYL	GLIPIZIDE
Structure	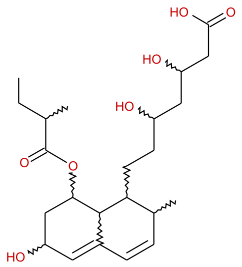	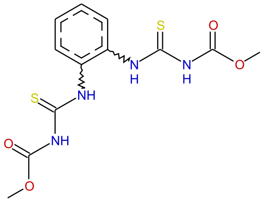	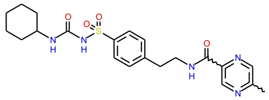
Actual Endpoint (-log C)	3.62791	4.02937	3.94991
Predicted Endpoint (-log C)	4.37763	3.44906	3.95594
Distance	0.688	0.886	0.889
Reference	NDA-19898	EPA COVER SHEET 0117;880301;(1)	NDA-17583

Model Applicability

Unknown features are fingerprint features in the query molecule, but not found in the training set.

1. OPS PC13 out of range. Value: -6.1434. Training min, max, SD, explained variance: -5.6594, 6.6668, 2.119, 0.0196.
2. Unknown ECFP_6 feature: 2018700401: [*]C([*])([*])O
3. Unknown ECFP_6 feature: 2025123907: [*]C([*])([*])O
4. Unknown ECFP_6 feature: 2024749573: [*]C([*])O
5. Unknown ECFP_6 feature: 2023785560: [*]C([*])O
6. Unknown ECFP_6 feature: 1908990050: [*]C[C@]1(C)[C@@H]([*])[*][*]C1([*])[*]
7. Unknown ECFP_6 feature: -483166673: [*]C(=[*])[C@]1(O)C[*][*]C1([*])[*]
8. Unknown ECFP_6 feature: -1049278438: [*]C([*])([*])[C@H]1C[*][*]C1([*])[*]
9. Unknown ECFP_6 feature: 333674688: [*]C[C@@H](C(=[*])[*])C([*])([*])[*]
10. Unknown ECFP_6 feature: -84975114: [*]CC(C)(C([*])[*])C([*])[*]
11. Unknown ECFP_6 feature: 412256466: [*]CCC([*])([*])[*]
12. Unknown ECFP_6 feature: 1908695621: [*]\C=C(/C([*])[*])\C([*])([*])[*]
13. Unknown ECFP_6 feature: 1195714366: [*]C([*])C(C)(O)C([*])[*]
14. Unknown ECFP_6 feature: 859433814: [*]C([*])([*])C
15. Unknown ECFP_6 feature: 413587124: [*]C([*])CC([*])([*])[*]
16. Unknown ECFP_6 feature: 464808839: [*]C(=CC(=[*])[*])[*]
17. Unknown ECFP_6 feature: -801490360: [*]C([*])CC([*])[*]
18. Unknown ECFP_6 feature: -211745668: [*]C([*])C(=O)C=[*]
19. Unknown ECFP_6 feature: -329826665: [*]CC(O)C([*])[*]

20. Unknown ECFP_6 feature: 80071435: [*]CC(O)C([*])([*])[*]
21. Unknown ECFP_6 feature: 865857320: [*]C([*])([*])C
22. Unknown ECFP_6 feature: -1790802833: [*]CCC([*])[*]
23. Unknown ECFP_6 feature: 1035165602: [*]CC(C)C

Feature Contribution

Top features for positive contribution

Fingerprint	Bit/Smiles	Feature Structure	Score
FCFP_6	451847724	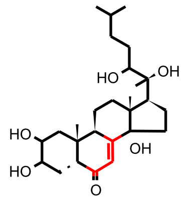 [*]\C=C\C(=[*])[*]	0.160
ECFP_6	-167460056	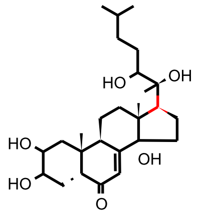 [*]C([*])[*]	0.136
ECFP_6	1559650422	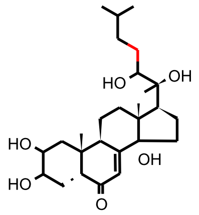 [*]C[*]	0.129

Top Features for negative contribution

Fingerprint	Bit/Smiles	Feature Structure	Score

ECFP_6	2106656448	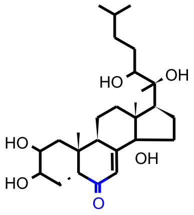 [*]C(=O)[*]	-0.110
FCFP_6	1	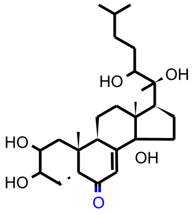 [*]O[*]	-0.102
FCFP_6	136597326	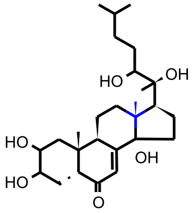 [*]C([*])C	-0.081

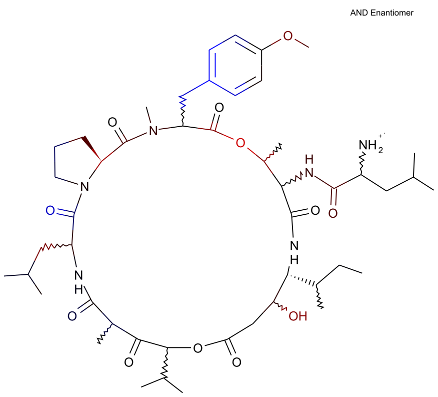

$C_{49}H_{79}N_6O_{12}$
Molecular Weight: 944.18455
ALogP: 3.176
Rotatable Bonds: 13
Acceptors: 12
Donors: 5

Structural Similar Compounds

Name	RESERPINE	FOSINOPRIL	DIARYLANILIDE YELLOW
Structure	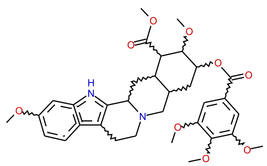	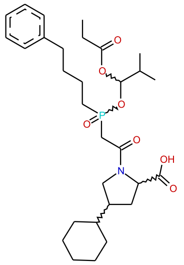	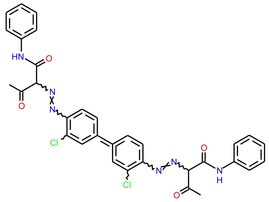
Actual Endpoint (-log C)	6.38645	3.14896	2.70208
Predicted Endpoint (-log C)	5.548	3.92187	3.76154
Distance	1.462	1.499	1.518
Reference	NTP 193 22	NDA-19915	NTP 30 C-4

Model Prediction

Prediction: 0.001
Unit: g/kg_body_weight
Mahalanobis Distance: 49.772
Mahalanobis Distance p-value: 2.56e-057

Mahalanobis Distance: The Mahalanobis distance (MD) is a generalization of the Euclidean distance that accounts for correlations among the X properties. It is calculated as the distance to the center of the training data. The larger the MD, the less trustworthy the prediction.
Mahalanobis Distance p-value: The p-value gives the fraction of training data with an MD greater than or equal to the one for the given sample, assuming normally distributed data. The smaller the p-value, the less trustworthy the prediciton. For highly non-normal X properties (e.g., fingerprints), the MD p-value is wildly inaccurate.

Model Applicability

Unknown features are fingerprint features in the query molecule, but not found in the training set.

1. Num_Atoms out of range. Value: 67. Training min, max, mean, SD: 3, 60, 15.593, 8.2.
2. Unknown FCFP_2 feature: 10: [*][NH2+][*]
3. Unknown FCFP_2 feature: 18028531: [*]C([*])[NH2+]C
4. Unknown FCFP_2 feature: -1817836174: [*]CC([NH2+][*])C(=[*])[*]
5. Unknown FCFP_2 feature: 136418580: [*][NH2+]C
6. Unknown ECFP_6 feature: -154530762: [*]N[*]
7. Unknown ECFP_6 feature: -591526139: [*][NH2+][*]
8. Unknown ECFP_6 feature: -555098937: [*]C([*])OC(=[*])[*]
9. Unknown ECFP_6 feature: 2024749573: [*]C([*])O
10. Unknown ECFP_6 feature: 1307307440: [*]:[c](:[*])OC
11. Unknown ECFP_6 feature: 1953224752: [*][C@@H]1[*][*]CN1C(=[*])[*]
12. Unknown ECFP_6 feature: 1617733200: [*]C([*])N(C)C(=[*])[*]
13. Unknown ECFP_6 feature: -1693599735: [*]C([*])NC(=[*])[*]
14. Unknown ECFP_6 feature: -2091181441: [*]C([*])NC(=[*])[*]
15. Unknown ECFP_6 feature: 1352327988: [*]C([*])[NH2+]C
16. Unknown ECFP_6 feature: -484970154: [*]N1[*][*]C[C@H]1C(=[*])[*]
17. Unknown ECFP_6 feature: -1332781180: [*]1[*]CCC1
18. Unknown ECFP_6 feature: -757679000: [*]N1[*][*]CC1
19. Unknown ECFP_6 feature: 1526862590: [*]C([*])C(=O)N([*])[*]
20. Unknown ECFP_6 feature: -410102202: [*]CC(N[*])C(=[*])[*]
21. Unknown ECFP_6 feature: -84540346: [*]C([*])CC([*])[*]

22. Unknown ECFP_6 feature: -1224874527: [*]CC(N([*])[*])C(=[*])[*]
23. Unknown ECFP_6 feature: 1035165602: [*]CC(C)C
24. Unknown ECFP_6 feature: 1691770380: [*]NC(C([*])[*])C([*])[*]
25. Unknown ECFP_6 feature: 1038798692: [*]CC(C)C([*])[*]
26. Unknown ECFP_6 feature: -329826665: [*]CC(O)C([*])[*]
27. Unknown ECFP_6 feature: 771121623: [*]C([*])C[c](:[*]):[*]
28. Unknown ECFP_6 feature: 866343404: [*]N([*])C
29. Unknown ECFP_6 feature: -1567199489: [*]NC(C([*])[*])C(=[*])[*]
30. Unknown ECFP_6 feature: -1886208901: [*]OC(=O)C([*])[*]
31. Unknown ECFP_6 feature: -949992060: [*]C([*])CC
32. Unknown ECFP_6 feature: -867777309: [*]NC(=O)C([*])[*]
33. Unknown ECFP_6 feature: 1535429263: [*]OC(C)C([*])[*]
34. Unknown ECFP_6 feature: -714938792: [*]C(=[*])C(C)C(=[*])[*]
35. Unknown ECFP_6 feature: 53207596: [*]C([*])CC(=[*])[*]
36. Unknown ECFP_6 feature: -855787384: [*]OC(C([*])[*])C(=[*])[*]
37. Unknown ECFP_6 feature: -212107780: [*]C([*])C(=O)C([*])[*]
38. Unknown ECFP_6 feature: -1167589895: [*]C([*])C(C)C
39. Unknown ECFP_6 feature: -1887539559: [*]CC(=O)O[*]
40. Unknown ECFP_6 feature: 865482986: [*]C([*])C
41. Unknown ECFP_6 feature: 20550775: [*]CC([NH2+][*])C(=[*])[*]
42. Unknown ECFP_6 feature: -649348348: [*]NC(=O)C([*])[*]
43. Unknown ECFP_6 feature: -1312559847: [*]C([*])CC([*])[*]
44. Unknown ECFP_6 feature: 866401773: [*][NH2+]C

Feature Contribution

Top features for positive contribution			
**Fingerprint**	**Bit/Smiles**	**Feature Structure**	**Score**
ECFP_6	-167460056	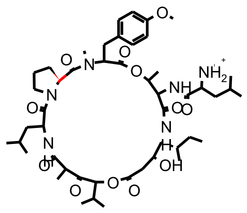 [*]C([*])[*]	0.136

FCFP_6	-1143715940	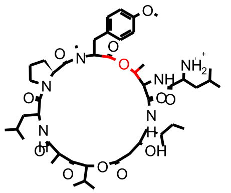<br>[*]C([*])OC([*])[*]	0.130
ECFP_6	1559650422	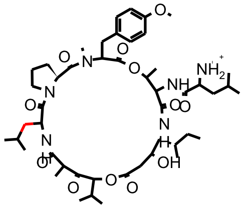<br>[*]C[*]	0.129
**Top Features for negative contribution**			
**Fingerprint**	**Bit/Smiles**	**Feature Structure**	**Score**
ECFP_6	2106656448	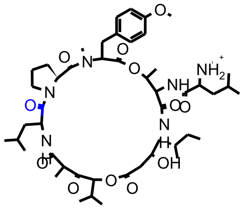<br>[*]C(=O)[*]	-0.110
FCFP_6	1	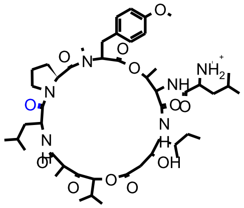<br>[*]O[*]	-0.102
FCFP_6	-453677277	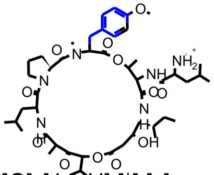<br>[*]C[c]1:[cH]:[*]:[c]([*]):[cH]:[cH]:1	-0.091

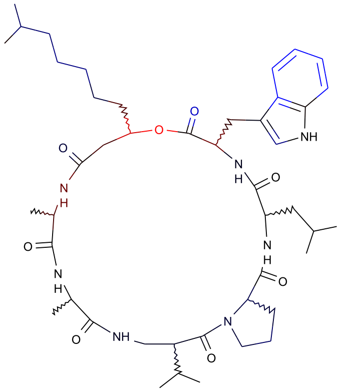

$C_{45}H_{69}N_7O_8$
Molecular Weight: 836.07145
ALogP: 4.986
Rotatable Bonds: 11
Acceptors: 8
Donors: 6

Model Prediction

Prediction: 0.012
Unit: g/kg_body_weight
Mahalanobis Distance: 49.702
Mahalanobis Distance p-value: 3.22e-057

Mahalanobis Distance: The Mahalanobis distance (MD) is a generalization of the Euclidean distance that accounts for correlations among the X properties. It is calculated as the distance to the center of the training data. The larger the MD, the less trustworthy the prediction.
Mahalanobis Distance p-value: The p-value gives the fraction of training data with an MD greater than or equal to the one for the given sample, assuming normally distributed data. The smaller the p-value, the less trustworthy the prediciton. For highly non-normal X properties (e.g., fingerprints), the MD p-value is wildly inaccurate.

Structural Similar Compounds

Name	DIARYLANILIDE YELLOW	RESERPINE	GLYBURIDE
Structure	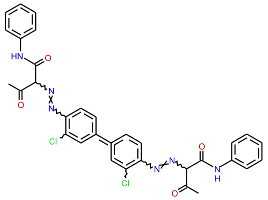	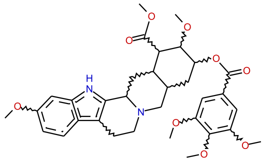	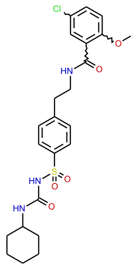
Actual Endpoint (-log C)	2.70208	6.38645	4.21661
Predicted Endpoint (-log C)	3.76154	5.548	4.21035
Distance	1.254	1.309	1.337
Reference	NTP 30 C-4	NTP 193 22	UPJ-26452

Model Applicability

Unknown features are fingerprint features in the query molecule, but not found in the training set.

1. All properties and OPS components are within expected ranges.
2. Unknown ECFP_6 feature: -152683720: [*]:[nH]:[*]
3. Unknown ECFP_6 feature: -154530762: [*]N[*]
4. Unknown ECFP_6 feature: -1020449580: [*][c]1:[*]:[*]:[nH]:[cH]:1
5. Unknown ECFP_6 feature: -2024509555: [*]C[c]1:[cH]:[*]:[*]:[c]:1:[*]
6. Unknown ECFP_6 feature: 1333660716: [*][c]1:[*]:[*]:[c](:[*]):[c]:1:[cH]:[*]
7. Unknown ECFP_6 feature: 1099224616: [*]:[cH]:[c]1:[nH]:[*]:[*]:[c]:1:[*]
8. Unknown ECFP_6 feature: -953984246: [*]:[c]1:[*]:[*]:[cH]:[nH]:1
9. Unknown ECFP_6 feature: 1997021792: [*]:[cH]:[cH]:[cH]:[*]
10. Unknown ECFP_6 feature: 771121623: [*]C([*])C[c](:[*]):[*]
11. Unknown ECFP_6 feature: -410102202: [*]CC(N[*])C(=[*])[*]
12. Unknown ECFP_6 feature: -1886208901: [*]OC(=O)C([*])[*]
13. Unknown ECFP_6 feature: -555098937: [*]C([*])OC(=[*])[*]
14. Unknown ECFP_6 feature: -554480104: [*]CC(C[*])O[*]
15. Unknown ECFP_6 feature: -1789838820: [*]CCC([*])[*]
16. Unknown ECFP_6 feature: -1790802833: [*]CCC([*])[*]
17. Unknown ECFP_6 feature: 1035165602: [*]CC(C)C
18. Unknown ECFP_6 feature: 53207596: [*]C([*])CC(=[*])[*]
19. Unknown ECFP_6 feature: -742538367: [*]CC(=O)N[*]
20. Unknown ECFP_6 feature: -1693599735: [*]C([*])NC(=[*])[*]
21. Unknown ECFP_6 feature: 1863041499: [*]NC(C)C(=[*])[*]

22. Unknown ECFP_6 feature: 865482986: [*]C([*])C
23. Unknown ECFP_6 feature: -867777309: [*]NC(=O)C([*])[*]
24. Unknown ECFP_6 feature: -1694930393: [*]CNC(=[*])[*]
25. Unknown ECFP_6 feature: -1457159889: [*]NCC([*])[*]
26. Unknown ECFP_6 feature: -2096927833: [*]CC(C([*])[*])C(=[*])[*]
27. Unknown ECFP_6 feature: -1167589895: [*]C([*])C(C)C
28. Unknown ECFP_6 feature: 1526862590: [*]C([*])C(=O)N([*])[*]
29. Unknown ECFP_6 feature: 1953224752: [*][C@@H]1[*][*]CN1C(=[*])[*]
30. Unknown ECFP_6 feature: -484970154: [*]N1[*][*]C[C@H]1C(=[*])[*]
31. Unknown ECFP_6 feature: -1332781180: [*]1[*]CCC1
32. Unknown ECFP_6 feature: -757679000: [*]N1[*][*]CC1
33. Unknown ECFP_6 feature: -84540346: [*]C([*])CC([*])[*]

Feature Contribution

Top features for positive contribution

Fingerprint	Bit/Smiles	Feature Structure	Score
ECFP_6	-167460056	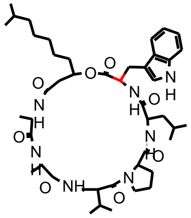 [*]C([*])[*]	0.136
FCFP_6	-1143715940	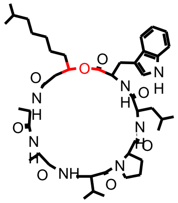 [*]C([*])OC([*])[*]	0.130
ECFP_6	1559650422	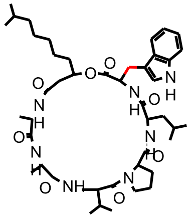 [*]C[*]	0.129

Top Features for negative contribution

Fingerprint	Bit/Smiles	Feature Structure	Score
FCFP_6	991735244	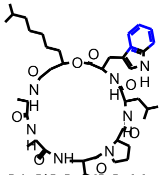 [*]:[c]1:[*]:[cH]:[cH]:[cH]:[cH]:1	-0.134
ECFP_6	1564392544	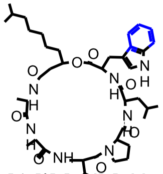 [*]:[c]1:[*]:[cH]:[cH]:[cH]:[cH]:1	-0.133
ECFP_6	2106656448	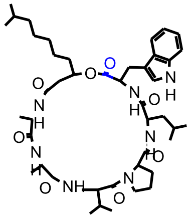 [*]C(=O)[*]	-0.110

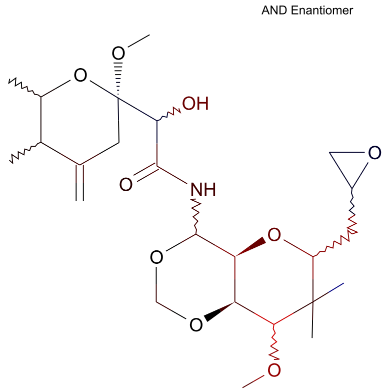

$C_{24}H_{39}NO_9$
Molecular Weight: 485.56776
ALogP: 0.267
Rotatable Bonds: 7
Acceptors: 9
Donors: 2

Model Prediction

Prediction: 0.001
Unit: g/kg_body_weight
Mahalanobis Distance: 41.299
Mahalanobis Distance p-value: 2.69e-044

Mahalanobis Distance: The Mahalanobis distance (MD) is a generalization of the Euclidean distance that accounts for correlations among the X properties. It is calculated as the distance to the center of the training data. The larger the MD, the less trustworthy the prediction.
Mahalanobis Distance p-value: The p-value gives the fraction of training data with an MD greater than or equal to the one for the given sample, assuming normally distributed data. The smaller the p-value, the less trustworthy the prediciton. For highly non-normal X properties (e.g., fingerprints), the MD p-value is wildly inaccurate.

Structural Similar Compounds

Name	LASIOCARPINE	PENICILLIN VK	PRAVASTATIN
Structure	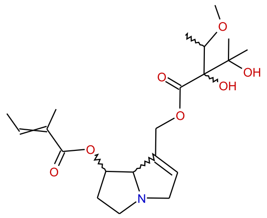	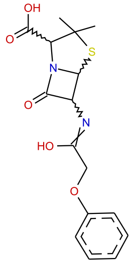	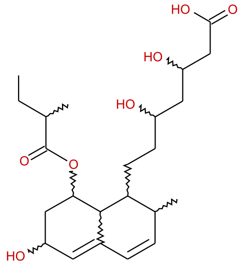
Actual Endpoint (-log C)	6.0703	2.99188	3.62791
Predicted Endpoint (-log C)	5.55369	4.18433	4.37763
Distance	0.615	0.785	0.829
Reference	NTP 39 34	NTP REPORT # 336	NDA-19898

Model Applicability

Unknown features are fingerprint features in the query molecule, but not found in the training set.

1. All properties and OPS components are within expected ranges.
2. Unknown FCFP_2 feature: 1327594201: [*]CC(O[*])(O[*])C([*])[*]
3. Unknown ECFP_6 feature: 1544874086: [*]=C
4. Unknown ECFP_6 feature: -1409796893: [*]C([*])OC([*])[*]
5. Unknown ECFP_6 feature: -1939757055: [*]C1[*][*]CO1
6. Unknown ECFP_6 feature: -409209140: [*]C([*])OC
7. Unknown ECFP_6 feature: -194719409: [*]C([*])OC([*])([*])[*]
8. Unknown ECFP_6 feature: 1667686968: [*]C([*])([*])OC
9. Unknown ECFP_6 feature: 2023785560: [*]C([*])O
10. Unknown ECFP_6 feature: -2091181441: [*]C([*])NC(=[*])[*]
11. Unknown ECFP_6 feature: 1067196438: [*]C([*])C(C)(C)C([*])[*]
12. Unknown ECFP_6 feature: -2123665288: [*]OC(C([*])[*])C([*])([*])[*]
13. Unknown ECFP_6 feature: -48623642: [*]CC(O[*])C([*])([*])[*]
14. Unknown ECFP_6 feature: -456441647: [*]OC(C([*])[*])C([*])[*]
15. Unknown ECFP_6 feature: -83576333: [*]C([*])CC1[*][*]1
16. Unknown ECFP_6 feature: -457637478: [*]NC(O[*])C([*])[*]
17. Unknown ECFP_6 feature: 859433814: [*]C([*])([*])C
18. Unknown ECFP_6 feature: -554480104: [*]CC(C[*])O[*]
19. Unknown ECFP_6 feature: -89681466: [*]OCO[*]
20. Unknown ECFP_6 feature: 837844420: [*]CC(O[*])(O[*])C([*])[*]
21. Unknown ECFP_6 feature: 825070174: [*]C(=[*])C(O)C([*])([*])[*]

22. Unknown ECFP_6 feature: -90310073: [*]C1CO1
23. Unknown ECFP_6 feature: -649348348: [*]NC(=O)C([*])[*]
24. Unknown ECFP_6 feature: 407900312: [*]C(=[*])CC([*])([*])[*]
25. Unknown ECFP_6 feature: 1863511924: [*]C([*])C(C)C(=[*])[*]
26. Unknown ECFP_6 feature: 1535429263: [*]OC(C)C([*])[*]
27. Unknown ECFP_6 feature: 1377749300: [*]CC(=C)C([*])[*]
28. Unknown ECFP_6 feature: 865482986: [*]C([*])C
29. Unknown ECFP_6 feature: -2085781978: [*]C(=C)[*]

Feature Contribution

Top features for positive contribution

Fingerprint	Bit/Smiles	Feature Structure	Score
ECFP_6	-167460056	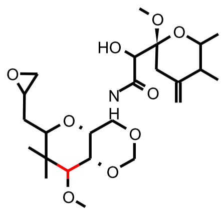 [*]C([*])[*]	0.136
FCFP_6	-1143715940	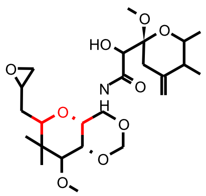 [*]C([*])OC([*])[*]	0.130
ECFP_6	1559650422	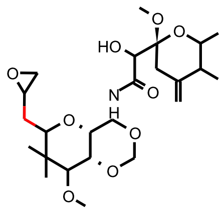 [*]C[*]	0.129

Top Features for negative contribution

Fingerprint	Bit/Smiles	Feature Structure	Score

FCFP_6	1	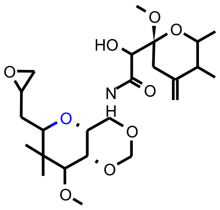 [*]O[*]	-0.102
FCFP_6	136597326	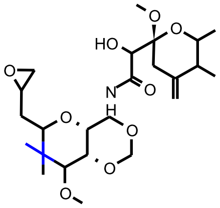 [*]C([*])C	-0.081
FCFP_6	566058135	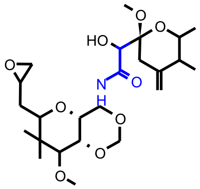 [*]NC(=O)C([*])[*]	-0.056

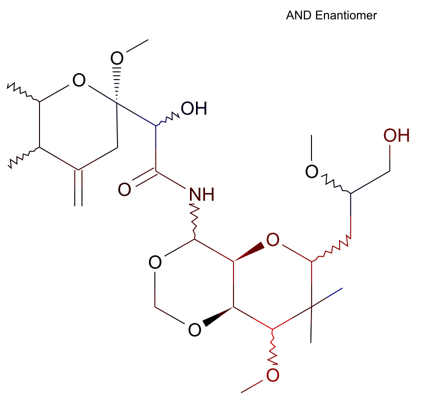

$C_{25}H_{43}NO_{10}$
Molecular Weight: 517.60962
ALogP: -9.1e-002
Rotatable Bonds: 9
Acceptors: 10
Donors: 3

Model Prediction

Prediction: 0.001
Unit: g/kg_body_weight
Mahalanobis Distance: 40.307
Mahalanobis Distance p-value: 1.13e-042

Mahalanobis Distance: The Mahalanobis distance (MD) is a generalization of the Euclidean distance that accounts for correlations among the X properties. It is calculated as the distance to the center of the training data. The larger the MD, the less trustworthy the prediction.
Mahalanobis Distance p-value: The p-value gives the fraction of training data with an MD greater than or equal to the one for the given sample, assuming normally distributed data. The smaller the p-value, the less trustworthy the prediciton. For highly non-normal X properties (e.g., fingerprints), the MD p-value is wildly inaccurate.

Structural Similar Compounds

Name	LASIOCARPINE	PRAVASTATIN	PENICILLIN VK
Structure	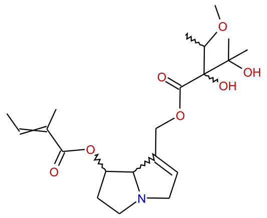	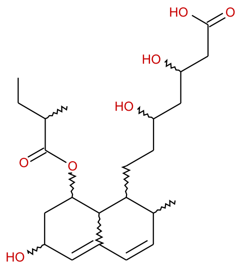	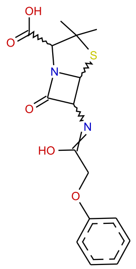
Actual Endpoint (-log C)	6.0703	3.62791	2.99188
Predicted Endpoint (-log C)	5.55369	4.37763	4.18433
Distance	0.695	0.769	0.901
Reference	NTP 39 34	NDA-19898	NTP REPORT # 336

Model Applicability

Unknown features are fingerprint features in the query molecule, but not found in the training set.

1. OPS PC13 out of range. Value: -5.9064. Training min, max, SD, explained variance: -5.6594, 6.6668, 2.119, 0.0196.
2. Unknown FCFP_2 feature: 1327594201: [*]CC(O[*])(O[*])C([*])[*]
3. Unknown ECFP_6 feature: 1544874086: [*]=C
4. Unknown ECFP_6 feature: -1409796893: [*]C([*])OC([*])[*]
5. Unknown ECFP_6 feature: -1939757055: [*]C1[*][*]CO1
6. Unknown ECFP_6 feature: -409209140: [*]C([*])OC
7. Unknown ECFP_6 feature: -410173153: [*]C([*])OC
8. Unknown ECFP_6 feature: -194719409: [*]C([*])OC([*])([*])[*]
9. Unknown ECFP_6 feature: 1667686968: [*]C([*])([*])OC
10. Unknown ECFP_6 feature: 2022454958: [*]CO
11. Unknown ECFP_6 feature: 2023785560: [*]C([*])O
12. Unknown ECFP_6 feature: -2091181441: [*]C([*])NC(=[*])[*]
13. Unknown ECFP_6 feature: 1067196438: [*]C([*])C(C)(C)C([*])[*]
14. Unknown ECFP_6 feature: -2123665288: [*]OC(C([*])[*])C([*])([*])[*]
15. Unknown ECFP_6 feature: -48623642: [*]CC(O[*])C([*])([*])[*]
16. Unknown ECFP_6 feature: -456441647: [*]OC(C([*])[*])C([*])[*]
17. Unknown ECFP_6 feature: -84540346: [*]C([*])CC([*])[*]
18. Unknown ECFP_6 feature: -457637478: [*]NC(O[*])C([*])[*]
19. Unknown ECFP_6 feature: 859433814: [*]C([*])([*])C
20. Unknown ECFP_6 feature: 197414153: [*]CC(C[*])O[*]

21. Unknown ECFP_6 feature: -89681466: [*]OCO[*]
22. Unknown ECFP_6 feature: -1909093651: [*]C([*])CO
23. Unknown ECFP_6 feature: 837844420: [*]CC(O[*])(O[*])C([*])[*]
24. Unknown ECFP_6 feature: -649348348: [*]NC(=O)C([*])[*]
25. Unknown ECFP_6 feature: 825070174: [*]C(=[*])C(O)C([*])([*])[*]
26. Unknown ECFP_6 feature: 407900312: [*]C(=[*])CC([*])([*])[*]
27. Unknown ECFP_6 feature: 1863511924: [*]C([*])C(C)C(=[*])[*]
28. Unknown ECFP_6 feature: 1535429263: [*]OC(C)C([*])[*]
29. Unknown ECFP_6 feature: 1377749300: [*]CC(=C)C([*])[*]
30. Unknown ECFP_6 feature: 865482986: [*]C([*])C
31. Unknown ECFP_6 feature: -2085781978: [*]C(=C)[*]

Feature Contribution

Top features for positive contribution			
**Fingerprint**	**Bit/Smiles**	**Feature Structure**	**Score**
ECFP_6	-167460056	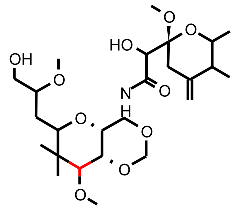 [*]C([*])[*]	0.136
FCFP_6	-1143715940	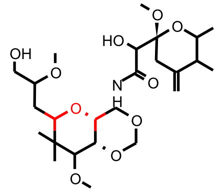 [*]C([*])OC([*])[*]	0.130
ECFP_6	1559650422	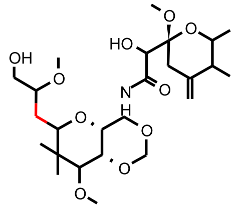 [*]C[*]	0.129

Top Features for negative contribution			
**Fingerprint**	**Bit/Smiles**	**Feature Structure**	**Score**

FCFP_6	1	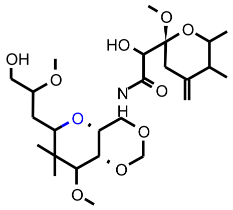 [*]O[*]	-0.102
FCFP_6	136597326	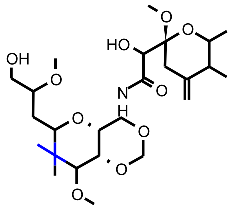 [*]C([*])C	-0.081
FCFP_6	566058135	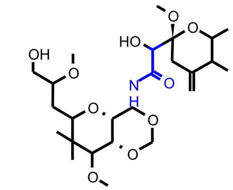 [*]NC(=O)C([*])[*]	-0.056

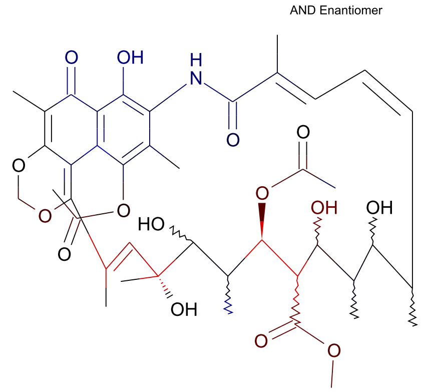

$C_{42}H_{53}NO_{15}$
Molecular Weight: 811.86791
ALogP: 2.745
Rotatable Bonds: 6
Acceptors: 15
Donors: 6

Model Prediction

Prediction: 0.001
Unit: g/kg_body_weight
Mahalanobis Distance: 47.019
Mahalanobis Distance p-value: 2.98e-053

Mahalanobis Distance: The Mahalanobis distance (MD) is a generalization of the Euclidean distance that accounts for correlations among the X properties. It is calculated as the distance to the center of the training data. The larger the MD, the less trustworthy the prediction.
Mahalanobis Distance p-value: The p-value gives the fraction of training data with an MD greater than or equal to the one for the given sample, assuming normally distributed data. The smaller the p-value, the less trustworthy the prediciton. For highly non-normal X properties (e.g., fingerprints), the MD p-value is wildly inaccurate.

Structural Similar Compounds

Name	CARMINE	OXYTETRACYCLINE .HCL	TETRACYCLINE .HCL
Structure	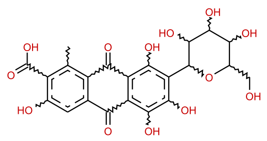	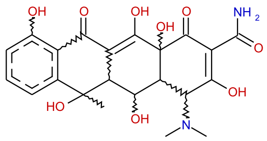	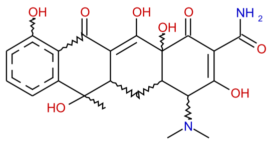
Actual Endpoint (-log C)	3.5162	2.56626	2.85193
Predicted Endpoint (-log C)	3.66829	3.75581	3.94748
Distance	1.216	1.251	1.288
Reference	FOOD.CHEM.TOXICOL.25.897.1987	NTP REPORT # 315	NTP REPORT # 344

Model Applicability

Unknown features are fingerprint features in the query molecule, but not found in the training set.

1. OPS PC13 out of range. Value: -5.7077. Training min, max, SD, explained variance: -5.6594, 6.6668, 2.119, 0.0196.
2. Unknown ECFP_6 feature: -154530762: [*]N[*]
3. Unknown ECFP_6 feature: 456978524: [*]C([*])OC(=[*])[*]
4. Unknown ECFP_6 feature: 2024749573: [*]C([*])O
5. Unknown ECFP_6 feature: 2018700401: [*]C([*])([*])O
6. Unknown ECFP_6 feature: -408473190: [*]C(=[*])OC
7. Unknown ECFP_6 feature: -556429595: [*]COC(=[*])[*]
8. Unknown ECFP_6 feature: 1311676480: [*]C(=[*])O[c](:[*]):[*]
9. Unknown ECFP_6 feature: 2019062761: [*]:[c](:[*])O
10. Unknown ECFP_6 feature: -1699286547: [*]C(=[*])N[c](:[*]):[*]
11. Unknown ECFP_6 feature: 308335392: [*]C([*])C(C([*])[*])C(=[*])[*]
12. Unknown ECFP_6 feature: 2011659532: [*]OC(C([*])[*])C([*])[*]
13. Unknown ECFP_6 feature: 824248976: [*]C([*])C(C)C([*])[*]
14. Unknown ECFP_6 feature: 305695353: [*]C([*])C(O)C([*])[*]
15. Unknown ECFP_6 feature: -521596699: [*]C1[*][*]C([*])([*])C1O
16. Unknown ECFP_6 feature: -40769921: [*]C([*])[C@](C)(O)C=[*]
17. Unknown ECFP_6 feature: 865482986: [*]C([*])C
18. Unknown ECFP_6 feature: 824611088: [*]C([*])C(C)C=[*]
19. Unknown ECFP_6 feature: -1683911134: [*]OC(=O)C([*])[*]

20. Unknown ECFP_6 feature: 859433814: [*]C([*])([*])C
21. Unknown ECFP_6 feature: 464446479: [*]C(=CC([*])([*])[*])[*]
22. Unknown ECFP_6 feature: 890368401: [*]\C=C/C([*])[*]
23. Unknown ECFP_6 feature: -3067141: [*]\C=C(/C)\C(=[*])[*]
24. Unknown ECFP_6 feature: -473922720: [*]OC(=O)C
25. Unknown ECFP_6 feature: 1796421070: [*]OC(=C([*])[*])C(=[*])[*]
26. Unknown ECFP_6 feature: 300955665: [*]\C=C/C=[*]
27. Unknown ECFP_6 feature: 1790105651: [*]C(=C(C(=[*])[*])[c](:[*]):[*])[*]
28. Unknown ECFP_6 feature: -813997308: [*]C(=[*])[c](:[c]([*]):[*]):[c]([*]):[*]
29. Unknown ECFP_6 feature: 470857763: [*]C(=CC=[*])[*]
30. Unknown ECFP_6 feature: 1792159373: [*]C(=C(C)C(=[*])[*])[*]
31. Unknown ECFP_6 feature: -89681466: [*]OCO[*]
32. Unknown ECFP_6 feature: -1531301414: [*]O[c](:[c]([*]):[*]):[c]([*]):[*]
33. Unknown ECFP_6 feature: 1717462980: [*]C(=[*])C(=O)[c](:[*]):[*]
34. Unknown ECFP_6 feature: -1660913849: [*][c](:[*]):[c](O):[c]([*]):[*]
35. Unknown ECFP_6 feature: 1576255326: [*][c](:[*]):[c](C):[c]([*]):[*]
36. Unknown ECFP_6 feature: -1659480185: [*]N[c](:[c]([*]):[*]):[c]([*]):[*]
37. Unknown ECFP_6 feature: 1298725959: [*]NC(=O)C(=[*])[*]

Feature Contribution

Top features for positive contribution

Fingerprint	Bit/Smiles	Feature Structure	Score
FCFP_6	451847724	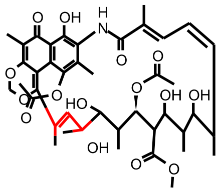 [*]\C=C\C(=[*])[*]	0.160
ECFP_6	-167460056	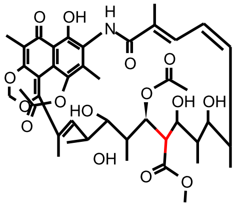 [*]C([*])[*]	0.136

FCFP_6	-1143715940	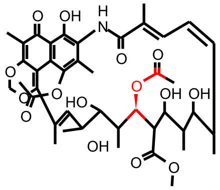 [*]C([*])OC([*])[*]	0.130
**Top Features for negative contribution**			
**Fingerprint**	**Bit/Smiles**	**Feature Structure**	**Score**
ECFP_6	2106656448	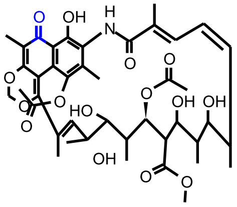 [*]C(=O)[*]	-0.110
FCFP_6	1	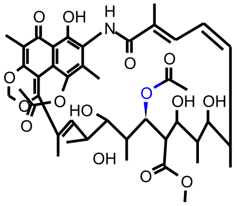 [*]O[*]	-0.102
FCFP_6	136597326	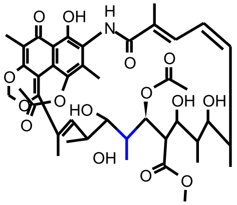 [*]C([*])C	-0.081

Simeprevir

TOPKAT_Chronic_LOAEL

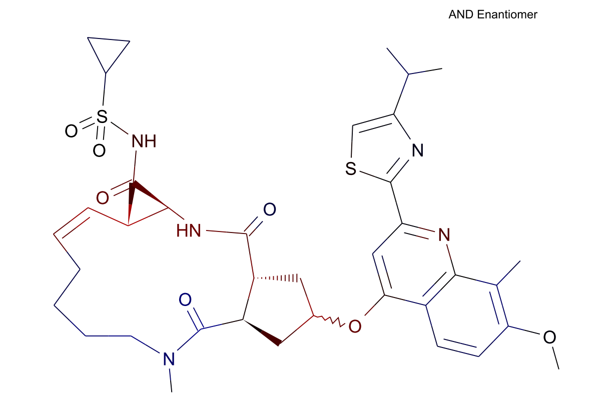

$C_{38}H_{47}N_5O_7S_2$
Molecular Weight: 749.93907
ALogP: 4.796
Rotatable Bonds: 8
Acceptors: 9
Donors: 2

Structural Similar Compounds

Name	RESERPINE	DIARYLANILIDE YELLOW	C.I.PIGMENT RED 23
Structure	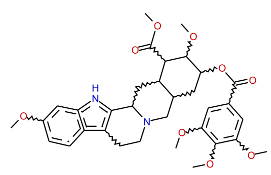	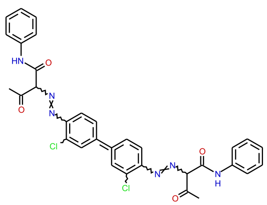	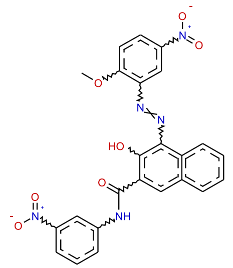
Actual Endpoint (-log C)	6.38645	2.70208	2.28997
Predicted Endpoint (-log C)	5.548	3.76154	3.52921
Distance	0.885	0.887	0.922
Reference	NTP 193 22	NTP 30 C-4	NTP 411 146

Model Prediction

Prediction: 0.002
Unit: g/kg_body_weight
Mahalanobis Distance: 55.670
Mahalanobis Distance p-value: 1.58e-065

Mahalanobis Distance: The Mahalanobis distance (MD) is a generalization of the Euclidean distance that accounts for correlations among the X properties. It is calculated as the distance to the center of the training data. The larger the MD, the less trustworthy the prediction.
Mahalanobis Distance p-value: The p-value gives the fraction of training data with an MD greater than or equal to the one for the given sample, assuming normally distributed data. The smaller the p-value, the less trustworthy the prediciton. For highly non-normal X properties (e.g., fingerprints), the MD p-value is wildly inaccurate.

Model Applicability

Unknown features are fingerprint features in the query molecule, but not found in the training set.

1. OPS PC1 out of range. Value: 18.64. Training min, max, SD, explained variance: -9.2986, 15.594, 5, 0.1094.
2. Unknown FCFP_2 feature: 1018942292: [*]NS(=O)(=O)C1[*][*]1
3. Unknown ECFP_6 feature: -154530762: [*]N[*]
4. Unknown ECFP_6 feature: 914325265: [*]:s:[*]
5. Unknown ECFP_6 feature: -797085356: [*]S(=[*])(=[*])[*]
6. Unknown ECFP_6 feature: -2095963820: [*][C@@H]1[*][*]C[C@H]1C(=[*])[*]
7. Unknown ECFP_6 feature: -867777309: [*]NC(=O)C([*])[*]
8. Unknown ECFP_6 feature: -1338907019: [*]C(=[*])NC1([*])[*][*]1
9. Unknown ECFP_6 feature: -309676563: [*]N[C@]1(C[C@H]1[*])C(=[*])[*]
10. Unknown ECFP_6 feature: 413587124: [*]C([*])CC([*])([*])[*]
11. Unknown ECFP_6 feature: -1049290660: [*]C1([*])C[C@H]1C=[*]
12. Unknown ECFP_6 feature: 890368401: [*]\C=C/C([*])[*]
13. Unknown ECFP_6 feature: 360408239: [*]C\C=C/[*]
14. Unknown ECFP_6 feature: -1331088410: [*]CCC=[*]
15. Unknown ECFP_6 feature: -1332781180: [*]1[*]CCC1
16. Unknown ECFP_6 feature: -757679000: [*]N1[*][*]CC1
17. Unknown ECFP_6 feature: 1616402542: [*]CN(C)C(=[*])[*]
18. Unknown ECFP_6 feature: 1526862590: [*]C([*])C(=O)N([*])[*]
19. Unknown ECFP_6 feature: -801490360: [*]C([*])CC([*])[*]
20. Unknown ECFP_6 feature: 1480368712: [*]OC1C[*][*]C1

21. Unknown ECFP_6 feature: -1818486371: [*]NC(=O)C1([*])[*][*]1
22. Unknown ECFP_6 feature: 946167604: [*]C(=[*])NS(=[*])(=[*])[*]
23. Unknown ECFP_6 feature: 866343404: [*]N([*])C
24. Unknown ECFP_6 feature: 1310940530: [*]C([*])O[c](:[*]):[*]
25. Unknown ECFP_6 feature: -428002189: [*]:[cH]:[c](:n:[*])[c](:[*]):[*]
26. Unknown ECFP_6 feature: 1333660716: [*][c]1:[*]:[*]:[c](:[*]):[c]:1:[cH]:[*]
27. Unknown ECFP_6 feature: 1576255326: [*][c](:[*]):[c](C):[c]([*]):[*]
28. Unknown ECFP_6 feature: 1048320787: [*][c](:[*]):[c](:n:[*]):[c](:[*]):[*]
29. Unknown ECFP_6 feature: -1426923364: [*][c]1:[*]:[*]:[cH]:s:1
30. Unknown ECFP_6 feature: -253227249: [*]:[c](:[*])[c]1:n:[*]:[*]:s:1
31. Unknown ECFP_6 feature: 1411720546: [*]C([*])[c]1:[cH]:[*]:[*]:n:1
32. Unknown ECFP_6 feature: -224638920: [*][c]1:[*]:[*]:s:[cH]:1
33. Unknown ECFP_6 feature: 733491677: [*]:[c](:[*])C(C)C
34. Unknown ECFP_6 feature: 1307307440: [*]:[c](:[*])OC
35. Unknown ECFP_6 feature: 2121941848: [*]NS(=O)(=O)C1[*][*]1
36. Unknown ECFP_6 feature: 2102150379: [*]S(=[*])(=O)[*]
37. Unknown ECFP_6 feature: -622223421: [*]S(=[*])(=[*])C1CC1

Feature Contribution

Top features for positive contribution			
**Fingerprint**	**Bit/Smiles**	**Feature Structure**	**Score**
FCFP_6	451847724	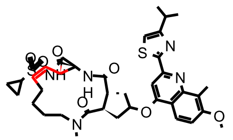 [*]\C=C\C(=[*])[*]	0.160
ECFP_6	-167460056	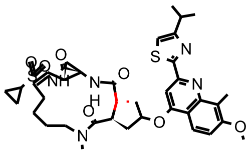 [*]C([*])[*]	0.136

FCFP_6	3	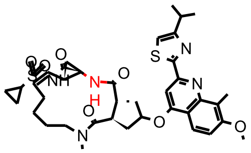 [*]O	0.092
**Top Features for negative contribution**			
**Fingerprint**	**Bit/Smiles**	**Feature Structure**	**Score**
ECFP_6	2106656448	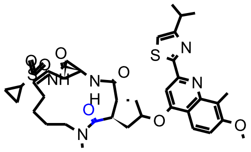 [*]C(=O)[*]	-0.110
FCFP_6	1	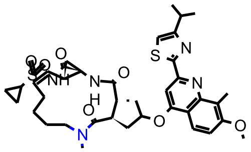 [*]O[*]	-0.102
FCFP_6	136597326	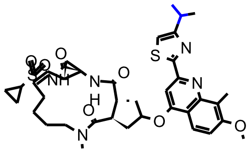 [*]C([*])C	-0.081

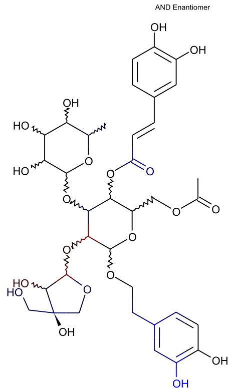

$C_{36}H_{46}O_{20}$
Molecular Weight: 798.73843
ALogP: -0.546
Rotatable Bonds: 16
Acceptors: 20
Donors: 10

Model Prediction

Prediction: 2.382
Unit: g/kg_body_weight
Mahalanobis Distance: 14.541
Mahalanobis Distance p-value: 1.01e-013

Mahalanobis Distance: The Mahalanobis distance (MD) is a generalization of the Euclidean distance that accounts for correlations among the X properties. It is calculated as the distance to the center of the training data. The larger the MD, the less trustworthy the prediction.
Mahalanobis Distance p-value: The p-value gives the fraction of training data with an MD greater than or equal to the one for the given sample, assuming normally distributed data. The smaller the p-value, the less trustworthy the prediciton. For highly non-normal X properties (e.g., fingerprints), the MD p-value is wildly inaccurate.

Structural Similar Compounds

Name	OXYTETRACYCLINE	ERYTHROMYCIN	4,4'-DIAMINO-2,2'-STILBENEDISULFONIC ACID.2NaSALT
Structure	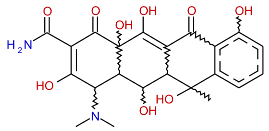	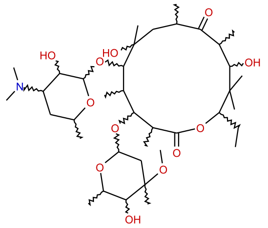	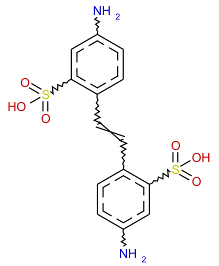
Actual Endpoint (-log C)	2.36214	3.29629	2.50759
Predicted Endpoint (-log C)	2.77834	4.83895	3.26068
Distance	1.763	1.855	2.106
Reference	NCI/NTP TR-315	NCI/NTP TR-338	NCI/NTP TR-412

Model Applicability

Unknown features are fingerprint features in the query molecule, but not found in the training set.

1. Molecular_Weight out of range. Value: 798.74. Training min, max, mean, SD: 74.122, 731.95, 245.25, 106.4.
2. Num_H_Donors out of range. Value: 10. Training min, max, mean, SD: 0, 7, 1.2135, 1.216.
3. Num_H_Acceptors out of range. Value: 20. Training min, max, mean, SD: 0, 13, 3.2809, 2.199.
4. Molecular_PolarSurfaceArea out of range. Value: 310.28. Training min, max, mean, SD: 0, 201.84, 63.052, 40.7.
5. Molecular_PolarSASA out of range. Value: 473.59. Training min, max, mean, SD: 0, 357.83, 109.38, 65.5.
6. OPS PC2 out of range. Value: 9.7706. Training min, max, SD, explained variance: -5.6103, 7.6016, 2.34, 0.1237.
7. OPS PC7 out of range. Value: 4.5303. Training min, max, SD, explained variance: -4.36, 3.9384, 1.418, 0.0454.
8. OPS PC13 out of range. Value: 3.5474. Training min, max, SD, explained variance: -2.9619, 2.8704, 1.049, 0.0249.
9. OPS PC14 out of range. Value: -2.3308. Training min, max, SD, explained variance: -2.0656, 3.3808, 1.011, 0.0231.

Feature Contribution

Top features for positive contribution

Fingerprint	Bit/Smiles	Feature Structure	Score
FCFP_2	-1143715940	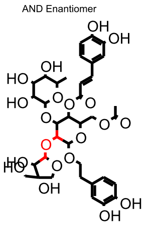 [*]C([*])OC([*])[*]	0.095
FCFP_2	3	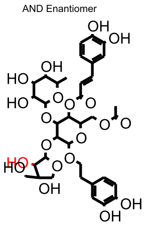 [*]O	0.074
FCFP_2	565998553	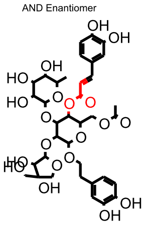 [*]OC(=O)C=[*]	0.008
**Top Features for negative contribution**			
**Fingerprint**	**Bit/Smiles**	**Feature Structure**	**Score**
FCFP_2	7	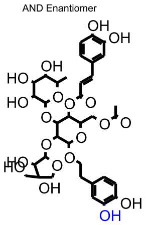 [*]O	-0.214

FCFP_2	-549108873	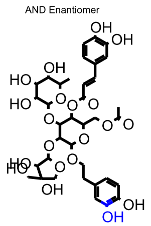 [*]:[c](:[*])O	-0.127
FCFP_2	1872154524	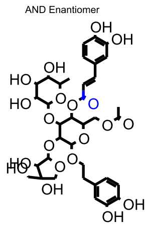 [*]C(=O)[*]	-0.105

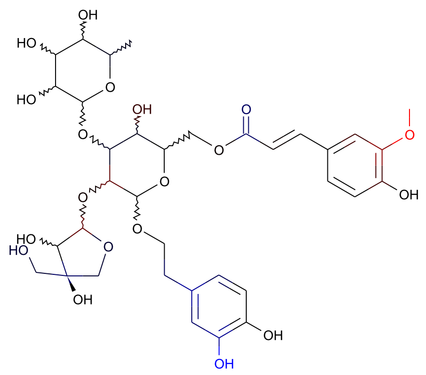

$C_{35}H_{46}O_{19}$
Molecular Weight: 770.72833
ALogP: -0.699
Rotatable Bonds: 15
Acceptors: 19
Donors: 10

Model Prediction

Prediction: 1.277
Unit: g/kg_body_weight
Mahalanobis Distance: 14.182
Mahalanobis Distance p-value: 5.56e-013

Mahalanobis Distance: The Mahalanobis distance (MD) is a generalization of the Euclidean distance that accounts for correlations among the X properties. It is calculated as the distance to the center of the training data. The larger the MD, the less trustworthy the prediction.
Mahalanobis Distance p-value: The p-value gives the fraction of training data with an MD greater than or equal to the one for the given sample, assuming normally distributed data. The smaller the p-value, the less trustworthy the prediciton. For highly non-normal X properties (e.g., fingerprints), the MD p-value is wildly inaccurate.

Structural Similar Compounds

Name	OXYTETRACYCLINE	ERYTHROMYCIN	4,4'-DIAMINO-2,2'-STILBENEDISULFONIC ACID.2NaSALT
Structure	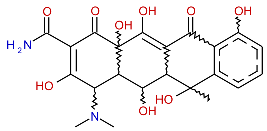	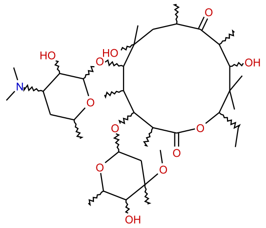	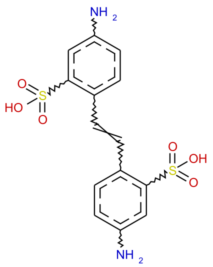
Actual Endpoint (-log C)	2.36214	3.29629	2.50759
Predicted Endpoint (-log C)	2.77834	4.83895	3.26068
Distance	1.648	1.749	2.004
Reference	NCI/NTP TR-315	NCI/NTP TR-338	NCI/NTP TR-412

Model Applicability

Unknown features are fingerprint features in the query molecule, but not found in the training set.

1. Molecular_Weight out of range. Value: 770.73. Training min, max, mean, SD: 74.122, 731.95, 245.25, 106.4.
2. Num_H_Donors out of range. Value: 10. Training min, max, mean, SD: 0, 7, 1.2135, 1.216.
3. Num_H_Acceptors out of range. Value: 19. Training min, max, mean, SD: 0, 13, 3.2809, 2.199.
4. Molecular_PolarSurfaceArea out of range. Value: 293.2. Training min, max, mean, SD: 0, 201.84, 63.052, 40.7.
5. Molecular_PolarSASA out of range. Value: 446.75. Training min, max, mean, SD: 0, 357.83, 109.38, 65.5.
6. OPS PC1 out of range. Value: 7.3866. Training min, max, SD, explained variance: -4.595, 7.2949, 2.487, 0.1398.
7. OPS PC2 out of range. Value: 9.5275. Training min, max, SD, explained variance: -5.6103, 7.6016, 2.34, 0.1237.
8. OPS PC7 out of range. Value: 4.5197. Training min, max, SD, explained variance: -4.36, 3.9384, 1.418, 0.0454.
9. OPS PC11 out of range. Value: 3.8793. Training min, max, SD, explained variance: -3.8346, 3.8752, 1.233, 0.0343.
10. OPS PC13 out of range. Value: 3.8687. Training min, max, SD, explained variance: -2.9619, 2.8704, 1.049, 0.0249.

Feature Contribution

Top features for positive contribution			
**Fingerprint**	**Bit/Smiles**	**Feature Structure**	**Score**
FCFP_2	136627117	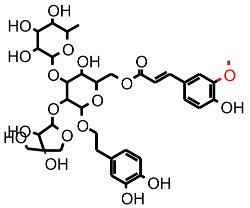 [*]OC	0.173
FCFP_2	-1143715940	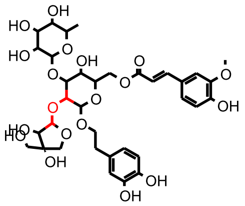 [*]C([*])OC([*])[*]	0.095
FCFP_2	1036089772	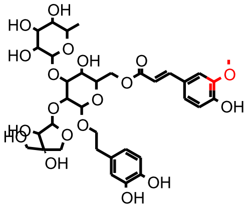 [*]:[c](:[*])OC	0.075

Top Features for negative contribution			
**Fingerprint**	**Bit/Smiles**	**Feature Structure**	**Score**
FCFP_2	7	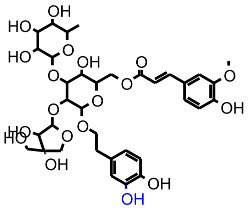 [*]O	-0.214

FCFP_2	-549108873	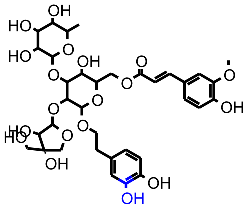 [*]:[c](:[*])O	-0.127
FCFP_2	1872154524	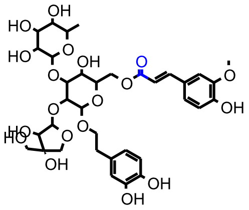 [*]C(=O)[*]	-0.105

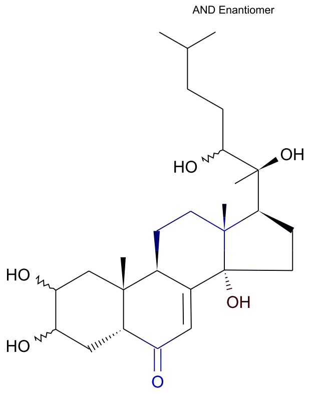

$C_{27}H_{44}O_6$
Molecular Weight: 464.63466
ALogP: 2.489
Rotatable Bonds: 5
Acceptors: 6
Donors: 5

Model Prediction

Prediction: 0.137
Unit: g/kg_body_weight
Mahalanobis Distance: 9.007
Mahalanobis Distance p-value: 0.00138

Mahalanobis Distance: The Mahalanobis distance (MD) is a generalization of the Euclidean distance that accounts for correlations among the X properties. It is calculated as the distance to the center of the training data. The larger the MD, the less trustworthy the prediction.
Mahalanobis Distance p-value: The p-value gives the fraction of training data with an MD greater than or equal to the one for the given sample, assuming normally distributed data. The smaller the p-value, the less trustworthy the prediciton. For highly non-normal X properties (e.g., fingerprints), the MD p-value is wildly inaccurate.

Structural Similar Compounds

Name	LASIOCARPINE	ACETOHEXAMIDE	HYDROCHLOROTHIAZIDE
Structure	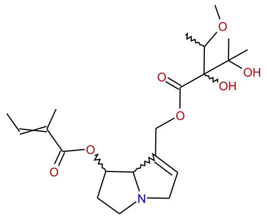	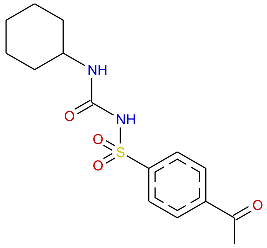	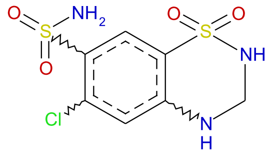
Actual Endpoint (-log C)	5.48403	2.55683	3.56001
Predicted Endpoint (-log C)	3.82301	3.62413	3.55045
Distance	0.872	0.922	0.974
Reference	NCI/NTP TR-039	NCI/NTP TR-050	NCI/NTP TR-357

Model Applicability

Unknown features are fingerprint features in the query molecule, but not found in the training set.

1. All properties and OPS components are within expected ranges.

Feature Contribution

Top features for positive contribution

Fingerprint	Bit/Smiles	Feature Structure	Score
FCFP_2	3	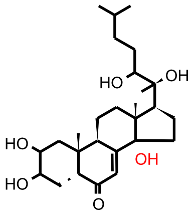 [*]O	0.074

Top Features for negative contribution

Fingerprint	Bit/Smiles	Feature Structure	Score

FCFP_2	-1272798659	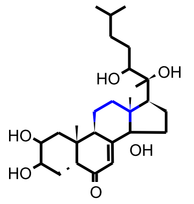 [*]CCC([*])([*])[*]	-0.111
FCFP_2	1872154524	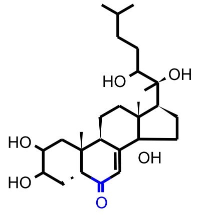 [*]C(=O)[*]	-0.105
FCFP_2	1	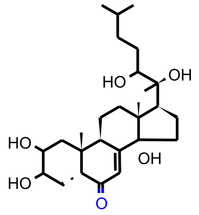 [*]O[*]	-0.080

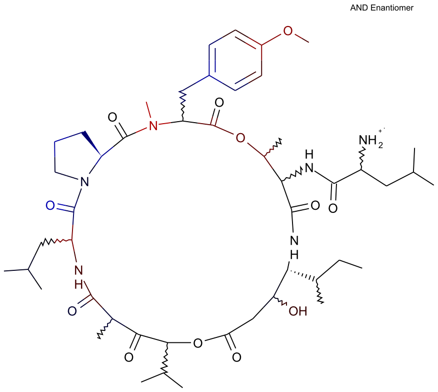

$C_{49}H_{79}N_6O_{12}$
Molecular Weight: 944.18455
ALogP: 3.176
Rotatable Bonds: 13
Acceptors: 12
Donors: 5

Model Prediction

Prediction: 0.002
Unit: g/kg_body_weight
Mahalanobis Distance: 15.135
Mahalanobis Distance p-value: 5.92e-015

Mahalanobis Distance: The Mahalanobis distance (MD) is a generalization of the Euclidean distance that accounts for correlations among the X properties. It is calculated as the distance to the center of the training data. The larger the MD, the less trustworthy the prediction.
Mahalanobis Distance p-value: The p-value gives the fraction of training data with an MD greater than or equal to the one for the given sample, assuming normally distributed data. The smaller the p-value, the less trustworthy the prediciton. For highly non-normal X properties (e.g., fingerprints), the MD p-value is wildly inaccurate.

Structural Similar Compounds

Name	ERYTHROMYCIN	RESERPINE	OXYTETRACYCLINE
Structure	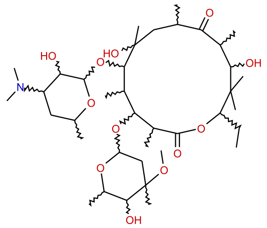	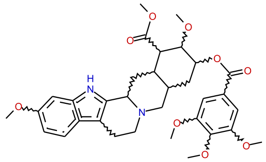	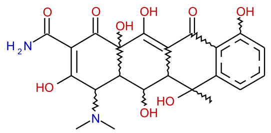
Actual Endpoint (-log C)	3.29629	6.13118	2.36214
Predicted Endpoint (-log C)	4.83895	4.38304	2.77834
Distance	0.971	1.511	1.517
Reference	NCI/NTP TR-338	NCI/NTP TR-193	NCI/NTP TR-315

Model Applicability

Unknown features are fingerprint features in the query molecule, but not found in the training set.

1. Molecular_Weight out of range. Value: 944.18. Training min, max, mean, SD: 74.122, 731.95, 245.25, 106.4.
2. Molecular_PolarSurfaceArea out of range. Value: 243.65. Training min, max, mean, SD: 0, 201.84, 63.052, 40.7.
3. OPS PC1 out of range. Value: 8.4984. Training min, max, SD, explained variance: -4.595, 7.2949, 2.487, 0.1398.
4. OPS PC9 out of range. Value: 5.4631. Training min, max, SD, explained variance: -2.8548, 3.3954, 1.263, 0.0360.
5. Unknown FCFP_2 feature: 10: [*][NH2+][*]
6. Unknown FCFP_2 feature: 18028531: [*]C([*])[NH2+]C
7. Unknown FCFP_2 feature: -1817836174: [*]CC([NH2+][*])C(=[*])[*]
8. Unknown FCFP_2 feature: 136418580: [*][NH2+]C

Feature Contribution

Top features for positive contribution

Fingerprint	Bit/Smiles	Feature Structure	Score

FCFP_2	136627117	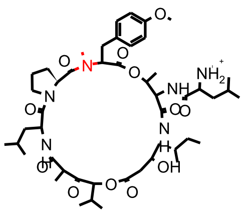 [*]OC	0.173
FCFP_2	-885550502	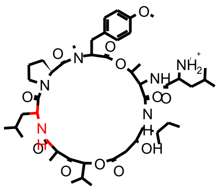 [*]C([*])NC(=[*])[*]	0.115
FCFP_2	-1143715940	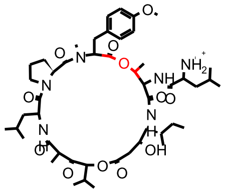 [*]C([*])OC([*])[*]	0.095
**Top Features for negative contribution**			
**Fingerprint**	**Bit/Smiles**	**Feature Structure**	**Score**
FCFP_2	-1272798659	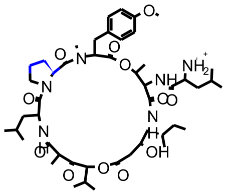 [*]CCC([*])([*])[*]	-0.111
FCFP_2	1872154524	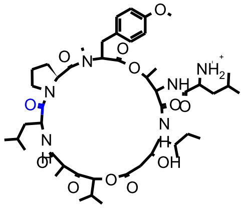 [*]C(=O)[*]	-0.105

FCFP_2	203677720	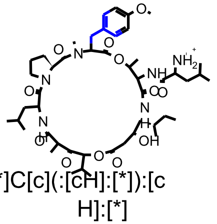<br>[*]C[c](:[cH]:[*]):[cH]:[*]	-0.083

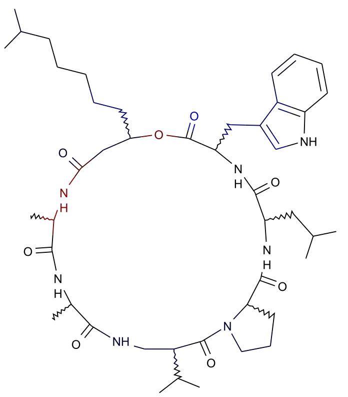

$C_{45}H_{69}N_7O_8$
Molecular Weight: 836.07145
ALogP: 4.986
Rotatable Bonds: 11
Acceptors: 8
Donors: 6

Model Prediction

Prediction: 0.021
Unit: g/kg_body_weight
Mahalanobis Distance: 15.238
Mahalanobis Distance p-value: 3.62e-015

Mahalanobis Distance: The Mahalanobis distance (MD) is a generalization of the Euclidean distance that accounts for correlations among the X properties. It is calculated as the distance to the center of the training data. The larger the MD, the less trustworthy the prediction.
Mahalanobis Distance p-value: The p-value gives the fraction of training data with an MD greater than or equal to the one for the given sample, assuming normally distributed data. The smaller the p-value, the less trustworthy the prediciton. For highly non-normal X properties (e.g., fingerprints), the MD p-value is wildly inaccurate.

Structural Similar Compounds

Name	ERYTHROMYCIN	RESERPINE	C.I.PIGMENT RED 23
Structure	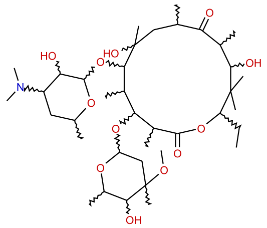	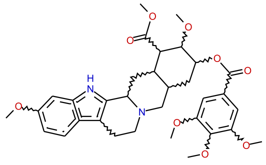	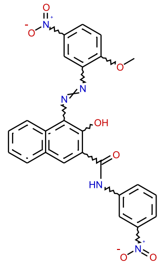
Actual Endpoint (-log C)	3.29629	6.13118	2.30052
Predicted Endpoint (-log C)	4.83895	4.38304	3.55333
Distance	1.109	1.326	1.359
Reference	NCI/NTP TR-338	NCI/NTP TR-193	NCI/NTP TR-411

Model Applicability

Unknown features are fingerprint features in the query molecule, but not found in the training set.

1. Molecular_Weight out of range. Value: 836.07. Training min, max, mean, SD: 74.122, 731.95, 245.25, 106.4.
2. Molecular_PolarSurfaceArea out of range. Value: 207.89. Training min, max, mean, SD: 0, 201.84, 63.052, 40.7.
3. OPS PC8 out of range. Value: 4.75. Training min, max, SD, explained variance: -3.8548, 3.9137, 1.331, 0.0400.
4. OPS PC12 out of range. Value: -3.71. Training min, max, SD, explained variance: -2.364, 2.9228, 1.079, 0.0263.

Feature Contribution

Top features for positive contribution

Fingerprint	Bit/Smiles	Feature Structure	Score
FCFP_2	-885550502	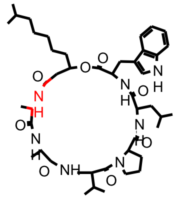 [*]C([*])NC(=[*])[*]	0.115

FCFP_2	-1143715940	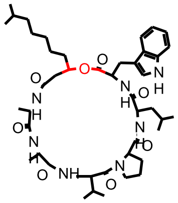 [*]C([*])OC([*])[*]	0.095
FCFP_2	3	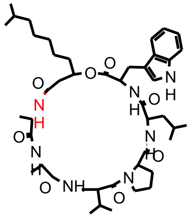 [*]O	0.074

Top Features for negative contribution

**Fingerprint**	**Bit/Smiles**	**Feature Structure**	**Score**
FCFP_2	-1272798659	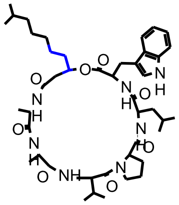 [*]CCC([*])([*])[*]	-0.111
FCFP_2	1872154524	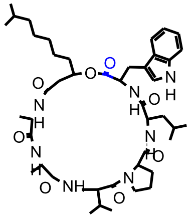 [*]C(=O)[*]	-0.105
FCFP_2	203677720	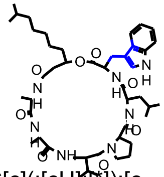 [*]C[c](:[cH]:[*]):[cH]:[*]	-0.083

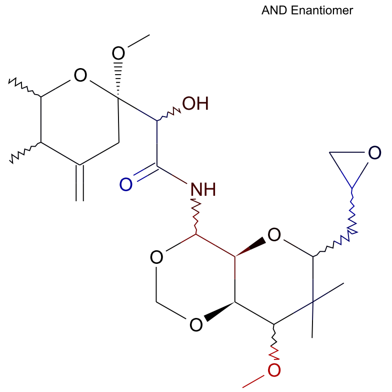

$C_{24}H_{39}NO_9$
Molecular Weight: 485.56776
ALogP: 0.267
Rotatable Bonds: 7
Acceptors: 9
Donors: 2

Model Prediction

Prediction: 0.018
Unit: g/kg_body_weight
Mahalanobis Distance: 9.765
Mahalanobis Distance p-value: 0.000105

Mahalanobis Distance: The Mahalanobis distance (MD) is a generalization of the Euclidean distance that accounts for correlations among the X properties. It is calculated as the distance to the center of the training data. The larger the MD, the less trustworthy the prediction.
Mahalanobis Distance p-value: The p-value gives the fraction of training data with an MD greater than or equal to the one for the given sample, assuming normally distributed data. The smaller the p-value, the less trustworthy the prediciton. For highly non-normal X properties (e.g., fingerprints), the MD p-value is wildly inaccurate.

Structural Similar Compounds

Name	LASIOCARPINE	MALAOXON	MATATHION
Structure	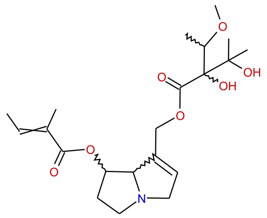	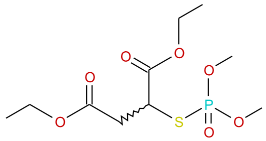	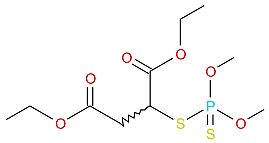
Actual Endpoint (-log C)	5.48403	3.84411	3.2637
Predicted Endpoint (-log C)	3.82301	3.97262	4.04535
Distance	0.539	0.781	0.806
Reference	NCI/NTP TR-039	NCI/NTP TR-135	NCI/NTP TR-192

Model Applicability

Unknown features are fingerprint features in the query molecule, but not found in the training set.

1. All properties and OPS components are within expected ranges.
2. Unknown FCFP_2 feature: 1327594201: [*]CC(O[*])(O[*])C([*])[*]

Feature Contribution

Top features for positive contribution			
**Fingerprint**	**Bit/Smiles**	**Feature Structure**	**Score**
FCFP_2	136627117	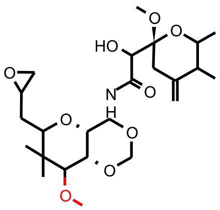 [*]OC	0.173

FCFP_2	-885550502	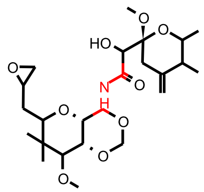 [*]C([*])NC(=[*])[*]	0.115
FCFP_2	-1143715940	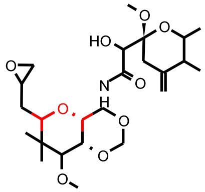 [*]C([*])OC([*])[*]	0.095
**Top Features for negative contribution**			
**Fingerprint**	**Bit/Smiles**	**Feature Structure**	**Score**
FCFP_2	-1272798659	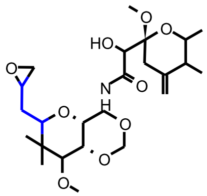 [*]CCC([*])([*])[*]	-0.111
FCFP_2	1872154524	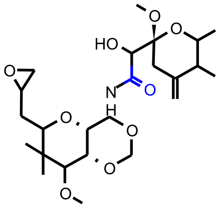 [*]C(=O)[*]	-0.105
FCFP_2	1	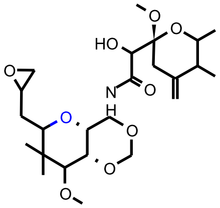 [*]O[*]	-0.080

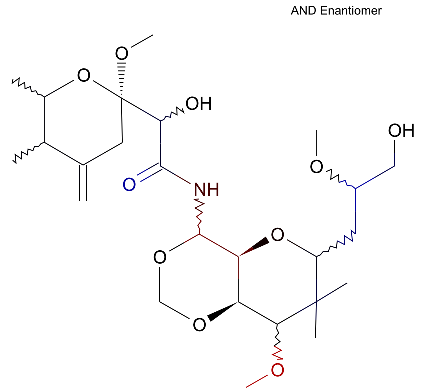

$C_{25}H_{43}NO_{10}$
Molecular Weight: 517.60962
ALogP: -9.1e-002
Rotatable Bonds: 9
Acceptors: 10
Donors: 3

Model Prediction

Prediction: 0.029
Unit: g/kg_body_weight
Mahalanobis Distance: 11.234
Mahalanobis Distance p-value: 3.34e-007

Mahalanobis Distance: The Mahalanobis distance (MD) is a generalization of the Euclidean distance that accounts for correlations among the X properties. It is calculated as the distance to the center of the training data. The larger the MD, the less trustworthy the prediction.
Mahalanobis Distance p-value: The p-value gives the fraction of training data with an MD greater than or equal to the one for the given sample, assuming normally distributed data. The smaller the p-value, the less trustworthy the prediciton. For highly non-normal X properties (e.g., fingerprints), the MD p-value is wildly inaccurate.

Structural Similar Compounds

Name	LASIOCARPINE	ERYTHROMYCIN	MATATHION
Structure	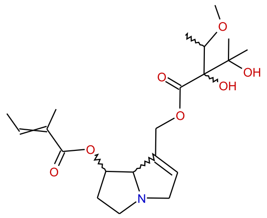	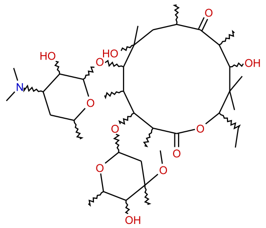	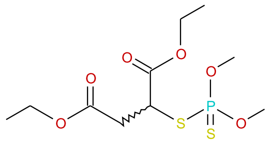
Actual Endpoint (-log C)	5.48403	3.29629	3.2637
Predicted Endpoint (-log C)	3.82301	4.83895	4.04535
Distance	0.652	0.887	0.931
Reference	NCI/NTP TR-039	NCI/NTP TR-338	NCI/NTP TR-192

Model Applicability

Unknown features are fingerprint features in the query molecule, but not found in the training set.

1. OPS PC9 out of range. Value: 3.5219. Training min, max, SD, explained variance: -2.8548, 3.3954, 1.263, 0.0360.
2. Unknown FCFP_2 feature: 1327594201: [*]CC(O[*])(O[*])C([*])[*]

Feature Contribution

Top features for positive contribution

Fingerprint	Bit/Smiles	Feature Structure	Score
FCFP_2	136627117	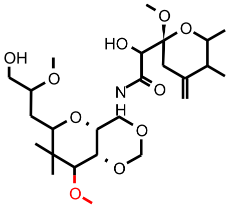 [*]OC	0.173

FCFP_2	-885550502	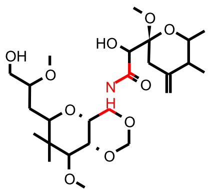 [*]C([*])NC(=[*])[*]	0.115
FCFP_2	-1143715940	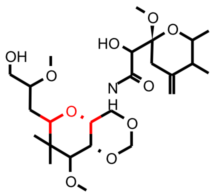 [*]C([*])OC([*])[*]	0.095
**Top Features for negative contribution**			
**Fingerprint**	**Bit/Smiles**	**Feature Structure**	**Score**
FCFP_2	-1272798659	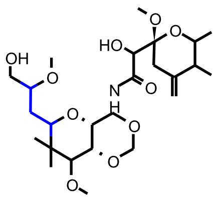 [*]CCC([*])([*])[*]	-0.111
FCFP_2	1872154524	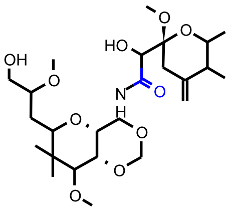 [*]C(=O)[*]	-0.105
FCFP_2	1	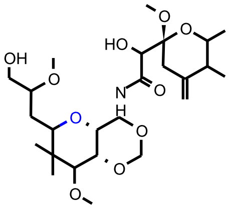 [*]O[*]	-0.080

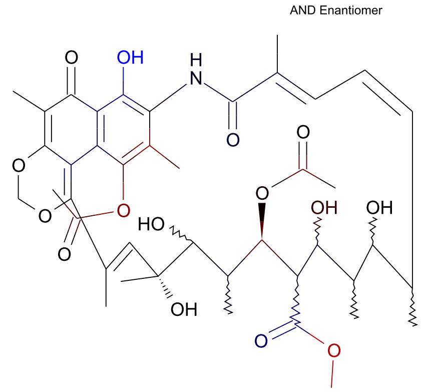

$C_{42}H_{53}NO_{15}$
Molecular Weight: 811.86791
ALogP: 2.745
Rotatable Bonds: 6
Acceptors: 15
Donors: 6

Structural Similar Compounds

Name	ERYTHROMYCIN	OXYTETRACYCLINE	4,4'-DIAMINO-2,2'-STILBENEDISULFONIC ACID.2NaSALT
Structure	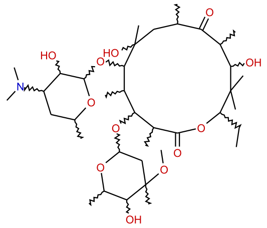	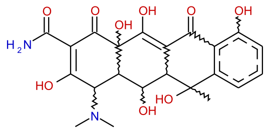	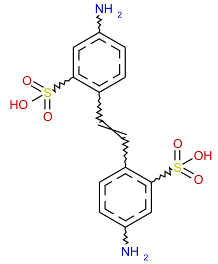
Actual Endpoint (-log C)	3.29629	2.36214	2.50759
Predicted Endpoint (-log C)	4.83895	2.77834	3.26068
Distance	0.962	1.168	1.445
Reference	NCI/NTP TR-338	NCI/NTP TR-315	NCI/NTP TR-412

Model Prediction

Prediction: 0.020
Unit: g/kg_body_weight
Mahalanobis Distance: 14.592
Mahalanobis Distance p-value: 7.96e-014

Mahalanobis Distance: The Mahalanobis distance (MD) is a generalization of the Euclidean distance that accounts for correlations among the X properties. It is calculated as the distance to the center of the training data. The larger the MD, the less trustworthy the prediction.
Mahalanobis Distance p-value: The p-value gives the fraction of training data with an MD greater than or equal to the one for the given sample, assuming normally distributed data. The smaller the p-value, the less trustworthy the prediciton. For highly non-normal X properties (e.g., fingerprints), the MD p-value is wildly inaccurate.

Model Applicability

Unknown features are fingerprint features in the query molecule, but not found in the training set.

1. Molecular_Weight out of range. Value: 811.87. Training min, max, mean, SD: 74.122, 731.95, 245.25, 106.4.
2. Num_H_Acceptors out of range. Value: 15. Training min, max, mean, SD: 0, 13, 3.2809, 2.199.
3. Molecular_PolarSurfaceArea out of range. Value: 244.67. Training min, max, mean, SD: 0, 201.84, 63.052, 40.7.
4. Molecular_PolarSASA out of range. Value: 373.6. Training min, max, mean, SD: 0, 357.83, 109.38, 65.5.
5. OPS PC9 out of range. Value: 4.4196. Training min, max, SD, explained variance: -2.8548, 3.3954, 1.263, 0.0360.
6. OPS PC13 out of range. Value: 3.6245. Training min, max, SD, explained variance: -2.9619, 2.8704, 1.049, 0.0249.

Feature Contribution

Top features for positive contribution

Fingerprint	Bit/Smiles	Feature Structure	Score

FCFP_2	136627117	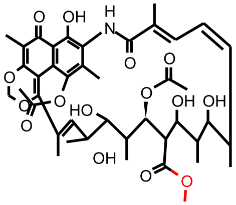 [*]OC	0.173
FCFP_2	-1143715940	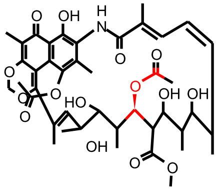 [*]C([*])OC([*])[*]	0.095
FCFP_2	1036089772	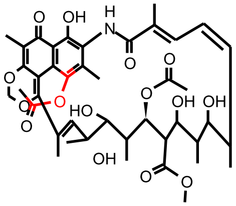 [*]:[c](:[*])OC	0.075

Top Features for negative contribution

Fingerprint	Bit/Smiles	Feature Structure	Score
FCFP_2	7	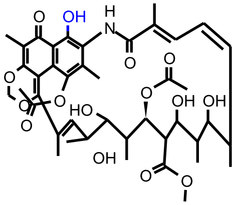 [*]O	-0.214
FCFP_2	-549108873	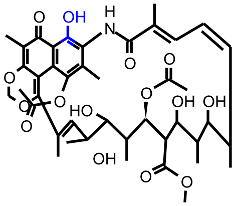 [*]:[c](:[*])O	-0.127

FCFP_2	1872154524	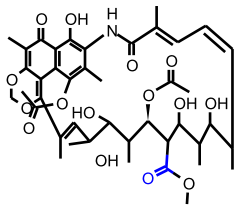 [*]C(=O)[*]	-0.105

Simeprevir

TOPKAT_Rat_Maximum_Tolerated_Dose_Feed

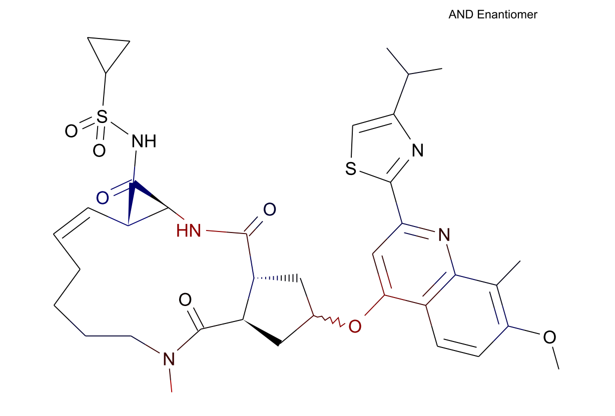

$C_{38}H_{47}N_5O_7S_2$

Molecular Weight: 749.93907

ALogP: 4.796

Rotatable Bonds: 8

Acceptors: 9

Donors: 2

Model Prediction

Prediction: 0.003

Unit: g/kg_body_weight

Mahalanobis Distance: 14.186

Mahalanobis Distance p-value: 5.46e-013

Mahalanobis Distance: The Mahalanobis distance (MD) is a generalization of the Euclidean distance that accounts for correlations among the X properties. It is calculated as the distance to the center of the training data. The larger the MD, the less trustworthy the prediction.
Mahalanobis Distance p-value: The p-value gives the fraction of training data with an MD greater than or equal to the one for the given sample, assuming normally distributed data. The smaller the p-value, the less trustworthy the prediciton. For highly non-normal X properties (e.g., fingerprints), the MD p-value is wildly inaccurate.

Structural Similar Compounds

Name	C.I.PIGMENT RED 23	RESERPINE	SALICYLAZOSULFAPYRIDINE
Structure	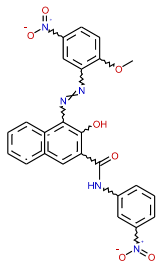	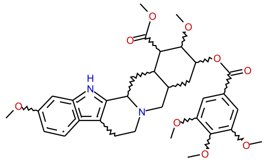	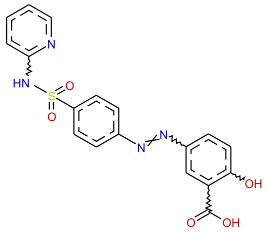
Actual Endpoint (-log C)	2.30052	6.13118	3.375
Predicted Endpoint (-log C)	3.55333	4.38304	2.80292
Distance	0.861	0.934	1.035
Reference	NCI/NTP TR-411	NCI/NTP TR-193	NCI/NTP TR-457

Model Applicability

Unknown features are fingerprint features in the query molecule, but not found in the training set.

1. Molecular_Weight out of range. Value: 749.94. Training min, max, mean, SD: 74.122, 731.95, 245.25, 106.4.
2. OPS PC9 out of range. Value: 5.11. Training min, max, SD, explained variance: -2.8548, 3.3954, 1.263, 0.0360.
3. OPS PC12 out of range. Value: -3.0299. Training min, max, SD, explained variance: -2.364, 2.9228, 1.079, 0.0263.
4. OPS PC14 out of range. Value: 4.236. Training min, max, SD, explained variance: -2.0656, 3.3808, 1.011, 0.0231.
5. Unknown FCFP_2 feature: 690481386: [*]:[c](:[*])[c]1:n:[*]:[*]:s:1
6. Unknown FCFP_2 feature: 1018942292: [*]NS(=O)(=O)C1[*][*]1

Feature Contribution

Top features for positive contribution			
Fingerprint	Bit/Smiles	Feature Structure	Score

FCFP_2	136627117	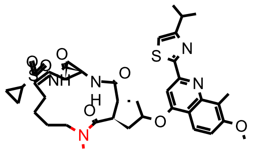 [*]OC	0.173
FCFP_2	-885550502	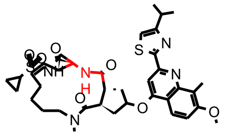 [*]C([*])NC(=[*])[*]	0.115
FCFP_2	1036089772	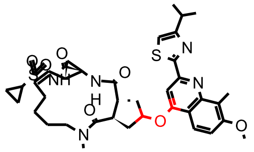 [*]:[c](:[*])OC	0.075
**Top Features for negative contribution**			
**Fingerprint**	**Bit/Smiles**	**Feature Structure**	**Score**
FCFP_2	-1272798659	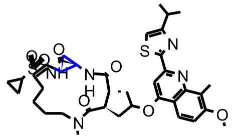 [*]CCC([*])([*])[*]	-0.111
FCFP_2	1872154524	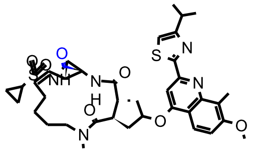 [*]C(=O)[*]	-0.105

FCFP_2	203677720	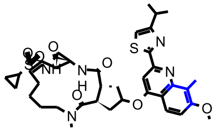	-0.083

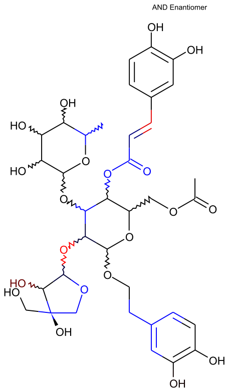

$C_{36}H_{46}O_{20}$

Molecular Weight: 798.73843

ALogP: -0.546

Rotatable Bonds: 16

Acceptors: 20

Donors: 10

Model Prediction

Prediction: 0.000

Unit: g/kg_body_weight

Mahalanobis Distance: 32.711

Mahalanobis Distance p-value: 1.81e-032

Mahalanobis Distance: The Mahalanobis distance (MD) is a generalization of the Euclidean distance that accounts for correlations among the X properties. It is calculated as the distance to the center of the training data. The larger the MD, the less trustworthy the prediction.
Mahalanobis Distance p-value: The p-value gives the fraction of training data with an MD greater than or equal to the one for the given sample, assuming normally distributed data. The smaller the p-value, the less trustworthy the prediciton. For highly non-normal X properties (e.g., fingerprints), the MD p-value is wildly inaccurate.

Structural Similar Compounds

Name	AMPICILLIN TRIHYDRATE	OCHRATOXIN	PENICILLIN VK
Structure	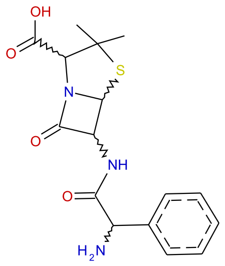	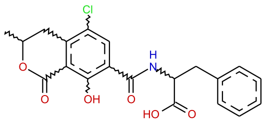	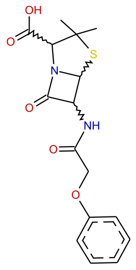
Actual Endpoint (-log C)	2.36724	6.28396	2.54455
Predicted Endpoint (-log C)	2.27651	5.12358	3.9702
Distance	3.016	3.070	3.220
Reference	NCI/NTP TR-318	NCI/NTP TR-358	NCI/NTP TR-336

Model Applicability

Unknown features are fingerprint features in the query molecule, but not found in the training set.

1. Molecular_Weight out of range. Value: 798.74. Training min, max, mean, SD: 68.074, 434.63, 171.13, 85.06.
2. Num_H_Donors out of range. Value: 10. Training min, max, mean, SD: 0, 3, 0.4375, 0.8311.
3. Num_H_Acceptors out of range. Value: 20. Training min, max, mean, SD: 0, 6, 1.6146, 1.644.
4. Molecular_PolarSASA out of range. Value: 473.59. Training min, max, mean, SD: 0, 223.97, 50.816, 55.15.
5. Molecular_PolarSurfaceArea out of range. Value: 310.28. Training min, max, mean, SD: 0, 138.03, 28.978, 32.1.
6. OPS PC1 out of range. Value: 18.036. Training min, max, SD, explained variance: -4.0008, 7.9165, 2.861, 0.2531.
7. OPS PC3 out of range. Value: -5.0208. Training min, max, SD, explained variance: -4.6235, 5.1158, 1.773, 0.0972.
8. OPS PC4 out of range. Value: 6.5302. Training min, max, SD, explained variance: -2.9402, 6.1938, 1.581, 0.0773.
9. OPS PC5 out of range. Value: -3.4665. Training min, max, SD, explained variance: -3.4, 4.1587, 1.489, 0.0686.
10. Unknown FCFP_2 feature: -415156552: [*]C[C@@]1(O)C[*][*]C1[*]

Feature Contribution

Top features for positive contribution

Fingerprint	Bit/Smiles	Feature Structure	Score

FCFP_2	1	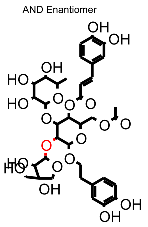 [*]O[*]	0.511
FCFP_2	451847724	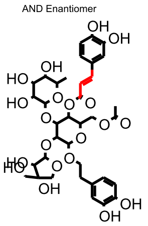 [*]\C=C\C(=[*])[*]	0.225
FCFP_2	3	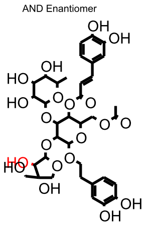 [*]O	0.104

Top Features for negative contribution

Fingerprint	Bit/Smiles	Feature Structure	Score
FCFP_2	136597326	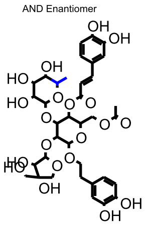 [*]C([*])C	-0.489
FCFP_2	203677720	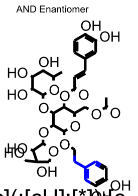 [*]C[c](:[cH]:[*]):[cH]:[*]	-0.406

FCFP_2	565998553	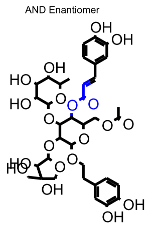 [*]OC(=O)C=[*]	-0.348

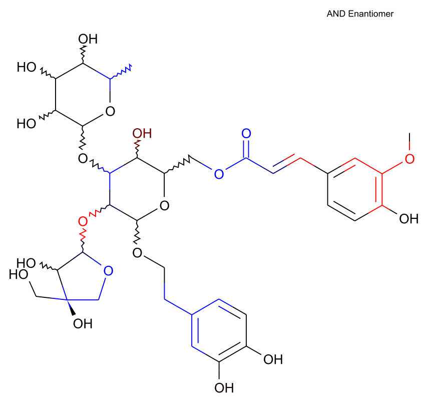

$C_{35}H_{46}O_{19}$

Molecular Weight: 770.72833

ALogP: -0.699

Rotatable Bonds: 15

Acceptors: 19

Donors: 10

Model Prediction

Prediction: 0.000

Unit: g/kg_body_weight

Mahalanobis Distance: 31.696

Mahalanobis Distance p-value: 1.36e-031

Mahalanobis Distance: The Mahalanobis distance (MD) is a generalization of the Euclidean distance that accounts for correlations among the X properties. It is calculated as the distance to the center of the training data. The larger the MD, the less trustworthy the prediction.
Mahalanobis Distance p-value: The p-value gives the fraction of training data with an MD greater than or equal to the one for the given sample, assuming normally distributed data. The smaller the p-value, the less trustworthy the prediciton. For highly non-normal X properties (e.g., fingerprints), the MD p-value is wildly inaccurate.

Structural Similar Compounds

Name	AMPICILLIN TRIHYDRATE	OCHRATOXIN	PENICILLIN VK
Structure	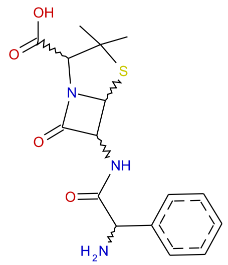	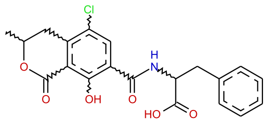	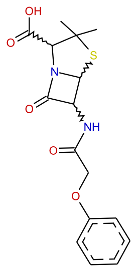
Actual Endpoint (-log C)	2.36724	6.28396	2.54455
Predicted Endpoint (-log C)	2.27651	5.12358	3.9702
Distance	2.886	2.940	3.049
Reference	NCI/NTP TR-318	NCI/NTP TR-358	NCI/NTP TR-336

Model Applicability

Unknown features are fingerprint features in the query molecule, but not found in the training set.

1. Molecular_Weight out of range. Value: 770.73. Training min, max, mean, SD: 68.074, 434.63, 171.13, 85.06.
2. Num_H_Donors out of range. Value: 10. Training min, max, mean, SD: 0, 3, 0.4375, 0.8311.
3. Num_H_Acceptors out of range. Value: 19. Training min, max, mean, SD: 0, 6, 1.6146, 1.644.
4. Molecular_PolarSASA out of range. Value: 446.75. Training min, max, mean, SD: 0, 223.97, 50.816, 55.15.
5. Molecular_PolarSurfaceArea out of range. Value: 293.2. Training min, max, mean, SD: 0, 138.03, 28.978, 32.1.
6. OPS PC1 out of range. Value: 17.908. Training min, max, SD, explained variance: -4.0008, 7.9165, 2.861, 0.2531.
7. OPS PC5 out of range. Value: -4.1198. Training min, max, SD, explained variance: -3.4, 4.1587, 1.489, 0.0686.
8. Unknown FCFP_2 feature: -415156552: [*]C[C@@]1(O)C[*][*]C1[*]

Feature Contribution

Top features for positive contribution			
**Fingerprint**	**Bit/Smiles**	**Feature Structure**	**Score**

FCFP_2	332760439	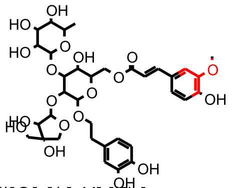 [*]O[c](:[cH]:[*]):[c]([*]):[*]	0.672
FCFP_2	1	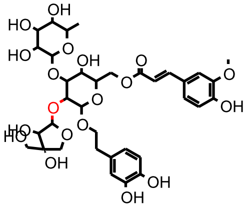 [*]O[*]	0.511
FCFP_2	451847724	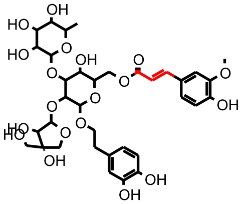 [*]\C=C\C(=[*])[*]	0.225
**Top Features for negative contribution**			
**Fingerprint**	**Bit/Smiles**	**Feature Structure**	**Score**
FCFP_2	136597326	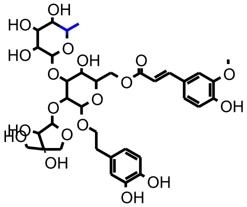 [*]C([*])C	-0.489
FCFP_2	203677720	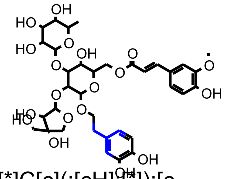 [*]C[c](:[cH]:[*]):[c H]:[*]	-0.406

FCFP_2	565998553	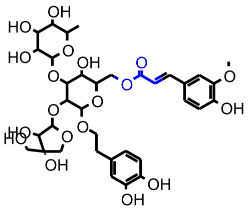 [*]OC(=O)C=[*]	-0.348

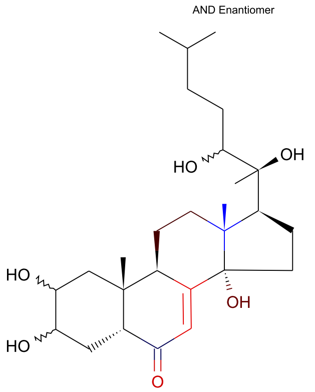

$C_{27}H_{44}O_6$
Molecular Weight: 464.63466
ALogP: 2.489
Rotatable Bonds: 5
Acceptors: 6
Donors: 5

Model Prediction

Prediction: 0.011
Unit: g/kg_body_weight
Mahalanobis Distance: 15.608
Mahalanobis Distance p-value: 7.66e-014

Mahalanobis Distance: The Mahalanobis distance (MD) is a generalization of the Euclidean distance that accounts for correlations among the X properties. It is calculated as the distance to the center of the training data. The larger the MD, the less trustworthy the prediction.
Mahalanobis Distance p-value: The p-value gives the fraction of training data with an MD greater than or equal to the one for the given sample, assuming normally distributed data. The smaller the p-value, the less trustworthy the prediciton. For highly non-normal X properties (e.g., fingerprints), the MD p-value is wildly inaccurate.

Structural Similar Compounds

Name	OCHRATOXIN	LITHOCHLOLIC ACID	AMPICILLIN TRIHYDRATE
Structure	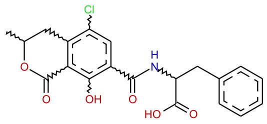	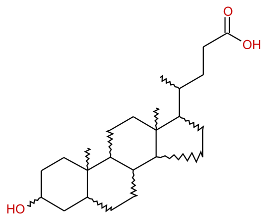	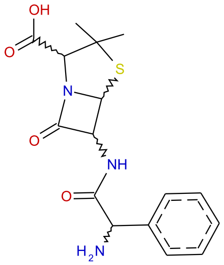
Actual Endpoint (-log C)	6.28396	2.87689	2.36724
Predicted Endpoint (-log C)	5.12358	3.8262	2.27651
Distance	1.097	1.139	1.149
Reference	NCI/NTP TR-358	NCI/NTP TR-175	NCI/NTP TR-318

Model Applicability

Unknown features are fingerprint features in the query molecule, but not found in the training set.

1. Molecular_Weight out of range. Value: 464.63. Training min, max, mean, SD: 68.074, 434.63, 171.13, 85.06.
2. Num_H_Donors out of range. Value: 5. Training min, max, mean, SD: 0, 3, 0.4375, 0.8311.
3. OPS PC7 out of range. Value: -3.0183. Training min, max, SD, explained variance: -2.8003, 2.9332, 1.16, 0.0416.
4. Unknown FCFP_2 feature: -415156552: [*]C[C@@]1(O)C[*][*]C1[*]

Feature Contribution

Top features for positive contribution

Fingerprint	Bit/Smiles	Feature Structure	Score
FCFP_2	1	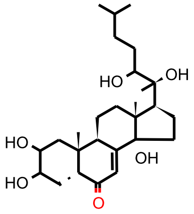 [*]O[*]	0.511

FCFP_2	451847724	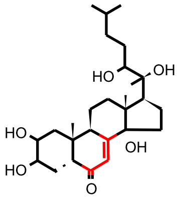 [*]\C=C\C(=[*])[*]	0.225
FCFP_2	3	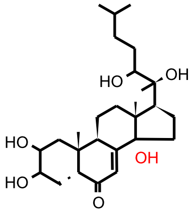 [*]O	0.104

Top Features for negative contribution

Fingerprint	Bit/Smiles	Feature Structure	Score
FCFP_2	136597326	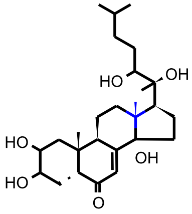 [*]C([*])C	-0.489
FCFP_2	1872154524	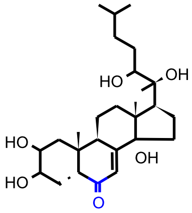 [*]C(=O)[*]	-0.307
FCFP_2	0	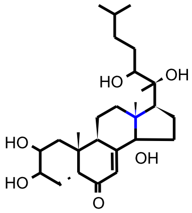 [*]C([*])[*]	-0.290

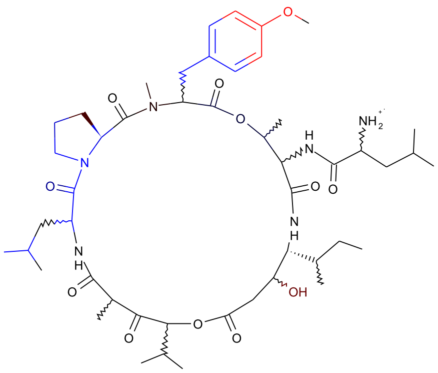

$C_{49}H_{79}N_6O_{12}$
Molecular Weight: 944.18455
ALogP: 3.176
Rotatable Bonds: 13
Acceptors: 12
Donors: 5

Model Prediction

Prediction: 0.000
Unit: g/kg_body_weight
Mahalanobis Distance: 21.220
Mahalanobis Distance p-value: 5.69e-021

Mahalanobis Distance: The Mahalanobis distance (MD) is a generalization of the Euclidean distance that accounts for correlations among the X properties. It is calculated as the distance to the center of the training data. The larger the MD, the less trustworthy the prediction.
Mahalanobis Distance p-value: The p-value gives the fraction of training data with an MD greater than or equal to the one for the given sample, assuming normally distributed data. The smaller the p-value, the less trustworthy the prediciton. For highly non-normal X properties (e.g., fingerprints), the MD p-value is wildly inaccurate.

Structural Similar Compounds

Name	OCHRATOXIN	AMPICILLIN TRIHYDRATE	PENICILLIN VK
Structure	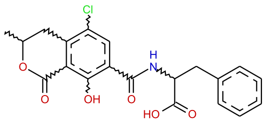	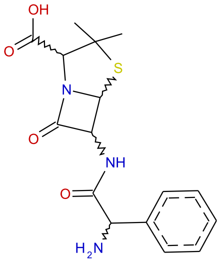	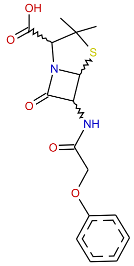
Actual Endpoint (-log C)	6.28396	2.36724	2.54455
Predicted Endpoint (-log C)	5.12358	2.27651	3.9702
Distance	2.033	2.072	2.076
Reference	NCI/NTP TR-358	NCI/NTP TR-318	NCI/NTP TR-336

Model Applicability

Unknown features are fingerprint features in the query molecule, but not found in the training set.

1. Molecular_Weight out of range. Value: 944.18. Training min, max, mean, SD: 68.074, 434.63, 171.13, 85.06.
2. Num_H_Donors out of range. Value: 5. Training min, max, mean, SD: 0, 3, 0.4375, 0.8311.
3. Num_H_Acceptors out of range. Value: 12. Training min, max, mean, SD: 0, 6, 1.6146, 1.644.
4. Molecular_PolarSASA out of range. Value: 320.47. Training min, max, mean, SD: 0, 223.97, 50.816, 55.15.
5. Molecular_PolarSurfaceArea out of range. Value: 243.65. Training min, max, mean, SD: 0, 138.03, 28.978, 32.1.
6. OPS PC1 out of range. Value: 12.091. Training min, max, SD, explained variance: -4.0008, 7.9165, 2.861, 0.2531.
7. OPS PC5 out of range. Value: -4.779. Training min, max, SD, explained variance: -3.4, 4.1587, 1.489, 0.0686.
8. OPS PC6 out of range. Value: -3.2308. Training min, max, SD, explained variance: -2.4321, 2.9885, 1.256, 0.0488.
9. Unknown FCFP_2 feature: 10: [*][NH2+][*]
10. Unknown FCFP_2 feature: 18028531: [*]C([*])[NH2+]C
11. Unknown FCFP_2 feature: -1817836174: [*]CC([NH2+][*])C(=[*])[*]
12. Unknown FCFP_2 feature: 136418580: [*][NH2+]C

Feature Contribution

Top features for positive contribution

Fingerprint	Bit/Smiles	Feature Structure	Score
FCFP_2	332760439	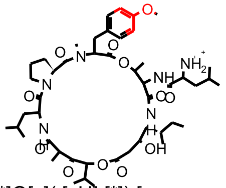 [*]O[c](:[cH]:[*]):[c]([*]):[*]	0.672
FCFP_2	1	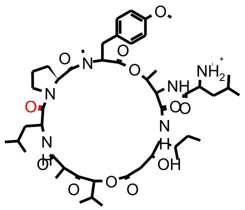 [*]O[*]	0.511
FCFP_2	3	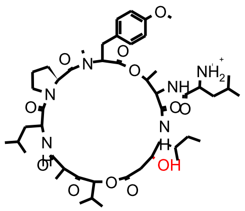 [*]O	0.104

Top Features for negative contribution

Fingerprint	Bit/Smiles	Feature Structure	Score
FCFP_2	136597326	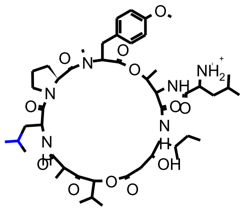 [*]C([*])C	-0.489

FCFP_2	203677720	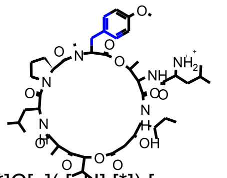 [*]C[c](:[cH]:[*]):[cH]:[*]	-0.406
FCFP_2	565998553	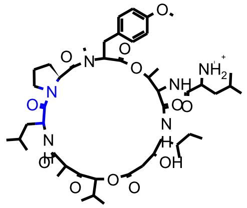 [*]OC(=O)C=[*]	-0.348

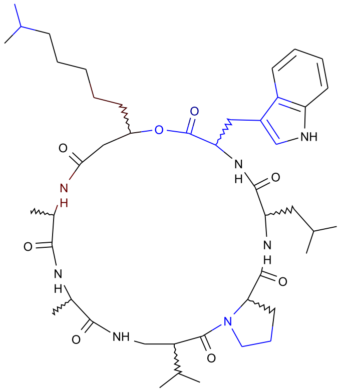

$C_{45}H_{69}N_7O_8$
Molecular Weight: 836.07145
ALogP: 4.986
Rotatable Bonds: 11
Acceptors: 8
Donors: 6

Model Prediction

Prediction: 0.076
Unit: g/kg_body_weight
Mahalanobis Distance: 20.938
Mahalanobis Distance p-value: 1.22e-020

Mahalanobis Distance: The Mahalanobis distance (MD) is a generalization of the Euclidean distance that accounts for correlations among the X properties. It is calculated as the distance to the center of the training data. The larger the MD, the less trustworthy the prediction.
Mahalanobis Distance p-value: The p-value gives the fraction of training data with an MD greater than or equal to the one for the given sample, assuming normally distributed data. The smaller the p-value, the less trustworthy the prediciton. For highly non-normal X properties (e.g., fingerprints), the MD p-value is wildly inaccurate.

Structural Similar Compounds

Name	OCHRATOXIN	AMPICILLIN TRIHYDRATE	PENICILLIN VK
Structure	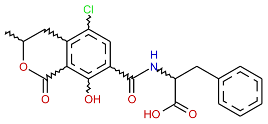	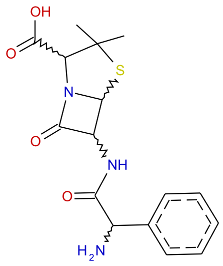	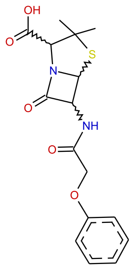
Actual Endpoint (-log C)	6.28396	2.36724	2.54455
Predicted Endpoint (-log C)	5.12358	2.27651	3.9702
Distance	1.679	1.876	1.880
Reference	NCI/NTP TR-358	NCI/NTP TR-318	NCI/NTP TR-336

Model Applicability

Unknown features are fingerprint features in the query molecule, but not found in the training set.

1. Molecular_Weight out of range. Value: 836.07. Training min, max, mean, SD: 68.074, 434.63, 171.13, 85.06.
2. Num_H_Donors out of range. Value: 6. Training min, max, mean, SD: 0, 3, 0.4375, 0.8311.
3. Num_H_Acceptors out of range. Value: 8. Training min, max, mean, SD: 0, 6, 1.6146, 1.644.
4. Molecular_PolarSASA out of range. Value: 302.44. Training min, max, mean, SD: 0, 223.97, 50.816, 55.15.
5. Molecular_PolarSurfaceArea out of range. Value: 207.89. Training min, max, mean, SD: 0, 138.03, 28.978, 32.1.
6. OPS PC1 out of range. Value: 10.753. Training min, max, SD, explained variance: -4.0008, 7.9165, 2.861, 0.2531.
7. OPS PC9 out of range. Value: -3.927. Training min, max, SD, explained variance: -2.7086, 2.9267, 1.019, 0.0321.
8. Unknown FCFP_2 feature: 19: [*]:[nH]:[*]
9. Unknown FCFP_2 feature: 1618184456: [*][c]1:[*]:[*]:[nH]:c:1
10. Unknown FCFP_2 feature: 307448885: [*]:[c]1:[*]:[*]:[nH]:[c]:1:c:[*]
11. Unknown FCFP_2 feature: 2005402822: [*]:[c]1:[*]:[*]:c:[nH]:1

Feature Contribution

Top features for positive contribution			
Fingerprint	Bit/Smiles	Feature Structure	Score

FCFP_2	1	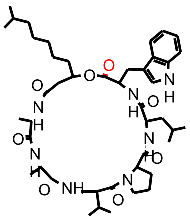 [*]O[*]	0.511
FCFP_2	3	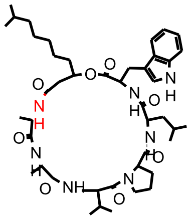 [*]O	0.104
FCFP_2	-1272798659	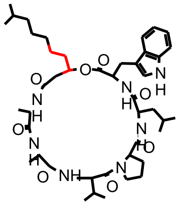 [*]CCC([*])([*])[*]	0.070
**Top Features for negative contribution**			
**Fingerprint**	**Bit/Smiles**	**Feature Structure**	**Score**
FCFP_2	136597326	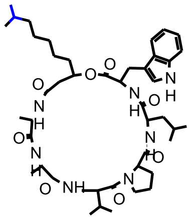 [*]C([*])C	-0.489
FCFP_2	203677720	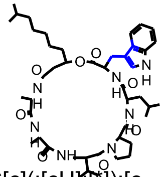 [*]C[c](:[cH]:[*]):[cH]:[*]	-0.406

FCFP_2	565998553	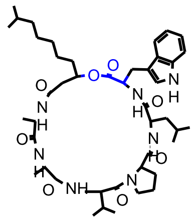[*]OC(=O)C=[*]	-0.348

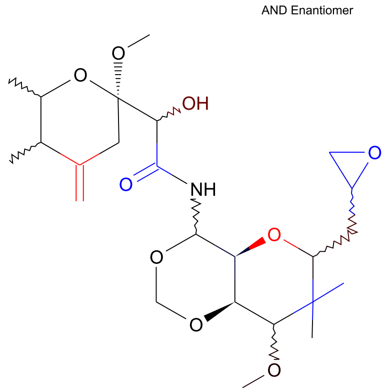

$C_{24}H_{39}NO_9$

Molecular Weight: 485.56776

ALogP: 0.267

Rotatable Bonds: 7

Acceptors: 9

Donors: 2

Model Prediction

Prediction: 0.000

Unit: g/kg_body_weight

Mahalanobis Distance: 14.212

Mahalanobis Distance p-value: 6.51e-012

Mahalanobis Distance: The Mahalanobis distance (MD) is a generalization of the Euclidean distance that accounts for correlations among the X properties. It is calculated as the distance to the center of the training data. The larger the MD, the less trustworthy the prediction.
Mahalanobis Distance p-value: The p-value gives the fraction of training data with an MD greater than or equal to the one for the given sample, assuming normally distributed data. The smaller the p-value, the less trustworthy the prediciton. For highly non-normal X properties (e.g., fingerprints), the MD p-value is wildly inaccurate.

Structural Similar Compounds

Name	PENICILLIN VK	AMPICILLIN TRIHYDRATE	OCHRATOXIN
Structure	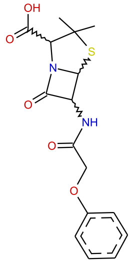	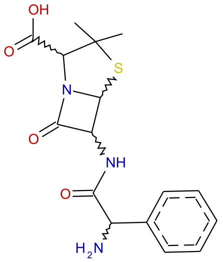	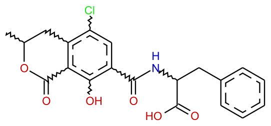
Actual Endpoint (-log C)	2.54455	2.36724	6.28396
Predicted Endpoint (-log C)	3.9702	2.27651	5.12358
Distance	0.867	1.038	1.144
Reference	NCI/NTP TR-336	NCI/NTP TR-318	NCI/NTP TR-358

Model Applicability

Unknown features are fingerprint features in the query molecule, but not found in the training set.

1. Molecular_Weight out of range. Value: 485.57. Training min, max, mean, SD: 68.074, 434.63, 171.13, 85.06.
2. Num_H_Acceptors out of range. Value: 9. Training min, max, mean, SD: 0, 6, 1.6146, 1.644.
3. OPS PC2 out of range. Value: 5.1088. Training min, max, SD, explained variance: -5.7911, 4.8501, 2.284, 0.1613.
4. Unknown FCFP_2 feature: 699559848: [*]NC(O[*])C([*])[*]
5. Unknown FCFP_2 feature: 470041467: [*]OCO[*]
6. Unknown FCFP_2 feature: 1327594201: [*]CC(O[*])(O[*])C([*])[*]

Feature Contribution

Top features for positive contribution			
Fingerprint	Bit/Smiles	Feature Structure	Score
FCFP_2	129344189	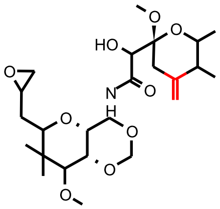 [*]C(=C)[*]	0.519

FCFP_2	1	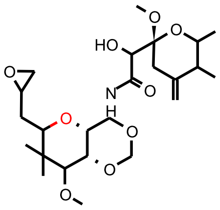<br>[*]O[*]	0.511
FCFP_2	3	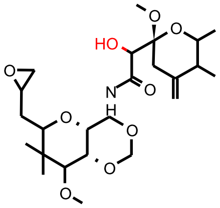<br>[*]O	0.104

Top Features for negative contribution

Fingerprint	Bit/Smiles	Feature Structure	Score
FCFP_2	136597326	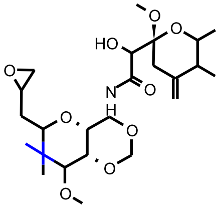<br>[*]C([*])C	-0.489
FCFP_2	1872154524	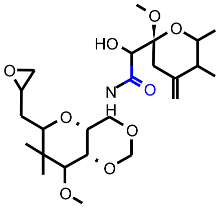<br>[*]C(=O)[*]	-0.307
FCFP_2	0	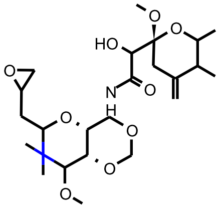<br>[*]C([*])[*]	-0.290

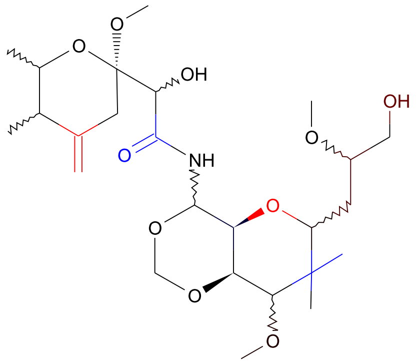

$C_{25}H_{43}NO_{10}$
Molecular Weight: 517.60962
ALogP: -9.1e-002
Rotatable Bonds: 9
Acceptors: 10
Donors: 3

Model Prediction

Prediction: 0.000
Unit: g/kg_body_weight
Mahalanobis Distance: 14.314
Mahalanobis Distance p-value: 4.69e-012

Mahalanobis Distance: The Mahalanobis distance (MD) is a generalization of the Euclidean distance that accounts for correlations among the X properties. It is calculated as the distance to the center of the training data. The larger the MD, the less trustworthy the prediction.
Mahalanobis Distance p-value: The p-value gives the fraction of training data with an MD greater than or equal to the one for the given sample, assuming normally distributed data. The smaller the p-value, the less trustworthy the prediciton. For highly non-normal X properties (e.g., fingerprints), the MD p-value is wildly inaccurate.

Structural Similar Compounds

Name	PENICILLIN VK	AMPICILLIN TRIHYDRATE	OCHRATOXIN
Structure	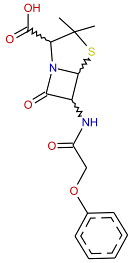	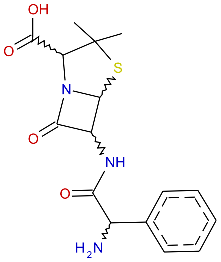	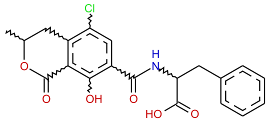
Actual Endpoint (-log C)	2.54455	2.36724	6.28396
Predicted Endpoint (-log C)	3.9702	2.27651	5.12358
Distance	1.058	1.079	1.222
Reference	NCI/NTP TR-336	NCI/NTP TR-318	NCI/NTP TR-358

Model Applicability

Unknown features are fingerprint features in the query molecule, but not found in the training set.

1. Molecular_Weight out of range. Value: 517.61. Training min, max, mean, SD: 68.074, 434.63, 171.13, 85.06.
2. Num_H_Acceptors out of range. Value: 10. Training min, max, mean, SD: 0, 6, 1.6146, 1.644.
3. Unknown FCFP_2 feature: 699559848: [*]NC(O[*])C([*])[*]
4. Unknown FCFP_2 feature: 470041467: [*]OCO[*]
5. Unknown FCFP_2 feature: 1327594201: [*]CC(O[*])(O[*])C([*])[*]

Feature Contribution

Top features for positive contribution

Fingerprint	Bit/Smiles	Feature Structure	Score
FCFP_2	129344189	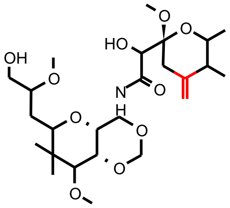 [*]C(=C)[*]	0.519

FCFP_2	1	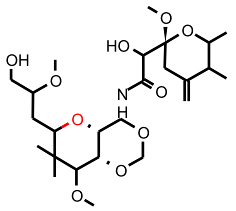 [*]O[*]	0.511
FCFP_2	3	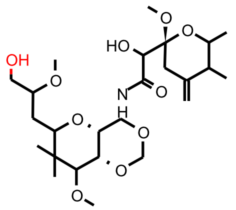 [*]O	0.104

Top Features for negative contribution

Fingerprint	Bit/Smiles	Feature Structure	Score
FCFP_2	136597326	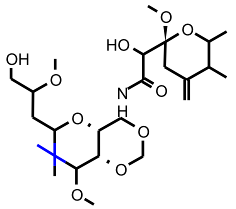 [*]C([*])C	-0.489
FCFP_2	1872154524	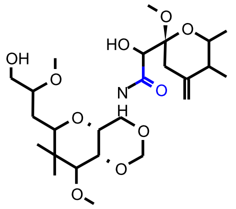 [*]C(=O)[*]	-0.307
FCFP_2	0	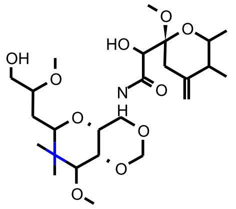 [*]C([*])[*]	-0.290

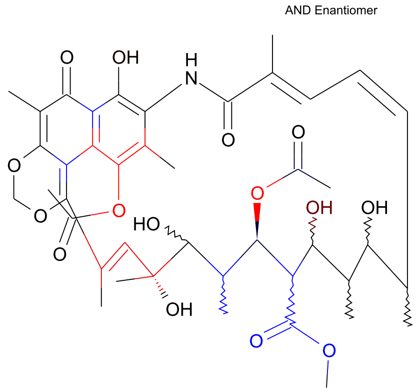

$C_{42}H_{53}NO_{15}$
Molecular Weight: 811.86791
ALogP: 2.745
Rotatable Bonds: 6
Acceptors: 15
Donors: 6

Model Prediction

Prediction: 0.000
Unit: g/kg_body_weight
Mahalanobis Distance: 21.357
Mahalanobis Distance p-value: 3.92e-021

Mahalanobis Distance: The Mahalanobis distance (MD) is a generalization of the Euclidean distance that accounts for correlations among the X properties. It is calculated as the distance to the center of the training data. The larger the MD, the less trustworthy the prediction.
Mahalanobis Distance p-value: The p-value gives the fraction of training data with an MD greater than or equal to the one for the given sample, assuming normally distributed data. The smaller the p-value, the less trustworthy the prediciton. For highly non-normal X properties (e.g., fingerprints), the MD p-value is wildly inaccurate.

Structural Similar Compounds

Name	OCHRATOXIN	AMPICILLIN TRIHYDRATE	PENICILLIN VK
Structure	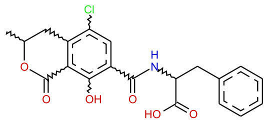	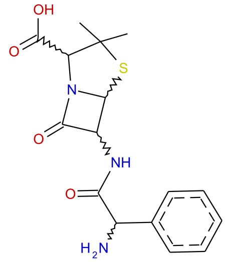	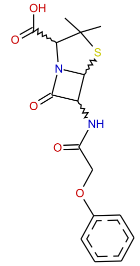
Actual Endpoint (-log C)	6.28396	2.36724	2.54455
Predicted Endpoint (-log C)	5.12358	2.27651	3.9702
Distance	2.082	2.101	2.191
Reference	NCI/NTP TR-358	NCI/NTP TR-318	NCI/NTP TR-336

Model Applicability

Unknown features are fingerprint features in the query molecule, but not found in the training set.

1. Molecular_Weight out of range. Value: 811.87. Training min, max, mean, SD: 68.074, 434.63, 171.13, 85.06.
2. Num_H_Donors out of range. Value: 6. Training min, max, mean, SD: 0, 3, 0.4375, 0.8311.
3. Num_H_Acceptors out of range. Value: 15. Training min, max, mean, SD: 0, 6, 1.6146, 1.644.
4. Molecular_PolarSASA out of range. Value: 373.6. Training min, max, mean, SD: 0, 223.97, 50.816, 55.15.
5. Molecular_PolarSurfaceArea out of range. Value: 244.67. Training min, max, mean, SD: 0, 138.03, 28.978, 32.1.
6. OPS PC1 out of range. Value: 13.716. Training min, max, SD, explained variance: -4.0008, 7.9165, 2.861, 0.2531.
7. Unknown FCFP_2 feature: -415156552: [*]C[C@@]1(O)C[*][*]C1[*]
8. Unknown FCFP_2 feature: 436915834: [*]OC(=C([*])[*])C(=[*])[*]
9. Unknown FCFP_2 feature: -1678275541: [*]C(=C(C(=[*])[*])[c](:[*]):[*])[*]
10. Unknown FCFP_2 feature: 470041467: [*]OCO[*]
11. Unknown FCFP_2 feature: -1549192822: [*]C(=[*])C(=O)[c](:[*]):[*]

Feature Contribution

Top features for positive contribution			
Fingerprint	Bit/Smiles	Feature Structure	Score

FCFP_2	332760439	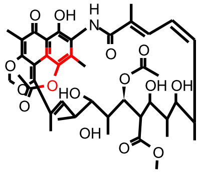<br>[*]O[c](:[cH]:[*]):[c<br>]([*]):[*]	0.672
FCFP_2	1	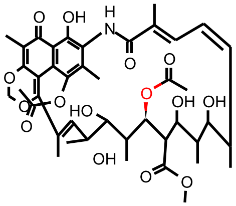<br>[*]O[*]	0.511
FCFP_2	451847724	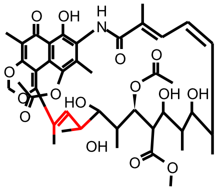<br>[*]\C=C\C(=[*])[*]	0.225
**Top Features for negative contribution**			
**Fingerprint**	**Bit/Smiles**	**Feature Structure**	**Score**
FCFP_2	136597326	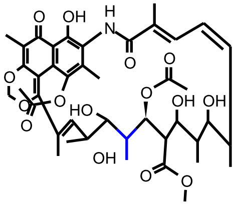<br>[*]C([*])C	-0.489
FCFP_2	203677720	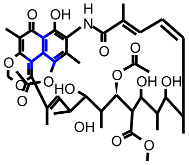<br>[*]C[c](:[cH]:[*]):[c<br>H]:[*]	-0.406

FCFP_2	565998553	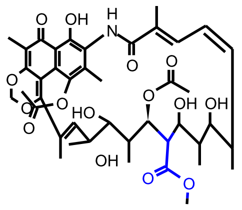<br>[*]OC(=O)C=[*]	-0.348

Simeprevir

TOPKAT_Rat_Maximum_Tolerated_Dose_Gavage

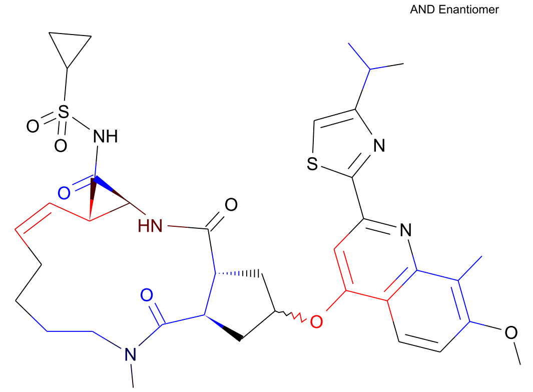

$C_{38}H_{47}N_5O_7S_2$

Molecular Weight: 749.93907

ALogP: 4.796

Rotatable Bonds: 8

Acceptors: 9

Donors: 2

Model Prediction

Prediction: 0.001

Unit: g/kg_body_weight

Mahalanobis Distance: 16.823

Mahalanobis Distance p-value: 1.77e-015

Mahalanobis Distance: The Mahalanobis distance (MD) is a generalization of the Euclidean distance that accounts for correlations among the X properties. It is calculated as the distance to the center of the training data. The larger the MD, the less trustworthy the prediction.
Mahalanobis Distance p-value: The p-value gives the fraction of training data with an MD greater than or equal to the one for the given sample, assuming normally distributed data. The smaller the p-value, the less trustworthy the prediciton. For highly non-normal X properties (e.g., fingerprints), the MD p-value is wildly inaccurate.

Structural Similar Compounds

Name	OCHRATOXIN	PENICILLIN VK	AMPICILLIN TRIHYDRATE
Structure	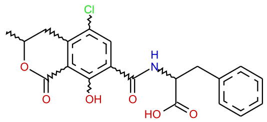	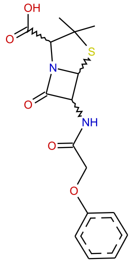	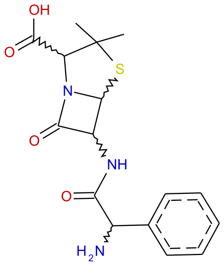
Actual Endpoint (-log C)	6.28396	2.54455	2.36724
Predicted Endpoint (-log C)	5.12358	3.9702	2.27651
Distance	1.425	1.545	1.716
Reference	NCI/NTP TR-358	NCI/NTP TR-336	NCI/NTP TR-318

Model Applicability

Unknown features are fingerprint features in the query molecule, but not found in the training set.

1. Molecular_Weight out of range. Value: 749.94. Training min, max, mean, SD: 68.074, 434.63, 171.13, 85.06.
2. Num_H_Acceptors out of range. Value: 9. Training min, max, mean, SD: 0, 6, 1.6146, 1.644.
3. Num_AromaticRings out of range. Value: 3. Training min, max, mean, SD: 0, 2, 0.5625, 0.693.
4. Molecular_PolarSASA out of range. Value: 272.1. Training min, max, mean, SD: 0, 223.97, 50.816, 55.15.
5. Molecular_PolarSurfaceArea out of range. Value: 193.5. Training min, max, mean, SD: 0, 138.03, 28.978, 32.1.
6. OPS PC1 out of range. Value: 9.6287. Training min, max, SD, explained variance: -4.0008, 7.9165, 2.861, 0.2531.
7. OPS PC6 out of range. Value: -3.4118. Training min, max, SD, explained variance: -2.4321, 2.9885, 1.256, 0.0488.
8. Unknown FCFP_2 feature: -415156552: [*]C[C@@]1(O)C[*][*]C1[*]
9. Unknown FCFP_2 feature: 690511177: [*]:n:[c]([c](:[*]):[*]):c:[*]
10. Unknown FCFP_2 feature: 690481386: [*]:[c](:[*])[c]1:n:[*]:[*]:s:1
11. Unknown FCFP_2 feature: 1018942292: [*]NS(=O)(=O)C1[*][*]1

Feature Contribution

Top features for positive contribution

Fingerprint	Bit/Smiles	Feature Structure	Score

FCFP_2	332760439	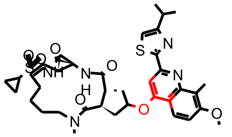 [*]O[c](:[cH]:[*]):[c]([*]):[*]	0.672
FCFP_2	1	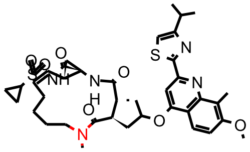 [*]O[*]	0.511
FCFP_2	451847724	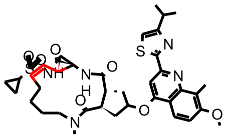 [*]\C=C\C(=[*])[*]	0.225
**Top Features for negative contribution**			
**Fingerprint**	**Bit/Smiles**	**Feature Structure**	**Score**
FCFP_2	136597326	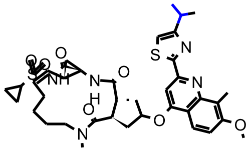 [*]C([*])C	-0.489
FCFP_2	203677720	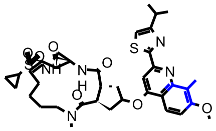 [*]C[c](:[cH]:[*]):[c H]:[*]	-0.406

FCFP_2	565998553	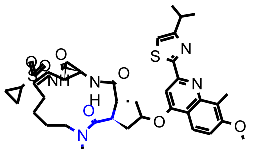 [*]OC(=O)C=[*]	-0.348

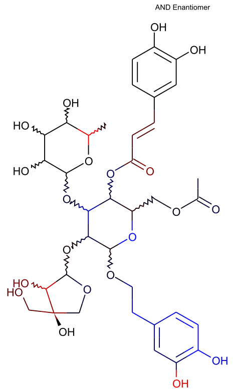

$C_{36}H_{46}O_{20}$
Molecular Weight: 798.73843
ALogP: -0.546
Rotatable Bonds: 16
Acceptors: 20
Donors: 10

Model Prediction

Prediction: 4.282
Unit: g/kg_body_weight
Mahalanobis Distance: 32.165
Mahalanobis Distance p-value: 3.15e-081

Mahalanobis Distance: The Mahalanobis distance (MD) is a generalization of the Euclidean distance that accounts for correlations among the X properties. It is calculated as the distance to the center of the training data. The larger the MD, the less trustworthy the prediction.
Mahalanobis Distance p-value: The p-value gives the fraction of training data with an MD greater than or equal to the one for the given sample, assuming normally distributed data. The smaller the p-value, the less trustworthy the prediciton. For highly non-normal X properties (e.g., fingerprints), the MD p-value is wildly inaccurate.

Structural Similar Compounds

Name	SECALONIC ACID	STREPTOMYCIN	NATAMYCIN
Structure	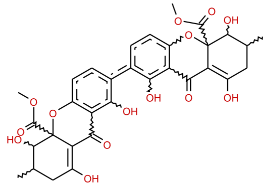	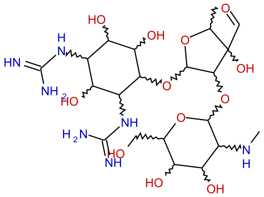	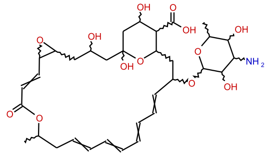
Actual Endpoint (-log C)	4.463	1.81	2.387
Predicted Endpoint (-log C)	3.37815	2.20648	2.63572
Distance	1.590	1.592	1.610
Reference	TXAPA9 48;A14;79	85ARAE 4;35;76/77	TXAPA9 8;97;66

Model Applicability

Unknown features are fingerprint features in the query molecule, but not found in the training set.

1. Num_H_Acceptors out of range. Value: 20. Training min, max, mean, SD: 0, 15, 3.0373, 1.958.
2. OPS PC4 out of range. Value: 13.771. Training min, max, SD, explained variance: -15.374, 12.471, 3.311, 0.0396.
3. OPS PC19 out of range. Value: -7.4333. Training min, max, SD, explained variance: -7.3465, 10.243, 1.885, 0.0128.
4. OPS PC38 out of range. Value: 6.5748. Training min, max, SD, explained variance: -4.9115, 5.821, 1.318, 0.0063.
5. Unknown ECFP_2 feature: 1953003528: [*]C[C@@]1(O)C[*][*]C1[*]
6. Unknown FCFP_6 feature: 16: [*][c](:[*]):[*]
7. Unknown FCFP_6 feature: -549108873: [*]:[c](:[*])O
8. Unknown FCFP_6 feature: 451371068: [*]\C=C\[c](:[*]):[*]
9. Unknown FCFP_6 feature: 1618154665: [*][c](:[*]):[cH]:[c]([*]):[*]
10. Unknown FCFP_6 feature: 74595001: [*][c](:[*]):[c](O):[cH]:[*]

Feature Contribution

Top features for positive contribution			
Fingerprint	Bit/Smiles	Feature Structure	Score

ECFP_6	642810091	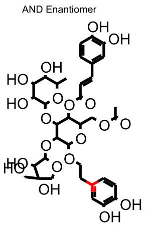<br>[*][c](:[*]):[*]	0.281
ECFP_6	-1074141656	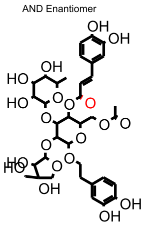<br>[*]=O	0.142
ECFP_6	2019062761	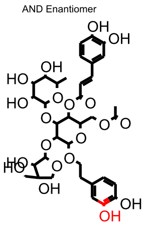<br>[*]:[c](:[*])O	0.138
**Top Features for negative contribution**			
**Fingerprint**	**Bit/Smiles**	**Feature Structure**	**Score**
ECFP_6	683445015	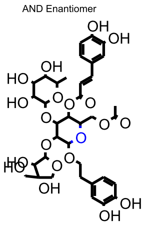<br>[*]O[*]	-0.266
FCFP_6	946589555	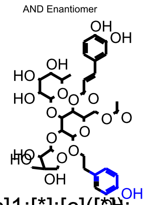<br>[*][c]1:[*]:[c]([*]):[c](O):[cH]:[cH]:1	-0.204

ECFP_6	734603939	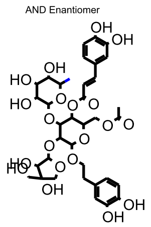 [*]C	-0.201

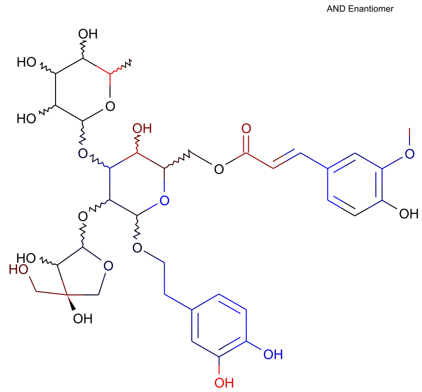

$C_{35}H_{46}O_{19}$
Molecular Weight: 770.72833
ALogP: -0.699
Rotatable Bonds: 15
Acceptors: 19
Donors: 10

Model Prediction

Prediction: 5.717
Unit: g/kg_body_weight
Mahalanobis Distance: 30.745
Mahalanobis Distance p-value: 6.72e-070

Mahalanobis Distance: The Mahalanobis distance (MD) is a generalization of the Euclidean distance that accounts for correlations among the X properties. It is calculated as the distance to the center of the training data. The larger the MD, the less trustworthy the prediction.
Mahalanobis Distance p-value: The p-value gives the fraction of training data with an MD greater than or equal to the one for the given sample, assuming normally distributed data. The smaller the p-value, the less trustworthy the prediciton. For highly non-normal X properties (e.g., fingerprints), the MD p-value is wildly inaccurate.

Structural Similar Compounds

Name	SECALONIC ACID	NATAMYCIN	STREPTOMYCIN
Structure	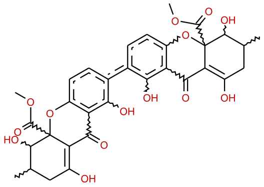	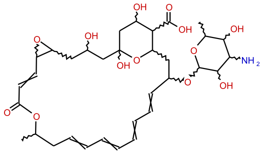	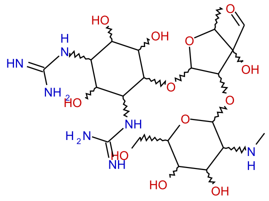
Actual Endpoint (-log C)	4.463	2.387	1.81
Predicted Endpoint (-log C)	3.37815	2.63572	2.20648
Distance	1.468	1.495	1.558
Reference	TXAPA9 48;A14;79	TXAPA9 8;97;66	85ARAE 4;35;76/77

Model Applicability

Unknown features are fingerprint features in the query molecule, but not found in the training set.

1. Num_H_Acceptors out of range. Value: 19. Training min, max, mean, SD: 0, 15, 3.0373, 1.958.
2. OPS PC4 out of range. Value: 13.261. Training min, max, SD, explained variance: -15.374, 12.471, 3.311, 0.0396.
3. Unknown ECFP_2 feature: 1953003528: [*]C[C@@]1(O)C[*][*]C1[*]
4. Unknown FCFP_6 feature: 16: [*][c](:[*]):[*]
5. Unknown FCFP_6 feature: -549108873: [*]:[c](:[*])O
6. Unknown FCFP_6 feature: 1618154665: [*][c](:[*]):[cH]:[c]([*]):[*]
7. Unknown FCFP_6 feature: 74595001: [*][c](:[*]):[c](O):[cH]:[*]
8. Unknown FCFP_6 feature: 451371068: [*]\C=C\[c](:[*]):[*]

Feature Contribution

Top features for positive contribution

Fingerprint	Bit/Smiles	Feature Structure	Score

ECFP_6	642810091	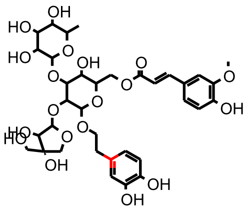 [*][c](:[*]):[*]	0.281
FCFP_6	136627117	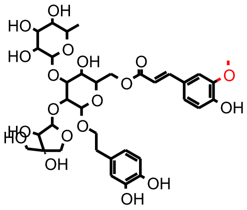 [*]OC	0.170
ECFP_6	-1074141656	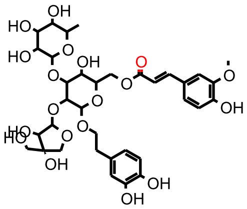 [*]=O	0.142
**Top Features for negative contribution**			
**Fingerprint**	**Bit/Smiles**	**Feature Structure**	**Score**
ECFP_6	683445015	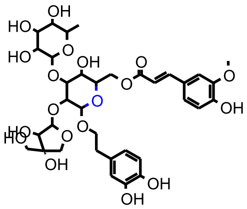 [*]O[*]	-0.266
FCFP_6	1676877079	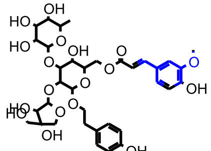 [*]O[c]1:[cH]:[c](C=[*]):[cH]:[*]:[c]:1[*]	-0.254

FCFP_6	946589555	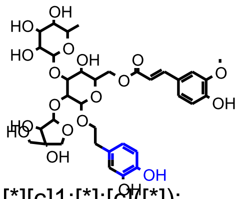[*][c]1:[*]:[c]([*]):[c](O):[cH]:[cH]:1	-0.204

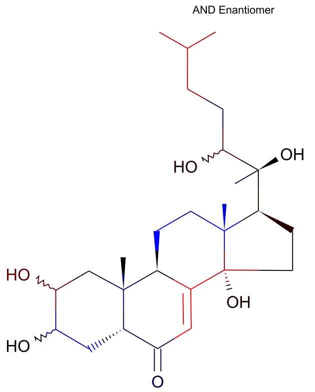

$C_{27}H_{44}O_6$
Molecular Weight: 464.63466
ALogP: 2.489
Rotatable Bonds: 5
Acceptors: 6
Donors: 5

Structural Similar Compounds

Name	PROSCILLARIDIN	CORTISOL; 21-(DIHYDROGEN PHOSPHATE); DISODIUM SALT (Na STRIPPED)	LINCOMYCIN
Structure	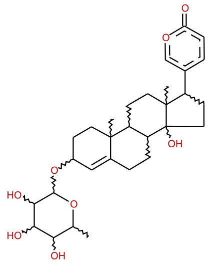	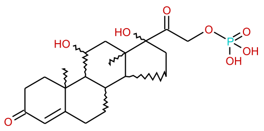	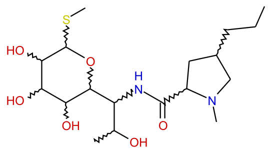
Actual Endpoint (-log C)	3.977	1.861	2.609
Predicted Endpoint (-log C)	2.58837	2.12291	2.17881
Distance	0.626	0.660	0.772
Reference	TXAPA9 20;599;71	YAKUD5 21;2117;79	85ERAY 1;186;78

Model Prediction

Prediction: 10.020
Unit: g/kg_body_weight
Mahalanobis Distance: 22.700
Mahalanobis Distance p-value: 2.33e-019

Mahalanobis Distance: The Mahalanobis distance (MD) is a generalization of the Euclidean distance that accounts for correlations among the X properties. It is calculated as the distance to the center of the training data. The larger the MD, the less trustworthy the prediction.
Mahalanobis Distance p-value: The p-value gives the fraction of training data with an MD greater than or equal to the one for the given sample, assuming normally distributed data. The smaller the p-value, the less trustworthy the prediciton. For highly non-normal X properties (e.g., fingerprints), the MD p-value is wildly inaccurate.

Model Applicability

Unknown features are fingerprint features in the query molecule, but not found in the training set.

1. OPS PC12 out of range. Value: 12.177. Training min, max, SD, explained variance: -7.2298, 11.676, 2.292, 0.0190.
2. Unknown ECFP_2 feature: -483166673: [*]C(=[*])[C@]1(O)C[*][*]C1([*])[*]
3. Unknown ECFP_2 feature: -1049278438: [*]C([*])([*])[C@H]1C[*][*]C1([*])[*]
4. Unknown ECFP_2 feature: 1195714366: [*]C([*])C(C)(O)C([*])[*]
5. Unknown ECFP_2 feature: 80071435: [*]CC(O)C([*])([*])[*]

Feature Contribution

Top features for positive contribution			
Fingerprint	Bit/Smiles	Feature Structure	Score

ECFP_6	642810091	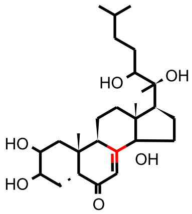<br>[*][c](:[*]):[*]	0.281
ECFP_6	1035165602	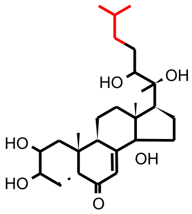<br>[*]CC(C)C	0.172
ECFP_6	-1074141656	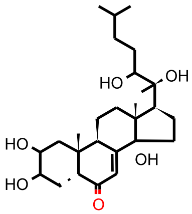<br>[*]=O	0.142
**Top Features for negative contribution**			
**Fingerprint**	**Bit/Smiles**	**Feature Structure**	**Score**
ECFP_6	2106656448	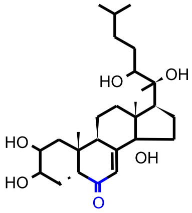<br>[*]C(=O)[*]	-0.352
ECFP_6	412256466	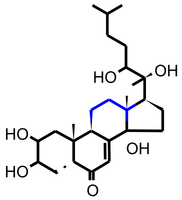<br>[*]CCC([*])([*])[*]	-0.229

ECFP_6	734603939	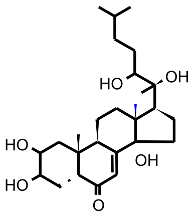 [*]C	-0.201

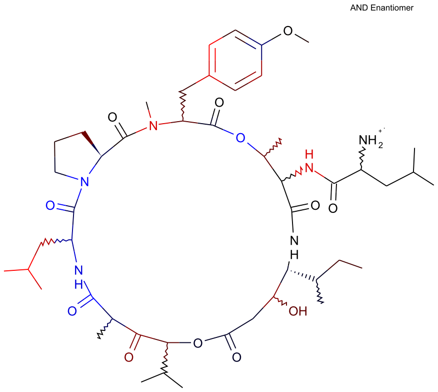

$C_{49}H_{79}N_6O_{12}$
Molecular Weight: 944.18455
ALogP: 3.176
Rotatable Bonds: 13
Acceptors: 12
Donors: 5

Model Prediction

Prediction: 0.274
Unit: g/kg_body_weight
Mahalanobis Distance: 36.965
Mahalanobis Distance p-value: 1.49e-123

Mahalanobis Distance: The Mahalanobis distance (MD) is a generalization of the Euclidean distance that accounts for correlations among the X properties. It is calculated as the distance to the center of the training data. The larger the MD, the less trustworthy the prediction.
Mahalanobis Distance p-value: The p-value gives the fraction of training data with an MD greater than or equal to the one for the given sample, assuming normally distributed data. The smaller the p-value, the less trustworthy the prediciton. For highly non-normal X properties (e.g., fingerprints), the MD p-value is wildly inaccurate.

Structural Similar Compounds

Name	NOVOBIOCIN; SODIUM SALT (Na STRIPPED)	SECALONIC ACID	CLINDAMYCIN-2-PHOSPHATE
Structure	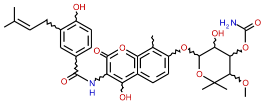	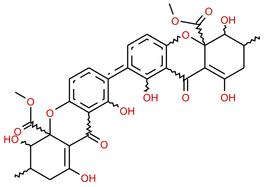	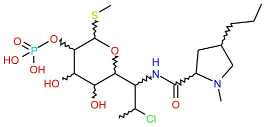
Actual Endpoint (-log C)	2.243	4.463	2.44
Predicted Endpoint (-log C)	2.88962	3.37815	2.51896
Distance	1.090	1.180	1.308
Reference	ARZNAD 8;386;58	TXAPA9 48;A14;79	TXAPA9 27;308;74

Model Applicability

Unknown features are fingerprint features in the query molecule, but not found in the training set.

1. Molecular_Weight out of range. Value: 944.18. Training min, max, mean, SD: 27.025, 835.89, 220.91, 97.79.
2. Unknown ECFP_2 feature: -591526139: [*][NH2+][*]
3. Unknown ECFP_2 feature: 1352327988: [*]C([*])[NH2+]C
4. Unknown ECFP_2 feature: -410102202: [*]CC(N[*])C(=[*])[*]
5. Unknown ECFP_2 feature: 1691770380: [*]NC(C([*])[*])C([*])[*]
6. Unknown ECFP_2 feature: -714938792: [*]C(=[*])C(C)C(=[*])[*]
7. Unknown ECFP_2 feature: 20550775: [*]CC([NH2+][*])C(=[*])[*]
8. Unknown ECFP_2 feature: 866401773: [*][NH2+]C
9. Unknown FCFP_6 feature: 10: [*][NH2+][*]
10. Unknown FCFP_6 feature: 16: [*][c](:[*]):[*]
11. Unknown FCFP_6 feature: 18028531: [*]C([*])[NH2+]C
12. Unknown FCFP_6 feature: -1817836174: [*]CC([NH2+][*])C(=[*])[*]
13. Unknown FCFP_6 feature: 1618154665: [*][c](:[*]):[cH]:[c]([*]):[*]
14. Unknown FCFP_6 feature: 136418580: [*][NH2+]C

Feature Contribution

Top features for positive contribution			
Fingerprint	Bit/Smiles	Feature Structure	Score

ECFP_6	642810091	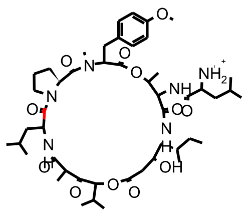 [*][c](:[*]):[*]	0.281
ECFP_6	-1897341097	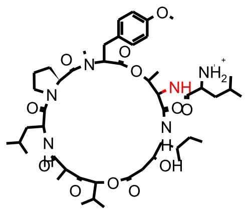 [*]N[*]	0.216
ECFP_6	1035165602	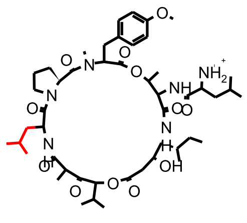 [*]CC(C)C	0.172

Top Features for negative contribution

Fingerprint	Bit/Smiles	Feature Structure	Score
ECFP_6	2106656448	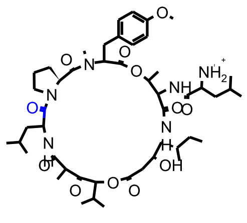 [*]C(=O)[*]	-0.352
ECFP_6	683445015	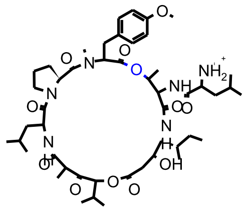 [*]O[*]	-0.266

ECFP_6	-176455838	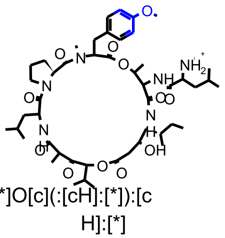 [*]O[c](:[cH]:[*]):[cH]:[*]	-0.257

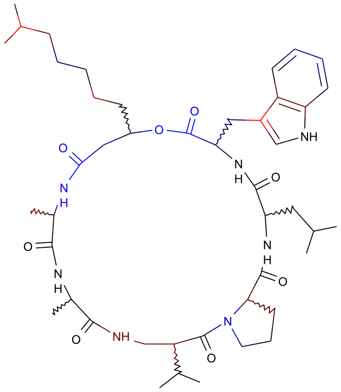

$C_{45}H_{69}N_7O_8$

Molecular Weight: 836.07145

ALogP: 4.986

Rotatable Bonds: 11

Acceptors: 8

Donors: 6

Model Prediction

Prediction: 4.897

Unit: g/kg_body_weight

Mahalanobis Distance: 36.521

Mahalanobis Distance p-value: 2.08e-119

Mahalanobis Distance: The Mahalanobis distance (MD) is a generalization of the Euclidean distance that accounts for correlations among the X properties. It is calculated as the distance to the center of the training data. The larger the MD, the less trustworthy the prediction.
Mahalanobis Distance p-value: The p-value gives the fraction of training data with an MD greater than or equal to the one for the given sample, assuming normally distributed data. The smaller the p-value, the less trustworthy the prediciton. For highly non-normal X properties (e.g., fingerprints), the MD p-value is wildly inaccurate.

Structural Similar Compounds

Name	NOVOBIOCIN; SODIUM SALT (Na STRIPPED)	SECALONIC ACID	VIRIDICATUM TOXIN
Structure	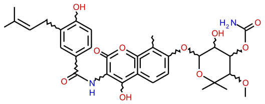	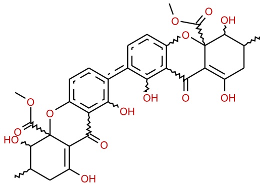	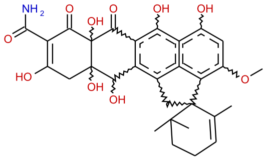
Actual Endpoint (-log C)	2.243	4.463	3.666
Predicted Endpoint (-log C)	2.88962	3.37815	3.0269
Distance	0.949	1.217	1.232
Reference	ARZNAD 8;386;58	TXAPA9 48;A14;79	TXAPA9 24;507;73

Model Applicability

Unknown features are fingerprint features in the query molecule, but not found in the training set.

1. Molecular_Weight out of range. Value: 836.07. Training min, max, mean, SD: 27.025, 835.89, 220.91, 97.79.
2. Unknown ECFP_2 feature: -410102202: [*]CC(N[*])C(=[*])[*]
3. Unknown ECFP_2 feature: 1863041499: [*]NC(C)C(=[*])[*]
4. Unknown FCFP_6 feature: 16: [*][c](:[*]):[*]
5. Unknown FCFP_6 feature: 19: [*]:[nH]:[*]
6. Unknown FCFP_6 feature: 1618184456: [*][c]1:[*]:[*]:[nH]:[cH]:1
7. Unknown FCFP_6 feature: 307448885: [*]:[cH]:[c]1:[nH]:[*]:[*]:[c]:1:[*]
8. Unknown FCFP_6 feature: 2005402822: [*]:[c]1:[*]:[*]:[cH]:[nH]:1
9. Unknown FCFP_6 feature: 1618154665: [*][c](:[*]):[cH]:[c]([*]):[*]

Feature Contribution

Top features for positive contribution			
Fingerprint	Bit/Smiles	Feature Structure	Score

ECFP_6	642810091	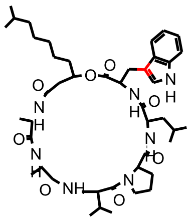 [*][c](:[*]):[*]	0.281
ECFP_6	1035165602	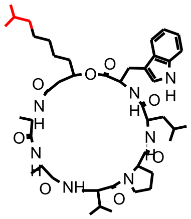 [*]CC(C)C	0.172
ECFP_6	-1074141656	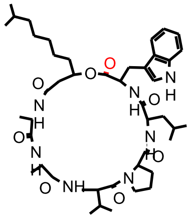 [*]=O	0.142

Top Features for negative contribution

Fingerprint	Bit/Smiles	Feature Structure	Score
ECFP_6	2106656448	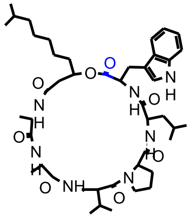 [*]C(=O)[*]	-0.352
ECFP_6	683445015	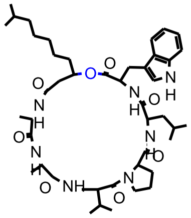 [*]O[*]	-0.266

FCFP_6	566058135	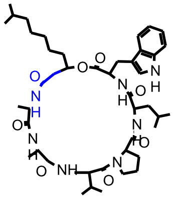 [*]NC(=O)C([*])[*]	-0.216

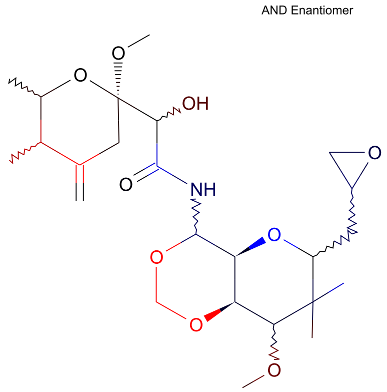

$C_{24}H_{39}NO_9$
Molecular Weight: 485.56776
ALogP: 0.267
Rotatable Bonds: 7
Acceptors: 9
Donors: 2

Model Prediction

Prediction: 0.141
Unit: g/kg_body_weight
Mahalanobis Distance: 26.821
Mahalanobis Distance p-value: 4.68e-042

Mahalanobis Distance: The Mahalanobis distance (MD) is a generalization of the Euclidean distance that accounts for correlations among the X properties. It is calculated as the distance to the center of the training data. The larger the MD, the less trustworthy the prediction.
Mahalanobis Distance p-value: The p-value gives the fraction of training data with an MD greater than or equal to the one for the given sample, assuming normally distributed data. The smaller the p-value, the less trustworthy the prediciton. For highly non-normal X properties (e.g., fingerprints), the MD p-value is wildly inaccurate.

Structural Similar Compounds

Name	INSARIOTOXIN	LASIOCARPINE	FLUOCINONID
Structure	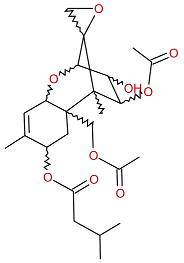	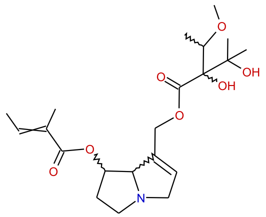	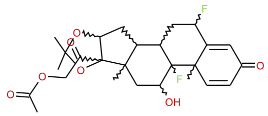
Actual Endpoint (-log C)	5.238	3.573	4.548
Predicted Endpoint (-log C)	3.50309	2.86996	3.335
Distance	0.587	0.634	0.703
Reference	DFSCDX 4;135;83	TXAPA9 17;290;70	NIIRDN 6;694;82

Model Applicability

Unknown features are fingerprint features in the query molecule, but not found in the training set.

1. OPS PC42 out of range. Value: 5.6955. Training min, max, SD, explained variance: -5.0672, 5.4639, 1.239, 0.0055.
2. Unknown ECFP_2 feature: -457637478: [*]NC(O[*])C([*])[*]
3. Unknown ECFP_2 feature: 837844420: [*]CC(O[*])(O[*])C([*])[*]
4. Unknown ECFP_2 feature: 825070174: [*]C(=[*])C(O)C([*])([*])[*]
5. Unknown FCFP_6 feature: 699559848: [*]NC(O[*])C([*])[*]
6. Unknown FCFP_6 feature: 1327594201: [*]CC(O[*])(O[*])C([*])[*]

Feature Contribution

Top features for positive contribution

Fingerprint	Bit/Smiles	Feature Structure	Score
FCFP_6	470041467	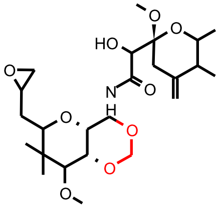 [*]OCO[*]	0.322

ECFP_6	642810091	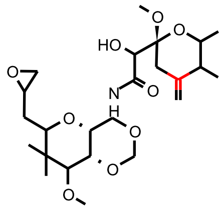 [*][c](:[*]):[*]	0.281
ECFP_6	-1897341097	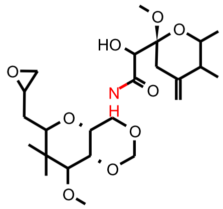 [*]N[*]	0.216

Top Features for negative contribution

Fingerprint	Bit/Smiles	Feature Structure	Score
ECFP_6	683445015	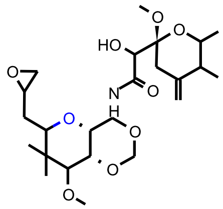 [*]O[*]	-0.266
FCFP_6	566058135	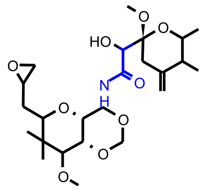 [*]NC(=O)C([*])[*]	-0.216
ECFP_6	734603939	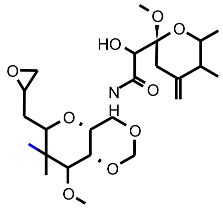 [*]C	-0.201

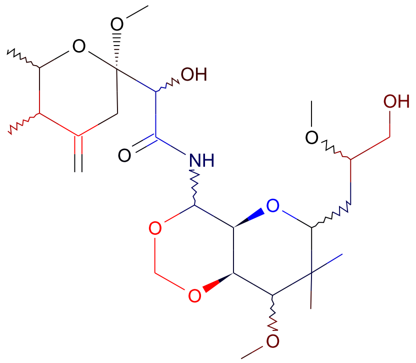

$C_{25}H_{43}NO_{10}$
Molecular Weight: 517.60962
ALogP: -9.1e-002
Rotatable Bonds: 9
Acceptors: 10
Donors: 3

Model Prediction

Prediction: 0.324
Unit: g/kg_body_weight
Mahalanobis Distance: 26.715
Mahalanobis Distance p-value: 2.21e-041

Mahalanobis Distance: The Mahalanobis distance (MD) is a generalization of the Euclidean distance that accounts for correlations among the X properties. It is calculated as the distance to the center of the training data. The larger the MD, the less trustworthy the prediction.
Mahalanobis Distance p-value: The p-value gives the fraction of training data with an MD greater than or equal to the one for the given sample, assuming normally distributed data. The smaller the p-value, the less trustworthy the prediciton. For highly non-normal X properties (e.g., fingerprints), the MD p-value is wildly inaccurate.

Structural Similar Compounds

Name	INSARIOTOXIN	LASIOCARPINE	LENAMPICILLIN .HCl (HCl STRIPPED)
Structure	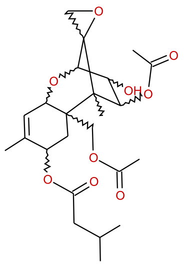	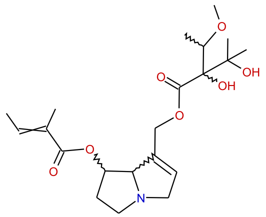	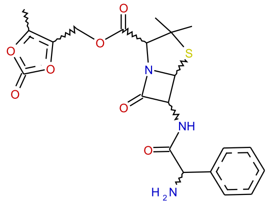
Actual Endpoint (-log C)	5.238	3.573	1.664
Predicted Endpoint (-log C)	3.50309	2.86996	2.37593
Distance	0.755	0.755	0.782
Reference	DFSCDX 4;135;83	TXAPA9 17;290;70	NKRZAZ 32(Suppl

Model Applicability

Unknown features are fingerprint features in the query molecule, but not found in the training set.

1. OPS PC44 out of range. Value: 6.5592. Training min, max, SD, explained variance: -4.6697, 6.3674, 1.209, 0.0053.
2. Unknown ECFP_2 feature: -457637478: [*]NC(O[*])C([*])[*]
3. Unknown ECFP_2 feature: 837844420: [*]CC(O[*])(O[*])C([*])[*]
4. Unknown ECFP_2 feature: 825070174: [*]C(=[*])C(O)C([*])([*])[*]
5. Unknown FCFP_6 feature: 699559848: [*]NC(O[*])C([*])[*]
6. Unknown FCFP_6 feature: 1327594201: [*]CC(O[*])(O[*])C([*])[*]

Feature Contribution

Top features for positive contribution			
Fingerprint	Bit/Smiles	Feature Structure	Score
FCFP_6	470041467	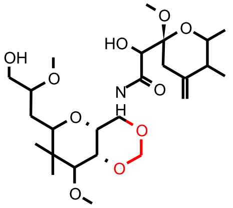 [*]OCO[*]	0.322

ECFP_6	642810091	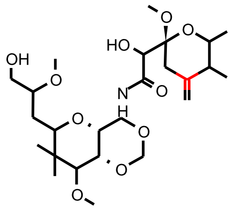 [*][c](:[*]):[*]	0.281
ECFP_6	-1897341097	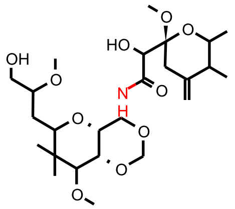 [*]N[*]	0.216
**Top Features for negative contribution**			
**Fingerprint**	**Bit/Smiles**	**Feature Structure**	**Score**
ECFP_6	683445015	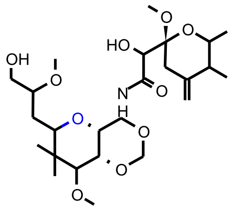 [*]O[*]	-0.266
FCFP_6	566058135	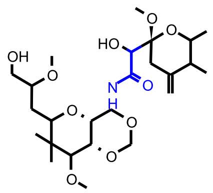 [*]NC(=O)C([*])[*]	-0.216
ECFP_6	734603939	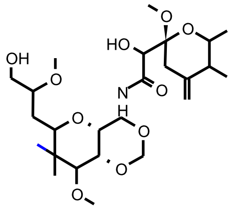 [*]C	-0.201

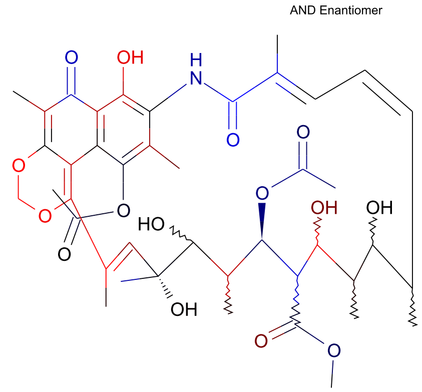

$C_{42}H_{53}NO_{15}$
Molecular Weight: 811.86791
ALogP: 2.745
Rotatable Bonds: 6
Acceptors: 15
Donors: 6

Model Prediction

Prediction: 0.166
Unit: g/kg_body_weight
Mahalanobis Distance: 31.033
Mahalanobis Distance p-value: 3.78e-072

Mahalanobis Distance: The Mahalanobis distance (MD) is a generalization of the Euclidean distance that accounts for correlations among the X properties. It is calculated as the distance to the center of the training data. The larger the MD, the less trustworthy the prediction.
Mahalanobis Distance p-value: The p-value gives the fraction of training data with an MD greater than or equal to the one for the given sample, assuming normally distributed data. The smaller the p-value, the less trustworthy the prediciton. For highly non-normal X properties (e.g., fingerprints), the MD p-value is wildly inaccurate.

Structural Similar Compounds

Name	SECALONIC ACID	NOVOBIOCIN; SODIUM SALT (Na STRIPPED)	NATAMYCIN
Structure	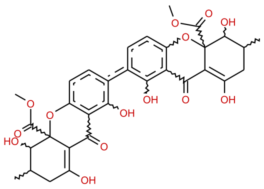	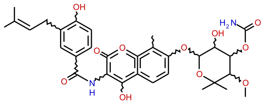	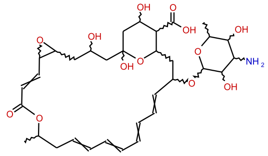
Actual Endpoint (-log C)	4.463	2.243	2.387
Predicted Endpoint (-log C)	3.37815	2.88962	2.63572
Distance	0.761	1.028	1.075
Reference	TXAPA9 48;A14;79	ARZNAD 8;386;58	TXAPA9 8;97;66

Model Applicability

Unknown features are fingerprint features in the query molecule, but not found in the training set.

1. OPS PC22 out of range. Value: -6.4102. Training min, max, SD, explained variance: -5.4726, 10.66, 1.747, 0.0110.
2. Unknown ECFP_2 feature: -40769921: [*]C([*])[C@](C)(O)C=[*]
3. Unknown ECFP_2 feature: 824611088: [*]C([*])C(C)C=[*]
4. Unknown ECFP_2 feature: 470857763: [*]C(=CC=[*])[*]
5. Unknown FCFP_6 feature: 16: [*][c](:[*]):[*]
6. Unknown FCFP_6 feature: -549108873: [*]:[c](:[*])O
7. Unknown FCFP_6 feature: -1678275541: [*]C(=C(C(=[*])[*])[c](:[*]):[*])[*]
8. Unknown FCFP_6 feature: 74595001: [*][c](:[*]):[c](O):[cH]:[*]

Feature Contribution

Top features for positive contribution

Fingerprint	Bit/Smiles	Feature Structure	Score

FCFP_6	470041467	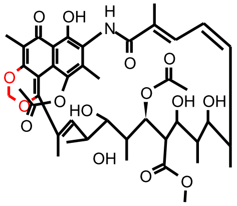 [*]OCO[*]	0.322
ECFP_6	642810091	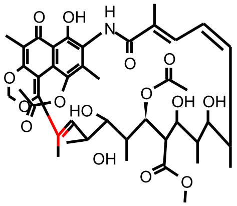 [*][c](:[*]):[*]	0.281
FCFP_6	436915834	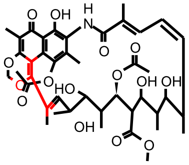 [*]OC(=C([*])[*])C(=[*])[*]	0.184

Top Features for negative contribution

**Fingerprint**	**Bit/Smiles**	**Feature Structure**	**Score**
ECFP_6	2106656448	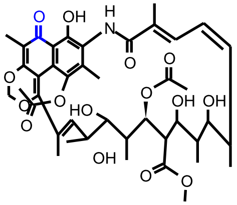 [*]C(=O)[*]	-0.352
ECFP_6	683445015	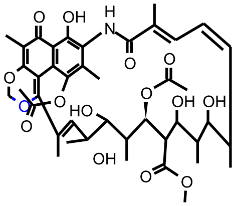 [*]O[*]	-0.266

FCFP_6	566058135	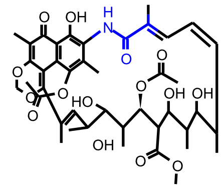 [*]NC(=O)C([*])[*]	-0.216

Simeprevir

TOPKAT_Rat_Oral_LD50

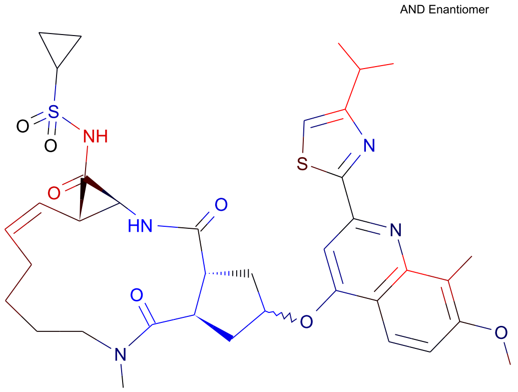

$C_{38}H_{47}N_5O_7S_2$

Molecular Weight: 749.93907

ALogP: 4.796

Rotatable Bonds: 8

Acceptors: 9

Donors: 2

Structural Similar Compounds

Name	ANAPREL	RESERPINE	PHOSPHOROTHIOIC ACID; O;O'-(SULFONYLDI-p-PHENYLENE) O;O;O';O'-TETRAMETHYL ESTER
Structure	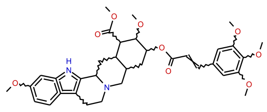	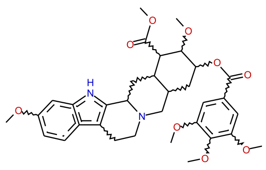	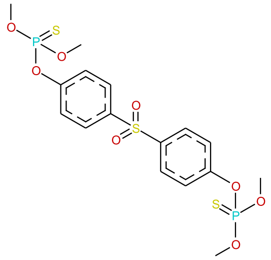
Actual Endpoint (-log C)	2.803	3.161	2.397
Predicted Endpoint (-log C)	2.99154	2.72801	3.8515
Distance	1.032	1.042	1.057
Reference	NIIRDN 6;898;82	PSSCBG 11;555;80	TXAPA9 21;315;72

Model Prediction

Prediction: 0.209

Unit: g/kg_body_weight

Mahalanobis Distance: 37.613

Mahalanobis Distance p-value: 1.1e-129

Mahalanobis Distance: The Mahalanobis distance (MD) is a generalization of the Euclidean distance that accounts for correlations among the X properties. It is calculated as the distance to the center of the training data. The larger the MD, the less trustworthy the prediction.
Mahalanobis Distance p-value: The p-value gives the fraction of training data with an MD greater than or equal to the one for the given sample, assuming normally distributed data. The smaller the p-value, the less trustworthy the prediciton. For highly non-normal X properties (e.g., fingerprints), the MD p-value is wildly inaccurate.

Model Applicability

Unknown features are fingerprint features in the query molecule, but not found in the training set.

1. All properties and OPS components are within expected ranges.
2. Unknown ECFP_2 feature: -309676563: [*]N[C@]1(C[C@H]1[*])C(=[*])[*]
3. Unknown ECFP_2 feature: -1049290660: [*]C1([*])C[C@H]1C=[*]
4. Unknown ECFP_2 feature: 2121941848: [*]NS(=O)(=O)C1[*][*]1
5. Unknown FCFP_6 feature: 16: [*][c](:[*]):[*]
6. Unknown FCFP_6 feature: 1747237384: [*][c](:[*]):n:[c](:[*]):[*]
7. Unknown FCFP_6 feature: 690511177: [*]:[cH]:[c](:n:[*])[c](:[*]):[*]
8. Unknown FCFP_6 feature: 1618154665: [*][c](:[*]):[cH]:[c]([*]):[*]
9. Unknown FCFP_6 feature: 690481386: [*]:[c](:[*])[c]1:n:[*]:[*]:s:1
10. Unknown FCFP_6 feature: 1018942292: [*]NS(=O)(=O)C1[*][*]1

Feature Contribution

Top features for positive contribution

Fingerprint	Bit/Smiles	Feature Structure	Score

FCFP_6	1186303932	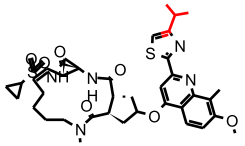 [*]:[c](:[*])C(C)C	0.375
ECFP_6	642810091	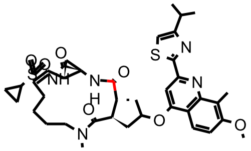 [*][c](:[*]):[*]	0.281
ECFP_6	-1897341097	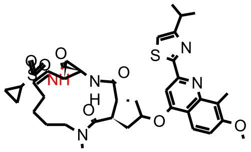 [*]N[*]	0.216

Top Features for negative contribution

**Fingerprint**	**Bit/Smiles**	**Feature Structure**	**Score**
ECFP_6	2106656448	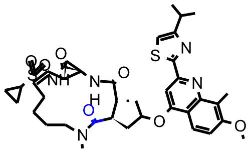 [*]C(=O)[*]	-0.352
ECFP_6	2077607946	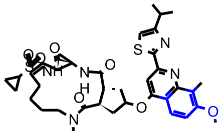 [*]O[c]1:[cH]:[cH]:[c](:[*]):[*]:[c]:1[*]	-0.252

ECFP_6	655739385	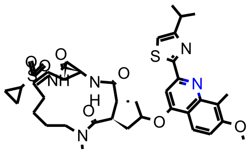 [*]:n:[*]	-0.239
